# Full-spectrum extract from *Cannabis sativa* DKJ127 for chronic low back pain: a phase 3 randomized placebo-controlled trial

**DOI:** 10.1038/s41591-025-03977-0

**Published:** 2025-09-29

**Authors:** Matthias Karst, Winfried Meissner, Sabine Sator, Jens Keßler, Volker Schoder, Winfried Häuser

**Affiliations:** 1 Department of Anesthesiology and Intensive Care Medicine, Pain Clinic, Hannover Medical School, Hannover, Germany; 2 Department of Anesthesiology and Intensive Care Medicine, Jena University Hospital, Friedrich Schiller University Jena, Jena, Germany; 3 Clinical Department for Pain Medicine, Medical University of Vienna, Vienna, Austria; 4 Medical Faculty Heidelberg, Department of Anesthesiology, Pain Medicine Section, Heidelberg University, Heidelberg, Germany; 5 Metronomia Clinical Research GmbH, Munich, Germany; 6 Department of Psychosomatic Medicine and Psychotherapy, Technical University of Munich, Munich, Germany; 7 Medical Center Pain Medicine and Mental Health, Saarbrücken, Germany

**Keywords:** Translational research, Randomized controlled trials, Diseases

## Abstract

Chronic low back pain (CLBP) affects over half a billion people worldwide. Current pharmacologic treatments offer limited efficacy and carry substantial risks, warranting the development of safe and effective alternatives. This multicenter, randomized, placebo-controlled phase 3 trial evaluated the efficacy and safety of VER-01 in CLBP. It enrolled 820 adults with CLBP (VER-01, *n* = 394; placebo, *n* = 426) and included a double-blind 12-week treatment phase (phase A), a 6-month open-label extension (phase B), followed by either a 6-month continuation (phase C) or randomized withdrawal (phase D). The primary endpoint of phase A was a change in mean numeric rating scale (NRS) pain intensity, with a change in total neuropathic pain symptom inventory (NPSI) score as a key secondary endpoint in participants with a neuropathic pain component (PainDETECT > 18). The primary endpoint for phase D was time to treatment failure. The study met its primary endpoint in phase A, with a mean pain reduction of −1.9 NRS points in the VER-01 group (mean difference (MD) versus placebo = −0.6, 95% confidence interval (CI) = −0.9 to −0.3; *P* < 0.001). Pain further decreased to −2.9 NRS points in phase B, with effects sustained through phase C. The study also met its key secondary endpoint of phase A, with a mean NPSI decrease of −14.4 (standard error, 3.3) points from baseline in the VER-01 arm (MD versus placebo = −7.3, 95% CI = −13.2 to −1.3; *P* = 0.017). Although phase D did not meet its primary endpoint (hazard ratio = 0.75, 95% CI = 0.44–1.27; *P* = 0.288), pain increased significantly more with placebo upon withdrawal (MD = 0.5, 95% CI = 0.0–1.0; *P* = 0.034). In phase A, the incidence of adverse events—mostly mild to moderate and transient—was higher with VER-01 than with placebo (83.3% versus 67.3%; *P* < 0.001). VER-01 was well-tolerated, with no signs of dependence or withdrawal. VER-01 shows potential as a new, safe and effective treatment for CLBP. ClinicalTrials.gov registration: NCT04940741.

## Abstract (web summary)

A phase 3 trial found that VER-01, a full-spectrum cannabis extract, reduces chronic low back pain, improves physical function and sleep and shows no signs of dependence or withdrawal.

## Main

With over half a billion prevalent cases in 2020, low back pain is globally the leading cause for work loss, disability and reduced quality of life across all ages and in both sexes^1,2^. Low back pain is a mixed pain condition with nociceptive, nociplastic and neuropathic pain characteristics^3^. When pain persists for more than 3 months, it is defined as chronic low back pain (CLBP). CLBP is often associated with severe impairments in sleep quality and physical function, which further contribute to the overall disease burden^4,5^. The treatment of CLBP involves a multimodal treatment approach combining pharmacological and nonpharmacological interventions, the latter including physical activity, exercise and physiotherapy^6–10^.

Pharmacological treatment options include the short-term use of nonsteroidal anti-inflammatory drugs (NSAIDs). However, NSAIDs are not suitable for long-term treatment due to severe side effects, including gastrointestinal ulcers, bleeding and an increased risk for cardiovascular events^11,12^. For patients requiring long-term analgesic treatment, opioids are frequently used, despite severe side effects and safety concerns. Approximately 20% of patients on long-term therapy experience opioid abuse, dependence, tolerance development and withdrawal symptoms^13^. The widespread use of opioids for CLBP combined with their high risk for dependence, misuse and fatal overdoses has substantially contributed to the global opioid epidemic, resulting in hundreds of thousands of deaths worldwide^14,15^. Accordingly, several clinical practice guidelines now advise against the use of opioids^6,7^, and there is widespread consensus among healthcare professionals, patient organizations and regulators on the urgent need to develop new nonaddictive analgesics for the short- and long-term treatment of CLBP with a superior safety profile. The limitations of existing treatments and the stagnation in the development of new analgesics have fueled growing public and scientific interest in the use of cannabis-based medicines for the management of chronic pain. However, the quality of evidence supporting the clinical use of cannabis-based products remains low, primarily due to small sample sizes, short treatment durations, inconsistent dosing regimens, heterogeneous outcome measures and variability across cannabis-based preparations^16–19^.

As naturally occurring botanical substances, cannabis plants exhibit substantial heterogeneity. Variability in the botanical raw material and manufacturing processes contributes to a substantial variety of product compositions, including levels of bioactive constituents^20,21^. Consequently, findings obtained with one cannabis extract cannot be extrapolated to others without appropriate comparative data. To ensure reproducible results, it is therefore essential to adequately characterize the investigational product and establish consistency across batches.

The investigational product VER-01 was comprehensively characterized using chromatographic and spectrometric methods to quantify cannabinoids, terpenes, flavonoids, carotenes, phytosterols, vitamins and fats, and chromatographic fingerprinting confirmed batch-to-batch consistency across multiple lots of VER-01. By providing a large-scale, placebo-controlled phase 3 trial of adequate duration using a chemically well-defined, full-spectrum cannabis extract in CLBP, this study addresses a critical gap in the clinical research of cannabis-based pharmacotherapy in chronic pain.

## Results

### Patient disposition and baseline characteristics

**Fig. 1:**
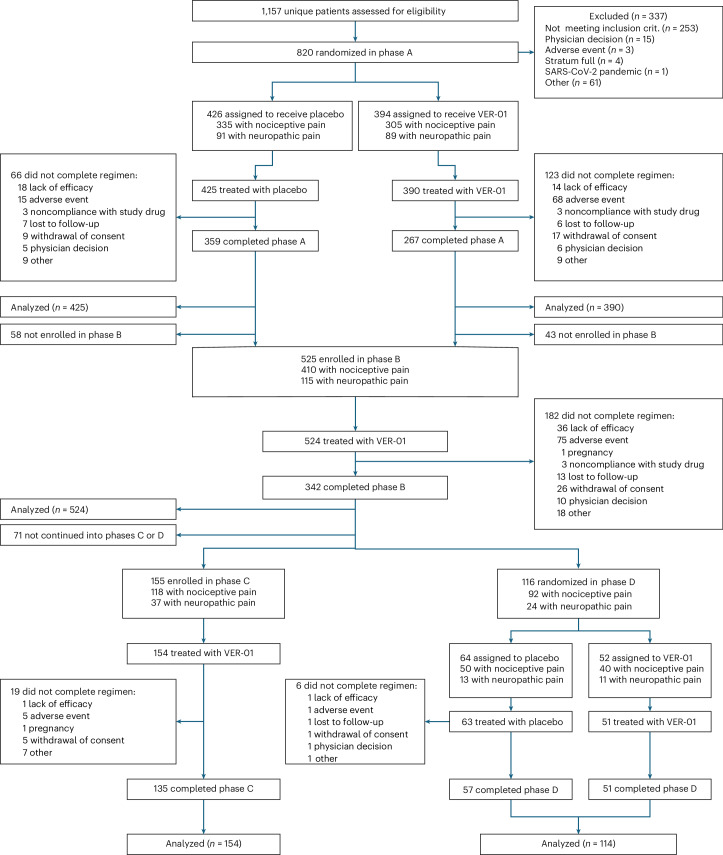
CONSORT chart of the trial. The efficacy, safety and tolerability analyses included all randomized participants who received at least one dose of the study medication during the respective study phase.

**Fig. 2:**
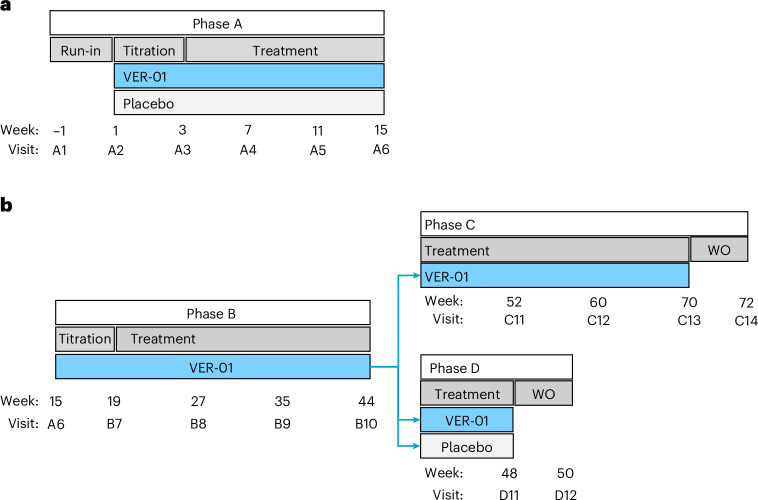
Trial design. **a**, The trial design of phase A. **b**, The trial design of phases B–D. WO, washout.

Between 7 July 2021 and 30 June 2023, 1,157 patients were assessed for eligibility and 820 participants were randomly assigned to VER-01 (*n* = 394) or placebo (*n* = 426; Fig. 1). A total of 525 participants continued to phase B, 155 continued to phase C and 116 participants were randomly assigned to VER-01 (*n* = 52) or placebo (*n* = 64) in phase D (Figs. 1 and 2). A total of 815 participants were included in the efficacy analysis of phase A, whereas 116 participants were included in that of phase D. The last visit of the study was on 26 March 2024.

Overall, the two groups in phase A were balanced with respect to baseline characteristics (Table 1). A total of 180 participants (22.0%) had a neuropathic pain component, and 193 (23.5%) were suffering from severe pain (numeric rating scale (NRS) ≥ 7) at baseline. A total of 461 participants (56.6%) were female, with a mean age of 52 years and a mean body mass index (BMI) of 29 kg m^−2^, showing no notable differences between the two groups. Baseline characteristics of participants with a neuropathic pain component were consistent across both treatment groups and matched those of all participants. Additionally, the demographics and baseline characteristics of participants in phases B, C and D were similar to those in phase A (Extended Data Tables 1–3). The most frequent concurrent diseases were hypertension (35.3%) and obesity (32.0%). In total, 99% of patients reported prior use of analgesics, with nonsteroidal anti-inflammatory and antirheumatic drugs being the most frequently reported medication (96.7%). The proportion of participants with at least two prior optimized analgesic therapies was balanced between arms, with 95.4% in the VER-01 arm and 94.8% in the placebo arm.

**Table 1:** Demographics and other trial baseline characteristics for participants starting phase A

Characteristics	VER-01 (*n* = 390)	Placebo (*n* = 425)
Age (year), mean (s.d.)	52.3 (14.2)	52.2 (13.4)
Female sex,^a^ *n* (%)	224 (57.4)	237 (55.8)
Race, *n* (%)		
White	373 (95.6)	419 (98.6)
Other	17 (4.4)	6 (1.4)
BMI at baseline (kg m^−^^2^), mean (s.d.)	28.88 (6.34)	29.01 (5.84)
Hypertension, *n* (%)	148 (37.9)	140 (32.9)
Type 2 diabetes, *n* (%)	43 (11.0)	36 (8.5)
Obesity, *n* (%)	115 (29.5)	146 (34.4)
Concomitant nondrug therapies, *n* (%)	117 (30.0)	115 (27.1)
Concomitant nondrug therapies for CLBP, *n* (%)	96 (24.6)	92 (21.6)
Prior nondrug therapy for CLBP, *n* (%)	38 (9.7)	37 (8.7)
PainDETECT baseline score, mean (s.d.)	12.4 (6.9)	13.0 (6.7)
PainDETECT baseline score >18, *n* (%)	88 (22.6)	91 (21.4)
Severe pain (NRS ≥ 7) at baseline, *n* (%)	100 (25.6)	93 (21.9)
Received ≥2 prior optimized therapies, *n* (%)	372 (95.4)	403 (94.8)
NRS pain baseline score, mean (s.d.)	6.1 (1.2)	6.0 (1.2)
NRS sleep baseline score, mean (s.d.)	5.2 (1.8)	5.2 (1.8)
RMDQ total score, mean (s.d.)	10.1 (4.7)	9.6 (4.9)
MOS-SS		
Sleep problems index I, mean (s.d.)	42.82 (9.23)	43.05 (9.57)
Sleep problems index II, mean (s.d.)	42.26 (8.82)	42.29 (9.19)
SF-36		
Mental component summary, mean (s.d.)	51.8 (10.8)	52.5 (10.4)
Physical component summary, mean (s.d.)	36.1 (7.2)	37.2 (7.2)

^a^Self-reported.

### Efficacy

Pain intensity at baseline was moderate to severe in both groups, with a mean NRS pain intensity of 6.1 (s.d. = 1.2) for VER-01 and 6.0 (s.d. = 1.2) for placebo.

**Fig. 3:**
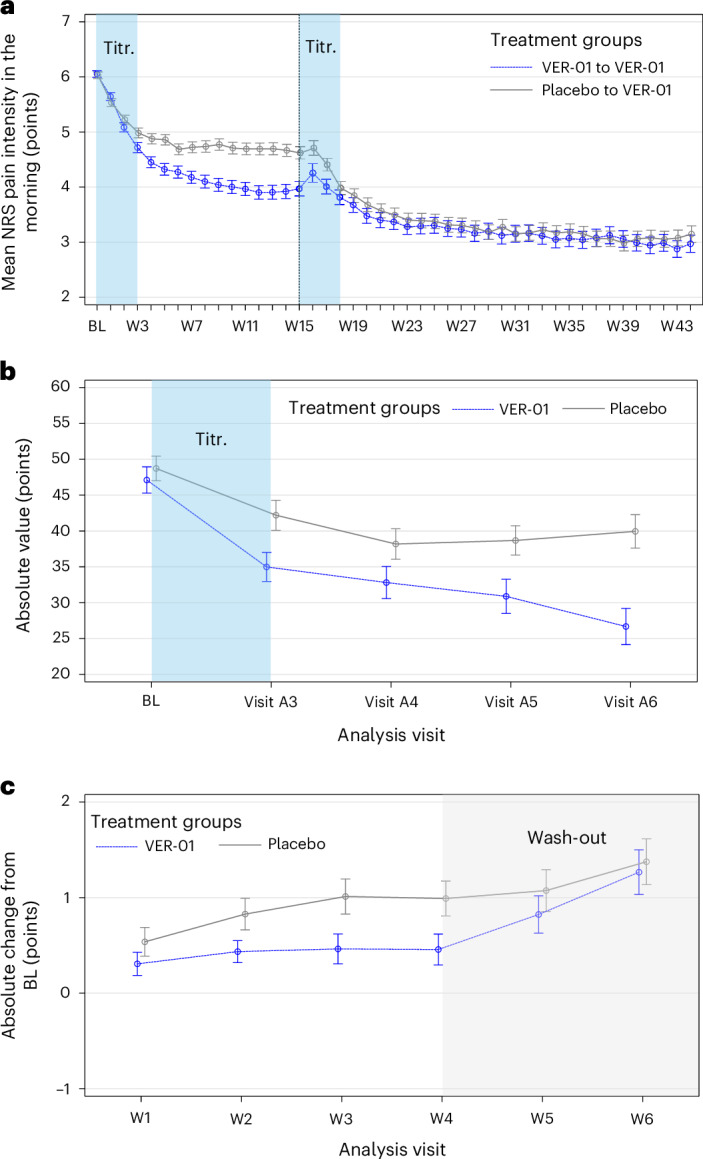
Effect of VER-01 on NRS pain and NPSI scores as compared with Placebo. **a**, Mean NRS pain intensity in phases A and B, as measured on an 11-point NRS for each study week of the placebo-controlled study phase A (VER-01, *n* = 390; placebo, *n* = 425) and the open-label phase B (VER-01, *n* = 524). **b**, Mean NPSI total scores for the subgroup of participants with a neuropathic pain component during phase A (VER-01, *n* = 88; placebo, *n* = 91). **c**, Change in mean NRS pain intensity after randomized withdrawal in phase D (VER-01, *n* = 51; placebo, *n* = 63). All bars display the range from mean ± s.e. of the mean. BL, baseline; W, week; Titr., titration.

The study met its primary endpoint in phase A. Mean pain intensity significantly decreased from baseline by −1.9 (s.e. = 0.2) NRS points in the VER-01 arm compared to −1.4 (s.e. = 0.2) in the placebo arm. VER-01 demonstrated a greater pain reduction compared to placebo with a mean difference (MD) of −0.6 (95% confidence interval (CI) = −0.9 to −0.3; *P* < 0.001). The difference between VER-01 and placebo was significantly in favor of VER-01 in every single study week of the 12-week treatment phase, showing consistent improvement over time (Fig. 3a). Sensitivity analyses using both last observation carried forward (LOCF) and baseline observation carried forward (BOCF) imputation yielded similar results to the primary analysis and remained statistically significant (Supplementary Tables 10 and 11). Post hoc analysis revealed no statistical interaction between treatment and sex (Supplementary Table 4).

The difference between VER-01 and placebo was even more pronounced in participants with a neuropathic pain component (MD = −1.5, 95% CI = −2.2 to −0.9; *P* < 0.001). Moreover, post hoc analyses revealed particularly pronounced effects of VER-01 in participants with severe pain (MD = −1.0, 95% CI = −1.8 to −0.1; *P* = 0.011).

The study also met its key secondary endpoint in phase A, with significant improvements in neuropathic symptoms (as measured by the Neuropathic Pain Symptom Inventory (NPSI)) among participants with a PainDETECT score >18 at baseline. The mean NPSI total scores at baseline were comparable between groups, with 47.1 (s.d. = 17.2) for VER-01 and 48.7 (s.d. = 16.4) for placebo. The mean NPSI total score decreased by −14.4 (s.e. = 3.3) points from baseline in the VER-01 arm compared to −7.2 (s.e. = 2.8) in the placebo arm, with an MD of −7.3 (95% CI = −13.2 to −1.3; *P* = 0.017). The difference between VER-01 and placebo was significantly in favor of VER-01 for every single visit of the treatment phase, showing consistent improvement over time (Fig. 3b). Compared to placebo, VER-01 reduced superficial spontaneous pain (MD = −1.3, 95% CI = −2.4 to −0.3; *P* = 0.015), deep spontaneous pain (MD = −1.2, 95% CI = −2.2 to −0.3; *P* = 0.001), evoked pain (MD = −1.14, 95% CI = −1.9 to −0.4; *P* = 0.003) and abnormal sensations (MD = −1.29, 95% CI = −2.2 to −0.4; *P* = 0.006).

The results of the primary and key secondary endpoints were further supported by all secondary efficacy endpoints (Table 2). The rate of participants with a ≥30% pain reduction was significantly higher for VER-01 compared to placebo (54.1% versus 39.5%), resulting in a number needed to treat to benefit (NNTB) of 6.8 (95% CI = 4.42–15.05; *P* < 0.001). Similarly, the rate of participants with a ≥50% pain reduction (32.2% versus 22.8%; *P* = 0.010) and a ≥2-point pain reduction (46.9% versus 35.6%; *P* = 0.001) was significantly higher in the VER-01 arm. Moreover, participants in the VER-01 arm took only about half the amount of rescue medication compared to participants in the placebo arm (mean (s.d.) = 10.5 (14.2) versus 18.3 (53.8) ibuprofen tablets; *P* < 0.001).

**Table 2:** Secondary endpoints of phase A

Parameters	VER-01 (*n* = 390)	Placebo (*n* = 425)	Difference between VER-01 and placebo	Odds ratio (95% CI)	*P* value
≥30% pain responder at week 15 (%)	54.1	39.5	14.6	1.7 (1.22–2.26)	<0.001^c^
≥50% pain responder at week 15 (%)	32.2	22.8	9.4	1.6 (1.11–2.22)	0.010^c^
Cumulative dose (Ibuprofen tablets) of rescue medication (s.d.)	10.5 (14.2)	18.3 (53.8)	−7.8	–	<0.001^d^
Cumulative dose (Ibuprofen in g) of rescue medication (s.d.)	8.4 (11.4)	14.6 (43.1)	−6.2	–	<0.001^d^
Mean CFB to week 15 in NRS sleep quality (s.d.)	−2.2 (2.2)	−1.5 (2.0)	−0.7	–	<0.001^e^
MOS-SS—mean CFB to visit A6 in sleep problems index I (s.d.)	6.5 (9.1)	4.1 (8.8)	2.4	–	<0.001^e^
MOS-SS—mean CFB to visit A6 in sleep problems index II (s.d.)	6.8 (8.9)	4.5 (8.3)	2.3	–	0.001^e^
RMDQ total score—mean CFB to visit A6	−3.1 (4.0)	−2.0 (4.1)	−1.1	–	<0.001^e^
≥30% RMDQ responder at visit A6 (%)	51.7	42.2	9.5	1.5 (1.06–2.01)	<0.001^f^
PGIC—participants with improvement of symptoms^a^ at visit A6 (%)	45.1	23.4	21.7	–	<0.001^c^
SF-36—mean CFB to visit A6 for physical component summary (s.d.)	5.9 (6.8)	3.7 (7.0)	2.1	–	<0.001^e^
SF-36—participants with improvement in quality of life^b^ at visit A6 (%)	46.1	31.2	14.9	–	<0.001^c^

^a^Improvement of symptoms—very much better and much better.

^b^Improvement in quality of life—somewhat better now than 1 year ago, much better now than 1 year ago.

^c^*P* value for the two-sided chi-squared test testing the null hypothesis that responder status and treatment group are independent.

^d^*P* value from the two-sided Wilcoxon test for two independent samples testing the null hypothesis that the distributions are equal in both treatment groups (post hoc analysis).

^e^*P* value for the two-sided *t* test for independent samples testing the null hypothesis that the mean CFB is equal in both treatment groups.

^f^*P* value for the two-sided chi-squared test testing the null hypothesis that responder status and treatment group are independent (post hoc analysis).

Additionally, participants in the VER-01 group reported significant improvements in both sleep quality and physical function. Sleep quality improved by −2.2 NRS points in the VER-01 arm compared to −1.5 in the placebo arm, with an MD of −0.7 NRS points (95% CI, −1.0 to −0.3; *P* < 0.001). Change from baseline (CFB) in Roland Morris Disability Questionnaire (RMDQ) total score was also significantly in favor of VER-01, with −3.1 (4.0) versus −2.0 (4.1) points (MD = −1.1, 95% CI = −1.8 to −1.1; *P* < 0.001). Post hoc analysis revealed that 51.7% of participants in the VER-01 arm achieved an improvement of the RMDQ score by at least 30% from baseline compared to 42.2% in the placebo arm (*P* = 0.019).

Improvements in pain intensity, sleep quality and physical function were associated with elevated quality of life and a positive global impression of change. The physical health component summary (PCS) score of the short-form 36 (SF-36) improved by 5.9 points in the VER-01 arm compared to 3.7 in the placebo arm, with an MD of 2.1 (95% CI = 1.0–3.2; *P* < 0.001). In total, 45.1% of participants in the VER-01 group reported an improvement in symptoms on the patient global impression of change (PGIC) scale compared to 23.4% in the placebo group, resulting in an NNTB of 4.6 (95% CI = 3.43–6.99; *P* < 0.001).

**Extended Data Fig. 1:**
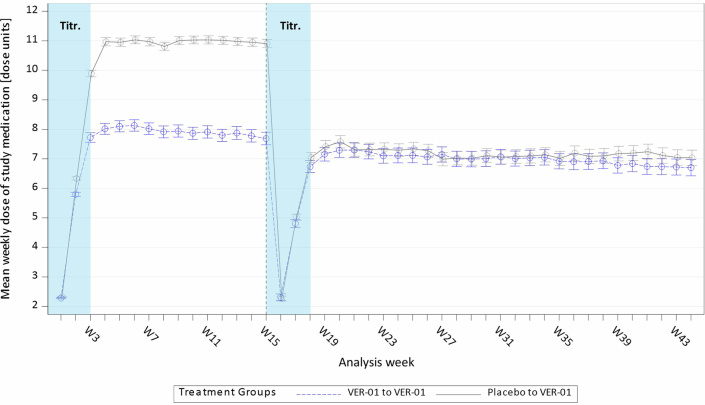
Weekly mean of daily dose of VER-01 and placebo in phase A and VER-01 in phase B. Bars display the range from mean ± standard error of the mean. Titr., titration.

Participants experienced further reductions in NRS pain intensity during the 6-month treatment period of phase B, with a decrease of 3 points compared to the phase A baseline (s.d. = 2.1; Fig. 3a). The percentage of participants experiencing a ≥30% reduction in pain further increased to 73.9%, while the percentage for those achieving a ≥50% pain reduction increased to 51.8%. In addition, participants reported further improvements in physical function, sleep quality and quality of life (Extended Data Table 4). Participants who completed phase B and participated in phase C were able to maintain their pain reduction over an additional 6-month period (Supplementary Fig. 1). There were no signs of diminishing efficacy or dose escalation over time (Extended Data Fig. 1 and Extended Data Tables 5–8).

Time to treatment failure did not differ significantly between VER-01 and placebo in phase D (hazard ratio = 0.75, 95% CI = 0.44–1.27; *P* = 0.288), with median times of 22 days for VER-01 and 11 days for placebo. However, following withdrawal, participants receiving placebo experienced a significant pain increase from the phase D baseline compared to VER-01 (MD = 0.5, s.d. = 0.24; *P* = 0.034; Fig. 3d).

### Tolerability and safety

In phase A, treatment-emergent adverse events (TEAEs) were reported by 83.3% of participants in the VER-01 group, compared to 67.3% in the placebo group (*P* < 0.001). The rate of serious adverse events (SAEs) was comparable between VER-01 and placebo (6.2% versus 6.8%; *P* = 0.699; Table 3). The most common TEAEs (≥10% of participants in the VER-01 arm) were dizziness, headache, nasopharyngitis, fatigue, nausea, COVID-19, dry mouth and somnolence (Table 3). Rates of headache (VER-01 = 15.9% versus placebo = 12.5%; *P* = 0.160), nasopharyngitis (11.0% versus 14.8%; *P* = 0.107) and COVID-19 (11.0% versus 8.5%; *P* = 0.218) were similar between the groups, whereas dizziness (42.8% versus 5.2%), fatigue (15.1% versus 5.2%), nausea (16.4% versus 3.8%), dry mouth (13.1% versus 3.8%) and somnolence (11.8% versus 0.7%) were more frequent with VER-01 (*P* < 0.001 for all). Most TEAEs for VER-01 were of mild-to-moderate intensity (94%). The incidence of drug-related TEAEs decreased over the course of the treatment period of phase A, converging toward a weekly incidence below 3% (Supplementary Table 8). Discontinuation due to AEs occurred in 17.3% of participants receiving VER-01, compared to 3.5% in the placebo (*P* < 0.001), most commonly due to dizziness, somnolence and nausea. Statistical comparisons of adverse event (AE) incidences were conducted post hoc.

**Table 3:** AEs of phase A

Parameters	VER-01 (*n* = 390)	Placebo (*n* = 425)	*P* value^a^
Any TEAE	325 (83.3%)	286 (67.3%)	<0.001
Any SAE	24 (6.2%)	29 (6.8%)	0.699
Any drug-related SAEs	5 (1.3%)	3 (0.7%)	0.405
AEs reported in ≥10% of participants			
Dizziness	167 (42.8%)	22 (5.2%)	<0.001
Headache	62 (15.9%)	53 (12.5%)	0.160
Nausea	64 (16.4%)	16 (3.8%)	<0.001
Fatigue	59 (15.1%)	22 (5.2%)	<0.001
Dry mouth	51 (13.1%)	16 (3.8%)	<0.001
Somnolence	46 (11.8%)	3 (0.7%)	<0.001
COVID-19	43 (11.0%)	36 (8.5%)	0.218
Nasopharyngitis	43 (11.0%)	63 (14.8%)	0.107

^a^The *P* value for the two-sided chi-squared test, testing the null hypothesis that the frequencies per TEAE category do not depend on treatment groups, was determined post hoc.

No deaths occurred during the study, and no clinically important treatment-related changes were observed for VER-01 in clinical laboratory parameters, vital signs or electrocardiograms (ECGs) when compared to placebo or throughout the study.

At the end of phase A, 68% of participants were satisfied with the tolerability of the treatment in the VER-01 arm compared to 79% in the placebo arm. Satisfaction with tolerability further increased during open-label, long-term treatment, reaching 83% at the end of phase B and 84% at the end of phase C.

No AEs indicative of drug abuse, dependence or withdrawal, as classified under the respective standardized MedDRA queries, were reported (Supplementary Tables 5 and 9). Furthermore, there were no signs of drug abuse based on urine drug tests and the addiction behavior checklist (ABC), and no participant fulfilled the classification criteria for substance dependence according to the 10th revision of the International Classification of Diseases (ICD-10). There were no withdrawal symptoms as measured with the cannabis withdrawal scale (CWS) after abrupt treatment discontinuation in phase D. The daily total intensity score and daily total functional impairment score of the CWS were comparable between VER-01 and placebo) Supplementary Figs. 4 and 5). A pooled analysis of TEAEs for all study phases is provided in Supplementary Tables 5–7.

## Discussion

The results of the VER-CLBP-001 trial demonstrate that VER-01 provides meaningful pain reduction compared to the placebo, accompanied by distinct improvements in physical function and sleep quality, two key factors that contribute to participants perceiving the effects of VER-01 as clinically meaningful. Additionally, participants in the VER-01 arm required substantially lower rescue medication use less rescue medication. These results are of high clinical relevance, as substantiated by a significantly higher proportion of participants randomized to VER-01 achieving well-recognized thresholds of meaningful pain relief, including a ≥30% and ≥50% pain reduction, as well as a ≥2-point decrease on the 11-point NRS^22,23^.

Notably, the NNTB for achieving a ≥30% pain response was 6.8, substantially below the widely accepted clinical relevance threshold of NNTB ≤10 in pain research and below the NNTB = 9 achieved with opioids in CLBP^24–26^. Moreover, significantly more participants in the VER-01 group reported a meaningful improvement in symptoms on the PGIC scale compared to the placebo group, an important indicator of the clinical relevance of treatment effects as recognized by the European Medicines Agency guideline on pain^27^.

Importantly, prolonged treatment with VER-01 was associated with further reductions in pain intensity, as well as continued improvements in physical function, sleep quality and health-related quality of life. Notably, the treatment effect was even more pronounced in participants with a neuropathic pain component and those with severe pain at baseline.

VER-01 was generally well-tolerated, particularly during long-term use. AEs, including those leading to early discontinuation in the VER-01 arm, were predominantly mild to moderate, with an overall intensity comparable to placebo. Most AEs associated with VER-01 were observed during the initial titration phase, were self-limiting and declined markedly within the first few days of treatment, suggesting a rapid onset of sustained tolerability. With 17%, the rate of AE-related discontinuations was higher than with placebo but below those reported for opioids in comparable study designs, with early discontinuations mainly seen in the titration phase^28–30^. Nature and timing of AE-related discontinuations suggest that physicians should advise patients that transient sensations of dizziness, somnolence and nausea may occur in the early titration phase to support treatment adherence. The overall rate of SAEs was comparable to placebo, and there were no signs of dose escalation, abuse, dependence or withdrawal.

These results are particularly relevant given the substantial unmet medical need in CLBP, where many patients remain inadequately treated due to the inherent limitations of current pharmacological options, primarily NSAIDs and opioids. NSAIDs are unsuitable for long-term use due to severe gastrointestinal and cardiovascular risks^11,12^, while opioids are associated with a high risk of addiction, tolerance development, withdrawal symptoms and have a major role in the global opioid crisis^13–15,31^. Against this background, VER-01 presents a promising new nonaddictive, safe and effective treatment option, particularly for long-term use.

Another important advantage of VER-01 over existing pharmacotherapies is its ability to significantly improve sleep quality and physical function. Chronic pain, particularly CLBP, is often associated with severe impairments in these domains, which not only diminish quality of life but also contribute to persistent pain and disease progression. Physical disability in CLBP is perpetuated by a vicious cycle of pain, stiffness and fear of movement, leading to progressive functional decline and reduced mobility^32^. Without effective intervention, this cycle reinforces long-term disability and further limits treatment success. Likewise, sleep disturbances are not merely a secondary symptom of pain but a key contributor to disease progression. Poor sleep exacerbates inflammation, reduces pain tolerance and leads to greater pain severity and prolonged pain duration. As a result, patients with disrupted sleep often experience greater disability, more persistent pain and a higher prevalence of comorbid depression^33,34^. Given these interdependent factors, addressing not only pain but also improving physical function and sleep quality is essential for the effective management of CLBP. Recent meta-analyses have shown that existing pharmacotherapies, including NSAIDs and opioids, have only a small and unlikely clinically relevant effect in these domains, highlighting a major gap in current treatment options^35,36^. By demonstrating clinically meaningful improvements not only in pain but also in physical function and sleep quality, VER-01 represents a promising therapeutic advance that directly targets the multifaceted burden of CLBP.

Notably, there remains a lack of rigorously designed, double-blind, placebo-controlled, multicenter clinical trials evaluating the efficacy and safety of standardized cannabis-based products in well-defined pain conditions, with well-defined dosing regimens and sufficiently long treatment durations. This double-blind, placebo-controlled, multicenter clinical trial addresses these gaps and contributes valuable evidence to the field, underscoring the importance of advancing the clinical understanding and therapeutic potential of full-spectrum cannabis extracts.

Some limitations of this study merit consideration. First, VER-01 was not directly compared with opioids; however, a dedicated follow-up study, evaluating VER-01 against standard opioid therapy, has already been conducted and will be published separately^37^. Second, a formal blinding questionnaire was not administered and prior cannabis experience was not recorded, but multiple indicators support successful blinding—the magnitude of the placebo response closely mirrors that observed in other blinded, randomized, controlled CLBP trials^28,29^, and both treatment arms exhibited a similar, rapid NRS pain decline during the titration period. Furthermore, the nature of AEs was similar between groups, and no instances of unintentional unblinding were reported. Another limitation is that cognitive function was not formally assessed. Although there were no patient reports indicative of impairment, future studies involving cannabinoids might consider incorporating specific cognitive assessments.

Finally, while the difference in time to treatment failure in phase D did not reach statistical significance, the observed trend strongly favors VER-01 with a hazard ratio of 0.75 (*P* = 0.288). The use of time to treatment failure as the primary endpoint to assess maintenance of efficacy represented a novel approach in CLBP, which led to uncertainty in estimating an appropriate sample size. However, the magnitude and direction of the effect, as well as the significantly larger pain increase among patients receiving placebo following withdrawal (MD = 0.5; *P* = 0.034), suggest that the lack of statistical significance in phase D reflects insufficient power rather than waning efficacy.

In conclusion, this phase 3 study provides robust evidence supporting the efficacy and safety of VER-01 in the treatment of CLBP. These findings highlight the importance of further research with VER-01 in other chronic pain conditions and suggest that VER-01 could play an important role in modern pain management.

## Methods

### Study design

This trial investigated the short- and long-term efficacy and safety of VER-01, following the recommendations of the European Medicines Agency and US Food and Drug Administration pain guidelines^27,38^. The trial was divided into four phases (Fig. 2). Phase A had a randomized, double-blind, placebo-controlled design with 1 week of run-in to assess baseline pain intensity, followed by 3 weeks of titration and 12 weeks of treatment. Phase B followed phase A and had a single-arm, open-label design with a 3-week titration period and a 6-month treatment period. All participants entering phase B underwent retitration, as blinding was maintained for phase A throughout the entire study. Participants completing phase B entered either phase C or D. Phase C had a single-arm, open-label design with a 6-month treatment period. Phase D had a double-blind, placebo-controlled, randomized withdrawal design with 4 weeks of treatment followed by 2 weeks of washout. The trial was conducted at 66 outpatient sites and university-based hospitals in Germany and Austria in accordance with the principles of the Declaration of Helsinki, the Good Clinical Practice guidelines of the International Council for Harmonization and applicable regulatory requirements. The trial was approved by ethics committees in each country (Supplementary Table 15), and written informed consent was obtained from all participants. No compensation was offered for participation in the study, except for reimbursement of meals and travel expenses. The trial was prospectively registered with ClinicalTrials.gov (NCT04940741) and EudraCT (2020-000107-36).

### Study participants

Patients were primarily recruited via notices in doctors’ offices, adverts in magazines and online advertising. Additionally, investigators participating in the study recruited patients from their existing databases. This study enrolled individuals who were at least 18 years of age and diagnosed with CLBP (low back pain for at least 3 months), with or without a neuropathic pain component. Individuals were only included if no treatable specific somatic cause for their CLBP was identified (for example, herniated vertebral disk) based on a detailed medical history assessment and a clinical examination. The presence of a neuropathic pain component at baseline was confirmed using the painDETECT questionnaire with a cut-off score >18 (ref. ^39^). Eligible individuals must have had, on average, a pain score of at least 4 points on an 11-point NRS 1 month before enrollment and during the run-in period. Individuals were eligible if drug treatment was indicated and previous optimized treatments with nonopioid analgesics (for example, ibuprofen, diclofenac and metamizol) did not lead to sufficient pain relief or were unsuitable due to contraindications or intolerance. Treatment was considered optimized when further dose escalation was medically inadvisable due to potential adverse effects or when it was unlikely that a higher dose would offer additional therapeutic benefit.

Participants were allowed to continue nondrug therapies for CLBP if these were maintained unchanged for at least 8 weeks before enrollment and if patients were willing to continue their therapy during phase A.

Participants were eligible for inclusion into phase B if they had successfully completed phase A. Participants completing phase B were eligible for inclusion in phase D if they had a clinically meaningful reduction in NRS pain scores (≥30%) at the end of phase B compared to baseline of phase A. Participants not meeting inclusion criteria for phase D were eligible for enrollment in phase C.

Participants were excluded if they had other painful comorbidities potentially interfering with their low back pain rating, a severe mental illness (for example, psychosis, schizophrenia and bipolar disorder), or a history of alcohol, drug or medication abuse. Moreover, participants who had used cannabis-based medicinal products within 30 days before screening were excluded. A full list of inclusion and exclusion criteria is provided in the Supplementary Note—Eligibility criteria.

### Study product

The investigational product VER-01 is a standardized full-spectrum extract derived from the patented *Cannabis sativa* L. strain DKJ127. The botanical raw material of VER-01 consists of the unfragmented, dried *C. sativa* DKJ127 L. flowers. The full-spectrum extract of the botanical raw material is manufactured in a Good Manufacturing Practice (GMP) certified facility and standardized to 5% tetrahydrocannabinol (THC).

Each dose unit (119 µl) of the finished product VER-01 contains 50 µl of the full-spectrum extract, delivering 2.5 mg THC, 0.1 mg cannabigerol and 0.02 mg cannabidiol, with sesame oil as excipient.

Moreover, VER-01 contains a complex, well-characterized mixture of terpenes, flavonoids, carotenes, phytosterols and other bioactive compounds.

The placebo contained sesame oil, cannabis aroma and colorants to mimic the appearance and sensory characteristics of VER-01.

The study products were provided in 30 ml amber glass bottles and taken orally with a dosing syringe, following the European Pain Federation recommendations, which advocate for oral preparations in the management of chronic pain^40^.

### Randomization and masking

The study included two randomizations, conducted at the beginning of phases A and D. Eligible participants were randomly assigned in a 1:1 ratio to VER-01 or placebo. Randomization was stratified according to the presence of a neuropathic pain component. Participants, investigators and study site personnel were masked to treatment assignment. VER-01 and the matching placebo were dispensed in identical amber glass bottles.

Randomization took place in blocks of four, using a computer-generated randomization list. The size of the randomization blocks was not disclosed to the investigators.

### Procedures

At the screening visit, participants were provided with an eDiary and instructed on its use. Most assessments were recorded daily in the eDiary, while others were recorded on paper at the trial site and subsequently transferred to the electronic data capture system by site personnel. At visit A2, eligible participants were randomized. Participants were advised to take the study drug either consistently with or without food. The participants’ individual optimal dose (both VER-01 and placebo) was determined during a 3-week titration period, following a written titration scheme. Provided that the study product was well-tolerated, the dose was increased every 3 days by one dose unit in the morning and one dose unit in the evening until the participant either experienced sufficient symptom relief or reached the maximum daily dose of 13 dose units. The morning and evening doses were adjusted independently.

Intake of analgesics other than rescue medication was prohibited during phase A, the last 3 weeks of phase B and phase D. The use of rescue medication (ibuprofen) was restricted to a maximum of 3 days per week and a daily dose of 2,400 mg. In case ibuprofen was contraindicated, paracetamol was offered at a maximum daily dose of 4,000 mg. Participants were not allowed to initiate a nondrug therapy during phase A, nor during the last 9 weeks preceding inclusion into phase D. If an ongoing nondrug therapy was already in place, it had to be continued unchanged throughout participation in phases A and D.

Participants used the eDiary to record their dosage, pain intensity (11-point NRS), sleep quality (11-point NRS) and use of rescue medication throughout all study phases. Additionally, participants used the eDiary to record potential withdrawal symptoms during phase D, using the CWS.

Phase A included six visits (Fig. 2)—screening (A1), randomization (A2), end of titration (A3) and three visits at 4-week intervals during the treatment phase (A4–A6). Phase B comprised four visits—one at the end of titration (B7) and three visits at 2-month intervals during the treatment phase (B8—B10). Phase C consisted of three visits at 2-month intervals during the treatment phase (C11–C13) and a follow-up visit (C14). Phase D involved two visits—one at the end of treatment (D11) and a follow-up visit (D12).

Safety assessments included AEs, laboratory parameters, vital signs, physical examinations, urine drug screens, pregnancy testing, 24-h ECG assessments (for a subset of 120 participants) and evaluation of substance dependence according to ICD-10 (F12.2) criteria. Investigators assessed the severity and causality of AEs, documenting onset, duration, seriousness and any actions or treatments taken.

A full schedule of assessments is provided in the attached protocol.

### Outcomes

The primary endpoint in phase A was the CFB (week −1) to week 15 in the mean weekly pain intensity in the morning, measured on an 11-point NRS (0 = ‘no pain’ and 10 = ‘worst pain imaginable’). The key secondary endpoint in phase A was the CFB in the NPSI total score at the end of phase A (visit A6) for participants with a neuropathic pain component. The NPSI includes ten items measuring the severity of spontaneous pain (four items), painful attacks (three items), provoked pain (two items) and abnormal sensations (one item)^41^. Each item was scored on an 11-point NRS. The total score ranges from 0 (no symptoms) to 100 (worst imaginable symptoms).

No primary endpoint was defined for the open-label study phases B and C. The primary endpoint of phase D was the time to treatment failure, defined as an increase in the 7-day mean of NRS morning pain intensity by ≥20% and ≥1 point compared to the phase D baseline week 44.

Across all study phases, the proportion of participants achieving ≥30% and ≥50% pain reduction was evaluated, reflecting outcomes widely considered clinically meaningful^22,23,42^.

Additional diary-based secondary efficacy endpoints assessed in all study phases included change in mean pain intensity (11-point NRS), intake of rescue medication, sleep quality on an 11-point NRS (0 = ‘not impacted’; 10 = ‘completely impacted’) and the proportion of participants with ≥30% and ≥50% sleep quality improvement.

Visit-based secondary efficacy endpoints assessed in all study phases included CFB in NPSI total scores, PGIC on a seven-point Likert scale using the question ‘How is your low back pain in comparison to before participation in the study?’ (0 = ‘very much better’ to 6 = ‘very much worse’) and quality of life evaluated with the short-form health survey 36v2 (SF-36)^43,44^. The SF-36 contains 36 questions about physical and mental well-being and was evaluated based on eight domain scores (physical functioning, role-physical, bodily pain, general health, vitality, social functioning, role-emotional and mental health), which were summarized to form two higher-ordered component summary measures (PCS and the mental health component summary). The scores ranged from 0 (maximum disability) to 100 (no disability).

The following visit-based secondary efficacy endpoints were additionally assessed in study phase A: the degree of disability was evaluated with the RMDQ at baseline and end of treatment^45^. The RMDQ is a 24-item questionnaire that assesses the impact of low back pain on functional activities, with scores ranging from 0 to 24, where higher scores indicate greater disability. In addition, sleep quality was measured using the Medical Outcomes Study Sleep Scale (MOS-SS), which consists of 12 items that assess perceived initiation and maintenance of sleep, respiratory problems during sleep, sleep duration, perceived adequacy of sleep and daytime somnolence^46^. The evaluation of the MOS-SS was based on two sleep problem indices. Higher sleep scores indicate a clinically favorable outcome.

Safety was assessed based on the incidence of AEs, including their seriousness, severity and relationship to the study drug. Additionally, patients’ satisfaction with tolerability was evaluated on a visit-by-visit basis using a five-point Likert scale. Substance dependence and abuse potential were measured visit-based with the ABC, a 20-item checklist used to monitor signs of addiction^47^. An ABC sum score of 3 or more indicates inappropriate drug use. Withdrawal symptoms were recorded daily in the eDiary with the CWS during treatment in phase D and during washout in phases C and D. The CWS consists of 19 items representing potential withdrawal symptoms associated with cannabis withdrawal. Each item is rated on a 0–10 scale, where 0 indicates ‘not at all’ and 10 indicates ‘extremely’^48^.

### Sample size estimation

To demonstrate the superiority of VER-01 over placebo for the primary endpoint in phase A, a total of 732 participants had to be randomized in a 1:1 ratio. The sample size calculation was based on an assumed treatment difference of 0.6 NRS points, an s.d. of 2.5 points, a two-sided significance level of 5% and 90% statistical power. For the key secondary endpoint of phase A, 180 participants with a neuropathic pain component had to be randomized in a 1:1 ratio to show superiority of VER-01 versus placebo. This calculation was based on an assumed treatment difference of 10.5 NPSI points, an s.d. of 25.0, a two-sided significance level of 5% and 80% statistical power. Based on a proportion of 22% of participants with a neuropathic pain component, these assumptions resulted in a total sample size of 808 participants for phase A. The enrollment was stopped after 808 participants were randomized. Eligible participants who were already in the run-in phase were still allowed to be randomized.

### Statistical analysis

Demographic data and other phase A baseline characteristics were summarized by treatment group and overall, separately for each study phase, based on the defined analysis sets.

The primary endpoint of phase A was tested by an analysis of covariance model, with treatment as the main effect and with baseline characteristics (presence of a neuropathic pain component; NRS morning pain intensity, age, sex and country) as covariates. The analysis was based on imputed data if intercurrent events prevented the collection of data or if data collected after the occurrence of intercurrent events would have distorted the interpretation of the results. Depending on the type of intercurrent event, data were imputed assuming missing at random (MAR) or missing not at random (MNAR). If data were MAR, these were imputed using a multiple imputation (MI) model based on available data from similar participants in the same treatment group. If data were MNAR (for example, early discontinuation due to AEs or loss of efficacy), data were imputed under the conservative approach of ‘jump to reference’.

This approach was considered conservative because it assumes that participants discontinuing VER-01 would experience outcomes similar to those in the placebo arm, regardless of any prior treatment benefit. Evidence from enriched-enrollment trials^49–51^ suggests that analgesic effects achieved before treatment discontinuation typically persist for several weeks, making a pain rebound above placebo unlikely. Additionally, participants discontinuing active treatment likely continue to benefit from nonspecific trial effects—such as positive expectations, enhanced clinical monitoring and behavioral adjustments—that are equally present in the control group. Finally, the nature of AEs prompting discontinuation was presumed to be mild to moderate and transient, making a lasting pain outcome worse than placebo unlikely.

MI based on the MNAR assumption was performed using the suggested placebo MI algorithm in ref. ^52^. In general, a two-step MI approach was followed. In the first step, an MCMC imputation model was calculated on 100 imputations to transform an arbitrary missing data pattern into a monotone missing pattern. In a second MI step, a monotone regression model was calculated on these 100 imputations. Parameter estimates were combined according to Rubin’s rule. BOCF as well as LOCF imputations were performed as a supportive analysis.

The key secondary endpoint was evaluated similarly to the primary endpoint of phase A using an analysis of covariance model, with treatment as the main effect and baseline characteristics (NPSI total score, age, sex and country) as covariates. The same imputation strategies were applied as for the primary endpoint. Safety was assessed based on AE incidence with post hoc comparisons performed using a two-sided chi-squared test.

To collect efficacy and safety data for at least 300 participants over 6 months, 500 participants were planned to be included in phase B. In addition, 150 participants were planned to be included in phase C to collect long-term efficacy and safety data for at least 100 participants over 12 months.

For the sample size calculation of phase D, a treatment failure rate of 25% was assumed for VER-01 and 55% for placebo, building on ref. ^53^. For the log-rank test comparing the two survival curves with regard to time to treatment failure, a sample size of 78 (39 per treatment group) was required to achieve 80% statistical power under a two-sided significance level of 5%.

The primary endpoint of phase D was analyzed using a Cox proportional hazards model with treatment as the main effect and baseline characteristics (presence of a neuropathic pain component, NRS pain intensity at week 43, age and sex) as covariates. Events indicating treatment failure (such as overdosage of rescue medication or study discontinuation due to intolerability, lack of efficacy or noncompliance) were classified as treatment failure events. Data from participants with intercurrent events not indicating treatment failure were considered missing and imputed under the MAR assumption. Discontinuation of the study for reasons not indicating treatment failure led to censoring in time-to-event analyses.

The analysis sets for phases A and D included all randomized participants who received at least one dose of study medication in the respective phase, with participants assigned to treatment groups as randomized (full analysis sets) or as treated (safety analysis sets). The safety analysis sets for phases B and C included all participants who received at least one dose of study medication in the respective phase. No independent data monitoring committee was used. All statistical analyses were conducted by an independent clinical research organization with SAS software, version 9.4 (SAS Institute).

### Reporting summary

Further information on research design is available in the Nature Portfolio Reporting Summary linked to this article.

## Online content

Any methods, additional references, Nature Portfolio reporting summaries, source data, extended data, supplementary information, acknowledgements, peer review information; details of author contributions and competing interests; and statements of data and code availability are available at 10.1038/s41591-025-03977-0.

## Supplementary information

Supplementary Information Supplementary Note (Eligibility criteria), Tables 1–15, Figs. 1–5 and Study protocol.

Reporting Summary

## Figures and Tables

**Extended Data Table 1:**
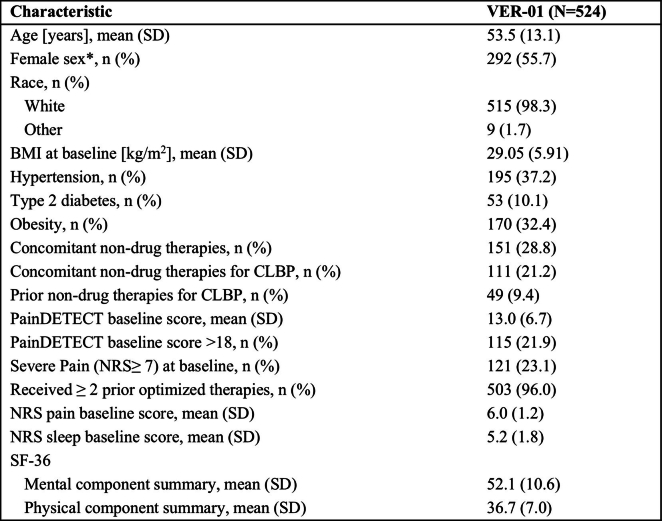
Demographics and other trial baseline characteristics for participants treated in phase B

SD, standard deviation; BMI, body mass index; RMDQ, Roland Morris Disability Questionnaire; MOS-SS, Medical Outcomes Study Sleep Scale; SF-36, short-form 36; NRS, numeric rating scale; CLBP, chronic low back pain; *self-reported; N, total number of patients in the corresponding treatment group.

**Extended Data Table 2:**
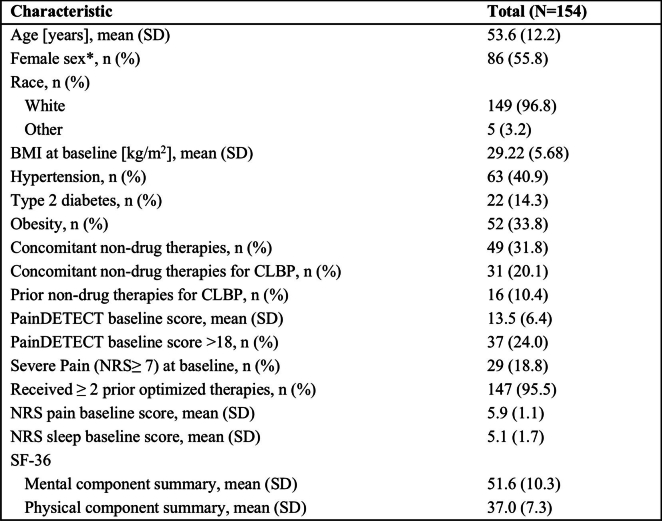
Demographics and other trial baseline characteristics for participants treated in phase C

SD, standard deviation; BMI, body mass index; RMDQ, Roland Morris Disability Questionnaire; MOS-SS, Medical Outcomes Study Sleep Scale; SF-36, short-form 36; NRS, numeric rating scale; CLBP, chronic low back pain; *self-reported; N, total number of patients in the corresponding treatment group.

**Extended Data Table 3:**
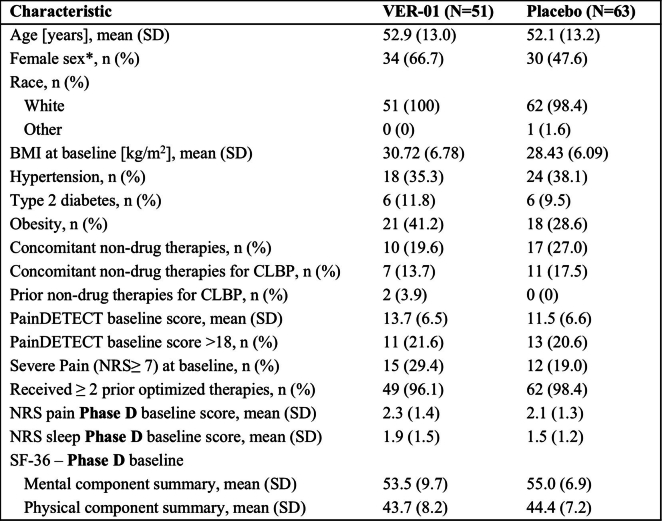
Demographics and other trial baseline characteristics for participants treated in phase D

SD, standard deviation; BMI, body mass index; RMDQ, Roland Morris Disability Questionnaire; MOS-SS, Medical Outcomes Study Sleep Scale; SF-36, short-form 36; NRS, numeric rating scale; CLBP, chronic low back pain; *self-reported; N, total number of patients in the corresponding treatment group.

**Extended Data Table 4:**
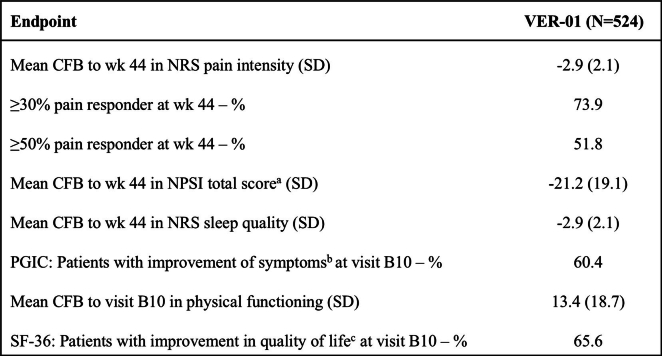
Secondary endpoints of phase B

Wk, week; CFB, change from phase A baseline; SF-36, short-form 36; PGIC, patient global impression of change; NRS, numeric rating scale; NPSI, neuropathic pain symptom inventory; N, total number of patients in the corresponding treatment group.

^a^Patients with a neuropathic pain component.

^b^Improvement of symptoms: very much better and much better.

^c^Improvement in quality of life—somewhat better now than 1 year ago, much better now than 1 year ago.

**Extended Data Table 5:**
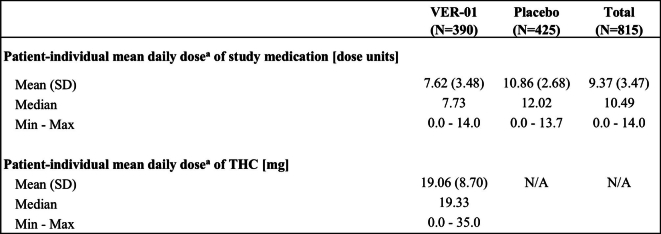
Patient-individual mean daily dose of study medication and THC for phase A treatment phase

N, total number of patients in the corresponding treatment group; SD, standard deviation; Min, minimum; Max, maximum; N/A, not applicable.

^a^The mean is based on all days of the treatment phase on which a morning and evening intake of study medication was foreseen, that is, all but on the first or last day.

**Extended Data Table 6:**
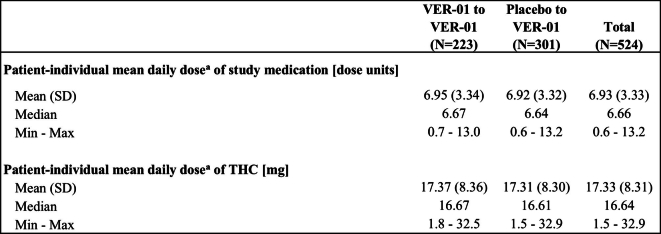
Patient-individual mean daily dose of study medication and THC for phase B treatment phase

N, total number of patients in the corresponding treatment group; SD, standard deviation; Min, minimum; Max, maximum.

^a^The mean is based on all days of the treatment phase on which a morning and evening intake of study medication was foreseen, that is, all but on the first or last day.

**Extended Data Table 7:**
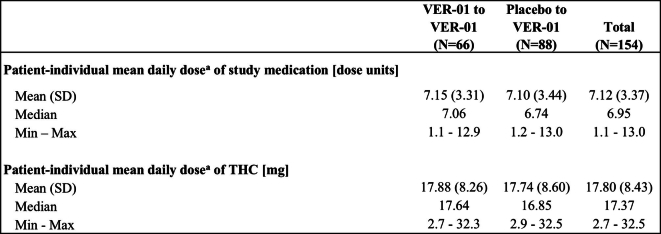
Patient-individual mean daily dose of study medication and THC for phase C

N, total number of patients in the corresponding treatment group; SD, standard deviation; Min, minimum; Max, maximum.

^a^The mean is based on all days of the treatment phase on which a morning and evening intake of study medication was foreseen, that is, all but on the first or last day.

**Extended Data Table 8:**
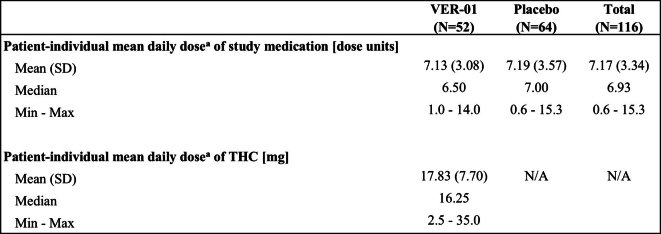
Patient-individual mean daily dose of study medication and THC for phase D

N, total number of patients in the corresponding treatment group; SD, standard deviation; Min, minimum; Max, maximum; N/A, not applicable.

^a^The mean is based on all days of the treatment phase on which a morning and evening intake of study medication was foreseen, that is, all but on the first or last day.

## Data Availability

The study protocol is provided in the Supplementary Information. Access to anonymized individual data and blank case report forms that underlie the results reported in this article can be requested by qualified researchers for academic purposes. Vertanical provides access within 3 months following review and approval of a research proposal, statistical analysis plan and execution of a data access agreement. Data are available to request after the indicated study has been approved in the USA and the European Union for a period of 5 years. Data will be shared through a secure online platform. Submit requests via https://vertanical.com/.

## Code availability

ClinCase by Quadratek Data Solutions, version 2.7, was used to capture and store participants’ clinical data. Analyses were performed with SAS software, version 9.4 (SAS Institute), in keeping with the statistical analysis plan.
