# Supplementary material for: Full-spectrum extract from Cannabis sativa DKJ127 for chronic low back pain: a phase 3 randomized placebo-controlled trial
Source: Nat Med. 2025 Sep 29;31(12):4189–96. doi: 10.1038/s41591-025-03977-0 (PMC12705446; doi:10.1038/s41591-025-03977-0)
Supplement: Supplementary file 1 — Supplementary Note (Eligibility criteria), Tables 1–15, Figs. 1–5 and Study protocol. [file 41591_2025_3977_MOESM1_ESM.pdf]

nature medicine

Supplementary information

https://doi.org/10.1038/s41591-025-03977-0

Full-spectrum extract from *Cannabis sativa* DKJ127 for chronic low back pain: a phase 3 randomized placebo-controlled trial

In the format provided by the authors and unedited

Content

1	Eligibility criteria	2
2	Demographics and baseline characteristics for subgroups	5
3	CLBP Duration at Screening	6
4	Sex-based analysis of primary Endpoint in Phase A	6
5	NRS Pain Scores in Phase C	7
6	Daily dose	7
7	Pooled analysis of TEAEs for subjects treated with VER-01	9
8	Pooled analysis of TEAEs leading to study drug discontinuation for subjects treated with VER-01	10
9	Pooled analysis of TEAEs for subjects treated with VER-01 by maximum severity	10
10	Percentage of patients with a least one related TEAE by analysis week	11
11	Types of Serious TEAEs	12
12	Cannabis Withdrawal Scale (CWS)	13
12.1	Daily total intensity score of CWS	13
12.2	Daily total functional impairment score of CWS	13
13	Sensitivity Analysis for the Primary Estimand of Phase A	14
14	Study schedule	15
15	List of involved independent Ethics Committees	19

1 Eligibility criteria

General Inclusion Criteria

1. Male and female patients (18 years and older)
2. Chronic (for at least three months) non-specific pain in the lower back (between the lower ribcage and the gluteal folds)
3. Pain intensity on average at least 4 points on an 11-point NRS (one month before the start of the study)
4. Patients with indicated drug treatment where previous optimised treatments with non-opioid analgesics have not led to sufficient pain relief or were unsuitable due to contraindications or intolerance
5. Willingness of both men and women to use a reliable method of contraception during study participation and for three months after taking the last dose of the IMP
6. Signed patient information and informed consent form is available
7. Understanding of the German language, ability to give consent and compliance
8. The patient has understood the instructions to avoid changes in lifestyle and dietary habits
9. The patient has understood the principle of the patient diary and gives their consent to keep it as instructed

Additional Inclusion Criteria for Phase A

1. Pain intensity averaged at least 4 points on an 11-point NRS (there must be at least 5 pain intensity readings in the morning from the run-in phase)
2. Willingness not to take any analgesic medication (non-opioid and opioid analgesics as well as adjuvant analgesics) during participation in study Phase A (except rescue medication)
3. Willingness to continue a current non-drug therapy unchanged as planned during participation in Phase A

Additional Inclusion Criteria for Phase B

1. Previous and complete participation in Phase A until and including Visit A6
2. Patient wishes to participate voluntarily in the long-term study
3. From the investigator's point of view, further participation is considered medically safe
4. Willingness not to take any additional analgesic medication (non-opioid and opioid analgesics as well as adjuvant analgesics) during the last three weeks of study Phase B (except rescue medication).

Additional Inclusion Criteria for Phase C

1. Previous and complete participation in Phase B until and including Visit B10
2. Patient wishes to participate voluntarily in the long-term study
3. From the investigator's point of view, further participation is considered medically safe

Additional Inclusion Criteria for Phase D

1. Previous and complete participation in Phase B until and including Visit B10 (patients received VER-01 for 26 weeks)
2. Patient has experienced a pain score improvement of at least 30% in treatment Phase B (mean value of the study week 43 compared to the mean value of the run-in phase, there must be at least four values from study week 43 and five values from the run-in phase)
3. Patient wishes to participate voluntarily in the study
4. From the investigator's point of view, further participation is considered medically safe
5. Willingness not to take any analgesic medication (non-opioid and opioid analgesics as well as adjuvant analgesics) during participation in study Phase D (except rescue medication)
6. Willingness to continue a current non-drug therapy unchanged as planned during study

General Exclusion Criteria

1. Professional groups for which the ability to operate machinery and drive vehicles is the primary activity (including truck, bus and forklift drivers, pilots)
2. Alcohol/drug/medication abuse and previous or current intake of methadone in the patient's medical history or suspected by the investigator
3. Intake of analgesic medication (non-opioid and opioid analgesics as well as adjuvant analgesics) within seven days prior to the start of the study
4. Taking cannabis-based products within 30 days prior to the start of the study
5. HIV, dementia (which impairs the assessment of symptoms)
6. Severe forms of the following diseases: Anaemia, hematological/autoimmune/endocrinal/ renal/hepatic/respiratory/cardiovascular or gastrointestinal diseases, symptomatic peripheral vascular diseases
7. Cardiovascular event in the past three months, poorly managed high blood pressure, untreated hypothyroidism, patients with Crigler-Najjar syndrome or Rotor syndrome, surgery within the past two months

8. Severe mental illnesses (e.g. psychosis, schizophrenia, bipolar disorder), severe depression that is not due to the chronic non-specific low back pain, or individuals at risk of suicide (examined using the MINI questionnaire)
9. Severe mental illness (psychosis, schizophrenia, bipolar disorder, severe depression, anxiety disorder) in a first-degree relative (parents and children); suicide in a first-degree relative (parents and children)
10. Patients with an active cancer or tumor-related pain or severe pain due to physical injury
11. Other painful comorbidities, excluding low back pain, that could interfere with the patient's evaluation during the study or the assessment of pain
12. Well-known strong adverse events in connection with cannabis consumption before the start of the study
13. Known allergy to cannabis and/or sesame seeds and products derived from them
14. Known hypersensitivity to the ingredients of the rescue medication
15. Planned blood donation, planned sperm or egg donation, planned freezing of eggs or sperm
16. Pregnancy, breastfeeding, desire to have children (within the next 20 months)
17. Participation in another clinical trial within the past 30 days before the start of the study
18. Inability to give consent, care dependency, patient has a legal guardian/caregiver, or is immobile
19. The patient is in need of special protection (e.g., incarcerated; institutionalized by a court or judicial authority; in a dependent or employment relationship with the sponsor, an external service provider of the sponsor (who is involved in the study conduct), the investigator, or the study site)

Additional Exclusion Criteria for Phase A:

1. In the case of a current non-drug therapy (e.g. physical or behavioural therapy, acupuncture, massage, thermotherapy), which significantly modulates the perception of pain, it was not maintained unchanged for at least eight weeks prior to study participation in Phase A

Additional Exclusion Criteria for Phase D

1. Intake of additional analgesic medication (non-opioid and opioid analgesics as well as adjuvant analgesics) within 21 days prior to the start of study Phase D (except rescue medication)
2. In the case of a current non-drug therapy (e.g. physical or behavioural therapy, acupuncture, massage, thermotherapy) that significantly modulates the perception of pain, it was not maintained unchanged for at least nine weeks prior to the start of study Phase D

2 Demographics and baseline characteristics for subgroups

**Table S1. Demographics and other baseline characteristics – neuropathic pain cohort (PainDETECT>18)**		
**Characteristic**	**VER-01 (N=88)**	**Placebo (N=91)**
Age [years], mean (SD)	52.4 (14.2)	52.0 (13.5)
Female sex*, n (%)	47 (53.4)	58 (63.7)
BMI at baseline [kg/m^2], mean (SD)	29.2 (6.4)	30.0 (6.6)
PainDETECT baseline score, mean (SD)	21.8 (2.7)	22.5 (3.2)
PainDETECT baseline score >18, n (%)	88 (100.0)	91 (100.0)
Severe Pain (NRS≥ 7) at baseline, n (%)	39 (44.0%)	31 (34.1%)
NRS pain score, mean (SD)	6.7 (1.2)	6.5 (1.4)
NPSI total score, mean (SD)	47.1 (17.2)	48.7 (16.4)
NRS sleep score, mean (SD)	6.2 (1.6)	6.0 (1.8)

**Table S2. Demographics and other baseline characteristics – severe pain cohort (NRS pain ≥ 7)**		
**Characteristic**	**VER-01 (N=101)**	**Placebo (N=93)**
Age [years], mean (SD)	51.1 (13.9)	51.9 (14.9)
Female sex*, n (%)	65 (64.4)	60 (64.5)
BMI at baseline [kg/m^2], mean (SD)	29.65 (6.83)	30.60 (7.36)
PainDETECT baseline score, mean (SD)	9.4 (5.2)	11.0 (4.6)
PainDETECT baseline score >18, n (%)	39 (38.6)	31 (33.3)
Severe Pain (NRS≥ 7) at baseline, n (%)	101 (100.0%)	93 (100.0%)
NRS pain score, mean (SD)	7.6 (0.6)	7.8 (0.6)
NRS sleep score, mean (SD)	6.8 (1.6)	6.8 (1.7)

Abbreviations: SD = Standard Deviation; BMI = Body Mass Index; NRS = Numeric Rating Scale; *self-reported; N = total number of patients in the corresponding treatment group

3 CLBP Duration at Screening

Table S3. Duration since start of CLBP symptoms at Screening

	VER-01 (N=390)		Placebo (N=425)		Total (N=815)	
	**Pat.n**	**Pat.%**	**Pat.n**	**Pat.%**	**Pat.n**	**Pat.%**
**≤ 3 months**	0	0.0	1	0.2	1	0.1
> 3 months until < 1 years	15	3.8	11	2.6	26	3.2
≥ 1 years until < 2 years	18	4.6	21	4.9	39	4.8
≥ 2 years until < 5 years	72	18.5	99	23.3	171	21.0
≥ 5 years until < 10 years	83	21.3	91	21.4	174	21.3
≥ 10 years	200	51.3	197	46.4	397	48.7
Missing	2	0.5	5	1.2	7	0.9

N = total number of patients in the corresponding treatment group;
Pat.n = number of patients in corresponding category; Pat.% = number of patients in the corresponding category / total number of patients with at least one dose of VER-01;

4 Sex-based analysis of primary Endpoint in Phase A

Table S4. Primary estimand for Phase A: ANCOVA including treatment and sex interaction for the mean change from baseline in NRS pain intensity in the morning at Week 15 based on imputed data

Effect	Minimum of p-value	Maximum of p-value
Treatment and sex interaction	0.208	0.996

The minimum and maximum p-value of the treatment*sex interaction term for the 100 imputations are displayed.

5 NRS Pain Scores in Phase C

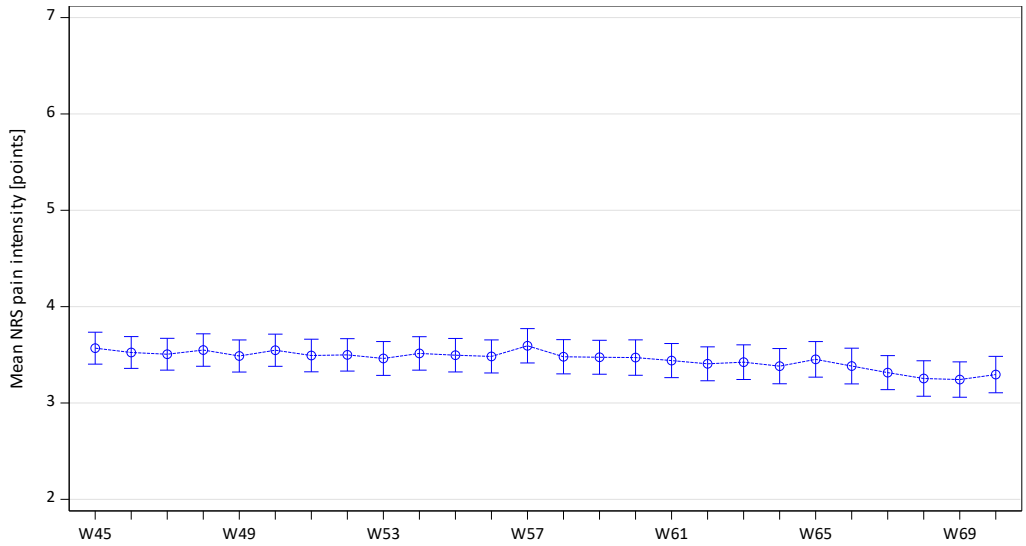

Figure S1. Mean NRS pain intensity in phase C (week 45 (W45) to week 70)

Bars display the range from mean +/- standard error of the mean

6 Daily dose

Phase C

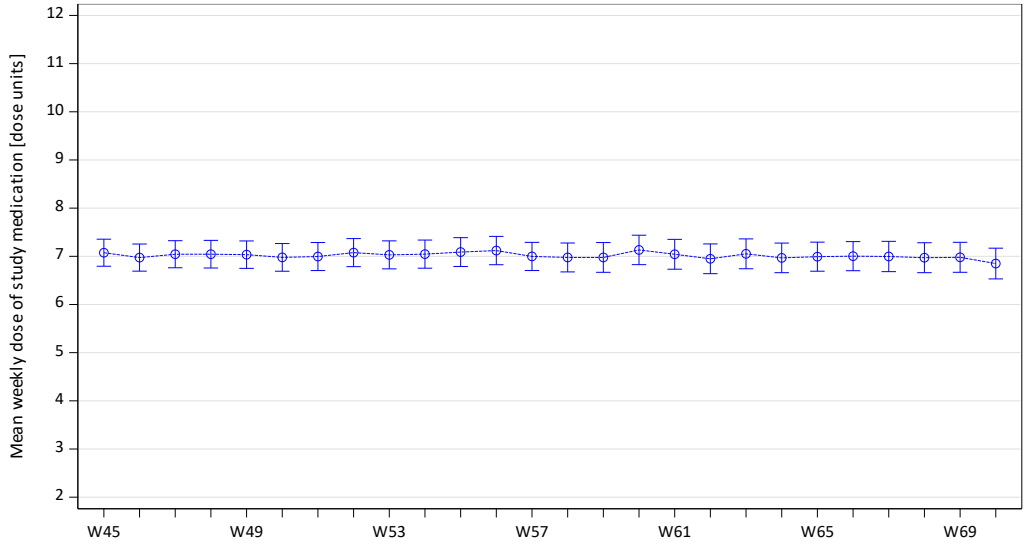

Figure S2. Weekly mean of daily dose of VER-01 in phase C (week 45 to 70)

Bars display the range from mean +/- standard error of the mean.

Phase D

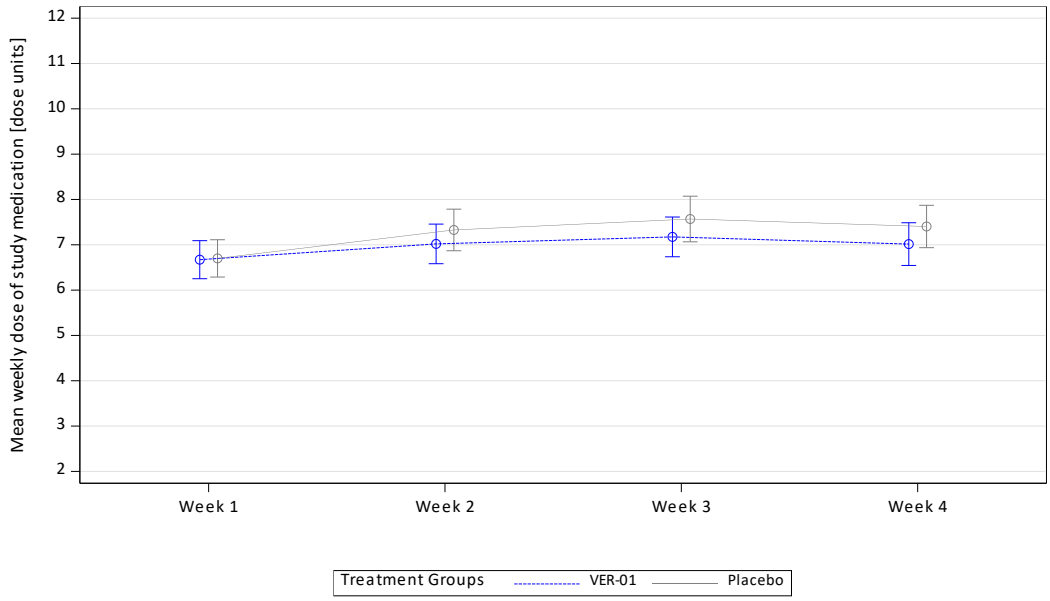

Figure S3. Weekly mean of daily dose of VER-01 and Placebo in phase D

Bars display the range from mean +/- standard error of the mean. Blue line = VER-01, gray line = placebo.

7 Pooled analysis of TEAEs for subjects treated with VER-01

Table S5. Pooled adverse events of phase A, B, C and D

	**VER-01 (N=691)**
Any TEAE	595 (86.1%)
Any SAE	84 (12.2%)
Any drug-related SAEs	16 (2.3%)
Adverse events reported in ≥10% of patients	
Dizziness	282 (40.8%)
Nasopharyngitis	134 (19.4%)
COVID-19	128 (18.5%)
Headache	113 (16.4%)
Nausea	108 (15.6%)
Fatigue	87 (12.6%)
Dry mouth	87 (12.6%)
Diarrhoea	75 (10.9%)
Somnolence	74 (10.7%)

Abbreviations: TEAE = Treatment Emergent Adverse Events; SAE = Serious Adverse Event; N = total number of patients in the corresponding treatment group

8 Pooled analysis of TEAEs leading to study drug discontinuation for subjects treated with VER-01

Table S6. Pooled analysis of TEAEs leading to study drug discontinuation for subjects treated with VER-01

	**Pat. n (%)**<br>**(N=691;**<br>**Patient years under exposure = 407)**	**Events (Event rate)**
Dizziness	35 (5.1%)	35 (0.0860)
Somnolence	11 (1.6%)	11 (0.0270)
Nausea	11 (1.6%)	11 (0.0270)

Abbreviations: TEAE = Treatment Emergent Adverse Events; SAE = Serious Adverse Event; N = total number of patients in the corresponding treatment group. Only TEAEs affecting >1% of patients are shown

9 Pooled analysis of TEAEs for subjects treated with VER-01 by maximum severity

Table S7. Pooled analysis of TEAEs for subjects treated with VER-01 by maximum severity

	**Pat. n (%)**<br>**N=691**
Any	595 (86.1%)
Mild	153 (22.1%)
Moderate	344 (49.8%)
Severe	98 (14.2%)

Abbreviations: TEAE = Treatment Emergent Adverse Events; SAE = Serious Adverse Event; N = total number of patients in the corresponding treatment group. Only TEAEs affecting >1% of patients are shown

10 Percentage of patients with a least one related TEAE by analysis week

Table S8. Percentage of patients with at least one related TEAE in respective analysis week by analysis week for Phase A – All patients treated with VER-01 in Phase A

		VER-01 (N=390)	
**Analysis week**	**n**	**Pat.n**	**Pat.%**
Week 1	390	74	19.0
Week 2	384	124	32.3
Week 3	378	91	24.1
Week 4	365	54	14.8
Week 5	346	39	11.3
Week 6	337	29	8.6
Week 7	331	21	6.3
Week 8	321	24	7.5
Week 9	304	15	4.9
Week 10	298	10	3.4
Week 11	289	8	2.8
Week 12	285	9	3.2
Week 13	278	8	2.9
Week 14	276	6	2.2
Week 15	275	3	1.1

N = total number of patients in the corresponding treatment group

11 Types of Serious TEAEs

Table S9. Serious TEAE categorized by MedDRA System Organ Class and Preferred Term, pooled across Phases A, B, C, and D, and reported in more than one participant

	VER-01 (N=691; Patient years under exposure = 406.99)			
**MedDRA System Organ Class**<br>**MedDRA Preferred Term**	**Pat.n**	**Pat.%**	**Events**	**Adverse event rate**
**Any**	16	2.3	25	0.0614
**Nervous system disorders**				
**Any**	8	1.2	10	0.0246
Syncope	4	0.6	5	0.0123
Headache	2	0.3	2	0.0049
**Psychiatric disorders**				
**Any**	5	0.7	7	0.0172
Hallucination	2	0.3	2	0.0049
**Investigations**				
Any	2	0.3	2	0.0049

TEAE = treatment emergent adverse event; SOC = System Organ Class; PT = Preferred Term; MedDRA = Medical Dictionary for Regulatory Activities;
N = total number of patients who took at least one dose of VER-01;
Pat.n = number of patients with an adverse event in corresponding MedDRA System Organ Class, Preferred Term;
Pat.% = number of patients with at least one adverse event of the specified adverse event type/ N;
An adverse event related to study drug is defined as one considered by the investigator to have at least a 'possible' relationship to study drug or the relationship to study drug was 'unassessable', 'not assessed', or missing.
Only serious related TEAEs which occurred for patients with at least one dose of VER-01 and with a start date during VER-01 treatment are considered for this table.
Adverse event rates are calculated as number of events / patient years under exposure.
Adverse events coded according to MedDRA version 26.0.

12 Cannabis Withdrawal Scale (CWS)

12.1 Daily total intensity score of CWS

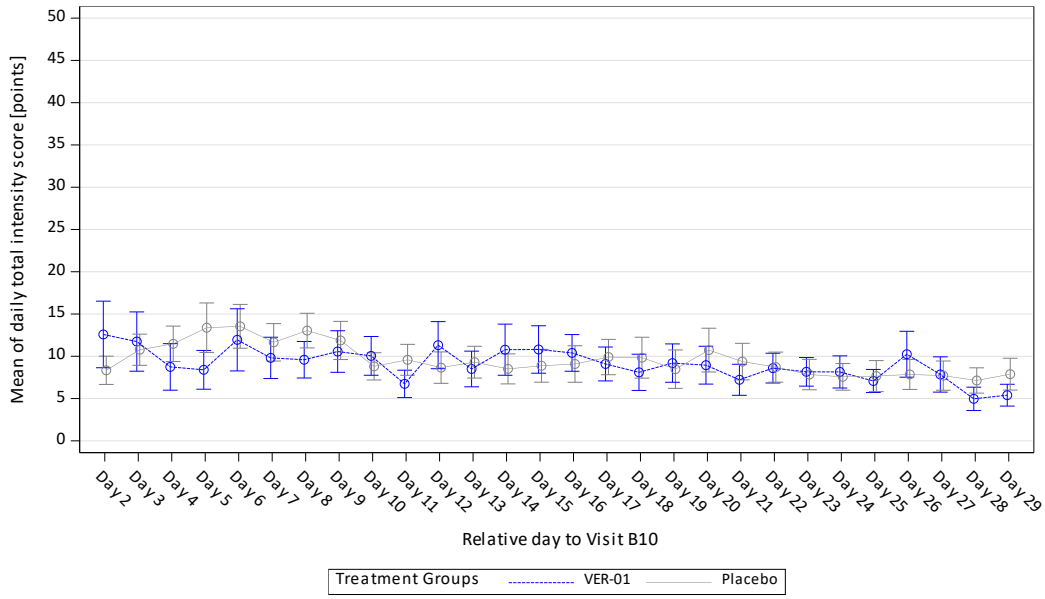

Figure S4. Daily total intensity score of CWS in the treatment phase of phase D.

Bars display the range from mean +/- standard error of the mean

12.2 Daily total functional impairment score of CWS

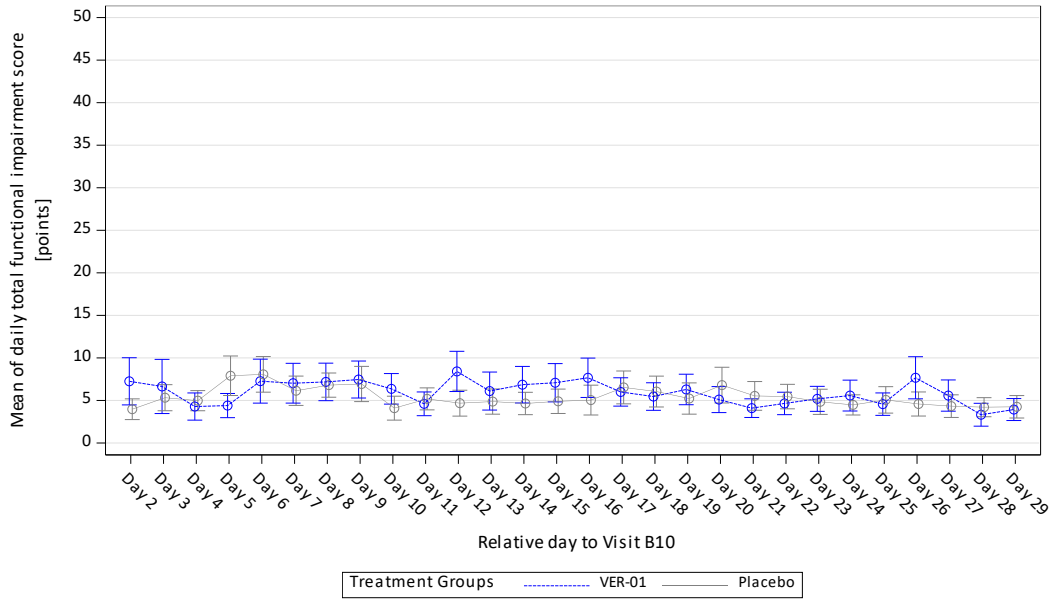

Figure S5. Daily total functional impairment score of CWS in the treatment phase of phase D.

Bars display the range from mean +/- standard error of the mean

13 Sensitivity Analysis for the Primary Estimand of Phase A

Table S10. Sensitivity Analysis for the Primary Estimand of Phase A: BOCF Imputation					
**BOCF Imputation**	**N**	**LS mean [points][a]**	**SE of LS mean [points]**	**95% CI for LS mean**	**p-value[b]**
VER-01	390	- 1.6	0.1	-1.9 to -1.3	-
Placebo	425	-1.2	0.1	-1.5 to -0.9	-
Difference VER-01-Placebo	-	-0.4	0.1	-0.7 to -0.1	0.005

ANCOVA = analysis of covariance; N = total number of patients per treatment group; LS mean = least square mean estimated from ANCOVA model; 95% CI = two-sided 95% confidence interval; SE = standard error; NRS = numeric rating scale;

[a]LS means and corresponding 95% confidence intervals based on the ANCOVA model with the change from baseline in mean NRS pain intensity in the morning at Week 15 with baseline observation carried forward (BOCF) imputation for imputation under the MNAR assumption as response variable and treatment group as main effect as well as baseline characteristics (neuropathic pain component, baseline NRS pain intensity, age, gender and country) as covariates; presented results are combined results for multiply imputed data using Rubin's rule;

[b]p-value = ANCOVA-based p-value for the two-sided tested null hypothesis that the mean change from baseline in NRS pain intensity in the morning at Week 15 is equal in both treatment groups;

Table S11. Sensitivity Analysis for the Primary Estimand of Phase A: LOCF Imputation					
**LOCF Imputation**	**N**	**LS mean [points][a]**	**SE of LS mean [points]**	**95% CI for LS mean**	**p-value[b]**
VER-01	390	-1.8	0.1	-2.0 to -1.5	-
Placebo	425	-1.3	0.1	-1.6 to -1.0	-
Difference VER-01-Placebo	-	-0.5	0.1	-0.7 to -0.2	<0.001

ANCOVA = analysis of covariance; N = total number of patients per treatment group; LS mean = least square mean estimated from ANCOVA model; 95% CI = two-sided 95% confidence interval; SE = standard error; NRS = numeric rating scale;

[a]LS means and corresponding 95% confidence intervals based on the ANCOVA model with the change from baseline in mean NRS pain intensity in the morning at Week 15 with missing data imputation for early discontinuation from study due to any reason using last observation carried forward (LOCF) as response variable and treatment group as main effect as well as baseline characteristics (neuropathic pain component, baseline NRS pain intensity, age, gender and country) as covariates; presented results are combined results for multiply imputed data using Rubin's rule;

[b]p-value = ANCOVA-based p-value for the two-sided tested null hypothesis that the mean change from baseline in NRS pain intensity in the morning at Week 15 is equal in both treatment groups;

14 Study schedule

Table S12. Tests during the clinical trial

	Phase A						Phase B				Phase C				Phase D	
**Study day [weekdays] (+/± possible tolerance [weekdays])**	**-8**	**1 (+3)**	**22 (+3)**	**50 (±3)**	**78 (±3)**	**106 (+3)**	**127 (+3)**	**183 (±3)**	**239 (±3)**	**309 (+3)**	**365 (±3)**	**421 (±3)**	**491 (±3)**	**505 (±3)**	**337 (+3)**	**351 (±3)**
**Study week**	**-2**	**1**	**4**	**8**	**12**	**16**	**19**	**27**	**35**	**45**	**53**	**61**	**71**	**73**	**49**	**51**
**Visit**	**A1**	**A2**	**A3**	**A4**	**A5**	**A6**	**B7**	**B8**	**B9**	**B10**	**C11**	**C12**	**C13**	**C14**	**D11**	**D12**
Information and inclusion of the patient																
Information and consent to study participation	x									x						
Determination of medical history and concurrent diseases	x															
Fulfilment of inclusion and exclusion criteria	x	x				x				x						
Documentation of demographic data	x															
Issuance of the notes on concomitant therapy	x															
Study-specific examinations																
Physical examination	x		x			x				x			x	x	x	x
Drug test (urine test): THC, cocaine, morphine, methamphetamine, amphetamine, methadone, benzodiazepine, ecstasy	x						x			x				x		
Drug test (urine test): cocaine, morphine, methamphetamine, amphetamine, methadone, benzodiazepine, ecstasy																x
	**A1**	**A2**	**A3**	**A4**	**A5**	**A6**	**B7**	**B8**	**B9**	**B10**	**C11**	**C12**	**C13**	**C14**	**D11**	**D12**
Blood draw (to determine a complete blood count and clinical	x					x	x			x			x			

chemistry parameters)																
Pregnancy test		x	x	x	x	x	x	x	x	x	x	x	x		x	
24-hour electrocardiogram (on 120 patients): in addition to A2 + A5, Day 1 of titration Phase A after taking the 1st dose		x			x											
Measurement of vital signs	x	x	x	x	x	x	x	x	x	x	x	x	x	x	x	x
Questionnaires and data collection																
Assessment of quality of life by the patient (SF-36)		x	x	x	x	x				x			x		x	
Assessment of sleep quality by the patient (MOS-SS)		x	x	x	x	x										
Assessment of disability and symptoms by the patient (RMD)	x	x				x										
Detection of neuropathic pain (painDETECT)	x															
Measurement of neuropathic pain (NPSI)	x	x	x	x	x	x	x	x	x	x	x	x	x	x	x	x
Assessment of the treatment results by the investigator						x				x			x		x	
Global assessment of symptoms by the patient						x				x			x		x	
Assessment of the treatment results and of tolerability by the patient						x				x			x		x	
Assessment of substance dependence by the investigator (ABC)			x	x	x	x	x	x	x	x	x	x	x		x	
	**A1**	**A2**	**A3**	**A4**	**A5**	**A6**	**B7**	**B8**	**B9**	**B10**	**C11**	**C12**	**C13**	**C14**	**D11**	**D12**
Assessment of substance dependence by the investigator, with potential diagnosis													x		x	
Determination of psychological disorders (MINI)	x															
Study product and rescue medication																
Rescue medication and pantoprazole given out (more can be given out if required (x))	x	(x)	(x)	(x)	(x)	(x)	(x)	(x)	(x)	(x)	(x)	(x)	(x)		(x)	

Documentation of the intake of rescue medication and pantoprazole, if applicable		x	x	x	x	x	x	x	x	x	x	x	x	x	x	x
Dispensation of the IMP (additionally for Visits AID1-AID8)		x	x	x	x	x	x	x	x	x	x	x				
Return of the IMP (additionally for Visits AID1-AID8) and check of compliance (IMP taken correctly)			x	x	x	x	x	x	x	x	x	x	x		x	
Documentation of rescue medication and concomitant therapies		x	x	x	x	x	x	x	x	x	x	x	x	x	x	x
eDiary																
Issuance of access data for e-Diary and instruction	x															
Checking the e-Diary		x	x	x	x	x	x	x	x	x	x	x	x	x	x	x
Patient safety																
Possible early discontinuation from study (additionally for Visits AID1-AID8)	x	x	x	x	x	x	x	x	x	x	x	x	x	x	x	x
Documentation of AEs/SAEs		x	x	x	x	x	x	x	x	x	x	x	x	x	x	x

*Patients' entries in the eDiary must also be checked between the visits, in particular in relation to SAEs

Table S13. Visits for additional dispensing of the IMP

**Study phase**	**Phase B**				**Phase C**			
**Study day [weekdays]**<br>**(+/± possible tolerance [weekdays])**	**155**<br>**(±3)**	**211**<br>**(±3)**	**267**<br>**(±3)**	**295**<br>**(±3)**	**337**<br>**(±3)**	**393**<br>**(±3)**	**449**<br>**(±3)**	**477**<br>**(±3)**
**Study week**	**23**	**31**	**39**	**43**	**49**	**57**	**65**	**69**
**Visit**	**AID 1**	**AID 2**	**AID 3**	**AID 4**	**AID 5**	**AID 6**	**AID 7**	**AID 8**
Return of the IMP and check of compliance (IMP taken correctly)	x	x	x	x	x	x	x	x

If applicable, return and documentation of rescue medication taken and any dispensing of rescue medication and pantoprazole (if necessary (x))	(x)	(x)	(x)	(x)	(x)	(x)	(x)	(x)
Dispensation of the IMP	x	x	x	x	x	x	x	x
Pregnancy test	x	x	x		x	x	x	
Documentation of AEs/SAEs	x	x	x	x	x	x	x	x
Possible early discontinuation from study	x	x	x	x	x	x	x	x

Table S14. Follow-up Visit

**Study day [weekdays]**<br>**(+/± possible tolerance [weekdays])**	**+ 14 (±3) days after study drop-out*/after Visit A6 or B10, in patients who do not participate in a subsequent study phase**
Physical examination	x
Measurement of vital signs	x
Drug test (urine tests): Cocaine, morphine, methamphetamine, amphetamine, methadone, benzodiazepine and ecstasy	x
Return of any investigational medicinal products and check of compliance (until study drop-out)	x
Return of any rescue medication taken and documentation of rescue medication taken (until study drop-out)	x
If applicable, assessment of the treatment results and of tolerance by the patient	x
Global assessment of symptoms by the patient	x
Assessment of the treatment results by the investigator	x
Documentation of AEs/SAEs	x
Documentation of concurrent medications	x

*A follow-up visit should only be held in the event of study dropout after the investigational medicinal product has been dispensed (in the event of a screen failure, no follow-up visit takes place)

15 List of involved independent Ethics Committees

Table S15. Independent Ethics committees involved in the study

Ethics Committee	Country
Ethik-Kommission der Landesärztekammer Baden-Württemberg, Liebknechtstr. 33, 70565 Stuttgart	Germany
Ethik-Kommission der Bayerischen Landesärztekammer, Mühlbaurstraße 16, 81677 München	Germany
Ethik-Kommission der Landesärztekammer Hessen, Hanauer Landstraße 152, 60314 Frankfurt am Main	Germany
*Ethik-Kommission der Ärztekammer Nordrhein, Tersteegenstraße 9, 40474 Düsseldorf	Germany
Ethik-Kommission der Landesärztekammer Rheinland-Pfalz, Deutschhausplatz 3, 55116 Mainz	Germany
Ethikkommissionen bei der Ärztekammer Schleswig-Holstein, Bismarckallee 8-12, 23795 Bad Segeberg	Germany
Ethik-Kommission an der Medizinischen Fakultät der Eberhard-Karls-Universität und am Universitätsklinikum Tübingen, Gartenstraße 47, 72074 Tübingen	Germany
Landesamt für Gesundheit und Soziales (LAGeSO), Geschäftsstelle der Ethik-Kommission des Landes Berlin, Turmstraße 21, 10559 Berlin	Germany
Ethikkommission der Landesärztekammer Brandenburg, Dreiferstraße 12, 03044 Cottbus	Germany
Ethikkommission der Ärztekammer Hamburg, Weidestraße 122b, 22083 Hamburg	Germany
Ethik-Kommission bei der Ärztekammer Niedersachsen, Unterkommission zur Beurteilung medizinischer Forschung am Menschen, Karl-Wiechert-Allee 18-22, 30625 Hannover	Germany
Ärztekammer Westfalen-Lippe und der Westfälischen Wilhelms-Universität Münster, Gartenstraße 210-214, 48147 Münster	Germany
Ethik-Kommission an der Medizinischen Fakultät der RWTH Aachen, MTI 2, Wendlingweg 2, 52074 Aachen	Germany
Ethik-Kommission bei der Ärztekammer des Saarlandes, Faktoreistraße 4, 66111 Saarbrücken	Germany
Sächsische Landesärztekammer – Ethikkommission, Schützenhöhe 16, 01099 Dresden	Germany
Ethik-Kommission des Landes Sachsen-Anhalt, c/o Landesamt, Kühnauer Straße 70, 06846 Dessau-Roßlau	Germany
Ethik-Kommission der Medizinischen Fakultät der Friedrich-Schiller-Universität Jena, Brachstraße 18, 07740 Jena	Germany

*Ethik-Kommission der Medizinischen Universität Wien, Borschkegasse 8b/E06, 1090 Wien	Austria
Ethik-Kommission des Landes Kärnten, Feschigstraße 11, 9020 Klagenfurt am Wörthersee	Austria
Ethik-Kommission der Stadt Wien, Thomas-Klestil-Platz 8/1, Eingang: Schnirchgasse 12/2/1, 1010 Wien	Austria
Johannes Kepler Universität Linz, Ethikkommission der MED, Med Campus I, Gebäude ADM, 7. OG, Krankenhausstraße 5, 4020 Linz	Austria
Ethik-Kommission der Medizinischen Universität Graz, LKH-Universitätsklinikum – Eingangsgebäude, Auenbruggerplatz 2, 3. OG, 8036 Graz	Austria

*Leading ethics committees

Protocol

Proof of efficacy, maintenance of efficacy, long-term safety and investigation of the potential for dependence and abuse and the effect of abrupt drug withdrawal of VER-01 in a multicentre study in the treatment of patients with chronic non-specific low back pain.

Sponsor:

Vertanical GmbH

Am Haag 14

82166 Gräfelfing

Germany

Study number/protocol code: VER-CLBP-001

Protocol Version 07-CA of September 22, 2023

EudraCT number: 2020-000107-36

The information in this protocol is strictly confidential. Its sole purpose is to provide information to the sponsor, the investigator, the study staff, the institutional review board/independent ethics committee, the authorities, and patients. This protocol must not be passed on to third parties without the consent of the sponsor.

I. Signatures

I declare my agreement with the protocol and its annexes (date, signature):

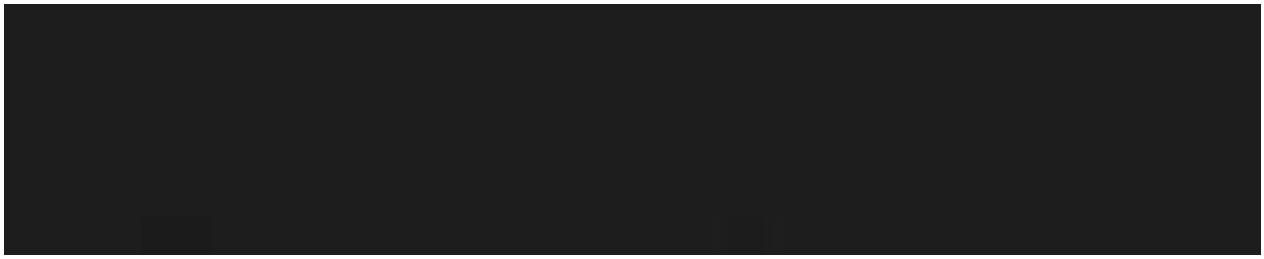

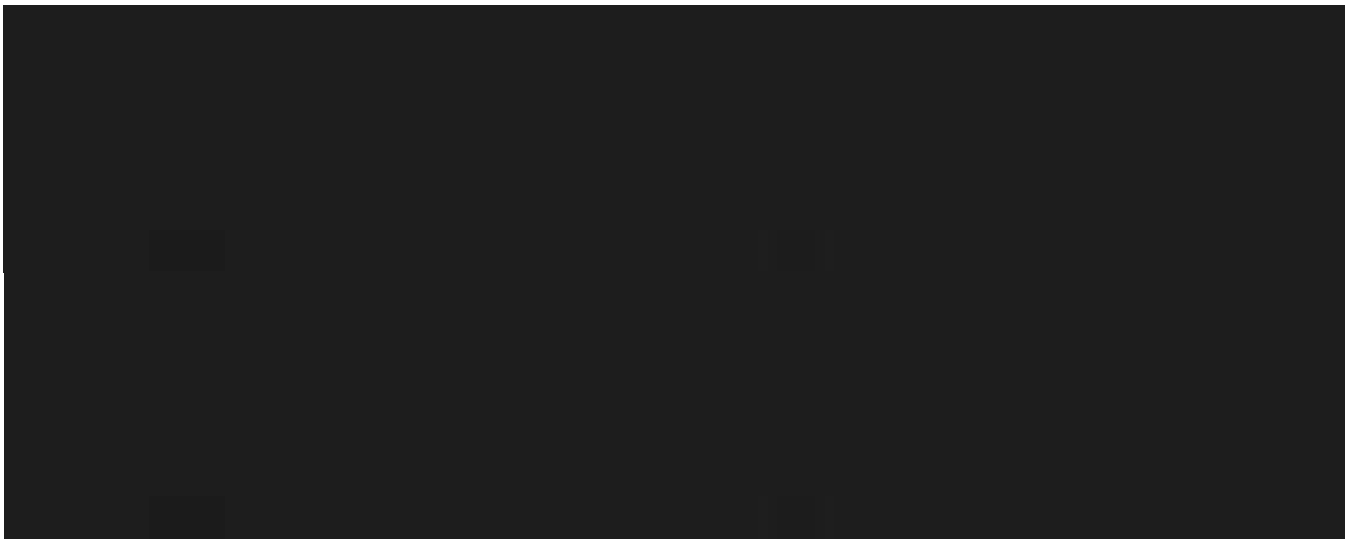

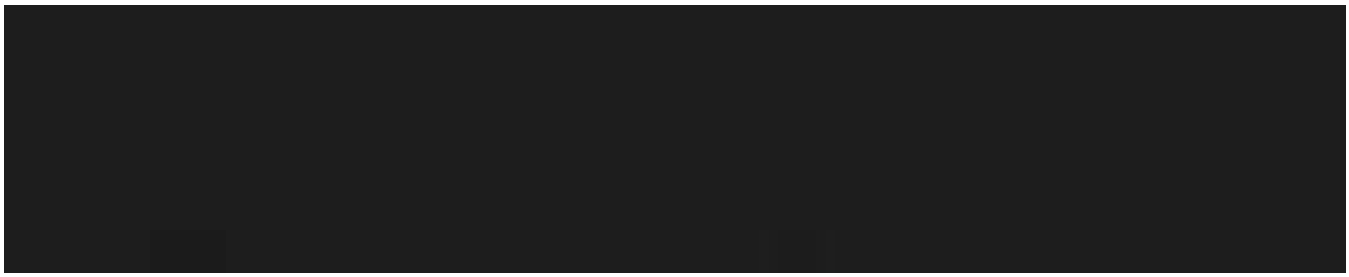

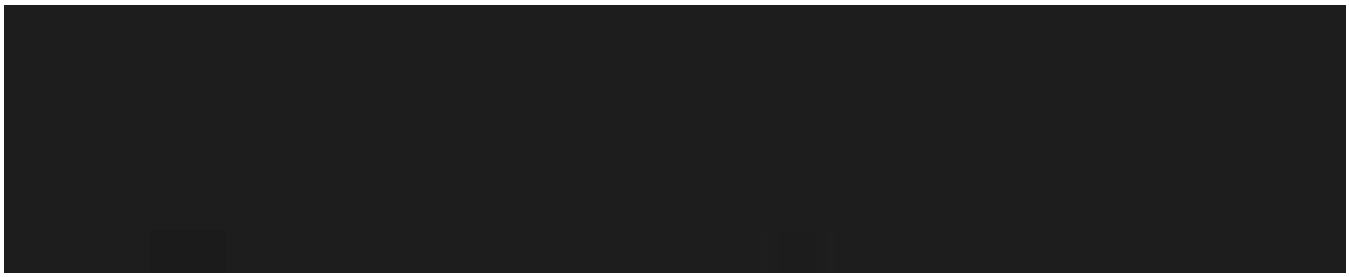

II. Table of Contents

I Signatures 2

II Table of Contents 4

III List of Abbreviations 10

IV Index of Tables 12

V List of figures 12

1. General 18

1.1 Overall overview of the clinical trial – study synopsis 18

1.2 Overview of the course of the study 25

1.3 Visit schedule 26

1.4 Organisational structure – people and institutions involved 31

1.4.1 Investigator and study sites 32

1.4.1.1 Requirements for investigator and study sites 33

1.4.2 Financing 34

2. Introduction 35

3. Medical background and purpose of the study 37

3.1 Chronic non-specific low back pain – prevalence, pathology, and available drug treatment 37

3.2 Cannabis-based medication for pain patients 40

3.3 Rationale for clinical trial VER-CLBP-001 41

4. Study design 43

4.1 Study design – overview 43

4.2 Discussion of the study design 44

4.3 Primary objective 47

4.3.1 Phase A 47

4.3.2 Phase B 47

4.3.3 Phase C 48

4.3.4 Phase D 48
4.4 Secondary and additional objectives 49
4.4.1 Key-secondary endpoint of Phase A 49
4.4.2 Further secondary objectives 49
5. Patients and study sites 50
5.1 Selection of study population and recruitment 50
5.2 Number of patients 50
5.3 Inclusion criteria 50
5.3.1 For the entire study (Phase A-D) 50
5.3.2 Additional inclusion criteria for Phase A 51
5.3.3 Additional inclusion criteria for Phase B 51
5.3.4 Additional inclusion criteria for Phase C 51
5.3.5 Additional inclusion criteria for Phase D 52
5.4 Exclusion criteria 52
5.4.1 Additional exclusion criteria for Phase A 54
5.4.2 Additional exclusion criteria for Phase D 54
5.5 Screen failures 55
6. Investigational medicinal product 56
6.1 Description and name of the investigational medicinal product VER-01 56
6.2 Description and name of the placebo 56
6.3 Production of the IMPs 57
6.4 Distribution of the IMPs 57
6.5 Labelling and packaging of the IMPs 57
6.5.1 Randomised labelling of the IMPs in Phases A and D 58
6.5.2 Open-label labelling in Phases B and C 58
6.6 Storage of the IMPs at the study site 59
6.7 Ordering and dispensing IMP at the study site 59

6.7.1 Ordering and dispensing of blinded IMPs (Phase A and D) 59
6.7.2 Ordering and dispensing of the IMPs (open-label Phase B and C) 60
6.8 Return of the IMPs 60
6.9 Loss of the IMPs 61
6.10 Titration and patient-specific posology 61
7. Conduct of the study 65
7.1 Legal framework conditions 65
7.2 Schedule 66
7.3 Visit schedule 66
7.4 Start and end of the clinical trial 81
7.4.1 Start of participation, briefing process, and consent 81
7.4.2 End of participation 82
7.4.2.1 Regular and premature end of treatment 82
7.4.2.2 Continued treatment after the regular or premature end of treatment 82
7.4.2.3 Procedures in the event of early discontinuation of treatment at the patient's request 83
7.4.2.4 Procedures in the event of early discontinuation of treatment in the investigator's view 83
7.4.3 Procedure for replacing patients 85
7.4.4 End of the clinical trial 85
7.4.4.1 Premature discontinuation of the study at individual study sites 85
7.4.4.2 Cancellation of the entire study by the sponsor 86
7.5 Medical history 87
7.6 Previous and concomitant therapy 87
7.7 Rescue medication 89
7.8 Collection, storage, and pick up of the samples 89
7.9 Recording the 24-hr ECG 91
7.10 Ability to drive 92

7.11 Travelling abroad 92
7.12 Contraceptive methods for study participants 92
7.13 Randomisation and blinding, and assignment to study phases 93
7.13.1 Randomisation R1 to treatment arms in placebo-controlled Phase A 93
7.13.2 Assignment to study Phase C or D and randomisation R2 to treatment groups in placebo-controlled Phase D 95
7.13.3 Blinding 96
7.14 Unblinding via emergency envelopes 96
7.15 Efficacy and safety parameters 97
7.15.1 Measurement of efficacy and safety parameters 97
7.15.1.1 Primary endpoints 97
7.15.1.2 Secondary and additional objectives 98
7.15.2 Overview of the safety and measurement parameters at individual visits 105
7.15.3 Suitability of measurement methods 123
7.16 Ensuring data quality 124
7.16.1 Monitoring 124
7.16.2 Audits/inspections 125
7.17 Documentation 125
7.17.1 Data management 126
7.17.2 Archiving 127
8. Safety 129
8.1 Definitions of adverse events 129
8.1.1 Adverse event 129
8.1.2 Serious adverse event or serious adverse drug reaction 129
8.1.3 Adverse drug reactions 130
8.1.4 Unexpected adverse drug reaction 130
8.1.5 Suspected unexpected serious adverse reaction 130
8.2 Documentation and follow-up of adverse events 130

8.2.1 Documentation of adverse events 131
8.2.2 Intensity of the adverse event 131
8.2.3 Connection between adverse events and the investigational medicinal product or the clinical trial 131
8.3 Notification of suspected unexpected serious adverse reactions and serious adverse events 132
8.4 Notification of a pregnancy 133
8.5 Investigator's report to the CRO for Pharmacovigilance 133
8.6 Second review by the CRO for Pharmacovigilance 134
8.7 Unblinding in relation to a patient 134
8.8 Reporting to institutional review boards/independent ethics committees and responsible federal authorities 134
8.9 Corrective measures 135
8.10 Investigator information about SUSARs 135
8.11 Annual report about the safety of trial subjects 136
8.12 Risk management 136
8.12.1 Anticipated/foreseeable clinical benefits 136
8.12.2 Safety aspects when taking the investigational medicinal product 136
8.12.3 Expected interactions with other pharmaceutical products 137
8.12.4 Risks in association with participation in the clinical trial 138
8.12.4.1 Risk due to blood draw 138
8.12.4.2 Risk of accidents on the way to and from the study site 138
8.12.4.3 Risk due to measurements with the ECG device 139
8.12.4.4 Risk associated with inclusion of elderly patients (without an upper age limit) 139
8.12.5 Risk minimisation measures 140
8.12.6 Risk/benefit justification 140
9. Statistical methods 141

9.1 General considerations 141
9.2 Determination of sample size 142
9.2.1 Determination of sample size for the primary efficacy endpoint in Phase A 142
9.2.2 Determination of sample size for the key-secondary efficacy endpoint in Phase A 143
9.2.3 Determination of sample size for investigation of long-term safety of at least six months in Phase B and 12 months in Phase C 144
9.2.4 Determination of sample size for the efficacy endpoint in phase D 145
9.3 Analysis of patient disposition 146
9.4 Analysis of demographics and baseline characteristics 146
9.5 Definition of intercurrent events 146
9.6 Phase A: Analysis of randomised parallel group comparison 149
9.6.1 Study populations 149
9.6.2 Estimand of the primary endpoint in Phase A 150
9.6.3 Estimands of secondary endpoints Phase A 152
9.6.3.1 Key-secondary estimand: NPSI 152
9.6.3.2 Derived secondary estimands: Pain intensity (NRS) 153
9.6.3.3 Supporting estimands 155
9.6.4 Analysis of efficacy 155
9.6.5 Analysis of safety and tolerability 156
9.6.6 Analysis of the potential for substance dependency and abuse 158
9.6.7 Handling of missing data and strategies for imputation 158
9.7 Phase B: Analysis of open-label extension 160
9.7.1 Study populations 160
9.7.2 Analysis of safety / long-term safety 161
9.7.3 Analysis of the potential for substance dependency and abuse 161
9.7.4 Analysis of efficacy 161
9.7.5 Handling of missing data and strategies for imputation 161

9.8 Phase C: Analysis of open-label extension 162
9.8.1 Study populations 162
9.8.2 Analysis of safety/long-term safety 162
9.8.3 Analysis of the potential for substance dependency and abuse 162
9.8.4 Analysis of effects of sudden drug withdrawal 163
9.8.5 Analysis of efficacy 163
9.8.6 Handling of missing data and strategies for imputation 163
9.9 Phase D: Analysis of maintenance of efficacy 163
9.9.1 Study populations 163
9.9.2 Estimand of the primary endpoint in Phase D 164
9.9.3 Analysis of maintenance of efficacy 165
9.9.4 Analysis of safety 166
9.9.5 Analysis of the potential for substance dependency and abuse 166
9.9.6 Analysis of effects of sudden drug withdrawal 166
9.9.7 Handling of missing data and strategies for imputation 166
9.10 Analysis of prior and concomitant medication 167
9.10.1 Analysis of rescue and concomitant pain medication 167
9.10.2 Analysis of non-drug therapies 168
9.11 Analysis of treatment exposure 168
9.12 Interim analyses 168
10. Changes to the protocol 169
10.1 Revisions 169
11. Ethical and regulatory aspects 170
11.1 Legal regulations observed, guidelines 170
11.2 Reports to authorities, approvals, and registration 170
11.3 Subject insurance 170
11.4 Institutional review boards/independent ethics committees 171

11.5 Ethical conduct of the clinical trial 171
11.6 Data protection 171
12. Use of data and publication 172
12.1 Final report 172
12.2 Publication 172
13. Bibliography 174
13.1 Laws and guidelines 174
14. Measures in the event of the regular conduct of the study being disrupted due to COVID-19 176
14.1 Carrying out patient visits 176
14.2 Carrying out monitoring visits 176

III. List of Abbreviations

Abbreviation	Meaning
ABC	Addiction Behaviour Checklist
ADR	Adverse Drug Reaction
AE	Adverse Event
AMG	Medicinal Products Act
ANCOVA	Analysis of Covariance
approx.	Approximately
BOCF	Baseline Observation Carried Forward
CBC	Complete Blood Count
CBD	Cannabidiol
CLBP	Chronic Low Back Pain
CNS	Central Nervous System
eCRF	Electronic case report form
CT	Clinical trial
CWS	Cannabis Withdrawal Scale
DASS	Depression-Anxiety-Stress Scales
GDPR	General Data Protection Regulation
FPFV	First Patient First Visit
GCP	Good Clinical Practice
IMP	Investigational Medicinal Product
Incl.	Including
ICD-10	International Statistical Classification of Diseases and Related Health Problems
ICH	International Council for Harmonisation of Technical Requirements for Pharmaceuticals for Human Use
ISF	Investigator Site File
ITT	Intention-to-Treat
J2R	Jump to Reference
LLT	Lower level term

LPLV	Last Patient Last Visit
LOCF	Last Observation Carried Forward
M(N)AR	Missing (not) at random
MedDRA	Medical Dictionary for Regulatory Activities
MI	Multiple Imputation
MINI	MINI International Neuropsychiatric Interview
Mil.	Million
MOS-SS	Medical Outcome Study Sleep Scale
PCI	Principal Coordinating Investigator
NRS	Numerical Rating Scale
NSAIDs	Nonsteroidal anti-inflammatory drugs
NPSI	Neuropathic Pain Symptom Inventory
NVL	National treatment guideline
PGIC	Patient Global Impression of Change
PP	Per-Protocol
RCT	Randomised Controlled Trial
RMD	Roland Morris Disability Questionnaire
SADR	Serious Adverse Drug Reaction
SAE	Serious Adverse Event
SAF	Safety Analysis Set
SAP	Statistical analysis plan
SDLL	Source Data Location List
SDV	Source data verification
SF-36	Short- Form 36 health questionnaire
SAE	Serious adverse event
SUSAR	Suspected Unexpected Serious Adverse Reaction
TEAE	Treatment Emergent Adverse Event
THC	Delta-9-tetrahydrocannabinol

IV. Index of tables

Table 1: Tests during the clinical trial 26

Table 2: Visits for additional dispensing of the IMP 29

Table 3: Follow-up Visit 29

Table 4: Requirements from the EMA guideline and implementation into the clinical trial VER-CLBP-001 46

Table 5: Non-opioid and adjuvant analgesics by pharmaceutical class with examples **Fehler! Textmarke nicht definiert.**

Table 6: Examples of non-drug therapies, which significantly modulate the perception of pain **Fehler! Textmarke nicht definiert.**

Table 7: Number of glass bottles dispensed per study phase and visit 59

Table 8: Investigator visits (regular and AID visits) at which full, opened and/or empty amber glass bottles are returned. After being documented, these are stored until the planned pick-up. 60

Table 9: Duration of the individual treatment phases of the study 66

Table 10: Visit schedule 67

Table 11: Investigations during the clinical trial 105

Table 12: Definition of intercurrent events 146

Table 13: Estimand of the primary endpoint in Phase A 150

Table 14: Key-secondary estimand of NPSI total score 152

Table 15: Secondary estimand of pain responders (30%/50%) 154

Table 16: Estimand of the primary endpoint in Phase D 164

V. List of figures

Figure 1: Overview of the duration and purpose of the individual phases of the study. 43

Figure 2: Number of patients (n) during the individual study phases. 50

Figure 3: Distribution of the IMPs: The sponsor is contacted for the order and then forwards the order to the logistics company. They deliver the goods to the relevant study sites. 57

Figure 4: Packaging of the IMPs for the placebo-controlled Phases A and D. To ensure blinding, IMPs are packaged into individual packs and marked with randomisation numbers. Four packs are packaged into one box together with emergency envelopes. Note: number of bottles shown in the illustration is indicative, does not necessarily correspond to the actual number. 58

Figure 5: Dispensing of the blinded IMPs for Phases A and D (Visits A2 and B10) to patients based on randomisation procedure. 60

Figure 6: Instructions for intake in the morning (right) and evening (left) during the titration phase. 63

Figure 7: Graphic representation of the reporting of SAEs 133

1. General

1.1 Overall overview of the clinical trial – study synopsis

**TITLE:** Proof of efficacy, maintenance of efficacy, long-term safety, and investigation of the potential for dependence and abuse and the effect of abrupt drug withdrawal of VER-01 in a multicentre study in the treatment of patients with chronic non-specific low back pain.	
**SPONSOR:**	Vertanical GmbH<br>Am Haag 14, 82166 Gräfelfing, Germany<br>Phone: +49 (0)89 7879790-78, Fax: +49 (0)89 7879790-79
**STUDY NUMBER:**	**VER-CLBP-001**
**EudraCT number:**	**2020-000107-36**
**NAME OF THE INVESTIGATIONAL MEDICINAL PRODUCTS:**	**VER-01 (and placebo)**
**INVESTIGATIONAL MEDICINAL PRODUCT VER-01; TYPE OF APPLICATION:**	• Active substance: Cannabis extract standardised to 5% delta 9-Tetrahydrocannabinol<br>• Excipients: Sesame oil<br>• Pharmaceutical form: Liquid preparation for oral use<br>• Type of application: Oral intake<br>• Dosage: 2.5 mg THC per single dose
**INDICATION:**	For the treatment of patients with chronic non-specific low back pain when drug treatment is indicated and previous optimised treatments with non-opioid analgesics have not led to sufficient pain relief or were unsuitable due to contraindications or intolerance.
**STUDY TYPE:**	Clinical phase III study
**PARTICIPATING COUNTRIES:**	Germany and Austria

STUDY DESIGN:

Division into four phases A, B, C and D:

Phase A: Randomised, double-blind, placebo-controlled titration; then double-blind, placebo-controlled treatment (12 weeks) (proof of efficacy, investigation of the potential for dependence and abuse)

Phase B: Open-label titration with VER-01; then long-term, open-label treatment with VER-01 (26 weeks) (evidence of long-term safety, investigation of the potential for dependency and abuse)

then assigned to Phase C or D according to inclusion criteria

**Phase C**	**Phase D**
Long-term, open-label treatment with VER-01 (additional 26 weeks) (evidence of long-term safety, investigation of the potential for dependency and abuse as well as the effects of sudden drug withdrawal)	Randomised, double-blind, placebo-controlled treatment (four weeks) (proof of maintained efficacy, investigation of the potential for dependency and abuse as well as the effects of sudden drug withdrawal)

STUDY DURATION:

- Planned start of recruitment: March 2021
- Enrolment of first patient: July 7, 2021
- Anticipated end (last patient last visit): June 2024

STUDY OBJECTIVE:

Proof of efficacy, maintained efficacy and long-term safety of VER-01 in the treatment of chronic non-specific low back pain where drug treatment is indicated and previous optimised treatments with non-opioid analgesics have not led to sufficient pain relief or are unsuitable due to contraindications or intolerance and the investigation of potential for dependence and abuse as well as the effect of sudden drug withdrawal.

PRIMARY END POINTS:

Phase A: Change in average pain intensity in the morning on an 11-point NRS compared to baseline (mean value of study Week 15 compared to the mean value of the seven days before Visit A2 (Study Week -1) with daily documentation of pain intensity in the diary by the patient)

Phase B: Safety and adverse reactions based on occurrence of treatment-related AEs/SAEs

Phase C: Safety and adverse reactions based on occurrence of treatment related AEs/SAEs

Phase D: Time until treatment failure defined as the time in days from randomization to Phase D (R2) until the first day of treatment failure. Treatment failure is assessed by the daily calculated seven-day mean value of the NRS pain score in the morning during the treatment period, which must have deteriorated by at least 20% and at least one point compared to baseline (mean value of the pain score in the morning of study Week 43). The first day of treatment failure is then the earliest day within this seven-day time window to which this criterion applies as a single day. Furthermore, treatment failure is defined as a premature discontinuation of treatment for selected reasons.

PROCESS:

Phase A: One week run-in, three weeks of randomised, double-blind, placebo-controlled titration with VER-01 or placebo; then 12 weeks double-blind, placebo-controlled treatment with VER-01 or placebo.

Phase B: Open-label, three weeks of titration with VER-01; 26 weeks of treatment with VER-01

then assigned to Phase C or D according to inclusion criteria

Phase C:

Open-label, 26 weeks of treatment with VER-01, two weeks wash-out (sudden withdrawal of the IMPs)

Phase D:

Randomised, double-blind, placebo-controlled, four weeks of treatment with VER-01 or placebo, two weeks wash-out (sudden withdrawal of the IMP)

TITRATION PHASE REQUIREMENTS:

- On Day 1: Start with 1 dose unit in the evening (2.5 mg THC)
  On Day 4: in addition to the dose taken in the evening, start with 1 dose unit in the morning (2.5 mg THC)
- Each dose will stay the same for at least three days; increase the dose according to the titration schedule until the patient-specific effective dose is reached (see Annex 1 "Titration phase instructions")
- Maximum 7 dose units in the evening (17.5 mg THC)
  Maximum 6 dose units in the morning (15 mg THC)
- If serious or unbearable adverse reactions arise, the patient must inform the investigator immediately and, if necessary, reduce the dose or stop taking it.
- Taken in the evening: Taken between 4pm and going to bed
- Taken in the morning: Taken between waking up and 12pm

TREATMENT PHASE REQUIREMENTS (A, B, C, D):

- VER-01 or placebo is withdrawn immediately in the event of early study discontinuation.
- In treatment Phases A and B, the patient-individual effective daily dose of VER-01 or placebo determined in the titration phase can be adjusted upwards or downwards by the patient if their condition or concomitant medication changes or adverse reactions occur causing discomfort. It is taken in the evening and morning, similarly to the titration phase.
  At the start of treatment phases C and D, the dose should be kept the same as in treatment phase B. The patient can then adjust the dose upwards or downwards if their condition or concomitant medication changes or if adverse reactions that cause discomfort occur. It is taken in the evening and morning, similarly to the titration phase.
- The single dose should not exceed 8 dose units (20 mg THC) and the maximum daily dose should not exceed 13 dose units (32.5 mg THC) during the treatment phases. The minimum daily dose is 1n (2.5 mg THC), to be taken in the evening.
- Taken in the evening: Taken between 4pm and going to bed
- Taken in the morning: Taken between waking up and 12pm

WASH-OUT PHASE REQUIREMENTS:

- VER-01 or placebo is suddenly withdrawn at Visit C13 (Phase C) or at Visit D11 (Phase D).

STUDY POPULATION/DIAGNOSIS:

Inclusion criteria:

1. Male and female patients (18 years and older)
2. Chronic (for at least three months) non-specific pain in the lower back (between the lower ribcage and the gluteal folds)
3. Pain intensity on average at least 4 points on an 11-point NRS (one month before the start of the study)
4. Patients with indicated drug treatment* where previous optimised treatments** with non-opioid analgesics have not led to sufficient pain relief or were unsuitable due to contraindications or intolerance.

   ** Drug treatment is indicated if analgesic drug therapy is considered supportive for the realisation of activating measures, or if the patient has unbearable functional disabilities as a result of the pain, despite regularly performing these measures.*

   *** Treatment is considered optimised when*

   I. a further increased drug dose is unsuitable from a medical perspective considering side effects and/or

   II. it is not expected that a higher drug dose would result in a further advantage in terms of efficacy.
5. Willingness of both men and women to use a reliable method of contraception during study participation and for three months after taking the last dose of the IMP
6. Signed patient information and informed consent form is available
7. Understanding of the local language, compliance and ability to give consent
8. The patient has understood the instructions to avoid changes in lifestyle and dietary habits
9. The patient has understood the principle of the patient diary and gives their consent to keep it as instructed

Additional for Phase A

a1. Average score of pain intensity in the morning in the 7 days before Visit A2 (Study Week -1) must be at least 4 points on an 11-point NRS. For the calculation, the last 7 pain intensity entries before Visit A2 are taken from the patient diary (there must be at least 5 pain intensity readings in the morning from Study Week -1).

a2. Willingness not to take any additional analgesic medication (non-opioid and opioid analgesics as well as adjuvant analgesics) during participation in study Phase A (except rescue medication)

a3. Willingness to continue a current non-drug therapy unchanged as planned during participation in Phase A

Additional for Phase B

b1. Previous and complete participation in Phase A until and including Visit A6

b2. Patient wishes to participate voluntarily in the long-term study

b3. From the investigator's point of view, further participation is considered medically safe

b4. Willingness not to take any additional analgesic medication (non-opioid and opioid analgesics as well as adjuvant analgesics) during the last three weeks of study Phase B (except rescue medication).

Additional for Phase C

c1. Previous and complete participation in Phase B until and including Visit B10

c2. Patient wishes to participate voluntarily in the long-term study

c3. From the investigator's point of view, further participation is considered medically safe

Additional for Phase D

d1. Previous and complete participation in Phase B until and including Visit B10

d2. Patient has experienced a morning pain score improvement of at least 30% in treatment Phase B (mean morning pain score value of study Week 43 compared to the mean morning pain score value in the seven days before Visit A2 (Study Visit -1), there must be at least four values from study Week 43 and five values from Study Week -1)

d3. Patient wishes to participate voluntarily in the study

d4. From the investigator's point of view, further participation is considered medically safe

d5. Willingness not to take any additional analgesic medication (non-opioid and opioid analgesics as well as adjuvant analgesics) during participation in study Phase D (except rescue medication)

d6. Willingness to continue a current non-drug therapy unchanged as planned during study participation in Phase D

d7. The patient took the investigational medicinal product on at least 5 out of 7 days in Study Week 43 (daily dose min. 1 n) and documented taking the investigational medicinal product in the patient diary.

Exclusion criteria:

1. Professional groups for which the ability to operate machinery and drive vehicles is the primary activity (including truck, bus and forklift drivers, pilots)
2. Alcohol/drug/medication abuse and previous or current intake of methadone in the patient's medical history or suspected by the investigator
3. Intake of analgesic medication (non-opioid and opioid analgesics as well as adjuvant analgesics) within seven days prior to the start of the study
4. Taking cannabis-based medicinal products within 30 days prior to the start of the study
5. HIV, dementia (which impairs the assessment of symptoms)
6. Severe forms of the following diseases: Anaemia, hematological/autoimmune/endocrinal/ renal/hepatic/respiratory/cardiovascular or gastrointestinal diseases, symptomatic peripheral vascular diseases
7. Cardiovascular event in the past three months, poorly managed high blood pressure, untreated hypothyroidism, patients with Crigler-Najjar syndrome or Rotor syndrome, surgery within the past two months

8. Severe mental illnesses (e.g., psychosis, schizophrenia, bipolar disorder) currently or in the past, severe depression currently or in the past that is/was not due to the chronic non-specific low back pain, or individuals at risk of suicide (examined using the MINI questionnaire: as soon as at least one module is complete)
9. Severe mental illness (psychosis, schizophrenia, bipolar disorder, severe depression, anxiety disorder) currently or in the past in a first-degree relative (parents and children); suicide in a first-degree relative (parents and children)
10. Patients with an active cancer or tumour-related pain or strong pain due to physical injuries
11. Other painful comorbidities, excluding low back pain, that could interfere with the patient's evaluation during the study or the assessment of pain
12. Well-known strong adverse events in connection with cannabis consumption before the start of the study
13. Known allergy to cannabis and/or sesame seeds and products derived from them
14. Known hypersensitivity to the ingredients of the rescue medication
15. Planned blood donation, planned sperm or egg donation, planned freezing of eggs or sperm
16. Pregnancy, breastfeeding, desire to have children (within the next 20 months)
17. Participation in another interventional clinical trial within the past 30 days before the start of the study
18. Inability to give consent, care dependency, patient has a legal guardian/caregiver, or is immobile
19. The patient is in need of special protection (e.g., incarcerated; institutionalized by a court or judicial authority; in a dependent or employment relationship with the sponsor, an external service provider of the sponsor (who is involved in the study conduct), the investigator, or the study site).

Additional for Phase A:

a1. In the case of a current non-drug therapy (e.g., physical or behavioural therapy, acupuncture, massage, thermotherapy), which significantly modulates the perception of pain, it was not maintained unchanged for at least eight weeks prior to study participation in Phase A.

Additional for Phase D

d1. Intake of additional analgesic medication (non-opioid and opioid analgesics as well as adjuvant analgesics) within 21 days prior to the start of study Phase D (except rescue medication).

d2. In the case of a current non-drug therapy (e.g., physical or behavioural therapy, acupuncture, massage, thermotherapy) that significantly modulates the perception of pain, it was not maintained unchanged for at least nine weeks prior to the start of study Phase D.

•

NUMBER OF PATIENTS (planned):

Phase A: 808 (of these, 180 with a painDETECT Score > 18) patients randomised to treatment with VER-01 or placebo in a 1:1 ratio

Phase B: 500 patients at Visit B7 (open-label VER-01)

Phase C: A minimum of 150 patients at Visit B10 for Phase C (open-label VER-01)

Phase D: A minimum of 80 patients at Visit B10 randomised to treatment with VER-01 or placebo in a 1:1 ratio with the goal to include up to 120 patients

DETERMINATION OF THE SAMPLE SIZE:

The sample size was determined with the aim of ensuring sufficient statistical power to evaluate the primary and key-secondary endpoints for Phase A, sufficient patient numbers to assess long-term safety and sufficient statistical power to evaluate the primary endpoint for Phase D. Taking into account the multi-phase study design, the total number of patients to be randomised into the study is 808 patients.

Primary endpoint to demonstrate efficacy (Phase A): To show superiority of treatment with VER-01 vs. placebo on a two-sided significance level of 5% and with 90% statistical power, 732 patients need to be randomly assigned in a 1:1 ratio to one of the two treatment groups in Phase A (VER-01 vs. placebo).

Key-secondary endpoint to demonstrate efficacy in patients with a neuropathic pain component (Phase A): To show superiority of treatment with VER-01 vs. placebo on a two-sided significance level of 5% and with 80% statistical power, 180 patients with a painDETECT Score > 18 need to be randomly assigned in a 1:1 ratio to one of the two treatment groups in Phase A (VER-01 vs. placebo). Since 22.2% of the patients have a neuropathic pain component, the total necessary sample size is 808 patients.

Investigation of long-term safety for at least six months: Assuming a drop-out rate of 40% during Phase B, 500 patients should be enrolled in Phase B, so that 300 patients complete Phase B. This sample size is sufficient to meet the requirement of available long-term safety data for at least six months.

Primary endpoint for maintenance of efficacy (Phase D): Assuming 25% treatment failure with VER-01 and 55% treatment failure after switching to placebo, the logrank test comparing the two (exponential) survival curves for time to treatment failure at a two-sided significance level of 5% requires a sample size of 78 (39 per treatment group) to achieve a power of 80%. Therefore, at least 80 patients should be included in Phase D (40 patients with a painDETECT Score above 12 and 40 patients with a painDETECT Score smaller than or equal to 12).

NUMBER OF STUDY SITES (planned):

Approx. 100 sites in Germany and Austria

PROVISION OF THE IMP:

The sponsor supplies VER-01 and placebo in the form of an oil that is identical in terms of appearance, taste, and smell.

1.2 Overview of the course of the study

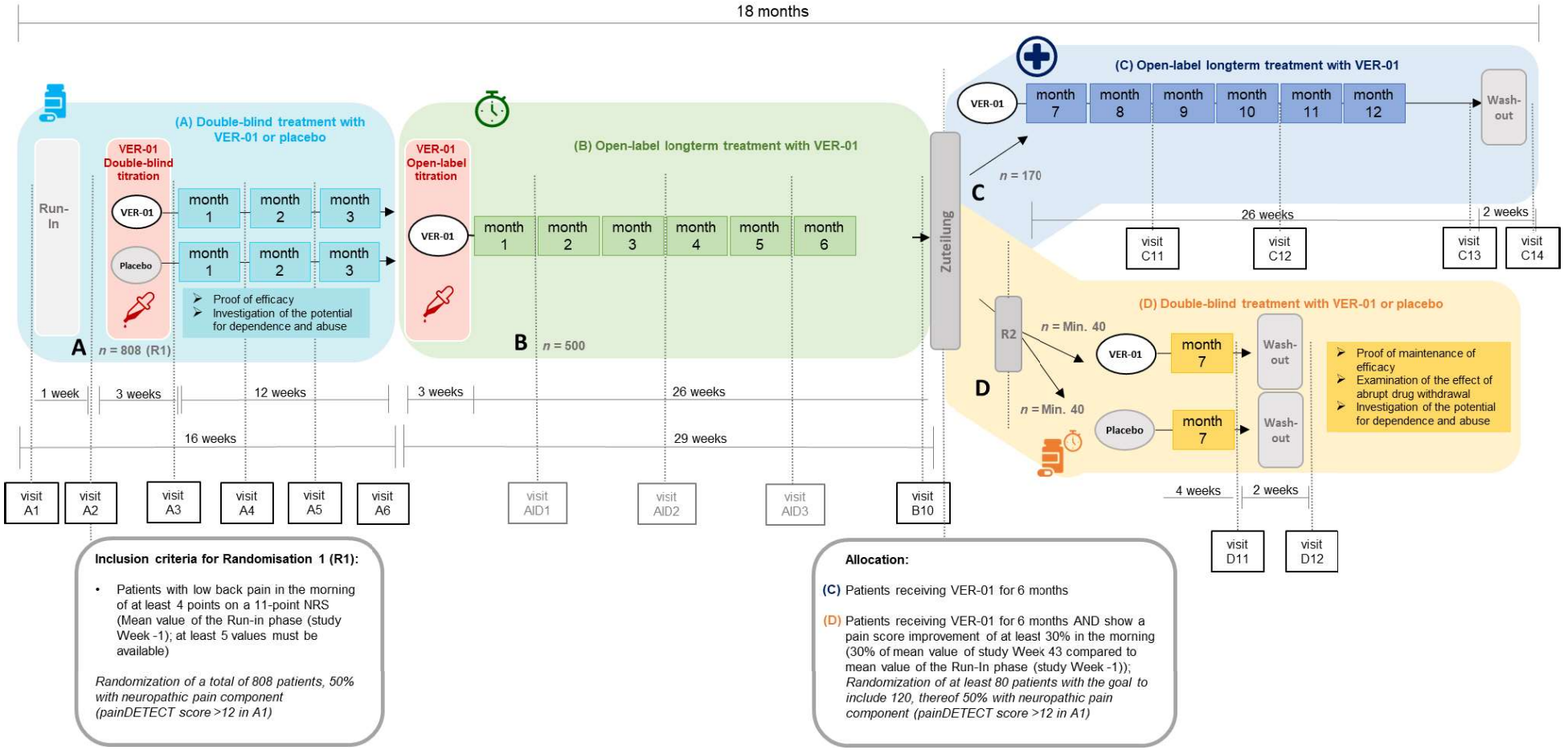

1.3 Visit schedule

Description of individual visits:

Table 1: Tests during the clinical trial

	Phase A						Phase B				Phase C				Phase D	
**Study day [weekdays] (+/± possible tolerance [week-days])**	-8	1 (+3)	22 (+3)	50 (±3)	78 (±3)	106 (+3)	127 (+3)	183 (±3)	239 (±3)	309 (+3)	365 (±3)	421 (±3)	491 (±3)	505 (±3)	337 (+3)	351 (±3)
**Study week**	-2	1	4	8	12	16	19	27	35	45	53	61	71	73	49	51
**Visit**	A1	A2	A3	A4	A5	A6	B7	B8	B9	B10	C11	C12	C13	C14	D11	D12
Information and inclusion of the patient																
Information and consent to study participation	x									x						
Determination of medical history and concurrent diseases	x															
Fulfilment of inclusion and exclusion criteria	x	x				x				x						
Documentation of demographic data	x															
Issuance of the notes on concomitant therapy	x															
Study-specific examinations																
Physical examination	x		x			x				x			x	x	x	x
Drug test (urine test): THC, cocaine, morphine, metham-phetamine, amphetamine, methadone, benzodiazepine, ecstasy	x						x			x				x		
Drug test (urine test): cocaine, morphine, methampheta-mine, amphetamine, methadone, benzodiazepine, ec-stasy																x

	A1	A2	A3	A4	A5	A6	B7	B8	B9	B10	C11	C12	C13	C14	D11	D12
Blood draw (to determine a complete blood count and clinical chemistry parameters)	x					x	x			x			x			
Pregnancy test		x	x	x	x	x	x	x	x	x	x	x	x		x	
24-hour electrocardiogram (on 120 patients): in addition to A2 + A5, Day 1 of titration Phase A after taking the 1st dose		x			x											
Measurement of vital signs	x	x	x	x	x	x	x	x	x	x	x	x	x	x	x	x
Questionnaires and data collection																
Assessment of quality of life by the patient (SF-36)		x	x	x	x	x				x			x		x	
Assessment of sleep quality by the patient (MOS-SS)		x	x	x	x	x										
Assessment of disability and symptoms by the patient (RMD)	x	x				x										
Detection of neuropathic pain (painDETECT)	x															
Measurement of neuropathic pain (NPSI)	x	x	x	x	x	x	x	x	x	x	x	x	x	x	x	x
Assessment of the treatment results by the investigator						x				x			x		x	
Global assessment of symptoms by the patient						x				x			x		x	
Assessment of the treatment results and of tolerability by the patient						x				x			x		x	
Assessment of substance dependence by the investigator (ABC)			x	x	x	x	x	x	x	x	x	x	x		x	
	A1	A2	A3	A4	A5	A6	B7	B8	B9	B10	C11	C12	C13	C14	D11	D12

Assessment of substance dependence by the investigator, with potential diagnosis													x		x	
Determination of psychological disorders (MINI)	x															
Study product and rescue medication																
Rescue medication and pantoprazole given out (more can be given out if required (x))	x	(x)	(x)	(x)	(x)	(x)	(x)	(x)	(x)	(x)	(x)	(x)	(x)		(x)	
Documentation of the intake of rescue medication and pantoprazole, if applicable		x	x	x	x	x	x	x	x	x	x	x	x	x	x	x
Dispensation of the IMP (additionally for Visits AID1-AID8)		x	x	x	x	x	x	x	x	x	x	x				
Return of the IMP (additionally for Visits AID1-AID8) and check of compliance (IMP taken correctly)			x	x	x	x	x	x	x	x	x	x	x		x	
Documentation of rescue medication and concomitant therapies		x	x	x	x	x	x	x	x	x	x	x	x	x	x	x
eDiary																
Issuance of access data for e-Diary and instruction	x															
Checking the e-Diary		x	x	x	x	x	x	x	x	x	x	x	x	x	x	x
Patient safety																
Possible early discontinuation from study (additionally for Visits AID1-AID8)	x	x	x	x	x	x	x	x	x	x	x	x	x	x	x	x
Documentation of AEs/SAEs		x	x	x	x	x	x	x	x	x	x	x	x	x	x	x

*Patients' entries in the eDiary must also be checked between the visits, in particular in relation to SAEs

Table 2: Visits for additional dispensing of the IMP

**Study phase**	**Phase B**				**Phase C**			
**Study day [weekdays]**<br>**(+/± possible tolerance [weekdays])**	**155 (±3)**	**211 (±3)**	**267 (±3)**	**295 (±3)**	**337 (±3)**	**393 (±3)**	**449 (±3)**	**477 (±3)**
**Study week**	**23**	**31**	**39**	**43**	**49**	**57**	**65**	**69**
**Visit**	**AID 1**	**AID 2**	**AID 3**	**AID 4**	**AID 5**	**AID 6**	**AID 7**	**AID 8**
Return of the IMP and check of compliance (IMP taken correctly)	x	x	x	x	x	x	x	x
If applicable, return and documentation of rescue medication taken and any dispensing of rescue medication and pantoprazole (if necessary (x))	(x)	(x)	(x)	(x)	(x)	(x)	(x)	(x)
Dispensation of the IMP	x	x	x	x	x	x	x	x
Pregnancy test	x	x	x		x	x	x	
Documentation of AEs/SAEs	x	x	x	x	x	x	x	x
Possible early discontinuation from study	x	x	x	x	x	x	x	x

Table 2: Follow-up Visit

**Study day [weekdays]**<br>**(+/± possible tolerance [weekdays])**	**+ 14 (±3) days after study drop-out*/after Visit A6 or B10, in patients who do not participate in a subsequent study phase**
Physical examination	x
Measurement of vital signs	x
Drug test (urine tests): Cocaine, morphine, methamphetamine, amphetamine, methadone, benzodiazepine and ecstasy	x

Return of any investigational medicinal products and check of compliance (until study drop-out)	x
Return of any rescue medication taken and documentation of rescue medication taken (until study drop-out)	x
If applicable, assessment of the treatment results and of tolerance by the patient	x
Global assessment of symptoms by the patient	x
Assessment of the treatment results by the investigator	x
Documentation of AEs/SAEs	x
Documentation of concurrent medications	x

*A follow-up visit should only be held in the event of study dropout after the investigational medicinal product has been dispensed (in the event of a screen failure, no follow-up visit takes place)

1.4 Organisational structure – people and institutions involved

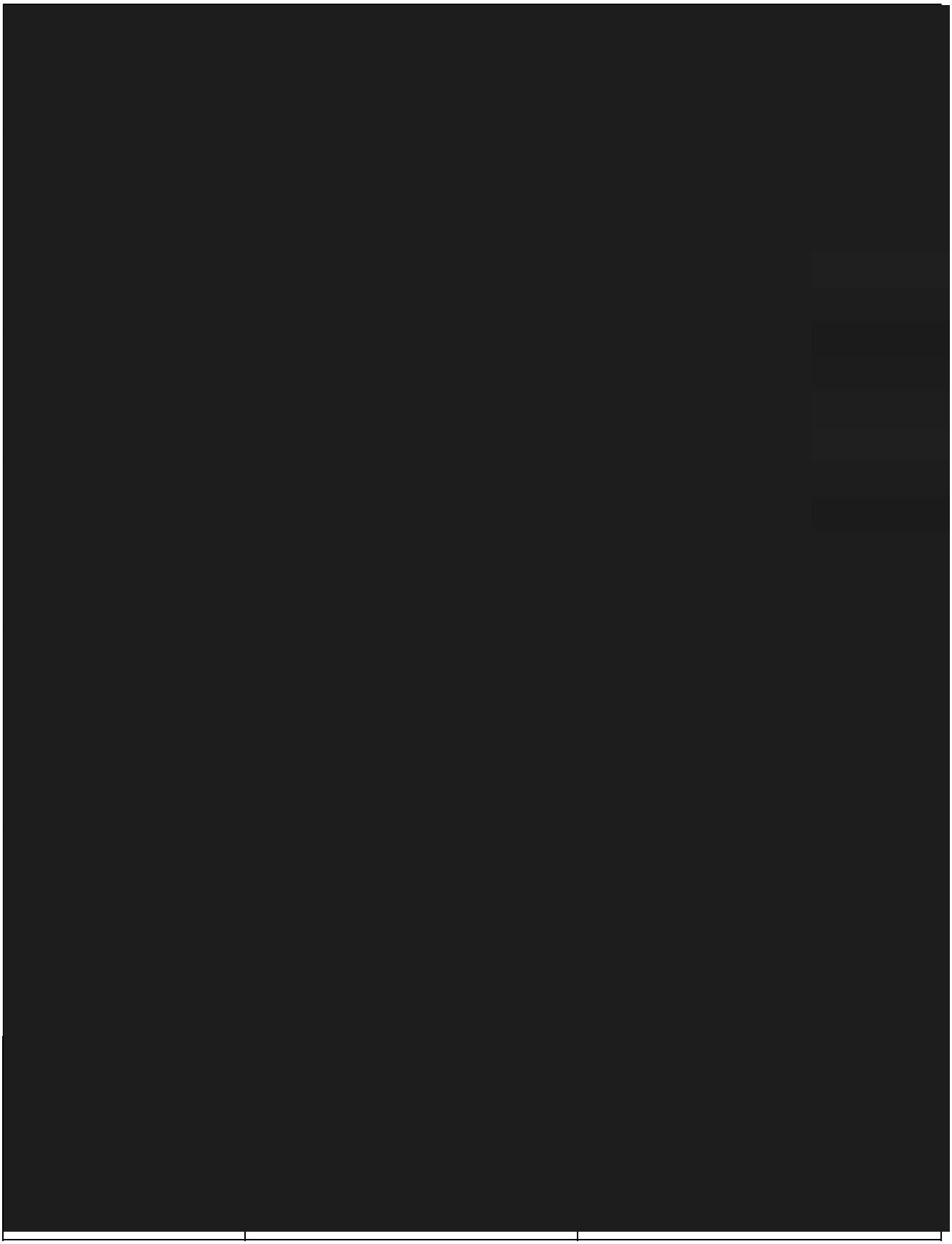

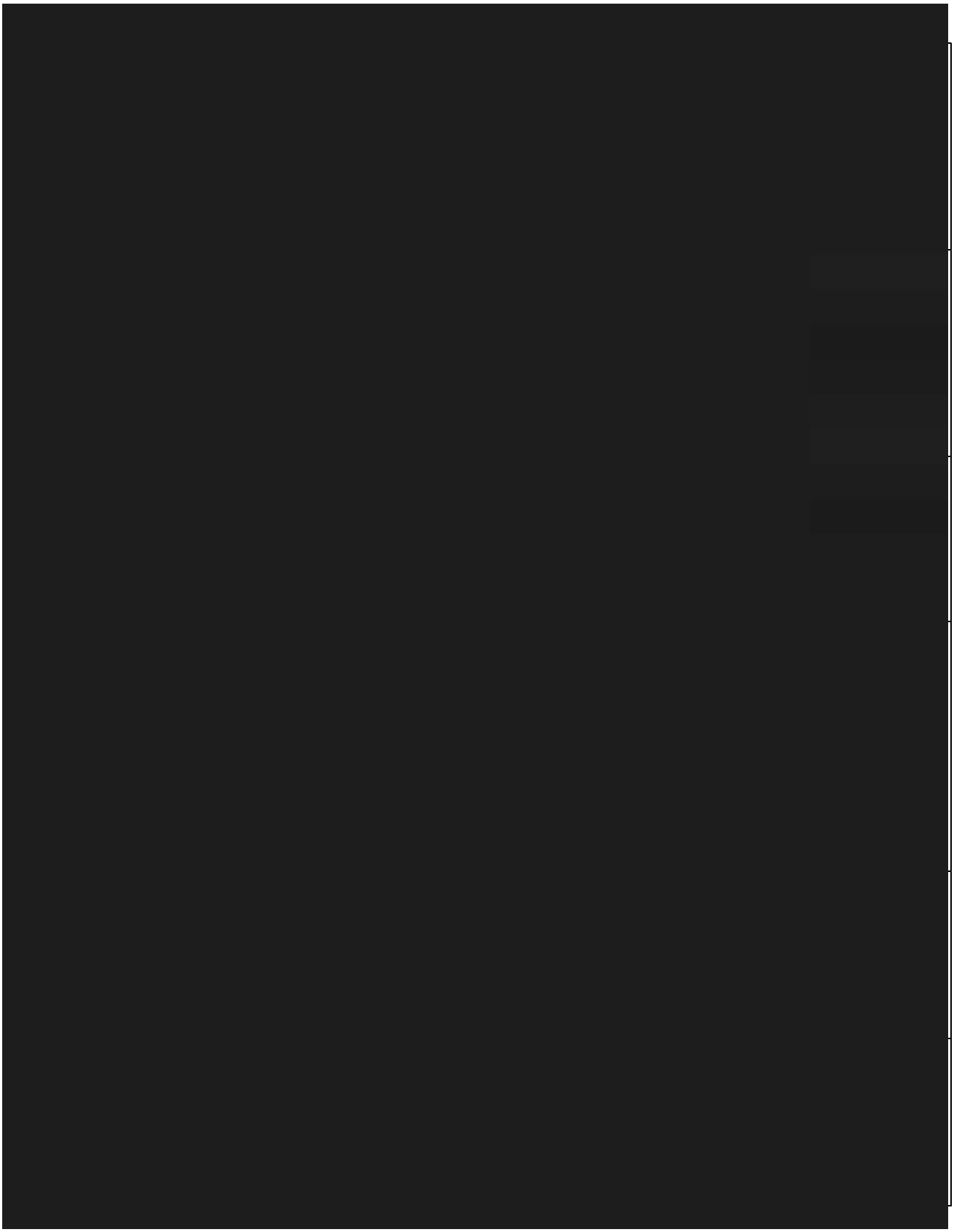

1.4.1 Investigator and study sites

The clinical trial is multicentre and is expected to be conducted in a total of 100 study sites in Germany and Austria.

The Principal Coordinating Investigator (NCI) for Austria and Germany will set up one study site each. The information about study sites, investigators, their deputies, and further medical members of the trial group is updated on an ongoing basis in a separate list. The finalised list will be enclosed with the final clinical trial report.

1.4.1.1 Requirements for investigator and study sites

The following documents provide evidence of the investigator's and the study sites' qualification.

Requirements for investigator and deputies:

- Current, professional CV (dated and signed)
- Information about clinical studies already conducted and other studies with patient recruitment
- Experience in the indication under investigation
- Evidence of training in general principles and rules for clinical studies, especially AMG and the ICH-GCP guideline
- Information about potential financial and other interests in connection with the IMP
- Evidence of two years of experience in conducting studies (for NCI only)

Requirements for members of the trial group (medical and non-medical members of the trial group apart from the principal investigator and deputy):

- Clinical experience and information about experience in conducting clinical studies
- Information about regulatory knowledge, evidence of training where applicable

Requirements for study sites:

- Information about room conditions, tools, devices, focuses of treatment and patient numbers in the indication being investigated
- Information about clinical studies already conducted, ongoing and planned at the study site
- Compliance with the applicable regulatory requirements (especially regarding storage and handling of the IMP)
- It is desirable for the study sites to have been certified in accordance with DIN ISO or equivalent national certification. If certification is not in place, processes of employee instruction and training should be documented in the form of SOPs or suitable descriptions. In addition, process descriptions for standard of care in the indication should be established. The study site must have the equipment and personnel required for the conduct of the

study. At the study site, the investigator and their deputy must be qualified in line with national requirements.

- Dedicated rooms should be available for the examinations.
- The recruitment potential of the study sites is set at a minimum of 4 patients. This requires sufficient patients with chronic non-specific low back pain and relevant prior treatment to be treated at the site.
- All sites are inspected for suitability as a study site as part of a screening visit by the sponsor or a monitor appointed by the sponsor. The information about study sites is documented on an ongoing basis. The final documentation will be enclosed with the final clinical trial report.

1.4.2 Financing

The clinical trial is sponsored by Vertanical GmbH.

2. Introduction

Chronic low back pain has been dominating the statistics of the most common pain syndromes for years and represents the second most common cause of physical and functional impairment globally. Prevalence worldwide has doubled over the past ten years.[1] In Germany, approx. 20% of the population suffers from chronic low back pain.[2] Over 20 million low back pain patients are treated in Germany every year, which makes low back pain one of the most expensive health conditions.[3] In order to understand the disease better, in 2020 the German Pain Association started the PAIN2020 project, along with Barmer health insurance, to investigate chronic pain patients over a period of three years. This project is sponsored by the innovation fund of the Federal Joint Committee (GBA) with €7 million and highlights the need for action to be taken for these symptoms.[4]

Low back pain refers to pain between the lower ribcage and the gluteal folds. It is considered chronic if the pain lasts for more than three months.[5] In around 90% of cases, the cause is uncertain and is therefore defined as non-specific.[6] Current treatment options are limited to the short-term use of non-opioid analgesics. If they do not lead to the desired improvement of the condition, the only available long-term treatment are opioids, a group of pharmaceuticals that can cause strong adverse drug reactions and hold a high addictive potential. In clinical practice, there is no better tolerated, scientifically validated alternative for the long-term drug treatment of patients with chronic non-specific low back pain.

Vertanical GmbH is therefore developing a prescription medication based on a cannabis extract standardised to 5% delta-9-tetrahydrocannabinol (THC) as an alternative to opioids. The study described here aims to demonstrate the efficacy of the IMP (development name VER-01) in the treatment of patients with chronic non-specific low back pain. The study is divided into four phases, in which efficacy, safety, maintained efficacy, potential for dependence and abuse and possible withdrawal symptoms following sudden withdrawal of VER-01 are investigated in comparison to placebo. The primary efficacy endpoint is defined as the change in average pain intensity in the morning from baseline on an 11-point NRS (mean value of the last seven days of the treatment phase (study Week 15) compared

[1] Allegri M, Montella S, Salici F et al. Mechanisms of low back pain: a guide for diagnosis and therapy. Research 2016, (doi: 10.12688/f1000research.8105.2)

[2] Robert Koch Institut (RKI). Gesundheit in Deutschland. Gesundheitsberichterstattung des Bundes. Gemeinsam getragen von RKI und Destatis. Berlin: RKI; 2012.

[3] Schmidt CO, Kohlmann T. What do we know about the symptoms of back pain? Epidemiological results on prevalence, incidence, progression and risk factors. Z Orthop Ihre Grenzgeb 2005;143(3): 292 - 298. DOI:10.1055/s-2005-836631

[4] https://www.pain2020.de/ Last revised: 19.11.2021

[5] Koes, B. W., MWm Van Tulder, and S. Thomas. "Diagnosis and treatment of low back pain." Bmj 332.7555 (2006): 1430-1434.

[6] Nationale Versorgungsleitlinie Nicht-spezifischer Kreuzschmerz, AWMF, 2. Auflage Version 01 2017

to the mean value of the seven days before Visit A2 (study Week -1) with daily documentation of pain intensity in the morning). The mean change in pain intensity versus baseline is compared between VER-01 and placebo treatment. The goal is to demonstrate statistically significant superiority for the VER-01 treatment arm.

3. Medical background and purpose of the study

3.1 Chronic non-specific low back pain – prevalence, pathology, and available drug treatment

One of the biggest health problems in western industrial nations is low back pain.[7,8,9,10] Alongside headaches, low back pain ranks among the most common pain problems.[11] In a study by the Robert Koch Institute, 20.7% of those surveyed had been suffering from chronic low back pain for three months or more. The low back pain study in 2003/2006 found lifetime prevalence of between 74% and 85% in Germany (diagnosis of low back pain at least once during life-time), with 7% of those surveyed suffering from severe and 9% from considerably debilitating low back pain.[12] In France, a prevalence of non-specific chronic low back pain of between 15% and 45% was reported, between 6.3% and 11.1% in the United Kingdom, 14.8% in Spain and the prevalence of chronic low back pain is estimated at 13.1% in the USA.[13] The prevalence has doubled in the last decade worldwide.[14]

According to the German telephone health survey 2009/2010, the frequency of chronic low back pain increases with age. Only 11% of those under 30 years, but 30% of those over 65 years suffer from chronic low back pain. The studies remain divided on gender distribution, with the Robert Koch Institute reporting slightly higher prevalence among women than men (25% vs. 17%), whereas a US study showed slightly higher prevalence among men. One thing that is common to the studies is that, with

[7] Allegri M, Montella S, Salici F et al. Mechanisms of low back pain: a guide for diagnosis and therapy. Research 2016, (doi: 10.12688/f1000research.8105.2)

[8] Windhager R, Radl R. Der Rückenschmerz. Österreichische Ärztezeitung. Universitätsklinik für Orthopädie, Medizinische Universität Graz, 2006

[9] Webb R, Brammah T, Lunt M, Urwin M, Allison T, Symmons D. Prevalence and Predictors of Intense, Chronic, and Disabling Neck and Back Pain in the UK General Population. Spine 2003;28:1195–1202

[10] Parthan, A., Evans, C. J., & Le, K. (2006). Chronic low back pain: epidemiology, economic burden and patient-reported outcomes in the USA. Expert Review of Pharmacoeconomics & Outcomes Research, 6(3), 359–369. doi:10.1586/14737167.6.3.359

[11] Göbel H. Epidemiology and costs of chronic pain syndromes exemplified by specific and unspecific low back pain. Schmerz 2001; 15(2): 92 - 98

[12] Robert Koch Institut (RKI). Gesundheit in Deutschland. Gesundheitsberichterstattung des Bundes. Gemeinsam getragen von RKI und Destatis. Berlin: RKI; 2012.

[13] Allegri M, Montella S, Salici F et al. Mechanisms of low back pain: a guide for diagnosis and therapy.

[14] Juniper, M., Le, T. K., & Mladsi, D. (2009). The epidemiology, economic burden, and pharmacological treatment of chronic low back pain in France, Germany, Italy, Spain and the UK: a literature-based review. Expert Opinion on Pharmacotherapy, 10(16), 2581–2592. doi:10.1517/14656560903304063

regard to the influence of socioeconomic factors, people with lower social status (measured by education, profession and income) complained more often about low back pain than people of middle or high social status, regardless of age.[15,16,17]

Low back pain also plays an important role economically. For years, it has been one of the main reasons for missed work, medical rehabilitation, and early retirement due to diminished capacity for work in western industrial nations.[18] Due to the high prevalence and indirect costs, chronic low back pain is one of the most expensive conditions to treat.[1] The direct and indirect costs due to non-specific low back pain amounted to €3.6 billion in Germany alone in 2008.[19] In the USA, the costs for the year 2006 were estimated at $100 billion.[20]

Low back pain is defined as pain that develops below the ribcage and above the gluteal folds, with or without radiation into other regions. The pathology can be classified based on the cause, duration, and severity. A distinction is made between the causes of specific and non-specific low back pain. Specific low back pain is the result of a clearly defined cause such as a fracture, a tumour, or osteoporosis and is treated with specific causative treatments. If a cause for the pain cannot be found with simple, clinical methods, it is regarded as non-specific low back pain, which concerns around 90% of cases. Depending on the duration of pain, low back pain is classified as acute, subacute, or chronic. If the pain is consistent for over three months, it is considered chronic.[21,22] Chronic low back pain is a complex mixed pain concept with two different pain components: nociceptive and neuropathic pain.[23] Nociceptive pain is the result of inflammatory processes, tissue damage, or excess strain on muscles, joints, fasciae, ligaments or tendons, which activates local nociceptors. In contrast, neuropathic pain is caused by compression and/or damage to nerve fibres. It is suspected that the neuropathic form of the pain is responsible for the ineffectiveness of classic painkillers such as paracetamol or NSAIDs in half of all

[15] Robert Koch Institut (RKI). Gesundheit in Deutschland. Gesundheitsberichterstattung des Bundes. Gemeinsam getragen von RKI und Destatis. Berlin: RKI; 2015. http://www.rki.de/DE/Content/Gesundheitsmonitoring/Gesundheitsberichterstattung/GesInDtld/gesundheit_in_deutschland_2015.pdf.

[16] Aggarwal S, Carter G, Sullivan M, et al. Characteristics of patients with chronic pain accessing treatment with medical cannabis in Washington State. J Opoiod Manag 2009

[17] Nationale Versorgungsleitlinie Nicht-spezifischer Kreuzschmerz, AWMF, 2. Auflage Version 01 2017

[18] Schmidt CO, Kohlmann T. What do we know about the symptoms of back pain? Epidemiological results on prevalence, incidence, progression and risk factors. Z Orthop Ihre Grenzgeb 2005;143(3): 292 - 298. DOI:10.1055/s-2005-836631

[19] Robert Koch Institut (RKI), Raspe H. Rückenschmerzen. Berlin: RKI; 2012 (Gesundheitsberichterstattung des Bundes; 53).

[20] J Bone Joint Surg Am. 2006. Katz JN, Lumbar disc disorders and low-back pain: socioeconomic factors and consequences.

[21] Nationale Versorgungsleitlinie Nicht-spezifischer Kreuzschmerz, AWMF, 2. Auflage Version 01 2017

[22] Koes, B. W., MWm Van Tulder, and S. Thomas. "Diagnosis and treatment of low back pain." Bmj 332.7555 (2006): 1430-1434.

[23] Guideline on the clinical development of medicinal products intended for the treatment of pain. EMA/CHMP/970057/2011, 15 Dez 2016.

patients with chronic non-specific low back pain. The prevalence of neuropathic components varies greatly from study to study: between 16% and 55%. Pain radiation is an important criterion, as neuropathic pain components increase the greater the distal radiation from the lower back.[24,25]

The treatment of chronic non-specific low back pain does not differ significantly from country to country, with the focus being on non-drug interventions such as promoting physical activity. If these do not result in any improvement, the guidelines indicate drug treatment.[26,27,28] In accordance with the German treatment guideline (NVL) for "non-specific low back pain", drug treatment is indicated if analgesic drug therapy is considered supportive for the realisation of activating measures, or if the patient has unbearable functional disabilities as a result of the pain, despite regularly performing these measures.[29] Patients initially receive non-opioid analgesics (NSAIDs such as ibuprofen, diclofenac or naproxen/metamizole), but they should be used for as short a time as possible and at the lowest effective dose due to their potential for considerable adverse drug reactions and interactions. For this reason, they are not suitable for long-term treatment. Due to the adverse drug reactions described for metamizole (leukopenia, hypotension, allergies, agranulocytosis), it is not authorised in many countries, such as Sweden, France, Greece, the United Kingdom, USA and Mexico.[30] Alternatively, serotonin–norepinephrine reuptake inhibitors (SSNRIs) such as duloxetine are recommended in the guidelines in some countries such as the United Kingdom.[31] For patients with insufficient response to non-opioid analgesics, the only available option for the longer-term drug treatment of chronic pain conditions is opioid analgesics. In accordance with national guidelines, however, opioids are only considered for long-term treatment if the patient experienced a clinically relevant reduction in pain and/or physical disability, with no or zero adverse reactions, after an initial, short-term treatment.

[24] Baron R et al.: Neuropathic low back pain in clinical practice. Eur J Pain, 2016; 20(6):861-873.

[25] Bonifer R.: Neuropathischer Rückenschmerz. Eur J Pain, 2017;

[26] Nationale Versorgungsleitlinie Nicht-spezifischer Kreuzschmerz, AWMF, 2. Auflage Version 01 2017

[27] ACP guideline: Noninvasive Treatments for Acute, Subacute, and Chronic Low Back Pain: A Clinical Practice Guideline From the American College of Physicians. Ann Intern Med. 2017;166:514-530. doi:10.7326/M16-2367

[28] NICE guideline: Low back pain and sciatica in over 16s: assessment and management (NG59). Published 30 November 2016. https://www.nice.org.uk/guidance/ng59

[29] Nationale Versorgungsleitlinie Nicht-spezifischer Kreuzschmerz, AWMF, 2. Auflage Version 01 2017

[30] Arzneimittelkommission der deutschen Ärzteschaft (AkdÄ), 2017. https://www.gelbe-liste.de/nachrichten/metamizol-agranulozytose

[31] NICE guideline: Low back pain and sciatica in over 16s: assessment and management (NG59). Published 30 November 2016. https://www.nice.org.uk/guidance/ng59

3.2 Cannabis-based medication for pain patients

The efficacy of THC and THC-rich cannabis products in chronic pain has already been studied in over 15 randomised controlled clinical studies with more than 1,600 patients. There are many reviews summarising and evaluating clinical evidence. In the period from 2015 to early 2019 alone, more than seven reviews were published with similar conclusions. Cannabis-based substances have been proven to be effective in comparison to placebo in terms of pain reduction for both neuropathic and nociceptive pain. A positive effect of cannabinoids was found even for individual criteria, such as sleep quality, quality of life or patient satisfaction.

In a cross-sectional study from 2017, data from patients suffering from pain who had taken opioids as monotherapy or opioids with cannabis over a period of six months was evaluated. 97% of patients who had taken both were able to reduce the amount of opioids and 81% stated that cannabis is more effective against pain than opioids. In addition, the adverse reactions of cannabis were more tolerable than those of opioids in 92% of patients.[32]

In addition to efficacy and considerably improved tolerability, cannabis products consumed orally exhibit a lower dependence and abuse potential in comparison to opioids. In two long-term studies with the drug Sativex (cannabis extract) and dronabinol (pure THC), there were no signs of consumption suggesting abuse, with the dose remaining constant over a period of up to four years.[33,34,35] The sudden discontinuation of long-term cannabis treatment also did not lead to withdrawal symptoms. Short-term sleep, emotional or appetite disturbances only occurred in isolated cases. Prescription data for synthetic THC, which has been available as an authorised medication on the American market for over 25 years, displays consistent use in the therapeutic dosage range without signs of 'doctor hopping', i.e.

[32] Reiman, Amanda, Mark Welty, and Perry Solomon.: Cannabis as a substitute for opioid-based pain medication: patient self-report. Cannabis and cannabinoid research 2.1 (2017): 160-166

[33] Lucas, Philippe.: Cannabis as an adjunct to or substitute for opiates in the treatment of chronic pain. Journal of psychoactive drugs 44.2 (2012): 125-133.

[34] Schoedel, Kerri Alexandra, et al.: A randomized, double-blind, placebo-controlled, crossover study to evaluate the subjective abuse potential and cognitive effects of nabiximols oromucosal spray in subjects with a history of recreational cannabis use. Human Psychopharmacology: Clinical and Experimental 26.3 (2011): 224-236.

[35] Schimrigk, Sebastian, et al.: Dronabinol is a safe long-term treatment option for neuropathic pain patients. European neurology 78.5-6 (2017): 320-329.

repeatedly changing doctors for renewed prescriptions. American addiction specialists have also not observed any abuse of dronabinol among patients.[36,37,38,39,40,41]

A detailed overview of findings from pre-clinical studies that have potential clinical significance and from other clinical studies is presented in tabular form in Appendix 2.

3.3 Rationale for clinical trial VER-CLBP-001

For patients with chronic non-specific low back pain, the only current long-term drug treatment available are opioid analgesics, which can present a significant problem in everyday medical practice. Specifically, the long-term use of opioids is associated with considerable adverse reactions. The most common adverse reactions include constipation (25-64%), nausea (20-35%), vomiting (10-20%), exhaustion, dry mouth, dizziness, increased sweating, impaired cognitive performance, itchiness, mood changes, headaches, and loss of sexual desire.[42,43,44,45] In clinical practice, opioid-induced constipation is described as the most common adverse reaction (occurrence of up to 90%), which not only impacts patients' quality of life and results in stopping treatment, but can also cause severe complications.[46] In

[36] Calhoun, Sarah R., Gantt P. Galloway, and David E. Smith. "Abuse potential of dronabinol (Marinol®)." Journal of psychoactive drugs 30.2 (1998): 187-196.

[37] Rog, David J., et al.: Randomized, controlled trial of cannabis-based medicine in central pain in multiple sclerosis. Neurology 65.6 (2005): 812-819.

[38] Nurmikko TJ, Serpell MG, Hoggart B, et al.: Sativex successfully treats neuropathic pain characterised by allodynia: A randomised, double-blind, placebo-controlled clinical trial. Pain 2007;133(1– 3):210–20

[39] Johnson, Jeremy R., et al.: Multicenter, double-blind, randomized, placebo-controlled, parallel-group study of the efficacy, safety, and tolerability of THC: CBD extract and THC extract in patients with intractable cancer-related pain. Journal of pain and symptom management 39.2 (2010): 167-179.

[40] Blake, David R., et al.: Preliminary assessment of the efficacy, tolerability and safety of a cannabis-based medicine (Sativex) in the treatment of pain caused by rheumatoid arthritis. Rheumatology 45.1 (2006): 50-52.

[41] Haroutounian, Simon, et al.: The effect of medicinal cannabis on pain and quality-of-life outcomes in chronic pain. The Clinical journal of pain 32.12 (2016): 1036-1043.

[42] Buynak, Robert, et al. "Efficacy and safety of tapentadol extended release for the management of chronic low back pain: results of a prospective, randomized, double-blind, placebo-and active-controlled Phase III study." Expert opinion on pharmacotherapy 11.11 (2010): 1787-1804.

[43] Baron, Ralf, et al. "Effectiveness of tapentadol prolonged release (PR) compared with oxycodone/naloxone PR for the management of severe chronic low back pain with a neuropathic component: a randomized, controlled, open-label, phase 3b/4 study." Pain Practice 16.5 (2016): 580-599.

[44] Khoromi, Suzan, et al. "Morphine, nortriptyline and their combination vs. placebo in patients with chronic lumbar root pain." Pain 130.1-2 (2007): 66-75.

[45] Steigerwald, Ilona, et al. "Effectiveness and safety of tapentadol prolonged release for severe, chronic low back pain with or without a neuropathic pain component: results of an open-label, phase 3b study." Current medical research and opinion 28.6 (2012): 911-936.

[46] https://www.medmedia.at/univ-innere-medizin/opioidinduzierte-obstipation/, zuletzt aufgerufen am 18.12.2019

addition, the high abuse potential and the risk of physical and psychological dependence limit the long-term administration of opioids.

Cannabis-based medications, however, have been proven to be better tolerated than opioids during long-term treatment for both neuropathic and nociceptive pain.[47,48,49,50,51] Yet, there is currently no cannabis-based or cannabinoid prescription drug available for the treatment of chronic non-specific low back pain. To date, cannabis-based drugs have been prescribed for pain on an off-label basis (Sativex® and Canemes®) or as a magistral medicinal preparation for dispensation to individual patients (Germany and Austria). Furthermore, in Germany cannabis treatments are available in the form of cannabis buds.

To be able to offer patients suffering from pain a more tolerable and authorised alternative to opioids, Vertanical GmbH is currently developing a prescription drug based on a cannabis extract standardised to 5% delta-9-tetrahydrocannabinol (THC). The clinical study described here should form the basis for the application for marketing authorisation of this medicinal product. The IMP VER-01 should be used in the following patient group:

Patients with chronic non-specific low back pain when drug treatment is indicated and a previous optimised treatment with non-opioids has not led to sufficient pain relief or was unsuitable due to contraindications or intolerance.

The aim of this clinical phase 3 study is to demonstrate the efficacy, maintained efficacy and long-term safety of VER-01, as well as to investigate the dependence and abuse potential as well as the effects of sudden drug withdrawal.

[47] Nurmikko TJ, Serpell MG, Hoggart B, et al. Sativex successfully treats neuropathic pain characterised by allodynia: A randomised, double-blind, placebo-controlled clinical trial. Pain 2007;133(1– 3):210–20

[48] Rog, David J., et al. "Randomized, controlled trial of cannabis-based medicine in central pain in multiple sclerosis." Neurology 65.6 (2005): 812-819.

[49] Johnson, Jeremy R., et al. "Multicenter, double-blind, randomized, placebo-controlled, parallel-group study of the efficacy, safety, and tolerability of THC: CBD extract and THC extract in patients with intractable cancer-related pain." Journal of pain and symptom management 39.2 (2010): 167-179.

[50] Blake, David R., et al. "Preliminary assessment of the efficacy, tolerability and safety of a cannabis-based medicine (Sativex) in the treatment of pain caused by rheumatoid arthritis." Rheumatology 45.1 (2006): 50-52.

[51] Haroutounian, Simon, et al. "The effect of medicinal cannabis on pain and quality-of-life outcomes in chronic pain." The Clinical journal of pain 32.12 (2016): 1036-1043.

4. Study design

4.1 Study design – overview

The multicentre phase III study is divided into four phases: Phase A, B, C and D. Phases A and D follow a double-blind, placebo-controlled design, while Phase B and C have an open-label design.

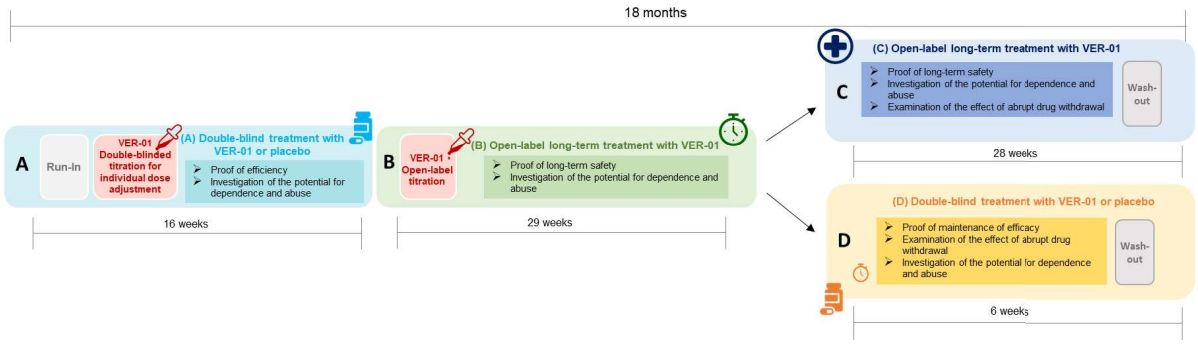

Figure 1: Overview of the duration and purpose of the individual phases of the study.

Phase A: Randomised, double-blind, placebo-controlled treatment over three months to demonstrate the efficacy of VER-01. After screening and a one-week run-in phase to determine baseline pain intensity in the morning, patients are randomized to a three-week double-blind placebo-controlled titration with VER-01 or placebo for individual dose finding (R1). Subsequently, patients start the 12-week double-blind, placebo-controlled treatment phase. In Phase A, patients attend six visits (see Chapter 1.3 and Figure 1 for details).

Phase B: Long-term, open-label treatment with VER-01 (26 weeks) to demonstrate long-term safety and investigate the potential for dependency and abuse. Patients who have completed Phase A and did not violate any of the inclusion or exclusion criteria for Phase B shall begin Phase B. After three weeks of open-label titration with VER-01 for dose-finding, the patients shall take VER-01 at their self-selected dose over a period of 26 weeks. In Phase B, four regular visits (B7, B8, B9, B10) and four visits for the withdrawal and dispensing of the IMP (AID1, AID2, AID3, AID4) take place. Should the planned number of patients in Phase B be reached early, further patients who then conclude Phase A will end the study as normal at Visit A6.

Phases C and D run in parallel, so that patients who have completed Phase B and did not violate any of the inclusion or exclusion criteria for Phase C and/or D can be assigned to one of these two phases.

The final assignment to one of the two phases is done using a statistical approach, so that at least 80 (target: 120) responders are initially included in Phase D. Should the planned number of patients in

Phase C and D be reached early, further patients ending Phase B thereafter will no longer necessarily be assigned to either of the two phases, but will end the study as normal after Phase B.

Phase C: Long-term, open-label treatment (additional 26 weeks) to demonstrate long-term safety and to investigate the potential for dependency and abuse as well as the effect of sudden drug withdrawal. Patients are assigned to this phase regardless of the level of pain reduction. They take VER-01 for a further 26 weeks, then stop taking it abruptly and undergo a two-week wash-out phase. A total of four regular visits (C11, C12, C13, C14) and four visits for withdrawal and dispensing of the IMP (AID5, AID6, AID7, AID8) take place in this phase.

Phase D: Randomised, double-blind, placebo-controlled treatment (4 weeks) to demonstrate maintained efficacy and investigate the potential as well as the effects of sudden drug withdrawal. To be included in Phase D, patients must have experienced pain reduction of at least 30% in the morning during treatment Phase B ("responders") and took the investigational medicinal product on at least 5 out of 7 days (daily dose at least 1 n) and documented use. In addition, only patients who have not used any non-drug therapy (e.g., physical or behavioural therapy, acupuncture, massage, thermotherapy) that significantly modulates pain sensation in the last nine weeks prior to Visit B10 or who have used a non-drug therapy unchanged for at least nine weeks prior to Visit B10 can be included in Phase D. Furthermore, the use of additional analgesic medication (non-opioid and opioid analgesics as well as adjuvant analgesics, except rescue medication) within 21 days prior to Visit B10 is an exclusion criterion for Phase D, so as not to confound the assessment of maintenance of efficacy of VER-01. After randomisation at the beginning of the phase, patients receive four weeks of treatment with VER-01 or placebo. Patients who receive placebo in this phase will undergo sudden, blinded drug withdrawal. This is followed by a two-week wash-out phase for follow-up. Overall, two visits take place in this phase.

4.2 Discussion of the study design

Randomised, placebo-controlled, double-blind studies are regarded as the gold standard for clinical trials in general. Blinding prevents bias about the efficacy of the IMP or placebo and therefore the study results are not influenced by the doctor's treatment of the patient or the patient's expectations about the IMP.[52] When developing a new medication for the treatment of pain, it is necessary in accordance with the new EMA guideline to demonstrate the efficacy and safety in comparison to placebo, as well as to investigate long-term safety and maintained efficacy. In addition, studies should be

[52] Reflection paper on the need for active control in therapeutic areas where use of placebo is deemed ethical and one or more established medicines are available, EMA/759784/2010, November 2010

planned to investigate possible signs of withdrawal and the potential for dependence and abuse.[53] Hence, a multi-phase, randomised, placebo-controlled study design was chosen to meet these requirements.

After a one-week run-in phase to record the baseline pain of the participating subjects, eligible patients will be double-blind randomised to a gradual, placebo-controlled titration up to the optimal patient-specific dose. The gradual increase in the daily dose makes it possible to minimise potential adverse reactions at excessively high ad-hoc doses of the IMP and takes the varying responses to the IMP of different patients into account.

The analgesic effect of VER 01 is being investigated in Phase A as monotherapy compared to placebo. After the three-week placebo-controlled titration phase, patients will receive verum or placebo for a period of 12 weeks. For this purpose, all pain-influencing concomitant therapies must have been discontinued for a sufficiently long time before taking the IMP for the first time, or they must have been administered unchanged in order not to influence the evaluation. The only exception to this is the rescue medication. The exact details of the exclusion criteria with regard to drug and non-drug concomitant therapies are explained in more detail in Chapters 5.3 and 5.4. This design meets the requirement of the EMA to demonstrate a sustained therapeutic effect in chronic pain during a treatment period of at least 12 weeks (without titration period) in efficacy studies relevant to approval.[54]

The ICH E1A guideline requires a treatment duration of at least twelve months with the IMP to investigate long-term safety and the potential for dependency and abuse. In addition, further treatment of a subpopulation is recommended in order to rule out adverse events that could only arise after being administered for a certain amount of time.[55] In line with this guideline, study Phase B involves 26 weeks of open-label treatment with VER-01 after a three-week open-label titration phase and a further 26 weeks with a subpopulation in Phase C (52 weeks overall). Re-titration at the beginning of Phase B is considered necessary since patients receiving a placebo during Phase A will need to re-titrate their dose slowly to minimise the risk of side effects. An alternative unblinding of patients at the end of Phase A would potentially distort the study effects and is consequently not possible. It is therefore inevitable that patients who received VER-01 in Phase A will also need to re-titrate their dose. During participation in study Phases B and C, adjustments to the non-drug concomitant therapies may be

[53] Guideline on the clinical development of medicinal products intended for the treatment of pain, EMA/CHMP/970057/2011, 15 December 2016

[54] Guideline on the clinical development of medicinal products intended for the treatment of pain, EMA/CHMP/970057/2011, 15 December 2016

[55] ICH E1 The Extent of Population Exposure to Assess Clinical Safety for Drugs Intended for Long-Term Treatment of Non-Life Threatening Conditions, Current Step 4 Version dated 27 October 1994

made, as the primary objective of these study phases is not to investigate efficacy or maintenance of efficacy. In addition, due to the long duration of the study phases and in the interest of patient well-being, patients should be given the opportunity to continue, restart or terminate non-drug therapies (e.g., physical therapy, massage). In addition, drug therapies may be started in study Phases B and C, the primary goal of which is to prove long-term safety, but the patient should keep the dosage as stable as possible.

The sudden withdrawal of the medication at the end of Phase C and the two-week wash-out phase serves to investigate possible signs of withdrawal. This design fulfils the requirements for demonstrating long-term safety and investigating the potential for dependency and abuse.

Evidence of maintained efficacy and the investigation of the potential for dependence or abuse of VER-01 in a randomised withdrawal study takes place in Phase D. Patients who successfully responded to treatment in Phase B and have not used additional treatments as specified are again randomised into the treatment (VER-01) or placebo group and maintained efficacy is investigated in the blinded, placebo-controlled phase. At the same time, this phase serves for the placebo-controlled determination of the dependence and abuse potential of VER-01. This design is also required by the EMA for the investigation of maintained efficacy.[56] In order to meet the requirements of the regulatory authorities and scientifically evaluate the maintenance of efficacy and possible withdrawal symptoms after long-term treatment with VER-01, the planned procedure is considered essential. During Phase D, patients record possible withdrawal symptoms in their diaries on a daily basis so that reliable data can be collected and evaluated to answer the question. In addition, only mild and short-term withdrawal symptoms are expected.

An overview of EMA requirements and their implementation in the various study phases is presented in Table 4.

Table 4: Requirements from the EMA guideline and implementation into the clinical trial VER-CLBP-001

**Detection required in accordance with EMA or targeted testing for:**	**Planned tests in the study VER-CLBP-001**			
	**Phase A** (3 months, double-blind, randomised, placebo-controlled)	**Phase B** (26 weeks, open-label active treatment)	**Phase C** (26 weeks, open-label active treatment)	**Phase D** (1 month, double-blind, randomised, placebo-controlled)
Efficacy	X			
Maintained efficacy				X
Safety	X	X	X	X

[56] Guideline on the clinical development of medicinal products intended for the treatment of pain, EMA/CHMP/970057/2011, 15 December 2016

Long-term safety		X	X	
Withdrawal symptoms			X	X
Potential for dependency and abuse	X	X	X	X

The selected sample size, efficacy and safety parameters were evaluated statistically (see Chapter 9).

4.3 Primary objective

4.3.1 Phase A

The primary objective of Phase A of the described study is to demonstrate the efficacy of VER-01 compared to placebo in the treatment of patients with chronic non-specific low back when drug treatment is indicated and a previous optimised treatment with non-opioids has not led to sufficient pain relief or was unsuitable due to contraindications or intolerance. The primary endpoint is defined as the change in average pain intensity in the morning compared to baseline on an 11-point NRS (mean value of study Week 15 compared to the mean value of the seven days prior to Visit A2 (study Week -1) with daily documentation of pain intensity in the morning). The mean change in pain intensity in the morning versus baseline is compared between VER-01 and placebo treatment. The aim is to demonstrate statistically significant superiority for the VER-01 treatment arm.

4.3.2 Phase B

In long-term, open-label treatment with VER-01 over 26 weeks, long-term safety, investigation of the potential for dependency and abuse will be investigated. They are determined by the investigator at the visits based on the following points:

- Occurrence of treatment related adverse events (AEs)/serious adverse events (SAE)s (Visit B7-B10, AID1-AID4)
- Determining a complete blood count and clinical chemistry parameters using a full-blood analysis (Visit B7 and B10)
- Vital signs (blood pressure, pulse, body temperature, body weight) (Visit B7-B10)
- Physical examination (lungs, heart, skin, lymph nodes, CNS) (Visit B10)
- Study dropout (Visit B7-B10, AID1-AID4)
- Assessment of substance dependence: Addiction Behaviours Checklist (ABC) (Visit B7-B10)

4.3.3 Phase C

In long-term, open-label treatment with VER-01, long-term safety is investigated over additional 26 weeks, as is the potential for dependency and abuse and the effects of sudden drug withdrawal. They are determined by the investigator at the visits based on the following points:

- Occurrence of treatment-related AEs/SAEs (Visit C11-C13, AID5-AID8)
- Analysis of complete blood count and clinical chemistry parameters using a full-blood sample (Visit C13)
- Vital signs (blood pressure, pulse, body temperature, body weight) (Visit C11-C14)
- Physical examination (lungs, heart, skin, lymph nodes, CNS) (Visit C13 and C14)
- Study dropout (Visit C11-C14, AID5-AID8)
- Assessment of substance dependence: Addiction Behaviours Checklist (ABC) (Visit C11-C13)
- Diagnosis of possible dependency syndrome (F12.2 according to ICD-10) (Visit C13)

4.3.4 Phase D

To be able to demonstrate the maintained efficacy of VER-01 on a placebo-controlled basis, patients with an improved pain score of at least 30% in the morning at the end of Phase B (average of study Week 43) in comparison to the start of Phase A (average of the seven days prior to Visit A2 (study Week -1)) are again randomised to treatment with VER-01 or placebo. The primary endpoint of Phase D is time to treatment failure, which is the time in days from randomisation to Phase D (R2) until the first day of treatment failure.

Treatment failure is assessed by the daily calculated seven-day mean value of the NRS pain score in the morning during the treatment period, which must have deteriorated by at least 20% and at least 1 point compared to baseline (mean value of study Week 43). The first day within this seven-day window for which this criterion is fulfilled is subsequently defined as the first day of treatment failure.

Furthermore, treatment failure is defined as an early discontinuation of treatment for selected reasons (see Chapter 9).

In addition, the potential for dependency and abuse as well as the effects of sudden drug withdrawal are investigated. They are determined by the investigator at the visits based on the following points:

- Recording of the number of days from second randomisation (R2 or Visit B10) until study drop-out before the end of the four-week treatment Phase D (Visit D11)
- Assessment of substance dependence: Addiction Behaviours Checklist (ABC) (Visit D11)
- Diagnosis of possible dependency syndrome (F12.2 according to ICD-10) (Visit D11)

The following points are documented by the patient:

- average pain intensity in the past 12 hours on an 11-point NRS (daily in the morning)

4.4 Secondary and additional objectives

4.4.1 Key-secondary endpoint of Phase A

According to the EMA guideline, the efficacy of VER-01 must be demonstrated for both the neuropathic and nociceptive pain components for the claimed indication. The determination of the influence of the treatment on the pain intensity of the neuropathic pain component is therefore defined as a key-secondary study objective.

Neuropathic pain is assessed at each visit during Phase A using the NPSI (Neuropathic Pain Symptom Inventory) questionnaire, which has been specifically designed and validated to assess different symptoms of neuropathic pain.[57] The key-secondary endpoint is the change in the overall NPSI score at Visit A6 compared to baseline (Visit A2). The NPSI is collected at baseline and after 15 weeks of treatment with VER-01 or placebo and the corresponding overall score is calculated. The mean change in the overall NPSI score from baseline is compared between VER-01 and placebo treatment for patients with a painDETECT score >18. The aim is to demonstrate the superiority of treatment with VER-01 over placebo treatment.

4.4.2 Further secondary objectives

The further secondary objectives across the phases are described in detail in Chapter 7.15.1.2.

[57] Bouhassira et al. "Development and validation of the neuropathic pain symptom inventory." Pain 108.3 (2004): 248-257.

5. Patients and study sites

5.1 Selection of study population and recruitment

The population being investigated consists of patients who meet the inclusion criteria as described in Chapter 5.3.

Patients are primarily made aware of the clinical trial via notices in doctors' offices and adverts in magazines and subway trains as well as online advertising. Interested individuals are subsequently screened to ensure they meet the inclusion and exclusion criteria. Eligible patients are then referred to appropriate study sites. In addition, investigators participating in the study recruit patients from their databases. See Chapter 7.4.1 for the specifics of screening, the consent process, and briefing.

5.2 Number of patients

A total of 808 patients will be initially randomised to Phase A. Over the course of the study, the number of patients will decline due to drop-outs throughout the different study phases (see Chapter 7.4.2 and 1). The anticipated reduction of patient numbers throughout the phases can be seen in Figure 2.

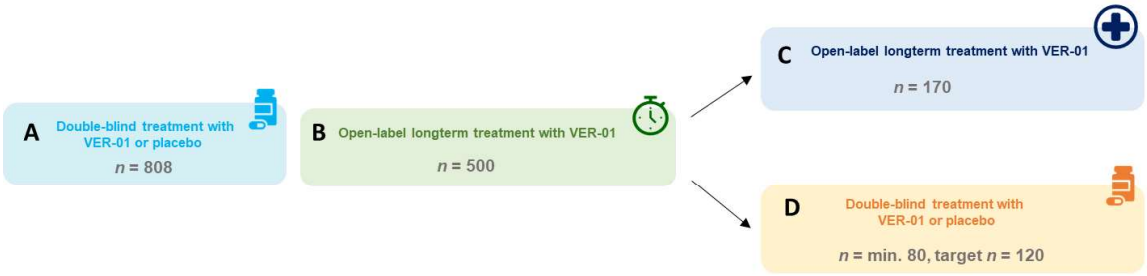

Figure 2: Number of patients (n) during the individual study phases.

5.3 Inclusion criteria

5.3.1 For the entire study (Phase A-D)

1. Male and female patients (18 years and older)
2. Chronic (for at least three months) non-specific pain in the lower back (between the lower ribcage and the gluteal folds)
3. Pain intensity on average at least 4 points on an 11-point NRS (one month before the start of the study)
4. Patients with indicated drug treatment* where previous optimised treatments** with non-opioid analgesics have not led to sufficient pain relief or were unsuitable due to contraindications or intolerance.

** Drug treatment is indicated if analgesic drug therapy is considered supportive for the realisation of activating measures, or if the patient has unbearable functional disabilities as a result of the pain, despite regularly performing these measures.*

*** Treatment is considered optimised when*

I. a further increased drug dose is unsuitable from a medical perspective considering side effects and/or

II. it is not expected that a higher drug dose would result in a further advantage in terms of efficacy.

5. Willingness of both men and women to use a reliable method of contraception during study participation and for three months after taking the last dose of the IMP
6. Signed patient information and informed consent form is available
7. Understanding of the local language, compliance and ability to give consent
8. The patient has understood the instructions to avoid changes in lifestyle and dietary habits
9. The patient has understood the principle of the patient diary and gives their consent to keep it as instructed

5.3.2 Additional inclusion criteria for Phase A

a1. Average score of pain intensity in the morning in the 7 days before Visit A2 (study Week -1) must be at least 4 points on an 11-point NRS. For the calculation, the last 7 pain intensity entries before Visit A2 are taken from the patient diary (there must be at least 5 pain intensity readings in the morning from study Week -1)

a2. Willingness not to take any additional analgesic medication (non-opioid and opioid analgesics as well as adjuvant analgesics) during participation in study Phase A (except rescue medication)

Non-opioid and adjuvant analgesics are listed in.Table 5.

a3. Willingness to continue a current non-drug therapy unchanged as planned during participationin Phase A.

Examples of non-drug therapies are listed in Table 6.

5.3.3 Additional inclusion criteria for Phase B

b1. Previous and complete participation in Phase A until and including Visit A6

b2. Patient wishes to participate voluntarily in the long-term study

b3. From the investigator's point of view, further participation is considered medically safe

b4. Willingness not to take any additional analgesic medication (non-opioid and opioid analgesics as well as adjuvant analgesics) during the last three weeks of study Phase B (except rescue medication).

Non-opioid and adjuvant analgesics are listed in Table 5.

5.3.4 Additional inclusion criteria for Phase C

c1. Previous and complete participation in Phase B until and including Visit B10

c2. Patient wishes to participate voluntarily in the long-term study

c3. From the investigator's point of view, further participation is considered medically safe

5.3.5 Additional inclusion criteria for Phase D

d1. Previous and complete participation in Phase B until and including Visit B10

d2. Patient has experienced a pain score improvement of at least 30% in treatment Phase B (mean value of pain score in the morning in study Week 43 compared to the mean value of the seven days prior to Visit A2 (study Week -1), there must be at least four values from study Week 43 and five values from study Week -1)

d3. Patient wishes to participate voluntarily in the study

d4. From the investigator's point of view, further participation is considered medically safe

d5. Willingness not to take any analgesic medication (non-opioid and opioid analgesics as well as adjuvant analgesics) during participation in study Phase D (except rescue medication) Non-opioid and adjuvant analgesics are listed in Table 5.

d6. Willingness to continue a current non-drug therapy unchanged as planned during study participation in Phase D.

Examples of non-drug therapies are listed in Table 6.

d7. The patient took the investigational medicinal product on at least 5 out of 7 days in study week 43 (daily dose min. 1 n) and documented taking the investigational medicinal product in the patient diary.

5.4 Exclusion criteria

1. Professional groups for which the ability to operate machinery and drive vehicles is the primary activity (including truck, bus and forklift drivers, pilots)
2. Alcohol/drug/medication abuse and previous or current intake of methadone in the patients' medical history or suspected by the investigator
3. Intake of analgesic medication (non-opioid and opioid analgesics as well as adjuvant analgesics) within seven days prior to the start of the study
   Non-opioid and adjuvant analgesics are listed in Table 5.
4. Taking cannabis-based medications within 30 days prior to the start of the study
5. HIV, dementia (which impairs the assessment of symptoms)
6. Severe forms of the following diseases: Anaemia, hematological/autoimmune/endocrinal/renal/hepatic/respiratory/cardiovascular or gastrointestinal diseases, symptomatic peripheral vascular diseases
7. Cardiovascular event in the past three months, poorly managed high blood pressure, untreated hypothyroidism, patients with Crigler-Najjar syndrome or Rotor syndrome, surgery within the past two months
8. Severe mental illnesses (e.g., psychosis, schizophrenia, bipolar disorder) currently or in the past, severe depression currently or in the past not due to chronic low back pain, or individuals at risk of suicide (examined using the MINI questionnaire: as soon as at least one module is complete)
9. Severe mental illness (psychosis, schizophrenia, bipolar disorder, severe depression, anxiety disorder) currently or in the past in a first-degree relative (parents and children); suicide in a first-degree relative (parents and children)
10. Patients with an active cancer or tumour-related pain or strong pain due to physical injuries
11. Other painful comorbidities, excluding low back pain, that could interfere with the patient's evaluation during the study or the assessment of pain

12. Well-known strong adverse events in connection with cannabis consumption before the start of the study
13. Known allergy to cannabis and/or sesame seeds and products derived from them
14. Known hypersensitivity to the ingredients of the rescue medication
15. Planned blood donation, planned sperm or egg donation, planned freezing of eggs or sperm
16. Pregnancy, breastfeeding, desire to have children (within the next 20 months)
17. Participation in another interventional clinical trial within the last 30 days before the start of the study
18. Inability to give consent, care dependency, patient has a legal guardian/caregiver, or is immobile
19. The patient is in need of special protection (e.g., incarcerated; institutionalized by a court or judicial authority; in a dependent or employment relationship with the sponsor, an external service provider of the sponsor (who is involved in the study conduct), the investigator, or the study site).

Table 1: Examples of prohibited concomitant medication during phase A and D and in the last 21 days of phase B: non-opioid and adjuvant analgesics acc. to drug class with examples

Non-opioid analgesics	
Drug class	Examples of drugs in the drug class
Acidic (non-opioid) analgesics / non-steroidal anti-inflammatory drugs (NSAID)	ASA (> 100 mg), ibuprofen, naproxen, diclofenac, piroxicam, indomethacin, phenylbutazone
Non-acidic (non-opioid) analgesics	Metamizole, paracetamol, phenazone, celecoxib, parecoxib
Centrally-acting non-opioid analgesics	Flupirtine
Topically-applied substances	Diclofenac, ibuprofen
Adjuvant analgesics	
Drug class	Examples of drugs in the drug class
Anticonvulsants	Pregabalin, gabapentin, carbamazepine
Tri-cyclic antidepressants (TCA)	Amitriptyline, imipramine
Serotonin norepinephrine reuptake inhibitors (SNRI)	Duloxetine, milnacipran, venlafaxine
Selective serotonin reuptake inhibitors (SSRI)	Fluoxetine, fluvoxamine, citalopram, escitalopram, sertraline
Monoamine oxidase inhibitors	Tranylcypromine, moclobemide
Other antidepressants	Mirtazapine, mianserin, bupropion, reboxetine
Topically-applied substances	Capsaicin, lidocaine

Opioids and THC-based medicines are also prohibited concomitant medication.

Table 6: Examples of non-drug therapies that significantly modulate pain perception

Non-drug therapies that significantly modulate pain perception
Physiotherapy or behavioural therapy
Relaxation techniques such as progressive muscle relaxation
Rehabilitation sport and functional training
Acupuncture
Ergotherapy
Manual therapy such as manipulation/mobilisation
Massage
Back exercise training
Thermotherapy

5.4.1 Additional exclusion criteria for Phase A

a1. In the case of a current non-drug therapy (e.g., physical or behavioural therapy, acupuncture, massage, thermotherapy), which significantly modulates the perception of pain, it was not maintained unchanged for at least eight weeks prior to study participation in Phase A.

Examples of non-drug therapies are listed in Table 6.

5.4.2 Additional exclusion criteria for Phase D

d1. Intake of additional analgesics (non-opioid and opioid analgesics) within 21 days prior to the start of study Phase D (except rescue medication)

d2. Non-opioid and adjuvant analgesics are listed in Table 5.

d3. In the case of a current non-drug therapy (e.g., physical or behavioural therapy, acupuncture, massage, thermotherapy), that significantly modulates the perception of pain, it was not maintained unchanged for at least nine weeks prior to the start of study Phase D.

d4. Examples of non-drug therapies are listed in Table 6.

5.5 Screen failures

All patients who have given their consent to participate, but were not randomised subsequently, are considered screen failures. A minimum amount of information is required to ensure transparent reporting of screen-failure study participants, to meet the requirements for the publication of the Consolidated Standards of Reporting Trials (CONSORT) and to respond to inquiries from regulatory authorities.

The minimum information required includes demographics, reasons for discontinuation and non-treatment-related serious adverse events, if these occur.

In the event of a screen failure, re-screening can take place provided that none of the reasons for drop-out apply:

- Violation of inclusion criterion no. 3
- Violation of inclusion criterion no. 5
- Violation of inclusion criterion no. 7
- Violation of inclusion criterion no. 8
- Violation of inclusion criterion no. 9
- Violation of inclusion criterion no. a1 (with the exception of cases in which technical difficulties with the eDiary made it impossible for the patient to make at least five pain score entries in the morning. In this case, it must be clear in the patient files that the reason for the entries not being made is not patient non-compliance. Re-screening cannot take place in the event of patient non-compliance)
- Violation of exclusion criterion no. 2
- Violation of exclusion criterion no. 5
- Violation of exclusion criterion no. 8
- Violation of exclusion criterion no. 9
- Violation of exclusion criterion no. 12
- Violation of exclusion criterion no. 13
- Violation of exclusion criterion no. 14

6. Investigational medicinal product

Two IMPs are used in the clinical trial: The IMP VER-01, a liquid full cannabis extract standardised to 2.1% delta 9-tetrahydrocannabinol, and a comparator free of the active-ingredient as placebo.

6.1 Description and name of the investigational medicinal product VER-01

Designation:	VER-01 (adjusted to 2.1% delta 9-tetrahydrocannabinol)
Pharmaceutical form:	Liquid preparation for oral intake
Primary packaging:	30 ml amber glass bottle including syringe with dose units scaled (“n”)
Active substance:	Cannabis extract standardised to 5% delta 9-tetrahydrocannabinol
Excipients:	Sesame oil
Dose unit:	1n (=2.5 mg THC per single dose)
Daily dose:	1n-13n (=2.5 -32.5 mg THC; patient-specific effective daily dose)
Use:	twice daily in the morning and evening (for a daily dose of 1n, this should be taken in the evening).
Taken in the evening:	Taken between 4pm and going to bed
Taken in the morning:	Taken between waking up and 12pm
Manufacturer:	Pharma Wernigerode GmbH, Paesel & Lorei GmbH & Co. KG (re-labelling)

6.2 Description and name of the placebo

Designation:	Placebo
Pharmaceutical form:	Liquid preparation for oral intake
Primary packaging:	30 ml amber glass bottle including syringe with dose units scaled (“n”)
Ingredients and excipients:	Sesame oil, terpenes, chlorophyll and carmine
Dose unit:	1n
Daily dose:	1-13n (patient-specific effective daily dose)
Use:	twice daily in the morning and evening (for a daily dose of 1n, this should be taken in the evening).
Taken in the evening:	Taken between 4pm and going to bed
Taken in the morning:	Taken between waking up and 12pm

Manufacturer: Pharma Wernigerode GmbH, Paesel & Lorei GmbH & Co. KG (re-labelling)

6.3 Production of the IMPs

VER-01 and placebo are produced in Germany by the contract manufacturer Pharma Wernigerode for Vertanical GmbH. The contract manufacturer possesses a manufacturing authorisation under German law (AMG) with many years of experience in the development and production of medications, especially in the form of liquid preparations for oral use. Furthermore, Pharma Wernigerode is specialised in the production of small batches of clinical IMPs. The manufacturer is GMP-certified, audited and possesses a permit to handle controlled drugs in accordance with Section 3 BtMG (German Narcotics Act).

6.4 Distribution of the IMPs

The IMPs are distributed by logistics companies. The IMPs are dispatched from the production site to national depots for storage and delivery to each study site. Delivery to the study sites is coordinated by the sponsor. The investigational medicinal products are released by the sponsor, after reviewing the investigational medicinal products' temperature logs

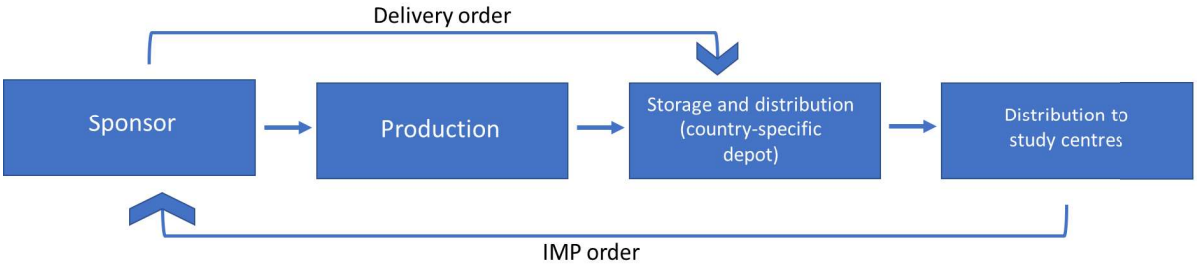

Figure 3: Distribution of the IMPs: The sponsor is contacted for the order and then forwards the order to the logistics company. They deliver the goods to the relevant study sites.

6.5 Labelling and packaging of the IMPs

The IMPs are labelled and packaged by the contract manufacturer, Pharma Wernigerode GmbH or, Paesel & Lorei GmbH & Co. KG (re-labelling). Labelling takes place in accordance with regulatory requirements, especially in consideration of specifications for primary and secondary packaging. In addition, the IMPs for study Phases A and D are encoded to ensure blinding.

6.5.1 Randomised labelling of the IMPs in Phases A and D

For the placebo-controlled Phases A and D, sufficient quantities of either IMP for one patient for the entire phase are packaged into one pack. Four of these identical packs are then packaged together into one box and sent to the study sites. The packs and IMPs are labelled with unique randomisation numbers with no possibility to distinguish between VER-01 and placebo. The number of amber glass bottles to be dispensed depending on the study phase and visit is shown in Table 7. The box always contains four associated emergency envelopes (including the randomisation numbers) to allow unblinding in a medical emergency (see Figure 4 and Chapter 7.14). These envelopes remain at the study site and are filed in the investigator site file so that the investigator can unblind in an emergency.

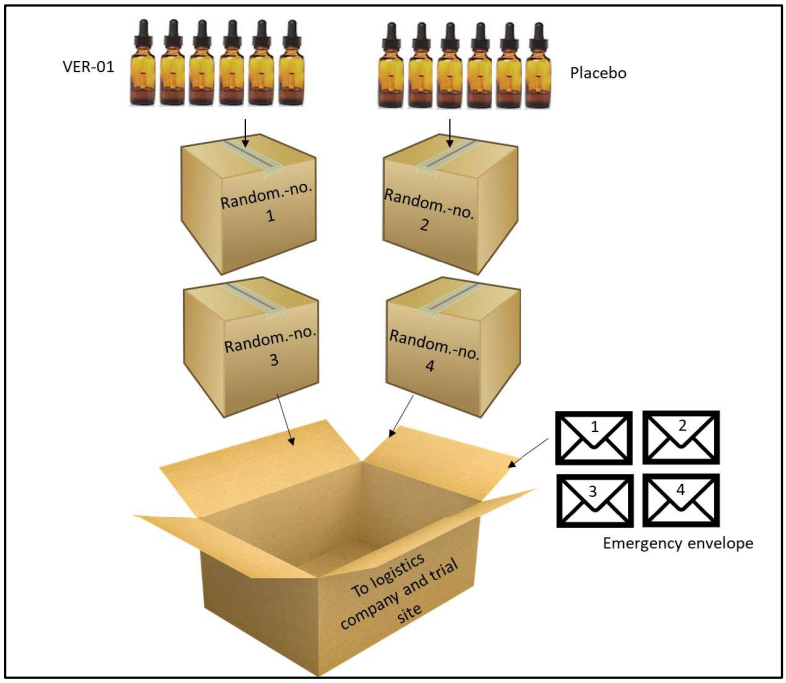

Figure 4: Packaging of the IMPs for the placebo-controlled Phases A and D. To ensure blinding, IMPs are packaged into individual packs and marked with randomisation numbers. Four packs are packaged into one box together with emergency envelopes. Note: number of bottles shown in the illustration is indicative, does not necessarily correspond to the actual number.

6.5.2 Open-label labelling in Phases B and C

During the open-label Phases B and C, the study site receives one delivery unit of 2 packs each containing 16 bottles of VER-01 per order. The necessary number of bottles to be dispensed at the relevant visits can be found in Table 7.

6.6 Storage of the IMPs at the study site

The IMPs must be stored at the study site in line with the specifications of national legislation for the storage of narcotics. Limited access must be guaranteed (e.g., lockable containers/rooms) and people with access permission must be known. Access by unauthorised individuals (especially children) must be prevented. The investigator is responsible for the proper storage of the IMPs

The IMPs must be stored at room temperature (15-25°C). Appropriate temperature conditions need to be monitored and documented every working day. The IMP is only allowed to be used within this clinical trial and should only be used until the specified expiration date ("use by: MM.YYYY").

6.7 Ordering and dispensing IMP at the study site

The IMP may only be dispensed to patients enrolled in the clinical trial by the individuals named in the responsibilities list (delegation log), which is filed in the ISF. Depending on the respective study phase and visit, a different number of bottles will be distributed (see Table 7).

Table 7: Number of glass bottles dispensed per study phase and visit

**Time of dispensing (visits)**	**A2**	**A3, A4, A5**	**A6**	**B7, AID1, B8, AID2, B9, AID3**	**AID4**	**B10**	**B10**	**AID5, C11, AID6, C12, AID7**	**AID8**
**# bottles**	2	2	2	2	1	2 (Phase D)	2 (Phase C)	2	1
**Study phase and duration**	Placebo or VER-01 titration (3 weeks)	Placebo or VER-01 (1 month)	Open-label VER-01 titration (3 weeks)	Open-label VER-01 (1 month)	Open-label VER-01 (1/2 month)	Placebo or VER-01 (1 month)	Open-label VER-01 (1 month)	Open-label VER-01 (1 month)	Open-label VER-01 (1/2 month)

6.7.1 Ordering and dispensing of blinded IMPs (Phase A and D)

To order blinded IMPs for Phases A and D, the study site must contact the sponsor.

At Visits A2 and B10, the IMPs for Phase A and D are dispensed to patients. For this purpose, the investigator calls the sponsor and receives a randomisation number randomly assigned to the corre-

sponding patient (see Chapter 7.13.1 and 7.13.2). Randomisation is stratified according to the painDETECTscore. The respective IMP with the assigned randomisation number is handed out by the doctor to the patient (see Figure 5).

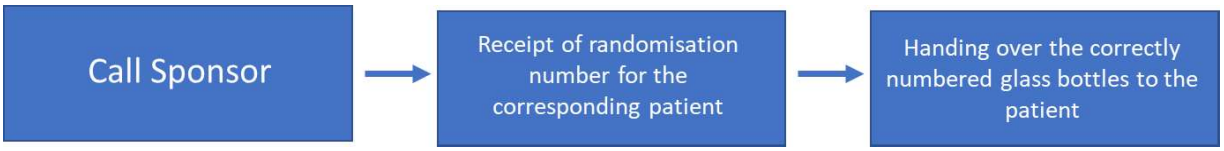

Figure 5: Dispensing of the blinded IMPs for Phases A and D (Visits A2 and B10) to patients based on randomisation procedure.

6.7.2 Ordering and dispensing of the IMPs (open-label Phase B and C)

The IMP is dispensed to the patients at defined visits in the open-label treatment phases. Visits in which the IMP is handed out and the respective number of bottles are specified in Table 7.

6.8 Return of the IMPs

Empty or open glass bottles must be returned by the patient at defined periods (see Table 8, visit schedule). The bottles are collected and documented by the study site. For correct documentation, each returned glass bottle (full, open or empty) is weighed individually by the investigator, the weight is noted in the form provided by the sponsor, which is filed in the patient file, and the bottles are stored in line with safety provisions until the sponsor arranges for them to be returned to the national depot. There, the IMPs are then properly destroyed. The documentation of the return and the weight of the amber glass bottles should follow the four-eyes principle, i.e. that each process should be checked by a second person.

If the immediate return of all IMPs is necessary (e.g., on grounds of safety, termination of the study etc.), the following process shall apply:

- the investigator informs all patients treated at their study site
- all patients return all unused and used IMPs to the investigator
- the investigator documents the date and the completeness of the returned IMPs in the form provided by the sponsor, which is filed in the patient file

If it is necessary for safety reasons, unblinding will take place as described under Chapter 7.14.

Table 8: Investigator visits (regular and AID visits) at which full, opened and/or empty amber glass bottles are returned. After being documented, these are stored until the planned pick-up.

	A3	A4	A5	A6	B7	AID1	B8	AID2	B9

Returning the study products (visits)	AID3	AID4	B10	AID5	C11	AID6	C12	AID7	AID8
	C13	D11							

6.9 Loss of the IMPs

In the event of a loss of the IMP by the patient (e.g., due to breaking a glass bottle, leaking content), a single glass bottle can be replaced once each during placebo-controlled treatment Phases A and D and the open-label treatment Phases B and C. To avoid misuse, it is not possible to dispense further quantities of IMP in the event of repeated loss.

6.10 Titration and patient-specific posology

Due to the very low toxicity and the broad therapeutic range as well as the high inter- and intraindividual variance with regards to pharmacokinetics, tolerability and efficacy, the optimal dose is determined within the course of a titration phase at the beginning of administration.

In general, the approach “start low, increase slowly and stay low” has become established for cannabis-based preparations in both clinical practice and clinical studies. This means that the patient slowly increases the dose in small steps (2.5 – 5 mg THC) on their own until they experience sufficient symptom relief, unacceptable side effects occur, or the maximum dose (32.5 mg THC per day and 17.5 mg THC per single dose in the titration phase or 20 mg THC per single dose in the treatment phase, respectively) is reached. If unacceptable side effects occur, the patient may reduce the dose and if the lower dose is tolerated well without sufficient symptom relief, they may try again to increase the dose since tolerance to adverse events develops over time. The maximum daily dose was limited to 32.5 mg THC and to two single doses, with a single dose maximum of 20 mg THC in the treatment phase. The maximum dose was chosen based on the expected therapeutically effective range as well as taking into account potential safety concerns. In clinical studies as well as in clinical practice, the mean therapeutically effective dose for most patients has been shown to be in the range of 18 to 26 mg THC. Intake is usually divided into two daily doses for oral preparations due to the pharmacokinetic profile. Increasing the dose to over 32.5 mg THC is rarely observed. In addition, a maximum dose of 32.5 mg THC and a single dose of 20 mg THC are considered safe, as no serious safety concerns have been identified for comparable THC-containing products even at supratherapeutic doses in the range of up to 90 mg THC.

The aspects mentioned are considered in the titration scheme of VER-01. Patients therefore start by taking a low initial daily dose of 2.5 mg THC in the evening, which normally does not trigger any subjectively perceived psychotropic effects and is well tolerated. Depending on the symptoms and pain

alleviation, the dose can be increased every three days until a sufficient therapeutic effect or negative adverse reactions occur. The exact approach can be found in the titration schedule (see Figure 6).

Since all patients who completed Phase A (regardless of whether they received placebo or verum) can be included in the open-label Phase B, the dose of VER-01 is titrated again at the start of Phase B. This ensures that patients who received placebo in Phase A can correctly re-titrate the optimal dose of the IMP over three weeks without unblinding Phase A.

For administering the IMP, a syringe is enclosed with the glass bottle, with a scale corresponding to one dose unit ("n"). This is used to draw and measure the desired dose.

TITRATION PHASE REQUIREMENTS (Phase A and B):

- On Day 1: Start with 1 dose unit in the evening (2.5 mg THC)
- On Day 4: In addition to the dose taken in the evening, start with 1 dose unit in the morning (2.5 mg THC)
- Each dose is continued for at least three days
- Dose is increased according to the titration schedule until the patient-specific effective dose is reached (see Figure 6)
- The total length of the titration phase is 21 days
- Maximum 7 dose units in the evening (17.5 mg THC)
- Maximum 6 dose units in the morning (15 mg THC)
- If serious or unbearable adverse reactions arise, the patient must inform the investigator immediately and, if necessary, reduce the dose or stop taking the IMP
- Taken in the evening: Taken between 4pm and going to bed
- Taken in the morning: Taken between waking up and 12pm
- In the event of signs of withdrawal occurring at the start of titration phase B, these can be treated symptomatically.

GUIDELINES FOR TREATMENT PHASE (Phases A, B, C, D):

- Treatment with VER-01 or placebo is stopped immediately in the event of study discontinuation
- In treatment Phases A and B, the optimal daily dose of VER-01 or placebo determined in the titration phase can be adjusted upwards or downwards by the patient if their condition or concomitant medication changes or adverse reactions causing discomfort occur. It is taken in the evening and morning, in the same way as in the titration phase.
- At the start of treatment phases C and D, the dose should be kept the same as in treatment phase B. The patient can then adjust the dose upwards or downwards if their condition or

concomitant medication changes or if adverse reactions that cause discomfort occur. It is taken in the evening and morning, in the same way as in the titration phase.

- The single dose should not exceed 8 dose units (20 mg THC) and the maximum daily dose should not exceed 13 dose units (32.5 mg THC) during the treatment phases. The minimum daily dose is 1n (2.5 mg THC), to be taken in the evening.
- Taken in the evening: Taken between 4pm and going to bed
- Taken in the morning: Taken between waking up and 12pm
- In the event of signs of withdrawal occurring at the start of treatment phase D, these can be treated symptomatically.

WASH-OUT PHASE REQUIREMENTS (Phase C and D):

- VER-01 or placebo is suddenly withdrawn at Visit C13 (Phase C) or at Visit D11 (Phase D).
- In the event of signs of withdrawal occurring in the wash-out phase, the procedure described in Chapter 7.4.1.2 must be followed.

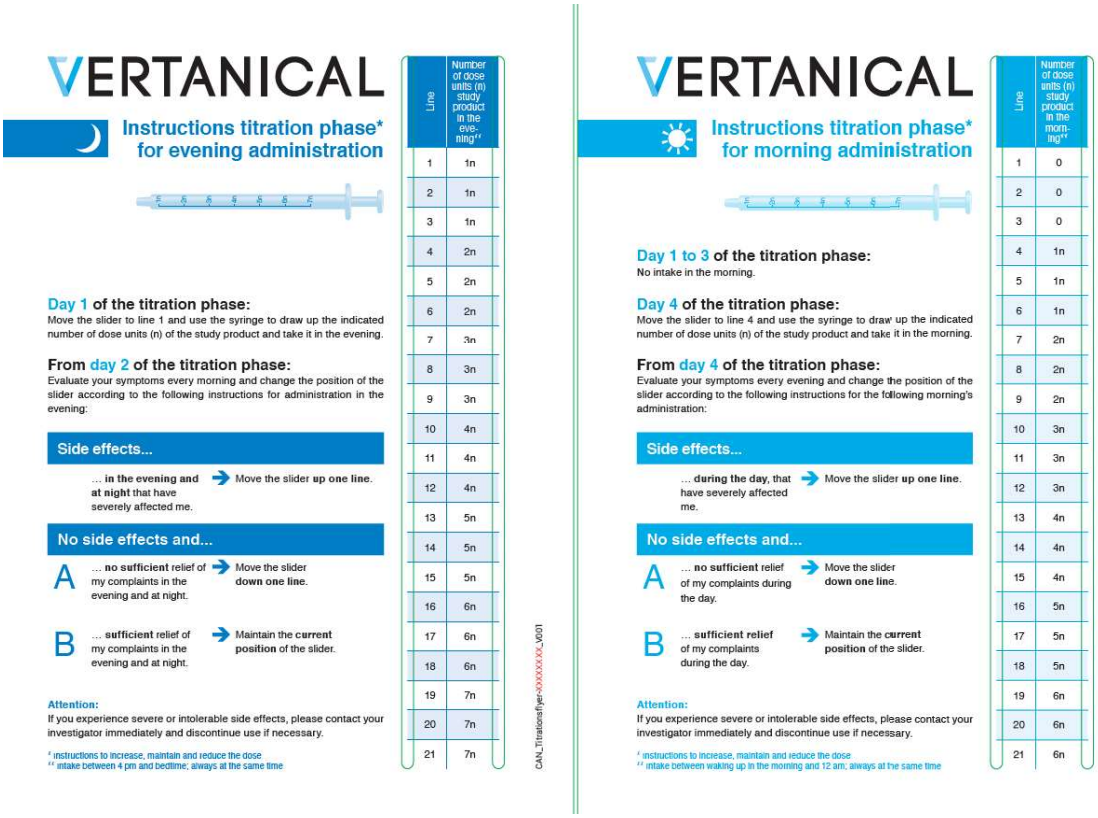

Figure 6: Instructions for intake in the morning (right) and evening (left) during the titration phase.

Rules for pausing the investigational medicinal product (phases A, B, C, D):

If detrimental adverse reactions occur in the titration or treatment phase, the patient should firstly reduce the dose in accordance with the titration regimen.

If the investigator finds temporarily suspending the investigational medicinal product to be necessary for safety reasons as a result of adverse effects, intake of the investigational medicinal product may be paused. The investigational medicinal product must only be administered again once the symptoms that led to the decision to pause use of the investigational medicinal product have completely subsided. The pause must be no longer than a maximum of seven days. If the pause lasts more than seven days, the investigator must make a decision about whether the patient is suitable for continued study participation. If the investigational medicinal product is administered again, it should be started at a maximum of 50% of the most recently administered dose. If necessary, the dose can be increased gradually in accordance with the titration regimen.

If pausing the investigational medicinal product for more than 7 days for other reasons (e.g. non-compliance by the patient), the patient's further participation in the study must be discussed with the sponsor. The decision about the patient's eligibility for further participation in the study must be made by the investigator. If the investigational medicinal product is administered again, it should be started at a maximum of 50% of the most recently administered dose. If necessary, the dose can be increased gradually in accordance with the titration regimen.

If the investigational medicinal product is permanently discontinued (regardless of the reason), it is not possible for the patient to continue participating in the study.

7. Conduct of the study

7.1 Legal framework conditions

In particular, the protocol and the study design were created in accordance with the following regulations:

- The ICH-GCP Guideline on Good Clinical Practice CPMP/ICH/135/95 (GCP Guideline)
- Declaration of Helsinki in the 1996 version (ICH Topic E6, Guideline for GCP) with the revisions of Edinburgh (2000), the clarifications of Washington (2002) and Tokyo (2004), as well as the revisions of Seoul (2008) and Fortaleza (2013),
- Regulation on the application of Good Clinical Practice in the conduct of clinical trials with medicinal products for human use (GCP Regulation - GCP-V),
- Regulation (EU) No. 536/2014 of the European Parliament and of the Council of 16 April 2014 on clinical trials of medicinal products for human use and repealing Directive 2001/20/EC
- Directive 2001/83/EC of the European Parliament and of the Council of 6 November 2001 on the Community code relating to medicinal products for human use
- Directive 2003/94/EC laying down the principles and guidelines of good manufacturing practice in respect of medicinal products for human use and investigational medicinal products for human use,
- Directive 2005/28/EC laying down principles and detailed guidelines for good clinical practice as regards investigational medicinal products for human use, as well as the requirements for authorisation of the manufacturing or importation of such products
- The detailed guidance of the European Commission on the handling of adverse drug reactions reports from clinical trials of April 2004 with the revision of 2006 (ENTR/CT3)
- Regulation (EU) 2016/679 of the European Parliament and of the Council of 27 April 2016 on the protection of individuals with regard to the processing of personal data (EU GDPR)
- Country-specific legal regulations (in particular the Medicines Act of the countries involved in the study),
- As well as other applicable legal regulations in the currently valid version.

The sponsor assures that the clinical trial is to be conducted in compliance with the approved protocol according to EMA/CHMP/ICH/135/1995 ICH E6[R2] 6.2.5 and Regulation (EU) No. 536/2014, respectively, and with the principles of GCP.

7.2 Schedule

The total duration of the clinical trial should amount to approx. 13 months per patient (in the case of participation in study Phases A, B and D) or approx. 18 months (in the case of participation in study Phases A, B and C). See Table 9 for precise timing.

Table 9: Duration of the individual treatment phases of the study

Phase	Duration
**A**	1-week screening phase to enrol eligible patients (run-in phase)
	3-week titration phase for dose-finding
	12 weeks of treatment for the patient (treatment phase)
**B**	3-week titration phase for dose-finding
	26 weeks of long-term treatment for the patient (treatment phase)
**C**	26 weeks of long-term treatment for the patient (treatment phase)
	2 weeks of follow-up of the sudden withdrawal of the IMP (wash-out phase)
**D**	4 weeks of treatment for the patient (treatment phase)
	2 weeks of follow-up of the sudden withdrawal of the IMP (wash-out phase)

7.3 Visit schedule

The total number of visits is up to 14 regular visits and eight additional visits for dispensing and returning the IMP for patients participating in Phases A, B and C and up to 12 regular visits and four additional visits for dispensing and returning the IMP for study subjects participating in Phases A, B and D. The visits should take place at the specified points in time if possible and within a timeframe of ±3 days. This excludes Visits A2, A3, A6, B7, B10 and D11, which can only take place within a time frame of +3 days, as shortening of the respective study phases should be avoided. The visits are held at the times stated in Table 10. To ensure scheduling of visits as precisely as possible by the investigator, the sponsor provides a visit planner.

If a patient cannot attend Visit A2 within the planned time window (8 + 3 days after Visit A1) due to an acute disease (e.g., fever), Visit A2 can be postponed by no more than one week (+ max. 7 days). If the patient also cannot attend Visit A2 within the prolonged period, they must be documented as a screen failure and may potentially repeat the run-in phase as part of re-screening.

Table 3: Visit schedule

Regular visit	Trial day	Time since the previous regular visit in days
A1	-8	0
A2	1	8
A3	22	21
A4	50	28
A5	78	28
A6	106	28
B7	127	21
B8	183	56
B9	239	56
B10	309	70
C11	365	56
C12	421	56
C13	491	70
C14	505	14
D11	337	28
D12	351	14

Unscheduled visits:

If an extraordinary visit takes place, it must be completely documented by the investigator. The assessments of the unscheduled visits are to be determined by the investigator; however, no THC drug test may be performed.

The procedures and activities of each individual visit are described in detail below:

Visit A1 (Week -2/Day -8): Start of the run-in phase

- Medical and data protection declaration for each patient and consent of each participating patient (signing the "Patient Information and Informed Consent Form for participation in the clinical trial")
- Issuance of the screening number
- Documentation of demographic data (age, sex, profession, ethnic affiliation)
- Measurement of vital signs (blood pressure, pulse, body temperature, weight, and height)
- Physical examination (lungs, heart, skin, lymph nodes, CNS)
- Blood draw to determine a complete blood count and clinical chemistry parameters
- Conducting a multi-panel drug test (rapid urine test) and interviewing the patient about drug/medication abuse
- Identification and documentation of possible previous and concomitant illnesses
- Completion of the questionnaire for the determination of psychological disorders (MINI)
- Ensuring that all inclusion criteria for the clinical trial are met and none of the exclusion criteria
- Documentation of potential previous and concomitant therapies
- Completion of the low back pain disability questionnaire (RMD questionnaire) by the patient
- Completion of the Neuropathic Pain Symptom Inventory (NPSI)
- Completion of the questionnaire to identify neuropathic pain (painDETECT)
- Issuing of the access data for the diary (eDiary) and introduction to the eDiary
- Ensuring that keeping the patient diary is understood by all patients
- Dispensing of rescue medication and instructions for patients
- Issuance of the study ID, issuance of the notes on concomitant therapy, and information about methods of contraception

Visit A2 (Week 1/Day 1 (+3)): Start of the titration phase in Phase A

- Perusing and discussing the diary for the run-in phase to make sure that keeping the patient diary was understood and complied with by all patients
- Review of the inclusion and exclusion criteria (potentially exclusion of patients)
- Measurement of vital signs (blood pressure, pulse, temperature)
- Pregnancy test for women of child-bearing potential
- Documentation of concomitant medications
- Return and documentation of any rescue medication taken, and any dispensing of new rescue medication
- Documentation of AEs/SAEs during the run-in phase

- Completion of the quality-of-life questionnaire (SF-36) by the patient
- Completion of the assessment of sleep quality questionnaire (MOS-SS) by the patient
- Completion of the low back pain disability questionnaire (RMD questionnaire) by the patient
- Completion of the Neuropathic Pain Symptom Inventory (NPSI)
- Issuance of the instructions for the titration phase and of additional syringes, as well as instruction of the patients
- Ensuring that keeping the patient diary and the rules for titration of the IMP are understood by all patients (especially avoiding driving a vehicle during the titration phase)
- Assignment of a randomisation number by the sponsor
- Dispensing of the IMP
- Where applicable, documentation of study drop-out following randomisation (if the patient has already taken the IMP as part of the visit, they should attend a follow-up appointment 14 days (±3 days) after drop-out)
- On 120 patients at selected study sites: Placement of the Holter monitor and instructing the patients in what must be kept in mind during the measurement (see Chapter 7.9). The ECG measurement takes 30 minutes baseline at the site and then 24 hours at home.

Visit A3 (Week 4/Day 22 (+3)): Start of the treatment phase in Phase A after titration is finished

- Perusing and discussing the diary for the titration phase to make sure that keeping the patient diary and the rules for taking the IMP were understood and complied with by all patients
- Return and weighing the IMP and check of compliance
- Measurement of vital signs (weight, blood pressure, pulse, temperature)
- Physical examination (lungs, heart, skin, lymph nodes, CNS)
- Pregnancy test for women of child-bearing potential
- Completion of the Neuropathic Pain Symptom Inventory (NPSI)
- Completion of the quality-of-life questionnaire (SF-36) by the patient
- Completion of the assessment of sleep quality questionnaire (MOS-SS) by the patient
- Documentation of concomitant medications
- Return and documentation of any rescue medication taken, and any dispensing of new rescue medication
- Documentation of AEs/SAEs during the titration phase
- Assessment of substance dependency (ABC) by the investigator
- Instruction of patients for treatment Phase A

- Ensuring that keeping the patient diary and the rules for taking the IMP are understood by all patients
- Dispensing of the IMP
- Where applicable, documentation of study drop-out (if the patient drops out of the study, they should attend a follow-up appointment 14 days (±3 days) after drop-out)

Visit A4 (Week 8/Day 50 (±3)): 1st month of the treatment phase in Phase A

- Perusing and discussing the diary for treatment Phase A to make sure that keeping the patient diary and the rules for taking the IMP were understood and complied with by all patients
- Return and weighing the IMP and check of compliance
- Dispensing of the new IMPs
- Measurement of vital signs (weight, blood pressure, pulse, temperature)
- Pregnancy test for women of child-bearing potential
- Completion of the quality-of-life questionnaire (SF-36) by the patient
- Completion of the assessment of sleep quality questionnaire (MOS-SS) by the patient
- Completion of the Neuropathic Pain Symptom Inventory (NPSI) by the patient
- Assessment of substance dependency (ABC) by the investigator
- Documentation of concomitant medications
- Return and documentation of any rescue medication taken, and any dispensing of new rescue medication
- Documentation of AEs/SAEs during the 1st month of treatment Phase A
- Where applicable, documentation of study drop-out (if the patient drops out of the study, they should attend a follow-up appointment 14 days (±3 days) after drop-out)

Visit A5 (Week 12/Day 78 (±3)): 2nd month of the treatment phase in Phase A

- Perusing and discussing the diary for treatment Phase A to make sure that keeping the patient diary and the rules for taking the IMP were understood and complied with by all patients
- Return and weighing the IMP and check of compliance
- Dispensing of new IMPs
- Measurement of vital signs (weight, blood pressure, pulse, temperature)
- Pregnancy test for women of child-bearing potential
- Completion of the quality-of-life questionnaire (SF-36) by the patient
- Completion of the assessment of sleep quality questionnaire (MOS-SS) by the patient
- Completion of the Neuropathic Pain Symptom Inventory (NPSI) by the patient
- Assessment of substance dependency (ABC) by the investigator

- Documentation of concomitant medications
- Return and documentation of any rescue medication taken, and any dispensing of new rescue medication
- Documentation of AEs/SAEs during the 2nd month of treatment Phase A
- Where applicable, documentation of study drop-out (if the patient drops out of the study, they should attend a follow-up appointment 14 days (±3 days) after drop-out)
- In patients who already received an ECG at Visit A2 at selected study sites: Placement of the Holter monitor and instructing the patients in what must be kept in mind during the measurement (see Chapter 7.9). An ECG measurement takes place over 24 hours.

Visit A6 (Week 16/Day 106 (+3)): End of treatment and study Phase A and where applicable start of the open-label titration phase of study Phase B

- Perusing and discussing the diary for treatment Phase A to make sure that keeping the patient diary and the rules for taking the IMP were understood and complied with by all patients
- Return and weighing the IMP and check of compliance
- Measurement of vital signs (weight, blood pressure, pulse, temperature)
- Physical examination (lungs, heart, skin, lymph nodes, CNS)
- Pregnancy test for women of child-bearing potential
- Blood draw to determine a complete blood count and clinical chemistry parameters
- Completion of the quality-of-life questionnaire (SF-36) by the patient
- Completion of the assessment of sleep quality questionnaire (MOS-SS) by the patient
- Completion of the low back pain disability questionnaire (RMD questionnaire) by the patient
- Completion of the Neuropathic Pain Symptom Inventory (NPSI) by the patient
- Assessment of substance dependency (ABC) by the investigator
- Assessment of the treatment results and of tolerability by the patient
- Global assessment of symptoms by the patient
- Assessment of the treatment results by the investigator based on efficacy and tolerability
- Return and documentation of any rescue medication taken, and any dispensing of new rescue medication
- Documentation of concomitant medications
- Documentation of AEs/SAEs during the 3rd month of treatment Phase A
- If applicable, scheduling a follow-up visit 14 days (±3 days) after Visit A6 for patients who are not enrolled in the subsequent study Phase B

- Ensuring that all inclusion criteria for Phase B of the clinical trial are met and there are no exclusion criteria
- Instruction of the patients in the titration phase
- Ensuring that keeping the patient diary and the rules for titration of the IMP are understood by all patients (especially avoiding driving a vehicle during the titration phase)
- Dispensing of the IMP VER-01 for the open-label titration phase

Visit B7 (Week 19/Day 127 (+3)): Start of the treatment phase in Phase B after titration is finished

- Perusing and discussing the diary for the open-label titration phase to make sure that keeping the patient diary and the rules for taking the IMP were understood and complied with by all patients
- Pregnancy test for women of child-bearing potential
- Measurement of vital signs (weight, blood pressure, pulse, temperature)
- Blood draw to determine a complete blood count and clinical chemistry parameters
- Conducting a multi-panel drug test (rapid urine test) and interviewing the patient about drug/medication abuse
- Completion of the Neuropathic Pain Symptom Inventory (NPSI) by the patient
- Assessment of substance dependency (ABC) by the investigator
- Return and documentation of any rescue medication taken, and any dispensing of new rescue medication
- Documentation of all drug and non-drug concomitant therapies
- Documentation of AEs/SAEs during the titration phase of open-label Phase B
- Return and weighing the IMP and check of compliance
- Issuance of the IMP VER-01 for the next month of open-label treatment Phase B and instruction of patients on open-label long-term treatment
- Ensuring that keeping the patient diary and the rules for taking the IMP are understood by all patients
- Where applicable, documentation of study drop-out (if the patient drops out of the study, they should attend a follow-up appointment 14 days (±3 days) after drop-out)

Visit AID1 (Week 23/Day 155 (±3)): Return and dispensing of IMP

- Return and weighing the IMP and check of compliance
- Return and documentation of any rescue medication taken, and dispensing of any new rescue medication
- Documentation of AEs/SAEs during the 1st month of treatment Phase B

- Pregnancy test for women of child-bearing potential
- Issuance of the IMP VER-01for the next month of open-label treatment Phase B and instruction of patients
- If applicable, documentation of study drop-out (if the patient drops out of the study, they should attend a follow-up appointment 14 days (±3 days) after drop-out)

Visit B8 (Week 27/Day 183 (±3)): 1st-2nd month of the treatment phase in Phase B

- Perusing and discussing the diary for treatment Phase B to make sure that keeping the patient diary and the rules for taking the IMP were understood and complied with by all patients
- Return and weighing the IMP and check of compliance
- Measurement of vital signs (weight, blood pressure, pulse, temperature)
- Performance of a pregnancy test for women of child-bearing potential
- Completion of the Neuropathic Pain Symptom Inventory (NPSI) by the patient
- Assessment of substance dependency (ABC) by the investigator
- Return and documentation of any rescue medication taken, and any dispensing of new rescue medication
- Documentation of concomitant medications
- Documentation of AEs/SAEs during the 1st-2nd months of treatment Phase B
- Dispensing of the IMP VER-01 for the next month of open-label treatment Phase B and instructing patients
- Where applicable, documentation of study drop-out (if the patient drops out of the study, they should attend a follow-up appointment 14 days (±3 days) after drop-out)

Visit AID2 (Week 31/Day 211 (±3)): Return and dispensing of IMP

- Return and weighing the IMP and check of compliance
- Documentation of AEs/SAEs during the 3rd month of treatment Phase B
- Pregnancy test for women of child-bearing potential
- Issuance of the IMP VER-01 for the next month of open-label treatment Phase B and instruction of patients

Visit B9 (Week 35/Day 239 (±3)): 3rd-4th month of the treatment phase in Phase B

- Perusing and discussing the diary for treatment Phase B to make sure that keeping the patient diary and the rules for taking the IMP were understood and complied with by all patients
- Return and weighing the IMP and check of compliance
- Measurement of vital signs (weight, blood pressure, pulse, temperature)
- Performance of a pregnancy test for women of child-bearing potential

- Completion of the Neuropathic Pain Symptom Inventory (NPSI) by the patient
- Assessment of substance dependency (ABC) by the investigator
- Return and documentation of any rescue medication taken, and any dispensing of new rescue medication
- Documentation of concomitant medications
- Documentation of AEs/SAEs during the 3rd-4th months of treatment Phase B
- Dispensing of the IMP VER-01 for the next month of open-label treatment Phase B and instructing patients
- Where applicable, documentation of study drop-out (if the patient drops out of the study, they should attend a follow-up appointment 14 days (±3 days) after drop-out)

Visit AID3 (Week 39/Day 267 (±3)): Return and dispensing of IMP

- Return and weighing the IMP and check of compliance
- Return and documentation of any rescue medication taken, and dispensing of any new rescue medication
- Documentation of AEs/SAEs during the 5th month of treatment Phase B
- Pregnancy test for women of child-bearing potential
- Issuance of the IMP VER-01 for the next month of open-label treatment Phase B and instruction of patients
- If applicable, documentation of study drop-out (if the patient drops out of the study, they should attend a follow-up appointment 14 days (±3 days) after drop-out)

Visit AID4 (Week 43/Day 295 (±3)): Return and dispensing of IMP

- Return and weighing the IMP and check of compliance
- Return and documentation of any rescue medication taken, and dispensing of any new rescue medication
- Documentation of AEs/SAEs during the 6th month of treatment Phase B
- Issuance of the IMP VER-01 for the next two weeks of open-label treatment Phase B and instruction of patients
- If applicable, documentation of study drop-out (if the patient drops out of the study, they should attend a follow-up appointment 14 days (±3 days) after drop-out)

Visit B10 (Week 45/Day 309 (+3)): End of treatment and study Phase B and potentially continuation of open-label treatment in study Phase C or randomisation and start of the blinded treatment in study Phase D

- Perusing and discussing the diary for treatment Phase B to make sure that keeping the patient diary and the rules for taking the IMP were understood and complied with by all patients
- Return and weighing the IMP and check of compliance
- Measurement of vital signs (weight, blood pressure, pulse, temperature)
- Physical examination (lungs, heart, skin, lymph nodes, CNS)
- Performance of a pregnancy test for women of child-bearing potential
- Blood draw to determine a complete blood count and clinical chemistry parameters

- Conducting a multi-panel drug test (rapid urine test) and interviewing the patient about drug/medication abuse
- Completion of the Neuropathic Pain Symptom Inventory (NPSI) by the patient
- Completion of the quality-of-life questionnaire (SF-36) by the patient
- Assessment of the treatment results and of tolerability by the patient
- Global assessment of symptoms by the patient
- Assessment of the treatment results by the investigator based on efficacy and tolerability
- Assessment of substance dependency (ABC) by the investigator
- Return and documentation of any rescue medication taken, and any dispensing of new rescue medication
- Documentation of concomitant medications
- Documentation of AEs/SAEs during the 5th-6th months of treatment Phase B
- If applicable, scheduling a follow-up visit (14 days (±3 days) after Visit B10) for patients who are not enrolled in the subsequent study Phase C or D
- Ensuring that all inclusion criteria for the clinical trial are met and there are no exclusion criteria
- Medical and data protection declaration for each patient and consent of each participating patient for study Phases C and D (signing the “Patient Information and Informed Consent Form for participation in the clinical trial”)
- Assignment of the patient to study Phase C or D by the sponsor
- Instruction of patients for “treatment Phase C” or “treatment Phase D”
- Ensuring that keeping the patient diary and the rules for taking the IMP are understood by all patients
- Dispensing of the IMP VER-01 for the next month of open-label treatment Phase C or dispensing the IMP for the entire four-week treatment Phase D and instructing the patient accordingly

Visit AID5 (Week 49/Day 337 (±3)): Return and dispensing of IMP

- Return and weighing the IMP and check of compliance
- Return and documentation of any rescue medication taken, and dispensing of any new rescue medication
- Documentation of AEs/SAEs during the 7th month of treatment Phase B
- Pregnancy test for women of child-bearing potential
- Issuance of the IMP VER-01 for the next month of open-label treatment Phase C and instruction of patients
- If applicable, documentation of study drop-out (if the patient drops out of the study, they should attend a follow-up appointment 14 days (±3 days) after drop-out)

Visit C11 (Week 53/Day 365 (±3)): 7th-8th month of the open-label treatment phase in Phase C

- Perusing and discussing the diary for treatment Phase C to make sure that keeping the patient diary and the rules for taking the IMP were understood and complied with by all patients
- Return and weighing the IMP and check of compliance
- Measurement of vital signs (weight, blood pressure, pulse, temperature)
- Performance of a pregnancy test for women of child-bearing potential
- Completion of the Neuropathic Pain Symptom Inventory (NPSI) by the patient
- Assessment of substance dependency (ABC) by the investigator
- Return and documentation of any rescue medication taken, and any dispensing of new rescue medication
- Documentation of concomitant medications
- Documentation of AEs/SAEs during the 7th-8th months of the open-label treatment Phase C
- Dispensing of the IMP VER-01 for the next month of open-label treatment Phase C and instructing patients
- Where applicable, documentation of study drop-out (if the patient drops out of the study, they should attend a follow-up appointment 14 days (±3 days) after drop-out)

Visit AID6 (Week 57/Day 393 (±3)): Return and dispensing of IMP

- Return and weighing the IMP and check of compliance
- Return and documentation of any rescue medication taken, and dispensing of any new rescue medication
- Documentation of AEs/SAEs during the 9th month of treatment Phase C
- Pregnancy test for women of child-bearing potential

- Issuance of the IMP VER-01 for the next month of open-label treatment Phase C and instruction of patients
- If applicable, documentation of study drop-out (if the patient drops out of the study, they should attend a follow-up appointment 14 days (±3 days) after drop-out)

Visit C12 (Week 61/Day 421 (±3)): 9th-10th month of the open-label treatment phase in Phase C

- Perusing and discussing the diary for treatment Phase C to make sure that keeping the patient diary and the rules for taking the IMP were understood and complied with by all patients
- Return and weighing the IMP and check of compliance
- Measurement of vital signs (weight, blood pressure, pulse, temperature)
- Performance of a pregnancy test for women of child-bearing potential
- Completion of the Neuropathic Pain Symptom Inventory (NPSI) by the patient
- Assessment of substance dependency (ABC) by the investigator
- Return and documentation of any rescue medication taken, and any dispensing of new rescue medication
- Documentation of concomitant medications
- Documentation of AEs/SAEs during the 9th-10th months of the open-label treatment Phase C
- Dispensing of the IMP VER-01 for the next month of open-label treatment Phase C and instructing patients
- Where applicable, documentation of study drop-out (if the patient drops out of the study, they should attend a follow-up appointment 14 days (±3 days) after drop-out)

Visit AID7 (Week 65/Day 449 (±3)): Return and dispensing of IMP

- Return and weighing the IMP and check of compliance
- Return and documentation of any rescue medication taken, and dispensing of any new rescue medication
- Documentation of AEs/SAEs during the 11th month of treatment Phase C
- Pregnancy test for women of child-bearing potential
- Issuance of the IMP VER-01 for the next month of open-label treatment Phase C and instruction of patients
- If applicable, documentation of study drop-out (if the patient drops out of the study, they should attend a follow-up appointment 14 days (±3 days) after drop-out)

Visit AID8 (Week 69/Day 477 (±3)): Return and dispensing of IMP

- Return and weighing the IMP and check of compliance

- Return and documentation of any rescue medication taken, and dispensing of any new rescue medication
- Documentation of AEs/SAEs during the 12th month of treatment Phase C
- Issuance of the IMP VER-01 for the next two weeks of open-label treatment Phase C and instruction of patients
- If applicable, documentation of study drop-out (if the patient drops out of the study, they should attend a follow-up appointment 14 days (±3 days) after drop-out)

Visit C13 (Week 71/Day 491 (±3)): End of the open-label treatment phase and start of the wash-out phase for study Phase C

- Perusing and discussing the diary for treatment Phase C to make sure that keeping the patient diary and the rules for taking the IMP were understood and complied with by all patients
- Return and weighing the IMP and check of compliance
- Measurement of vital signs (weight, blood pressure, pulse, temperature)
- Physical examination (lungs, heart, skin, lymph nodes, CNS)
- Performance of a pregnancy test for women of child-bearing potential
- Blood draw to determine a complete blood count and clinical chemistry parameters
- Completion of the Neuropathic Pain Symptom Inventory (NPSI) by the patient
- Completion of the quality-of-life questionnaire (SF-36) by the patient
- Assessment of the treatment results and of tolerability by the patient
- Global assessment of symptoms by the patient
- Assessment of the treatment results by the investigator based on efficacy and tolerability
- Assessment of substance dependency (ABC) by the investigator
- Assessment of substance dependence by the investigator, with potential diagnosis (F12.2 per ICD-10)
- Return and documentation of any rescue medication taken, and any dispensing of new rescue medication
- Documentation of concomitant medications
- Documentation of AEs/SAEs during the 11th-12th months of the open-label treatment Phase C
- Instruction of the patient for wash-out Phase C
- Ensuring that keeping the patient diary is understood and complied with by all patients
- Where applicable, documentation of study drop-out (if the patient drops out of the study, they should attend a follow-up appointment 14 days (±3 days) after drop-out)

Visit C14 (Week 73/Day 505 (±3)): End of study Phase C and the entire clinical trial

- Perusing and discussing the diary for the wash-out Phase C to make sure that keeping the patient diary was complied with by all patients
- Measurement of vital signs (weight, blood pressure, pulse, temperature)
- Physical examination (lungs, heart, skin, lymph nodes, CNS)
- Conducting a multi-panel drug test (rapid urine test) and interviewing the patient about drug/medication abuse
- Completion of the Neuropathic Pain Symptom Inventory (NPSI) by the patient
- Return and documentation of any rescue medication taken
- Documentation of concomitant medications
- Documentation of AEs/SAEs during the wash-out Phase C
- Where applicable, documentation of study drop-out
- Documentation of all required information at the end of the study
    - In the event of signs of withdrawal occurring in the wash-out phase, the procedure described in Chapter 7.4.2.2 must be followed.

Visit D11 (Week 49/Day 337 (+3)): End of treatment Phase D and start of the wash-out phase for study Phase D:

- Perusing and discussing the diary for treatment Phase D to make sure that keeping the patient diary and the rules were understood and complied with by all patients
- Return and weighing the IMP and check of compliance
- Measurement of vital signs (weight, blood pressure, pulse, temperature)
- Physical examination (lungs, heart, skin, lymph nodes, CNS)
- Pregnancy test for women of child-bearing potential
- Completion of the Neuropathic Pain Symptom Inventory (NPSI) by the patient
- Completion of the quality-of-life questionnaire (SF-36) by the patient
- Assessment of the treatment results and of tolerability by the patient
- Global assessment of symptoms by the patient
- Assessment of the treatment results by the investigator based on efficacy and tolerability
- Assessment of substance dependency (ABC) by the investigator
- Assessment of substance dependence by the investigator, with potential diagnosis (F12.2 per ICD-10)
- Return and documentation of any rescue medication taken, and any dispensing of new rescue medication
- Documentation of concomitant medications

- Documentation of AEs/SAEs during treatment Phase D
- Instruction of the patient for wash-out Phase D
- Ensuring that keeping the patient diary is understood and complied with by all patients
- Where applicable, documentation of study drop-out (if the patient drops out of the study, they should attend a follow-up appointment 14 days (±3 days) after drop-out)

Visit D12 (Week 51/Day 351 (±3)): End of study Phase D and the entire clinical trial

- Perusing and discussing the wash-out diary for Phase D to make sure that keeping the patient diary and the rules were complied with by all patients
- Measurement of vital signs (weight, blood pressure, pulse, temperature)
- Physical examination (lungs, heart, skin, lymph nodes, CNS)
- Conducting a multi-panel drug test (rapid urine test) and interviewing the patient about drug/medication abuse
- Completion of the Neuropathic Pain Symptom Inventory (NPSI) by the patient
- Return and documentation of any rescue medication taken
- Documentation of concomitant medications
- Documentation of AEs/SAEs during the wash-out Phase D
- Where applicable, documentation of study drop-out
- Documentation of all required information at the end of the study

In the event of signs of withdrawal occurring in the wash-out phase, the procedure described in Chapter 7.4.2.2 must be followed

Visit Follow-up (14 days (±3 days) after study dropout or 14 days (±3 days) after Visit A6/B10 for patients who are not enrolled in a subsequent study phase): final examination

- Physical examination (lungs, heart, skin, lymph nodes, CNS)
- Measurement of vital signs (weight, blood pressure, pulse, temperature)
- Conducting a drug test (rapid urine tests) for cocaine, morphine, methamphetamine, amphetamine, methadone, benzodiazepine, ecstasy
- and interviewing the patient about drug/medication abuse (THC, cocaine, morphine, methamphetamine, amphetamine, methadone, benzodiazepine, ecstasy)
- If applicable, return and weighing the IMP and check of compliance (until study drop-out; does not apply for follow-up after regular end of treatment following Visit A6/B10)
- If applicable, return and documentation of rescue medication taken (until study drop-out; does not apply for follow-up after regular end of treatment following Visit A6/B10)
- Assessment of the treatment results and of tolerability by the patient

- Global assessment of symptoms by the patient
- Assessment of the treatment results by the investigator based on efficacy and tolerability
- Documentation of AEs/SAEs
- Documentation of concomitant medications

In the event of signs of withdrawal occurring in the follow-up phase, the procedure described in Chapter 7.4.2.2 must be followed

7.4 Start and end of the clinical trial

The first patient was enrolled in the study on 07.07.2021 (FPFV).

7.4.1 Start of participation, briefing process, and consent

The nature, significance and scope of the clinical trial will be explained to each patient by the investigator in an appropriate and understandable way in an in-person consultation before their admission to the clinical trial. This includes the explanation of the potential benefits and adverse reactions to the IMP (VER-01 and the placebo), as well as the necessity and significance of a placebo-controlled clinical trial. The purpose and scope of the collection and use of personal data as part of the conduct of the study are also explained to the patient.

The patient can only be admitted to the study after granting written consent for both participation in the study and processing of personal data. The sponsor provides the study site with the "*Patient Information and Informed Consent Form for participation in the clinical trial*" form before the study begins, which includes the medical briefing/informed consent form and the data protection declaration/consent.

A copy of the *"Patient Information and Informed Consent Form for participation in the clinical trial"* form signed and dated by the patient and investigator is stored in the investigator's ISF. The patient will be given a further signed copy of the signed form along with a copy of the insurance certification with the corresponding terms and conditions. During monitoring, it is checked whether the participating patient has declared their consent before the start of the clinical trial and the patient information and informed consent form has been documented properly in the patient file.

If new information becomes available that could considerably impact future health and medical care, patients will be informed immediately and the patient information is updated accordingly. As soon as the updated patient information approved by the institutional review board/independent ethics committee is available, re-consent will be obtained from the patients.

All documents that patients receive as part of the study, especially the patient information and the informed consent form for participation in the clinical trial, as well as adverts for the clinical trial are submitted to the involved ethics committee for inspection before use.

7.4.2 End of participation

7.4.2.1 Regular and premature end of treatment

Intake of the IMP ends normally after approx. 4 (Phase A), 11 (Phase B), 18 (Phase C) or 13 months (Phase D) of study participation through sudden withdrawal. This is followed by a two-week wash-out phase (Phase C, D) or follow-up phase (Phase A, B) for follow-up.

Regular end of treatment after study Phase C or D

The final visit in study Phases C and D is Visit C14 or D12 after the end of the respective wash-out. As part of Visit C14/D12, signs of possible symptoms of withdrawal or dependency are determined by reviewing the cannabis withdrawal scale completed by the patient in the diary and recording any AEs/SAEs that occur.

During study Phase C and D, any adverse event that occurs, including potential withdrawal symptoms as they cannot be distinguished due to the blinded study design, are managed as AEs (see Chapter 8.2).

Regular end of treatment after study Phase A or B

In the event of regular end-of-treatment after study Phase A or B (patient has completed Phase A/B up to and inclusive Visit A6/B10, but is not included in a subsequent study phase), a follow-up visit will take place after a 14-day (± 3 days) follow-up phase. Until the follow-up visit, adverse events and the use of adjuvant therapies should be documented daily in the diary.

Premature end of treatment (within study Phases A, B, C, D)

In the event of premature end of treatment/study drop-out, a follow-up visit will take place after a 14-day (± 3 days) follow-up phase. Until the follow-up visit, adverse events and the use of adjuvant therapies should be documented daily in the diary.

7.4.2.2 Continued treatment after the regular or premature end of treatment

Procedure in the case of withdrawal symptoms during the 14-day wash-out phase/follow-up phase

In the event of withdrawal symptoms occurring during the regular 14-day wash-out phase/follow-up phase, these can be treated symptomatically by the investigator.

Procedure in the case of withdrawal symptoms after the 14-day wash-out phase/follow-up phase

For patients experiencing withdrawal effects beyond the 14-day wash-out phase or the 14-day follow-up phase or patients requiring a longer follow-up following a medical assessment by the investigator, the wash-out period/follow-up phase will be extended by a maximum of a further 14 days. In this case, the follow-up visit must take place after the prolonged wash-out phase/follow-up phase. If a visit takes place in the meantime, this should be documented as an unscheduled visit. The patient records any withdrawal symptoms that occur during this period in the patient diary.

In the event of withdrawal symptoms, these can be treated symptomatically by the investigator.

If the doctor believes that symptomatic treatment during the prolonged wash-out phase/follow-up phase will not suffice, a gradual taper after re-institution of at most 50% of the last daily dose of the IMP is possible. The exact duration of the re-institution and tapering phase is at the discretion of the investigator depending on the re-instituted daily dose, but must not exceed 21 days.

If the investigator finds it necessary, the patient will be referred to their general practitioner after the end of the clinical trial. With the patient's permission, the investigator will inform the general practitioner about the treatment in the clinical trial and potentially recommend further treatment.

7.4.2.3 Procedures in the event of early discontinuation of treatment at the patient's request

Every patient can withdraw from their participation in the study at any time of their own volition without stating reasons. In such cases, the date of drop-out and any potential reasons must be documented. For these patients, a final visit (the same as at the regular end of study) should be performed two weeks (± 3 days) after the study drop-out (follow-up visit). These patients should keep a daily diary of adverse events and the use of concomitant therapies until the follow-up visit. In the event of signs of withdrawal occurring in the follow-up phase, the procedure described in Chapter 7.4.2.2 must be followed. A follow-up visit after premature study drop-out is only necessary once the patient has started taking the IMP (after Visit A2). If the patient drops out due to the onset of side effects, the patient should receive medical treatment if necessary.

7.4.2.4 Procedures in the event of early discontinuation of treatment in the investigator's view

The decision about a medically necessary end of treatment is primarily taken by the Investigator. If the investigator wishes to consult on a possible end of treatment in non-urgent cases, the sponsor may be contacted (who may consult with the NCI).

Contact details:

Phone: +49 (0) 151/20928003

Availability:

- Monday - Thursday: 08:00 – 16:00
- Friday: 08:00 – 15:00

Patients who fulfil the following criteria during the clinical trial may be excluded from the study by the investigator:

- If one or more exclusion criteria arise or are detected over the course of the study
- Significant patient non-adherence/non-compliance from the investigator's point of view (patient does not follow physician's instructions to a significant extent).
- Occurrence of AEs and/or SAEs that are the result of the IMP
- Evidence of pregnancy
- Permanent loss of contact between the investigator and the patient / lost to follow-up (the investigator should attempt to contact the patient twice by telephone and, if unsuccessful, once in writing (letter/fax/e-mail). If the investigator does not receive a response after two weeks, the study termination will be documented as lost to follow-up).
- Relocation
- Serious violation of the study protocol
- Operations that preclude the consistent intake of the IMP due to the extent of the operation and the length of sick leave
- CBC lab results and/or clinical parameters provide information about serious illnesses or abnormalities the patient may have that would expose the patient to an unacceptable level of risk, or which could compromise the interpretation of the study data
- If active suicidal thoughts or suicidal behaviour of the patient is detected
- Newly developed conditions not yet described in the inclusion/exclusion criteria, but which do not allow continuation of protocol-compliant treatment because they cause substantial changes in the individual benefit-risk assessment that indicate discontinuation of study medication.
- Unblinding of the patient initiated by the investigator
- Patient's participation in another clinical trial involving an investigational medicinal product or participation in another type of medical research that is considered scientifically or medically incompatible with this study
- Patient requires treatment with another medication that has been shown to be effective for treating the study indication
- Patient withdraws consent to participate in study

If a patient needs to be excluded from the study based on the doctor's assessment, the two-week (± 3 days) follow-up period (follow-up visit) should still be observed, if exclusion happened after the first intake of the IMP (Visit A2).

In the event of signs of withdrawal occurring in the follow-up phase, the procedure described in Chapter 7.4.2.2 must be followed.

In the event of psychological problems (e.g., suicidal thoughts, suspected psychosis) being detected, patients should be referred to their general practitioner. If the contact details of the general practitioner have not been given in the document "Patient information and consent form" and/or the patient is currently not in the care of a general practitioner, patients will be referred to a specialist by the investigator.

7.4.3 Procedure for replacing patients

Patients who are excluded from further study participation prior to randomization R1 will be replaced by new patients. Patients who are excluded from further study participation after randomization R1 or R2 should not be replaced by new patients.

7.4.4 End of the clinical trial

The Last Patient, Last Visit (LPLV) is defined as the regular end of the clinical trial. The end of the study is communicated in accordance with country-specific legislation. Within a year of the end of the clinical trial, the sponsor sends a summary of the final report, which contains all significant events, to the responsible competent authority and institutional review board/independent ethics committee. The final report by the monitor marks the proper closure of the respective study sites.

7.4.4.1 Premature discontinuation of the study at individual study sites

The investigator (or if applicable their deputy) can stop the clinical trial early, if they e.g.

- assess the continuation of the study as no longer reasonable from an ethical and/or medical perspective
- believe that the resources for continuation can no longer be covered (e.g., lack of personnel or equipment capacities)

The sponsor is authorised to exclude some study sites from the clinical trial at any time for medical or administrative reasons and to demand the return of all study materials from these study sites. Possible reasons for closing a study site are in particular:

- the conduct of the study in accordance with the protocol proves to be infeasible at this site
- the rate of recruitment is insufficient

- data quality is insufficient
- false information is demonstrably provided in the CRF
- the confidentiality agreement or the investigator contract is violated
- other study site-related termination criteria defined in the protocol are met

In the event of the premature end of the clinical trial at a study site, the investigator must inform the patients immediately. The patients included before the study discontinuation will be treated further and documented in accordance with the protocol until the individual study end.

7.4.4.2 Cancellation of the entire study by the sponsor

Should relevant medical or ethical concerns arise during the clinical trial or if the clinical trial proves to be infeasible, the entire study will be terminated by the sponsor. In such an event, the reasons will be documented in detail. Patients who were still receiving treatment at the time of study termination must undergo a final examination that is documented accordingly. The investigator may recommend further treatment to the patient's general practitioner. The sponsor takes the decision to terminate the trial in consultation with the NCI and if necessary, an independent statistician.

The premature termination of the clinical trial will be considered particularly, in the following cases

- the risk/benefit ratio for the patients changes significantly
- the use of the IMP is no longer justifiable
- the sponsor feels that stopping the clinical trial is necessary for safety reasons (e.g., on advice of the NCI)
- early evidence of superiority or inferiority of a treatment group is provided based on an interim analysis or other research results
- the clinical trial proves to be infeasible
- the patient recruitment rate is insufficient
- if the number of dropouts is so high that an orderly completion of the clinical trial can no longer be expected
- decision of the competent authority, e.g., as a result of an inspection
- withdrawal of the approval by the institutional review board/independent ethics committee, e.g., in the case of serious violations of GCP, the protocol, or misconduct that unacceptably compromises the safety or rights of patients or questions the validity of the study results.

The termination of the study is reported to the involved ethics committee and authorities by the sponsor in line with the deadlines specified in national legislation.

7.5 Medical history

During Visit A1, the medical history of the patient is documented by the investigator for at least two years prior to the start of the study. During this period, all diseases except non-relevant diseases (e.g., flu-like infection, gastrointestinal infection) of the patient have to be documented. In addition, all the patient's serious illnesses (e.g., cancer, stroke, myocardial infarction) and operations over the period of two years before the start of the study must also be documented.

7.6 Previous and concomitant therapy

Patients with chronic non-specific low back pain when drug treatment is indicated and a previous optimised treatment with non-opioids has not led to sufficient pain relief or was unsuitable due to contraindications or intolerance can be enrolled in the study.

Drug treatment is indicated if analgesic drug therapy is considered supportive for the realisation of activating measures, or if the patient has unbearable functional disabilities as a result of the pain, despite regularly performing these measures. In addition, treatment is considered optimised when a further increase in drug dose is unsuitable from a medical perspective considering side effects and/or it is not expected that a higher drug dose would result in a further advantage in terms of efficacy.

In order to be enrolled, no analgesics must have been taken in the last seven days before the start of the study. Also, during study Phases A and D, in which the efficacy and maintenance of efficacy of VER-01 is investigated, no additional analgesic medication (non-opioid and opioid analgesics, as well as adjuvant analgesics) are allowed. The use of rescue medication is only permitted in the case of acute pain worsening (conditions for use described in Chapter 7.7). During study Phases B and C, the start of new drug treatments is permitted, but the dosage should be kept as stable as possible. Non-opioid and adjuvant analgesics are listed by pharmaceutical class with examples in Table 5 .

With regards to additional non-drug therapies (e.g., physical or behaviour therapy, acupuncture, massage, thermotherapy) that significantly modulate pain sensation, such therapies must have been applied unchanged for at least nine weeks before the start of the study and the start of Phase D. Existing therapies should be continued as planned during study participation in Phase A and D, but no new therapies shall be started. Existing therapies must have been applied for at least eight weeks before the start of the study and start of Phase D.

During study Phases B and C, non-drug therapies can be restarted, continued, or terminated. At no point during participation in the study may cannabis-based medications on the market be taken. Examples of non-drug therapies are listed in Table 6.

At the start of the clinical trial (Visit A1), the investigator documents all previous optimised drug treatments in the CRF as well as current drug or non-drug therapies used for their chronic non-specific low back pain. In the case of drug treatments, the product name (trade name and strength specified), the pharmaceutical form, dosage, and period of use are specified. For previous non-drug therapies, a clear description, duration/frequency, and period of use are noted. In the case of current concomitant drug and non-drug therapies, the indication is documented for each treatment in addition to the previously described information.

At Visit A1, the investigator explains the procedure with existing and new treatments during participation in the study to the patient. The investigator ensures that the study does not result in the patient suspending any medication that is necessary under the guidelines due to a concomitant illness.

7.7 Rescue medication

Tablets of ibuprofen (800 mg) are available as rescue medication. They can be taken at a daily dose of up to 2,400 mg and, regardless of the dose, on a maximum of three days per week (no intake within the 24 hours prior to visits with the exception of AID visits 1-8, the follow-up visit and unscheduled visits). When ibuprofen is contraindicated, e.g., due to existing T-ASA therapy or the concomitant disease Meulengracht's disease (Gilbert's syndrome), paracetamol can be used as rescue medication in the form of tablets (500 mg) in the case of acute pain worsening (max. 4,000 mg/d and, regardless of the dose, on a maximum of three days a week; no intake within the 24 hours prior to visits, with the exception of AID visits 1-8, the follow-up visit and unscheduled visits). When taking ibuprofen as rescue medication, pantoprazole should be used (according to the packaging insert/summary of product characteristics, a 20 mg tablet should be taken per day if ibuprofen was taken) to prevent gastric ulcers. The study site provides patients with a pack of rescue medication at Visit A1. Additional medication is only dispensed when necessary, queried at each visit. The use of the rescue medication must be documented in the diary by the patient and is automatically displayed in the eCRF. The patient must take the rescue medication with them to each visit and the investigator must check whether the use of the rescue medication documented in the patient diary matches up with consumption, based on the missing tablets in the blister pack. Any used or unused rescue medication must be returned to the investigator by the patient (including empty packets). Dispensing and return are documented in the CRF and ISF by the investigator and monitored by the monitor. The study sites receive a stock of rescue medication from the sponsor.

7.8 Collection, storage, and pick up of the samples

At Visits A1, A6, B7, B10 and C13, blood samples are drawn to analyse the complete blood count and to determine clinical chemistry parameters. For this purpose, around 15.5 ml of blood is drawn from each patient and stored properly at the study site until pick-up. The study sites receive kits from the central laboratory for this purpose. If necessary, these can be reordered via the central laboratory – instructions with the precise description of blood draws, storage, shipment, and ordering can be found in the ISF. The sponsor provides normal range values for the complete blood count and clinical chemistry values as well as quality certification of the central laboratory in the ISF. The following blood values are tested:

<u>Complete blood count:</u>

Leukocytes, absolute and percentage of lymphocytes, monocytes, basophils, eosinophils, neutrophils, thrombocytes, erythrocytes, haemoglobin (MCH and MCHC), haematocrit and MCV value (mean corpuscular volume)

Clinical chemistry parameters:

Alanine aminotransferase (ALT), aspartate aminotransferase (AST), albumin, calcium, chloride, alkaline phosphatase, bicarbonate, cholesterol, creatine-kinase (CK), creatinine, direct bilirubin, lactate dehydrogenase (LDH), magnesium, globulin, glucose, gamma-glutamyl-transferase (GGT), indirect bilirubin, phosphate, potassium, sodium, total bilirubin, total protein, triglycerides, urea, uric acid.

Drug test (rapid urine test)

In addition, the presence of THC (only at screening Visit A1 and the visits in study Phases B and C), cocaine, morphine, methamphetamine, amphetamine, methadone, benzodiazepine and ecstasy is determined at Visits A1, B7, B10, C14, D12 and the follow-up visit by using a rapid urine test to screen for possible drug or medication abuse, as well as previous use of cannabis-based preparations and opioids (see Section 5.4 exclusion criteria). The drug tests are used according to the manufacturer's instructions for use.

At Visits A1, B7, B10 and C14, the following substances are tested for via a drug test: THC, cocaine, morphine, methamphetamine, amphetamine, methadone, benzodiazepine and ecstasy.

At Visits D12, all follow-up visits and, if a drug test is performed during an unscheduled visit, the following substances are tested for via a drug test: Cocaine, morphine, methamphetamine, amphetamine, methadone, benzodiazepine and ecstasy.

Patients who have a positive result for one of the substances at Screening Visit A1 will not be included in the study according to the exclusion criteria.

Since the drug tests can only detect the substances mentioned for short period of time before last administration and does not test for alkyl nitrites, barbiturates, mephedrone and alcohol, the investigator must additionally ask the patient at the visits mentioned whether any of the substances (THC, cocaine, morphine, methamphetamine, amphetamine, methadone, benzodiazepine, ecstasy, alkyl nitrites, barbiturates, mephedrone, alcohol) have been used in the past (at Visit A1) or have been used since the last visit (at B7, B10, C14, D12 and the follow-up visit) for non-medical purposes over a longer period of time and assess whether alcohol/drug/medication abuse is suspected from a medical perspective.

Patients who test positive for any of the substances (except THC and morphine, if taken for medical purposes) at Visit B7 and at the end of Study Phase B (Visit B10), or if alcohol/drug/medication abuse

is suspected by the investigator or becomes known through interviewing the patient, will be excluded from further study participation.

Patients who test positive for morphine and have not abused it must discontinue morphine or the opioid in question or pause the investigational medicinal product if, in the opinion of the investigator, there is a safety risk from concomitant use with the investigational medicinal product. The procedure for pausing the investigational medicinal product is described in more detail in Chapter 6.10. In the event of misuse of morphine (suspected by the investigator or confirmed by the patient), the patient's study participation must be terminated.

Patients who test positive for morphine at Visit B10 cannot be included in Study Phase D according to exclusion criterion d1. For these patients, inclusion in Study Phase C is only possible if the additional inclusion criteria for Study Phase C (c1 - c3) are met and no misuse of morphine (suspected by the investigator or confirmed by the patient) has occurred.

Furthermore, the drug tests will be performed at the end of study participation (Visit C14, D12, follow-up visit). Patients who test positive for any of the substances (except THC and morphine) or alcohol/drug/medication abuse is suspected by the investigator or confirmed by patient interview will, with the patient’s consent, be referred to their primary care physician and will receive contact information for an addiction counseling center from the investigator.

7.9 Recording the 24-hr ECG

During Phase A of the study, a 24-hr ECG is performed on two occasions on a subpopulation of approx. 120 patients at selected study sites. The baseline ECG is recorded for these patients during Visit A2 over a period of 30 minutes. The first 24-hour ECG is then taken for these patients (after the 1st dose). The second 24-hr ECG is started during Visit A5 (after the patient-specific effective dose has been taken for eight weeks).

At Visit A2, a Holter monitor is fastened to the patient’s upper body at the study site for approx. 24 hours and the procedure is explained. In these 24 hours, the 24-hr ECG is recorded after the IMP is taken in the evening for the first time. Because Visit A2 must take place before 4 p.m., the recording takes longer than 24 hours, because the patient should start taking the IMP for the first time in the evening (defined from 4 p.m. until bedtime) and an ECG should be recorded for 24 hours from that time on. At Visit A5, a Holter monitor is again fastened to the patient’s upper body at the study site for another 24 hours

During the ECG measurement, the patient can go home and go about their daily life, but must not exercise or shower. After the end of the ECG measurement, the patient is asked to return the device to the investigator. A detailed description of the use of the ECG devices can be found in the ISF.

The ECG devices are supplied by the sponsor; the evaluation of the QT interval at predefined points in time is performed by a cardiologist assigned by Vertanical.

7.10 Ability to drive

During both titration phases in Study Phases A and B, patients should not drive vehicles, as adverse reactions could impair the ability to drive until the patient's ideal dose is found. Patients are informed that the ultimate decision about whether they are able to drive is up to them.

7.11 Travelling abroad

In some countries, it is illegal to carry cannabis-based products with you. Patients are therefore instructed to always check the legal status of VER-01 before they travel abroad and carry their study ID with them at all times.

7.12 Contraceptive methods for study participants

According to the inclusion criterion, women and men must agree to use a reliable method of contraception during study participation and for three months after the last dose of the investigational medicinal product. Male study participants must declare their willingness to explain participation in this clinical study to their female sexual partners and the need for contraception. The following contraceptive methods are considered acceptable:

1. combined (containing both estrogen and progestogen) hormonal contraception associated with inhibition of ovulation (oral*, intravaginal, transdermal)
2. progestogen-only hormonal contraception associated with inhibition of ovulation (oral*, injectable, implantable)
3. intrauterine device (IUD)
4. intrauterine hormone-releasing system (IUS)
5. bilateral tubal occlusion
6. vasectomised partner (provided that partner is the sole sexual partner of the study participant and that the vasectomised partner has received medical assessment of the surgical success).
7. sexual abstinence (defined as refraining from heterosexual intercourse during the entire study period and for at least three months after the end of the study).

*Please note that oral hormonal contraception does not provide sufficient protection against pregnancy, since diarrhea and/or vomiting may occur as possible side effects of the investigational medicinal product (see 8.12.2). In the event this contraceptive method is used, an additional non-oral contraceptive method (e.g., condom) must be used during and at least three months after termination of study participation.

For women of child-bearing potential, a pregnancy test (rapid urine test) will be performed regularly during visits to exclude the possibility of pregnancy.

A woman is considered of childbearing potential (WOCBP), i.e. fertile, following menarche and until becoming post-menopausal unless permanently sterile. Permanent sterilisation methods include hysterectomy, bilateral salpingectomy and bilateral oophorectomy.

A postmenopausal state is defined as no menses for 12 months without an alternative medical cause.

7.13 Randomisation and blinding, and assignment to study phases

The study involves two randomisations at the start of Phases A and D, respectively.

7.13.1 Randomisation R1 to treatment arms in placebo-controlled Phase A

The allocation of patients to treatment with VER-01 or placebo takes place at Visit A2 when the patient's participation in the study and treatment have been confirmed after all inclusion and exclusion criteria have been reviewed. To check inclusion criterion no. a1, the average NRS pain score on the morning of the seven-day run-in phase prior to Visit A2 (study Week -1) is recorded in the eCRF and displayed (at least 5 of the 7 values must be available for calculation). Randomisation to the VER-01 vs. placebo treatment groups is carried out in a 1:1 ratio and stratified according to the final score of the painDETECT questionnaire at visit A1 (>12 or ≤12), in order to ensure that a sufficient number of patients with a neuropathic pain component are enrolled. The painDETECT questionnaire is a validated screening instrument here for the detection of neuropathic pain components.[58] For patients with a final score of ≤ 12 points, the presence of a neuropathic pain component is unlikely (< 15%); a neuropathic pain component can be present between 13 and 18 points, and above 18 points, the presence of a neuropathic pain component is very likely (> 90%). While stratification is performed at a cut-off value of 12, the key secondary endpoint is, however, evaluated at a cut-off value of > 18, taking into account a written statement of the BfArM (dated April 11, 2022), as the BfArM advises against a pooled analysis of patients with a score of > 18 and a score between 13 and 18.

The allocation of patients to treatment groups is controlled so that a total of 50% of randomized patients have a painDETECT score >12 and 50% of randomized patients a painDETECT score ≤ 12. Upon inclusion of all patients to be randomized (404 patients) from one of the two patient groups (painDETECT score >12 or ≤ 12), the sponsor will inform all study sites and instruct it not to include any further

[58] Freynhagen, Rainer, et al. "Pain DETECT: a new screening questionnaire to identify neuropathic components in patients with back pain." *Current medical research and opinion* 22.10 (2006): 1911-1920.

patients from the relevant patient group in the study at Visit A1. The study completion of the respective patients will be documented by the investigator as "stratum full" at Visit A1.

For randomisation, a telephone hotline has been set up by the sponsor.

Contact details:

Tel.: +49 (0)151/21317922

Office hours:

- Monday - Thursday: 08:00 to 16:00
- Friday: 08:00 to 15:00

Randomisation R1 takes place as follows:

- Before making contact, the study site must make sure that the patient has not violated any of the inclusion or exclusion criteria. Otherwise, the patient must be regarded as a screen failure and randomisation cannot take place
- The investigator or an authorised member of staff calls the hotline
- The study site number, screening number and painDETECT score of the patient are transmitted by the study site, as well as the average NRS pain intensity on the morning of the 7 days before Visit A2 (study week -1). The average NRS pain intensity on the morning of study Week -1 is calculated automatically in the eCRF after Visit A2 (incl. date) (at least 5 values must be available for calculation)
- In case that for one of the two patient groups (painDETECT Score >12 or painDETECT Score ≤ 12) all patients to be randomized (404 patients) have already been included, the study site will be informed that the patient will be excluded from further study participation. The study completion of the respective patient must be documented by the investigator at Visit A2 as "stratum full"
- The caller (if all inclusion and exclusion criteria are met) will be informed of the randomisation number of the patient used to identify the IMP to be dispensed (the sponsor selects the randomisation number using a computer program)
- The investigator takes two bottles from the packaging with the corresponding randomisation number and hands them out to the patient

Randomisation takes place in appropriate blocks in accordance with a balanced group design using a computer-generated randomisation list. The size of the randomisation blocks will not be disclosed to the investigator. Randomised patients who drop out of the clinical trial retain their randomisation number.

After Phase A, all patients who meet the requirements for further participation (see Chapter 5.3) transition to Phase B, if Phase B has not yet reached 500 cases at visit B7

7.13.2 Assignment to study Phase C or D and randomisation R2 to treatment groups in placebo-controlled Phase D

The second randomisation R2 is used to assign treatment groups for placebo-controlled Phase D. New randomisation numbers are used in randomisation R2.

At the end of Phase B at Visit B10, the responder status at the end of Phase B will be determined for all patients who have completed Phase B. A patient is considered a responder if a relative improvement in pain intensity in the morning on the 11-point NRS scale of at least 30% compared to baseline was achieved at the end of treatment Phase B. This is done by comparing the patient-specific relative change in the mean value of the NRS pain intensity in the morning of study Week 43 with the mean value of the NRS pain intensity in the morning within the seven days prior to Visit A2 (study Week -1). To calculate the average value of study Week 43, at least four of out seven diary entries must be available in the morning. If there are fewer diary entries, the patient is automatically considered a non-responder. The responder status of the patient at the end of Phase B is calculated automatically in the eCRF.

Up to 120 responders are to be included in Phase D. Patients will be assigned to the two treatment groups VER-01 vs. placebo in a 1:1 ratio stratified according to the presence of a neuropathic pain component at Phase A baseline as described for randomisation R1. Upon inclusion of all patients to be randomized (60 patients) from one of the two patient groups (painDETECT score >12 or painDETECT score ≤ 12), patients from the respective patient group with responder status will be included in Phase C. Patients are assigned to treatment groups in Phase D using an appropriate block size that is not disclosed to blinded personnel.

Allocation to study Phase C or D, and if applicable randomization R2, takes place as follows:

- Before making contact, the study site must make sure that the patient, at least from one of the two study phases (Phase C or D), has not violated any of the inclusion or exclusion criteria. Otherwise, the patients' participation in the study should end as normal after the end of study Phase B.
- The investigator or an authorised member of staff calls the hotline (see section on randomization R1 for contact details)
- The investigator or authorised personnel convey the study site number, screening number, painDETECT score and the patient's responder status at the end of Phase B as indicated in the eCRF (responder/non-responder). The responder status is calculated automatically in the eCRF after Visit B10 (incl. date).
- The caller will be informed of the patient's assignment to Phase C or D

If allocated in Phase D, the caller is given the patient randomisation number, which identifies the IMP to be dispensed to the patient. Patients who are non-responders at the end of Phase B are allocated to Phase C by the hotline. Phase C patients will receive long-term treatment with VER-01 for a further six months. A minimum of 150 patients will be enrolled in Phase C, of which at least 20 patients should be responders.

7.13.3 Blinding

As part of blinding, the two independent randomisation lists described above are created for study Phases A and D. These lists contain an allocation of the randomisation numbers to the treatment (VER-01 or placebo). The secondary packaging and IMPs are labelled with the corresponding randomization numbers.

A sealed copy of the randomisation list is stored by the randomisation list manager (a person at the sponsor not involved directly in the study conduct, who created the randomisation list) and is only known to the randomisation list manager and the contract manufacturer of the IMPs. Neither person is involved in the conduct of the study. The randomisation list is not made known to the study site, the blinded biostatisticians, the sponsor's project team, or the clinical monitor.

7.14 Unblinding via emergency envelopes

Emergency envelopes are created together with each randomisation to enable immediate unblinding in an emergency. When the IMPs for Phases A and D are delivered, each package contains the corresponding emergency envelope. The inside of the envelope must not be visible from the outside. The following information is contained on or in the emergency envelope:

On the emergency envelope:

Study title

Study number or protocol code

Note: "This envelope must only be opened in an emergency!"

Randomisation number

In the emergency envelope:

Study title

Study number or protocol code

Randomisation number

Treatment group

The emergency envelopes are stored in the ISF after the box is opened. The sponsor documents how many emergency envelopes were produced, to whom they have been distributed to and when they were picked up again. At each monitoring visit, the monitor checks the integrity of the seal of each emergency envelope or whether opening the envelope was justified and documented.

The investigator has the sole authority to decide whether to perform an emergency unblinding. If an emergency envelope is opened, the date, the time and reason for opening are marked on the envelope and signed by the investigator. The sponsor must also be informed immediately. In the event that emergency unblinding is necessary but the investigator cannot be reached, the patient's study ID card lists the telephone number of a contact person (tel.: +49 (0) 89 896 799 52) who can be contacted 24/7 (24 hours, Monday - Sunday, including holidays) by medical personnel to perform the emergency unblinding.

At the end of the clinical trial, all emergency envelopes (opened or unopened) are returned to the sponsor.

7.15 Efficacy and safety parameters

7.15.1 Measurement of efficacy and safety parameters

7.15.1.1 Primary endpoints

Below, all primary objectives to be recorded within study Phases A-D are explained in detail. The precise collection times or which data is recorded at each visit can be found in the visit schedule in Chapter 7.3 and Table 11 in Chapter 7.15.2.

<u>Primary endpoint in Phase A:</u>

The intensity of pain is evaluated on an 11-point NRS scale. Patients document their pain sensation in a diary every day in the morning immediately after waking up and in the evening immediately before going to bed. The question is:

"Click the number that describes your average pain over the last 12 hours (where 0 = "no pain" and 10 = "worst pain imaginable")"

The primary efficacy endpoint is the absolute change from baseline in mean pain intensity at study Week 15 measured in the morning on the 11-point NRS scale. The mean pain intensity at study week 15 is the mean value of the NRS morning pain intensity recorded in the diary over the seven days in Week 15 of the study. The mean NRS baseline pain intensity is defined as the mean value of the NRS morning pain intensity recorded in the diary over the seven days before Visit A2 (study Week -1). The

difference between the mean NRS pain intensity at study Week 15 and at baseline is calculated individually for each patient.

Primary endpoint in Phase B:

The primary objective in Phase B is the number and proportion of patients who developed an AE in Phase B that the investigator believes was related to the use of VER-01 and the number and severity of these AEs.

Primary endpoint in Phase C

The primary objective of Phase C is defined in the same way as the primary objective of Phase B.

Primary endpoint in Phase D

The primary endpoint of Phase D is time to treatment failure, which is the time in days from randomisation to Phase D (R2) until the first day of treatment failure. Treatment failure is assessed by the daily calculated seven-day mean value of the NRS pain score in the morning during the treatment period, which must have deteriorated by at least 20% and at least 1 point compared to baseline (mean value of study Week 43). The first day within this seven-day window for which this criterion is fulfilled is subsequently defined as the first day of treatment failure. Furthermore, treatment failure is defined as an early discontinuation of treatment for selected reasons (see Chapter 9).

The pain intensity is recorded by the patient in the diary on an 11-point NRS scale in the morning during the entire four-week treatment phase.

7.15.1.2 Secondary and additional objectives

Below, all secondary objectives to be recorded within study Phases A-D are explained in detail. The precise collection times or which data is recorded at each visit can be found in the visit schedule in Chapter 7.3 and Table 11 in Chapter 7.15.2.

Mean change in neuropathic pain

Neuropathic pain is measured by the Neuropathic Pain Symptom Inventory (NPSI). The questionnaire contains 12 questions to distinguish and quantify five different clinically relevant dimensions of neuropathic pain syndromes. The evaluation is based on the total score and individual subscores (spontaneous burning pain, spontaneous pressing pain, paroxysmal pain, evoked pain and paraesthesia/dysthesia) according to Bouhassira *et al.* (2004).[59]

[59] Bouhassira, Didier, et al. "Development and validation of the neuropathic pain symptom inventory." *Pain* 108.3 (2004): 248-257.

The key-secondary endpoint of Phase A is defined as the absolute change from baseline of the NPSI total score in patients with a painDETECT score > 18 at the end of Phase A. The difference between the NPSI total score at Visit A6 and the NPSI total score at Visit A2 (baseline) is calculated individually for each patient.

As further secondary endpoints of Phase A, the absolute changes compared to baseline of the individual NPSI subscores at Visit A6 are analysed. The absolute change compared to baseline at Visit A6 is defined analogously to the total NPSI score. In Phases B, C and D, the absolute change from baseline of the total NPSI score and the individual NPSI subscores at the end of the treatment of the respective phase are evaluated as secondary efficacy endpoints.

Pain responders (30% and 50%) in the morning, as well as in the morning and evening

The secondary efficacy endpoints for Phase A are defined as the number and proportion of 30% and 50% pain responders in the morning, and morning and evening at the end of Phase A (at study Week 15).

A patient is defined as a 30%/50% pain responder in the morning or morning and evening if the relative improvement over baseline (study Week -1) in mean pain intensity in the morning or morning and evening on the 11-point NRS scale at study Week 15 is at least 30%/50%. The calculation of the patient-specific mean pain intensity at baseline and study Week 15 is analogous to the primary endpoint.

In Phases B, C and D, the number and proportion of 30% and 50% pain responders at the end of treatment of the respective phase is considered to be the secondary efficacy endpoint, analogous to Phase A.

Change in pain intensity in the morning, in the evening, as well as in the morning and evening

In study Phases A, B, C and D, absolute changes from baseline in mean pain intensity at the end of each treatment phase measured in the morning (primary endpoint of Phase A), in the evening, or morning and evening on the 11-point NRS scale are evaluated as secondary efficacy endpoints. The calculation of the patient-specific mean pain intensity at the end of the treatment phase and at baseline (study Week -1) is analogous to the primary efficacy endpoint of Phase A. In Phase D, the mean value of the pain intensity in the morning, in the evening, or morning and evening at study Week 43 (end of treatment phase B) serves as the baseline value.

Sleep quality NRS

Sleep quality is evaluated daily in the patient diary on an 11-point NRS scale. The question is:

> “Click the number that states how greatly your pain impacted your sleep last night (where 0 = “no impact” and 10 = “completely impacted”)”

The absolute change from baseline in mean sleep quality over the course and at the end of each treatment phase measured on the 11-point NRS scale is evaluated as a secondary efficacy endpoint in Phases A, B, C and D. Mean baseline sleep quality is calculated as the average of the seven days before Visit A2 (study Week -1), and mean sleep quality at the end of the treatment phase is calculated as the average of the seven days in the last scheduled study week of each phase.

Sleep quality MOS-SS

A further evaluation of sleep quality is performed by the patient using the Medical Outcomes Study Sleep Scale (MOS-SS), which includes 12 questions to assess sleep quality. The analysis is performed in accordance with Hays *et al.* 2005[60], with sleep quality over the past four weeks being assessed.

As a secondary endpoint in Phase A, the scores and absolute changes from baseline of sleep quality measured by MOS-SS per survey point are evaluated.

Intake of rescue and concomitant medication

The rescue and concomitant medication taken is documented daily in the patient diary using the following questions:

"In the last 24 hours, have you taken rescue medication (ibuprofen or paracetamol) to treat your chronic non-specific low back pain (possible responses: Yes/No)?

If "Yes": "Please select which:" (possible response: ibuprofen/paracetamol)

If Ibuprofen: "Number of Ibuprofen tablets (800 mg)" (input as free text)

"Have you also taken pantoprazole?" (Possible responses: Yes/No)

If yes: "Number of pantoprazole tablets (20 mg)" (input as free text)

If paracetamol: "Number of paracetamol tablets (500 mg)" (input as free text)

"In the past 24 hours, have you taken or used a new medication or has existing intake or use changed (e.g. in relation to dosage or frequency)? AND/OR In the past 24 hours, have you taken or used a new non-pharmaceutical treatment or have you changed your use of an existing non-pharmaceutical treatment (e.g. in relation to dosage or frequency)?" (Possible responses: Yes/No)

[60] Hays RD, Martin SA, Sesti AM, Spritzer KL (2005): Psychometric properties of the Medical Outcomes Study Sleep measure; Sleep Medicine; 6; 41 – 44. doi:10.1016/j.sleep.2004.07.006

If yes: “Please make one or multiple new entries on the welcome page under “Use of medications and/or non-pharmaceutical treatments”. In the event that existing medication or non-pharmaceutical treatment was changed, please enter the end date for the first entry afterwards.”

In addition, the concomitant medication taken at the visits are documented by the investigator in the CRF.

The secondary endpoint for Phases A, B, C and D is the cumulative dose of rescue medication taken and the relative cumulative dose of rescue medication taken in relation to the individual duration of the respective study phase within the respective Phase.

Depression Anxiety Stress Scales (DASS)

Depression, anxiety, and stress are recorded weekly using the Depression-Anxiety-Stress Scales (DASS) in the patient diary in the morning. The scales consist of seven questions each on the individual parameters of depression, anxiety and stress, recorded in relation to the previous week. The evaluation is based on sum scores according to Nilges and Essau (2015)[61].

As a secondary endpoint in Phases A-D, the scores and absolute changes from baseline of depression, anxiety and stress levels measured by the DASS per time point of assessment are evaluated.

Global assessment of symptoms PGIC

The global assessment of symptoms by the patient is queried at the end of each treatment phase at the visit using the Patient Global Impression of Change (PGIC) with the following question:

“How is your low back pain in comparison to before participation in the study?”

The responses are recorded numerically on a 7-point Likert scale.

The evaluation is both numerical and based on the proportion of patients per category of the global assessment of symptoms by the patient per survey point.

Satisfaction with the treatment result – patient

Satisfaction with the treatment result is evaluated by the patient at the end of each treatment phase using a 5-point Likert scale with the question:

“How satisfied are you with the treatment result?”

The evaluation is both numerical and based on the proportion of patients per category of satisfaction with the treatment outcome by the patient per survey point.

Satisfaction with tolerability - patient

[61] Nilges, P., and C. Essau. "Die depressions-angst-stress-skalen." Der Schmerz 29.6 (2015): 649-657.

Satisfaction with tolerability is evaluated by the patient at the end of each treatment phase using a 5-point Likert scale with the question:

"How satisfied are you with the tolerability of the investigational medicinal product?"

The evaluation is carried out both numerically and based on the proportion of patients per category of satisfaction with the patient's tolerability per survey point.

Satisfaction with the treatment result – investigator

Satisfaction with the treatment result is evaluated by the investigator at the end of each treatment phase using a 5-point Likert scale with the question:

"How satisfied are you with the treatment result for this patient?"

The evaluation is both numerical and based on the proportion of patients per category of satisfaction with the treatment outcome by the investigator per survey point.

Safety and adverse reactions

The evaluation of the safety and adverse reactions of the IMP in Phase A and D takes place in the same way as the collection of the primary endpoint in Phases B and C.

Quality of life SF-36

Quality of life is evaluated via the Short-Form 36 health questionnaire (SF-36), which contains 36 questions about physical and mental wellbeing over the past four weeks. The aggregate summary scales are evaluated in line with license requirements.

The scores and absolute changes in quality of life from baseline measured by the Short Form 36 (SF-36) per survey point are evaluated as secondary endpoints in Phases A-D.

Bodily function, disability due to low back pain (RMD)

Bodily function and disability due to low back pain is measured using the Roland Morris Disability Questionnaire (RMD) consisting of 24 questions about different activities. The scores and absolute changes from baseline in bodily function, as well as impairment due to low back pain assessed by the RMD per survey point are evaluated as secondary endpoints in Phase A.

Determination of psychological disorders (MINI)

The MINI International Neuropsychiatric Interview (MINI) (standard version) is conducted during Screening Visit A1 to investigate the presence of any of the 17 most common psychiatric disorders. This interview covers the therapeutic areas of behaviour and behavioural mechanisms, psychological disorders and chemically induced disorders, as well as the following therapeutic indications: Suicidal tendencies, psychotic disorders, anxiety disorders, depressive disorders, panic disorder, obsessive-

compulsive disorder, bulimia nervosa, anorexia nervosa, antisocial personality disorder, bipolar disorders, alcohol addiction/abuse, drug addiction/abuse, binge-eating disorders, generalised anxiety disorder, post-traumatic stress disorder. The interviewer, in this case the investigator, asks structured questions which can be answered with "yes" or "no". Patients who are suspected to have a current or past psychiatric disorder based on the MINI interview (at least one module completed) are not included in the study.

Determination of psychological disorder in the family history (1st degree relatives)

To investigate the presence of psychological disorders in the patient's first-degree relatives (parents and children), the patient is asked the following two questions during Screening Visit A1

"Are the following psychological disorders known currently/previously in a first-degree relative (parents and children): psychoses, schizophrenia, bipolar disorder, severe depression, anxiety disorder?".

Possible answers "yes"/"no"

"Has a first-degree relative (parents and children) committed suicide?"

Possible answers "yes"/"no"

Patients who answered "yes" to any of the questions will not be included in the study.

Addiction (ABC)

Possible dependence on the part of the patient as well as potential for abuse are determined using the Addiction Behaviour Checklist (ABC), which consists of 20 questions (Yes/No questions) about addictive behaviour between and during the visits. Three or more positive answers indicate possible substance abuse. The evaluation is carried out absolutely based on the number of questions answered in the affirmative according to Wu *et al* (2006).[62]

Diagnosis of substance dependence ICD-10

Possible dependence syndrome in accordance with ICD-10 (F12.2) is diagnosed by assessing six substance dependence criteria. Three or more positive responses over a period of 12 months define substance dependence.

Adverse events (AEs)

The occurrence of AEs is asked about daily in the patient diary through the following question:

[62] Wu, Stephen M., et al. "The addiction behaviors checklist: validation of a new clinician-based measure of inappropriate opioid use in chronic pain." *Journal of pain and symptom management* 32.4 (2006): 342-351.

"Have you experienced any new symptoms, illnesses, or injuries in the last 24 hours (which you did not experience at the start of the clinical study and were not documented by your doctor at Visit A1 as a previous/concomitant illness)? (possible responses: Yes/No)

If yes, "Please enter all events that occurred (symptoms, illnesses, injuries) on the welcome page under "Occurrence of events (symptoms, illnesses, injuries)". If you have used any additional medication and/or non-drug treatment for symptom(s), illness(es), or injury/injuries, please enter this on the welcome page under "Use of medications and/or non-pharmaceutical treatments".

Adverse events at the visits are documented by the investigator in the case report form.

Withdrawal symptoms (CWS)

Withdrawal symptoms are recorded in the patient diary daily during the wash-out phase of Study Phases C and D and during the treatment phase of Study Phase D using the Cannabis Withdrawal Scale (CWS) on an 11-point NRS scale on the basis of 19 questions about the intensity of signs of withdrawal and their negative impact on daily activity. The evaluation uses aggregate scores for intensity and functional disability, per Allsop *et al* (2011).[63]

[63] Allsop, David J., et al. "The Cannabis Withdrawal Scale development: patterns and predictors of cannabis withdrawal and distress." *Drug and alcohol dependence* 119.1-2 (2011): 123-129.

7.15.2 Overview of the safety and measurement parameters at individual visits

Table 11: Investigations during the clinical trial

**Phase A: Placebo-controlled treatment with VER-01 to demonstrate efficacy**					
**1. Collected daily in the diary by the patient**					
**Primary endpoint**	**Measurement instrument**	**Question(s)/description**	**Scale**	**Times surveyed**	**Processing duration**
**Pain score**	11-point Numerical Rating Scale (NRS)	"Click the number that describes your average pain over the last 12 hours (where 0 = "no pain" and 10 = "worst pain imaginable")	0 = no pain<br>to<br>10 = worst pain imaginable	• Daily in the morning<br>• Run-In, titration, treatment	< 1 min
**Secondary end-points**	**Measurement instrument**	**Question(s)/description**	**Scale**	**Times surveyed**	**Processing duration**
**Pain responders (30%)**	see primary endpoint			• Daily in the morning and evening<br>• Run-in, titration, treatment	< 1 min
**Pain responders (50%)**	see primary endpoint			• Daily in the morning and evening<br>• Run-in, titration, treatment	< 1 min
**Pain score**	Refer to primary endpoint			• Daily in the morning and the evening<br>• Run-in, titration, treatment period	< 1 min

**Sleep quality**	11-point NRS	"Click the number that states how greatly your pain impacted your sleep last night (where 0 = "no impact" and 10 = "completely impacted")"	0 = not impacted<br>to<br>10 = completely impacted	• Daily in the morning<br>• Run-in, titration, treatment	< 1 min
**Compliance**<br>**(IMP taken correctly)**	---	How many dose units you have taken this morning?"<br>"How many dosage units (n) of the investigational medicinal product have you taken this evening?"	---	• Daily in the morning and evening<br>• Titration, treatment	< 1 min
**Adverse events**	---	"Have you developed new symptoms, illnesses or injuries in the past 24 hours (that were not present before the study started and were documented as previous/concomitant illnesses by your investigator at Visit A1)?" (Possible responses: Yes/No)<br>If yes: "Please enter all events that occurred (symptoms, illnesses, injuries) on the welcome page under "Occurrence of events (symptoms, illnesses, injuries)". If you have taken additional medications and/or non-pharmaceutical therapies for pain, illness or an injury, please enter this on the welcome page under "Use of medications and/or non-pharmaceutical treatments".	---	On days on which adverse events arise	0-2 min

**Taking rescue medication and concomitant therapies**	---	"In the past 24 hours, have you taken rescue medication (ibuprofen or paracetamol) to treat your chronic non-specific lower back pain?" (Possible responses: Yes/No)<br>If yes: "Please select which:" (possible response: ibuprofen/paracetamol)<br>If Ibuprofen: "Number of Ibuprofen tablets (800 mg)" (input as free text)<br>"Have you also taken pantoprazole?" (Possible responses: Yes/No)<br>If yes: "Number of pantoprazole tablets (20 mg)" (input as free text)<br>If paracetamol: "Number of paracetamol tablets (500 mg)" (input as free text)<br><br>"In the past 24 hours, have you taken or used a new medication or has existing intake or use changed (e.g. in relation to dosage or frequency)? AND/OR In the past 24 hours, have you taken or used a new non-pharmaceutical treatment or have you changed your use of an existing non-	---	• Daily in the morning<br>• Run-in, titration, treatment	0-2 min

		pharmaceutical treatment (e.g. in relation to dosage or frequency)?" (Possible responses: Yes/No)<br>If yes: "Please make one or multiple new entries on the welcome page under "Use of medications and/or non-pharmaceutical treatments". In the event that existing medication or non-pharmaceutical treatment was changed, please enter the end date for the first entry afterwards."			

**2. Collected weekly in the diary by the patient**					
**Secondary endpoints**	**Measurement instrument**	**Question(s)/description**	**Scale**	**Times surveyed**	**Processing duration**
**Depression, anxiety and stress**	Depression anxiety stress scales (DASS)	Seven questions each about depression, anxiety and stress; evaluation refers to the past week	0 = did not apply to me at all<br>to<br>3 = affected me very greatly or most of the time	• Weekly in the morning<br>• Run-in, titration, treatment	4 min
**3. Collected during the visits by the investigator**					
**Secondary endpoints**	**Measurement instrument**	**Question(s)/description**	**Scale**	**Times surveyed**	**Processing duration**
**Satisfaction with the treatment result**	5-point Likert scale	"How satisfied are you with the treatment result for this patient?"	0 = very satisfied<br>to<br>4 = very unsatisfied	A6 (or last visit)	<1 min
**Safety and adverse reactions**	---	1) Onset of AEs/SAEs<br>2) Determining a complete blood count and clinical chemistry parameters using a full-blood sample<br>3) 24-hour Holter ECG (on 120 patients)<br>4) Physical examination (lungs, heart, skin, lymph nodes, CNS)<br>5) Vital signs (blood pressure, pulse, body temperature, body weight)<br>6) Study drop-out	---	1) A2, A3, A4, A5, A6<br>2) A1, A6<br>3) A2, Day 1 of the titration phase: from taking the 1st dose, A5<br>4) A1, A3, A6<br>5) A1, A2, A3, A4, A5, A6<br>6) A1, A2, A3, A4, A5, A6	1) 5 min<br>2) 5 min<br>3) 5-10 min<br>4) 15 min<br>5) 10 min<br>6) <1 min

**Taking rescue medication and concomitant therapies**	---	Check of the use of rescue medication and the blisters of rescue medication documented in the patient diary<br>Check of the use of concomitant treatments documented in the patient diary. Documentation of the adjuvant therapies by the investigator.	---	A2, A3, A4, A5, A6	2 min
**Compliance**<br>**(IMP taken correctly)**	---	Has the patient brought back VER-01 or placebo?<br>If yes, indicate the weight of the amber glass bottle(s)<br><br>- If no, specify the reason (product was disposed of by the patient, product was lost, other)	---	A3, A6 (or last visit)	2 min
**Substance dependence**	Addiction Behaviours Checklist (ABC)	20 questions about addiction behaviours between and during visits	Yes/No questions<br>≥3 positive answers indicate possible substance abuse	A3, A4, A5, A6	10 min

**4. Collected by the patient at visits**					
***Key-secondary* end-points**	**Measurement instrument**	**Question(s)/description**	**Scale**	**Times surveyed**	**Processing duration**
**Measurement of neuropathic pain**	Neuropathic Pain Symptom Inventory (NPSI)	12 questions about the differentiation and quantification of five different clinically relevant dimensions of neuropathic pain syndrome that are relevant to treatment. (spontaneous burning pain, spontaneous pressure pain, paroxysmal pain, evoked pain and paraesthesia/dysthaesia) per Bouhassira (2004).	0 = no perception of this pain<br>to<br>1 = very strong perception of this pain	A1, A2, A3, A4, A5, A6	5 min
**Secondary end-points**	**Measurement instrument**	**Question(s)/description**	**Scale**	**Times surveyed**	**Processing duration**
**Quality of life**	Short Form 36 (SF-36)	36 questions about physical and mental wellbeing over the past four weeks	---	A2, A3, A4, A5, A6	5 min
**Sleep quality**	Medical Outcomes Study Sleep Scale (MOS-SS)	12 questions about the assessment of sleep quality over the past four weeks	---	A2, A3, A4, A5, A6	5 min
**Bodily function, disability due to back pain**	Roland Morris Disability (RMD) Questionnaire	24 questions about different activities	---	A1, A2, A6	5 min
**Global assessment of symptoms by the patient**	Patient Global Impression of Change (PGIC), 7-point Likert scale	"How is your low back pain in comparison to before participation in the study?"	0 = very much better<br>to<br>6 = very much worse	A6	<1 min
**Satisfaction with the treatment result**	5-point Likert scale	"How satisfied are you with the treatment result?"	0 = very satisfied<br>to<br>4 = very unsatisfied	A6 (or last visit)	<1 min
**Satisfaction with tolerability**	5-point Likert scale	"How satisfied are you with the tolerability of the investigational medicinal product?"	0 = very satisfied<br>to<br>4 = very unsatisfied	A6 (or last visit)	<1 min

**Phase B: Long-term, open-label treatment with VER-01 to demonstrate long-term safety and to investigate the potential for dependency and abuse (period of 26 weeks)**					
**1. Collected by the investigator at visits**					
**Primary endpoint**	**Measurement instrument**	**Question(s)/description**	**Scale**	**Times surveyed**	**Processing duration**
**Safety and adverse reactions**	---	1) Occurrence of treatment-related AEs/SAEs	---	1) B7, AID1, B8, AID2, B9, AID3, AID4, B10	1) 5 min
**Secondary endpoints**	**Measurement instrument**	**Question(s)/description**	**Scale**	**Times surveyed**	**Processing duration**
**Safety and adverse reactions**	---	1) Determining a complete blood count and clinical chemistry parameters using a full-blood sample<br>2) Vital signs (blood pressure, pulse, body temperature, body weight)<br>3) Physical examination (lungs, heart, skin, lymph nodes, CNS)<br>4) Study drop-out	---	1) B7, B10<br>2) B7, B8, B9, B10<br>3) B10<br>4) B7, AID1, B8, AID2, B9, AID3, AID4, B10	2) 5 min<br>3) 10 min<br>4) 15 min<br>5) <1 min
**Taking rescue medication and concomitant therapies**	---	See Phase A	---	B7, B8, B9, B10	2 min
**Satisfaction with the treatment result**	5-point Likert scale	How satisfied are you with the treatment result for this patient?	0 = very satisfied<br>to<br>4 = very unsatisfied	B10<br>(or last visit)	<1 min
**Compliance**<br>**(IMP taken correctly)**	---	Has the patient brought back VER-01?<br>• If yes, indicate the weight of the amber glass bottle(s)<br>• If no, specify the reason (product was disposed of by the patient, product was lost, other)	---	B8, AID1, B9, AID2, B9, AID3, AID4, B10 (or last visit)	2 min

**Substance dependence**	Addiction Behaviours Checklist (ABC)	20 questions about addiction behaviours between and during visits	Yes/no questions<br>≥3 positive answers indicate possible substance abuse	B7, B8, B9, B10	10 min

**2. Collected daily in the diary by the patient**					
**Secondary endpoints**	**Measurement instrument**	**Question(s)/description**	**Scale**	**Times surveyed**	**Processing duration**
**Pain responders (30%)**<br>**Pain responders (50%)**<br>**Pain score**	11-point Numerical Rating Scale (NRS)	See section Phase A	0 = no pain<br>to<br>10 = most severe pain imaginable	Daily in the morning and evening during the titration and treatment phase	<1 min
**Sleep quality**	11-point NRS	See section Phase A	0 = not impacted<br>up to 10 = completely impacted	Daily in the morning during the titration and treatment phase	<1 min
**Taking rescue medication and concomitant therapies**	---	See section Phase A	---	Daily in the morning and evening during the titration and treatment phase	0-2 min
**Compliance (IMP taken correctly)**	---	See section Phase A	---	• Daily in the morning and evening<br>• Titration, treatment	<1 min

**Adverse events**	---	See section Phase A	---	On days on which adverse events arise	0-2 min
**3. Collected weekly by the patient in the diary**					
**Secondary end-points**	**Measurement instrument**	**Question(s)/description**	**Scale**	**Times surveyed**	**Processing duration**
**Depression, anxiety and stress**	Depression anxiety stress scales (DASS)	Seven questions each about depression, anxiety and stress; evaluation refers to the past week	0 = did not apply to me at all to<br>3 = affected me very greatly or most of the time	• Weekly in the morning<br>• Titration, treatment	4 min
**4. Collected by the patient during the visits**					
**Secondary end-points**	**Measurement instrument**	**Question(s)/description**	**Scale**	**Times surveyed**	**Processing duration**
**Quality of life**	Short Form 36 (SF-36)	36 questions about physical and mental wellbeing over the past four weeks	---	B10	5 min
**Measurement of neuropathic pain**	Neuropathic Pain Symptom Inventory (NPSI)	12 questions about the differentiation and quantification of five different clinically relevant dimensions of neuropathic pain syndrome that are relevant to treatment.	0 = no perception of this pain<br>to<br>1 = very strong perception of this pain	B7, B8, B9, B10	5 min
**Global assessment of symptoms**	Patient Global Impression of Change (PGIC), 7-point Likert scale	"How is your low back pain in comparison to before participation in the study?"	0 = very much better to<br>6 = very much worse	B10	<1 min
**Satisfaction with the treatment result**	5-point Likert scale	"How satisfied are you with the treatment result?"	0 = very satisfied<br>to<br>4 = very unsatisfied	B10 (or last visit)	<1 min

**Satisfaction with tolerability**	5-point Likert scale	"How satisfied are you with the tolerability of the investigational medicinal product?"	0 = very satisfied<br>to<br>4 = very unsatisfied	B10 (or last visit)	<1 min

**Phase C: Open-label, long-term treatment with VER-01 to demonstrate long-term safety, examine the potential for dependence and abuse as well as the effects of sudden drug withdrawal**<br>**(Analysis along with the endpoints recorded in Phase B; period of 52 weeks)**					
**1. Collected by the investigator at visits**					
**Primary endpoint**	**Measurement instrument**	**Question(s)/description**	**Scale**	**Times surveyed**	**Processing duration**
**Safety and adverse reactions**	---	Occurrence of treatment-related AEs/SAEs	---	AID5, C11, AID6, C12, AID7, AID8, C13, C14	5 min
**Secondary endpoints**	**Measurement instrument**	**Question(s)/description**	**Scale**	**Times surveyed**	**Processing duration**
**Safety and adverse reactions**	---	1) Determining a complete blood count and clinical chemistry parameters using a full-blood sample<br>2) Vital signs (blood pressure, pulse, body temperature, body weight)<br>3) Physical examination (lungs, heart, skin, lymph nodes, CNS)<br>4) Study drop-out	---	1) C13<br>2) C11, C12, C13, C14<br>3) C13, C14<br>4) AID5, C11, AID6, C12, AID7, AID8, C13, C14	1) 5 min<br>2) 10 min<br>3) 15 min<br>4) <1 min
**Intake of rescue medication and concomitant therapies**	---	See section Phase A	---	C11, C12, C13, C14	2 min
**Satisfaction with the treatment result**	5-point Likert scale	"How satisfied are you with the treatment result for this patient?"	0 = very satisfied<br>to<br>4 = very unsatisfied	C13<br>(or last visit)	<1 min

**Compliance (IMP taken correctly)**	---	Has the patient brought back VER-01?<br>• If yes, indicate the weight of the amber glass bottle(s)<br>• If no, specify the reason (product was disposed of by the patient, product was lost, other)	---	AID5, C11, AID6, C12, AID7, AID8, C13 (or last visit)	2 min
**Substance dependence**	Addiction Behaviours Checklist (ABC)	20 questions about addiction behaviours between and during visits	Yes/no questions<br>≥3 positive answers indicate possible substance abuse	C11, C12, C13	10 min
**Diagnosis of substance dependence**	International Statistical Classification of Diseases and Related Health Problems (ICD-10)	6 diagnostic criteria to define substance dependence	≥3 positive answers diagnose substance dependence	C13	15 min

**2. Collected daily in the diary by the patient**					
**Secondary endpoints**	**Measurement instrument**	**Question(s)/description**	**Scale**	**Times surveyed**	**Processing duration**
**Pain responders (30%)**	11-point Numerical Rating Scale (NRS)	See section Phase A	0 = no pain<br>to<br>10 = most severe pain imaginable	Daily in the morning and evening during the treatment and wash-out phase	<1 min
**Pain responders (50%)**					
**Pain score**					
**Sleep quality**	11-point NRS	See section Phase A	0 = not impacted<br>up to 10 = completely impacted	Daily in the morning during the treatment and wash-out phase	<1 min

**Intake of rescue medication and concomitant therapies**	---	See section Phase A	---	Daily in the morning during the treatment and wash-out phase	0-2 min
**Withdrawal symptoms**	Cannabis Withdrawal Scale (CWS)	19 questions about the intensity of signs of withdrawal and their negative impact on daily activity	0 = not at all/no negative impact<br>to<br>10 = extreme/complete impact	Daily in the morning during the wash-out phase	5-10 min
**Compliance (IMP taken correctly)**	---	See section Phase A	---	Daily in the morning and the evening during the treatment phase	<1 min
**Adverse events**	---	See section Phase A	---	On days on which adverse events arise	0-2 min
**3. Collected weekly in the diary by the patient**					
**Secondary endpoints**	**Measurement instrument**	**Question(s)/description**	**Scale**	**Times surveyed**	**Processing duration**
**Depression, anxiety and stress**	Depression anxiety stress scales (DASS)	Seven questions each about depression, anxiety, and stress; evaluation refers to the past week	0 = did not apply to me at all<br>to<br>3 = affected me very greatly or most of the time	• Weekly in the morning<br>• Treatment, wash-out	4 min
**4. Collected during the visits by the patient**					

Secondary endpoints	Measurement instrument	Question(s)/description	Scale	Times surveyed	Processing duration
**Quality of life**	Short Form 36 (SF-36)	36 questions about physical and mental wellbeing over the past four weeks	---	C13	5 min
**Measurement of neuropathic pain**	Neuropathic Pain Symptom Inventory (NPSI)	12 questions about the differentiation and quantification of five different clinically relevant dimensions of neuropathic pain syndrome that are relevant to treatment.	0 = no perception of this pain<br>to<br>1 = very strong perception of this pain	C11, C12, C13, C14	5 min
**Global assessment of symptoms**	Patient Global Impression of Change (PGIC), 7-point Likert scale	"How is your low back pain in comparison to before participation in the study?"	0 = very much better to<br>6 = very much worse	C13	<1 min
**Satisfaction with the treatment result**	5-point Likert scale	"How satisfied are you with the treatment result?"	0 = very satisfied<br>to<br>4 = very unsatisfied	C13<br>(or last visit)	<1 min
**Satisfaction with tolerability**	5-point Likert scale	"How satisfied are you with the tolerability of the investigational medicinal product?"	0 = very satisfied<br>to<br>4 = very unsatisfied	C13<br>(or last visit)	<1 min

**Phase D: Double-blind, placebo-controlled treatment with VER-01 to demonstrate the maintenance of efficacy, examination of the potential for dependence and abuse as well as the effects of sudden drug withdrawal**					
**1. Collected daily in the diary by the patient and at the visits by the investigator**					
**Primary endpoint**	**Measurement instrument**	**Question(s)/description**	**Scale**	**Times surveyed**	**Processing duration**
**Time to treatment failure: Number of days from randomisation R2 until the 1st day of treatment failure**	--	Study drop-out	--	D11	<1 min

	11-point NRS	"Click the number that describes your average pain over the last 12 hours (where 0 = "no pain" and 10 = "worst pain imaginable")	0 = no pain<br>to<br>10 = worst pain imaginable	Daily in the morning during the treatment phase	<1 min
**Secondary endpoints**	**Measurement instrument**	**Question(s)/description**	**Scale**	**Times surveyed**	**Processing duration**
**Proportion of patients with treatment failure**	see primary endpoint				
**2. Collected daily in the diary by the patient**					
**Secondary endpoints**	**Measurement instrument**	**Question(s)/description**	**Scale**	**Times surveyed**	**Processing duration**
**Pain score**	11-point NRS	See primary endpoint	0 = no pain<br>to<br>10 = worst pain imaginable	• Daily in the morning and evening<br>• Treatment and wash-out	<1 min
**Sleep quality**	11-point NRS	See section Phase A	0 = not impacted<br>to<br>10 = completely impacted	• Daily in the morning<br>• Treatment and wash-out	<1 min
**Taking rescue medication and concomitant therapies**	---	See section Phase A	---	• Daily in the morning<br>• Treatment and wash-out	0-2 min
**Compliance (IMP taken correctly)**	---	See section Phase A	---	Daily in the morning and the evening during the treatment phase	<1 min

**Adverse events**	---	See section Phase A	---	On days on which adverse events arise	0-2 min
**Withdrawal symptoms**	Cannabis Withdrawal Scale (CWS)	19 questions about the intensity of signs of withdrawal and their negative impact on daily activity	0 = not at all/no negative impact<br>to<br>10 = extreme/complete impact	Daily in the morning during the treatment phase and the wash-out phase	5-10 min
**3. Collected weekly in the diary by the patient**					
**Secondary endpoints**	**Measurement instrument**	**Question(s)/description**	**Scale**	**Times surveyed**	**Processing duration**
**Depression, anxiety and stress**	Depression anxiety stress scales (DASS)	Seven questions each about depression, anxiety, and stress; evaluation refers to the past week	0 = did not apply to me at all<br>to<br>3 = affected me very greatly or most of the time	• Weekly in the morning<br>• Treatment, wash-out	4 min
**4. Collected during the visits by the investigator**					
**Secondary endpoints**	**Measurement instrument**	**Question(s)/description**	**Scale**	**Times surveyed**	**Processing duration**
**Satisfaction with the treatment result**	5-point Likert scale	How satisfied are you with the treatment result for this patient?	0 = very satisfied<br>to<br>4 = very unsatisfied	D11 (or last visit)	<1 min
**Safety and adverse reactions**	---	1) Onset of AEs/SAEs<br>2) Vital signs (blood pressure, pulse, body temperature, body weight)<br>3) Physical examination (lungs, heart, skin, lymph nodes, CNS)<br>4) Study drop-out	---	1) D11, D12<br>2) D11, D12<br><br>3) D11, D12<br>4) D11, D12	1) 5 min<br>2) 10 min<br><br>3) 15 min<br>4) <1 min

**Taking rescue medication and concomitant therapies**	---	See section Phase A	---	D11, D12	2 min
**Compliance**<br>**(IMP taken correctly)**	---	Has the patient brought back VER-01 or placebo?<br>• If yes, indicate the weight of the amber glass bottle(s)<br><br>- If no, specify the reason (product was disposed of by the patient, product was lost, other)	---	D11 (or last visit)	2 min
**Substance dependence**	Addiction Behaviours Checklist (ABC)	20 questions about addiction behaviours between and during visits	Yes/no questions<br>≥3 positive answers indicate possible substance abuse	D11	10 min
**Diagnosis of substance dependence**	International Statistical Classification of Diseases and Related Health Problems (ICD-10)	6 diagnostic criteria to define substance dependence	≥3 positive answers diagnose substance dependence	D11	15 min

5. Collected by the patient at visits					
**Secondary endpoints**	**Measurement instrument**	**Question(s)/description**	**Scale**	**Times surveyed**	**Processing duration**
**Quality of life**	Short Form 36 (SF-36)	36 questions about physical and mental wellbeing over the past four weeks	---	D11	5 min
**Measurement of neuropathic pain**	Neuropathic Pain Symptom Inventory (NPSI)	12 questions about the differentiation and quantification of five different clinically relevant dimensions of neuropathic pain syndrome that are relevant to treatment.	0 = no perception of this pain<br>to<br>1 = very strong perception of this pain	D11, D12	5 min
**Global assessment of symptoms by the patient**	Patient Global Impression of Change (PGIC), 7-point Likert scale	"How is your low back pain in comparison to before participation in the study?"	0 = very much better<br>to<br>6 = very much worse	D11	<1 min
**Satisfaction with the treatment result**	5-point Likert scale	"How satisfied are you with the treatment result?"	0 = very satisfied<br>to<br>4 = very unsatisfied	D11 (or last visit)	<1 min
**Satisfaction with tolerability**	5-point Likert scale	"How satisfied are you with the tolerability of the investigational medicinal product?"	0 = very satisfied<br>to<br>4 = very unsatisfied	D11 (or last visit)	<1 min

7.15.3 Suitability of measurement methods

Phase A (evidence of efficacy):

The primary efficacy endpoint is defined as the change in average pain intensity in the morning versus baseline (study Week -1) on an 11-point NRS. The measurement method in the study described here to demonstrate this recommended pain reduction after treatment with VER-01 is based on the information collected from patients each day on an 11-point scale via questionnaires. This is a common approach in the assessment of pain treatments and generates reliable data to clearly define pain reduction. Patients answering a brief questionnaire each day does not represent excessive burden for them and can be considered appropriate.

In addition, ECG measurements are taken in Phase A in approx. 120 patients (3x), which may cause inconvenience but are considered acceptable due to the low frequency.

Phase B and Phase C (investigation of long-term safety):

To study the long-term safety of VER-01, all adverse and serious adverse events that occur are documented and monitored. In addition, regular physical examinations (3x), measurements of vital signs (8x) and blood tests (3x) are performed to identify negative effects due to treatment with VER-01. Additionally, the number of study drop-outs is continuously monitored. This comprehensive clinical data will be sufficient to make a clear statement about the long-term safety of the IMP. At the same time, the burden for participating patients is low, even considering the chronic pain they are suffering from, since they have already received a non-drug therapy and non-opioid analgesics for the treatment of their CLBP without sufficient pain relief. Only the blood draw leads to discomfort, but due to the low frequency of these procedures, they are considered reasonable.

Phase D (evidence of maintained efficacy):

In accordance with the EMA guideline, patients who have a satisfactory response to at least six months of open-label treatment (30% responders) should be enrolled to investigate maintained efficacy. These patients should be investigated for maintained efficacy in a randomised withdrawal study. These requirements are reflected in Phase D of the clinical trial described here, in which patients are randomised to receive VER-01 or placebo over four weeks following a prior 26 weeks of open-label treatment (Phase B).

It can therefore be assumed that sufficient data can be collected for a reliable assessment of maintained efficacy. For patients in the placebo group, this approach can mean a deterioration in their pain, as they will be taken off successful treatment. Yet, this approach is considered reasonable due to the

short period of a maximum of four weeks. Furthermore, patients will be provided with rescue medication and they have the option to drop out of the study at any time.

Secondary objectives:

Validated questionnaires and recognised measurement instruments, which have been used in numerous studies investigating pain patients, will be used to record secondary objectives.

7.16 Ensuring data quality

7.16.1 Monitoring

The sponsor guarantees continuous monitoring before, during, and after the clinical trial. The objective of monitoring is to check that the rights and safety of trial subjects, the validity, verifiability, and completeness of the study data are ensured and protected, and that the study is being conducted in accordance with the protocol, GCP, and applicable legal regulations.

Before the start of the clinical trial, all aspects such as the protocol, the CRFs and documentation are discussed with the investigator and their employees and training is provided. At each monitoring appointment, the facilities, storage of the IMPs, CRFs and all other study-specific documentation is inspected. Monitoring takes place on a risk-adapted and centralised basis.

All study sites and investigators declare their consent to visits by the monitor to the study site at regular intervals and provide appropriate assistance for these visits. This is arranged in contracts governing the conduct of the study with the study sites and the responsible investigator. The monitor is given the right to compare the eCRFs with the original documents (health forms, ECG, lab printouts etc.). The study sites and the investigators shall allow the monitor direct access to all necessary documents for the monitoring of the specific trial, as well as the sponsor as part of sponsor-accompanied visits. The point and purpose of these monitoring visits are as follows:

- Inspection of the informed consent forms
- Checking of patient safety (occurrence and documentation/reporting of AEs and SAEs)
- Checking that the eCRFs are accurate and complete
- Validation of eCRFs compared to original data (Source Data Verification, SDV)
- Checking handling of the IMPs
- Evaluation of the progress of the trial and the rate of recruitment
- Checking compliance with the protocol
- GCP-compliant conduct of the study at the study site

- Discussing the conduct of the study and any deficiencies identified with the investigator

A report is generated for each visit, which documents the progress of the clinical trial and all difficulties identified (e.g., refused inspection). Monitoring is performed according to the GCP guideline as published in the current version and the monitoring plan for this study.

7.16.2 Audits/inspections

The sponsor has the right to perform audits at the study site and other facilities involved in the study for the purpose of quality assurance. The aim of audits is to check the validity, verifiability, and completeness of the data and the reliability of the clinical trial as well as to check that patient rights and patient safety are guaranteed. For this purpose, the sponsor may bring in people who are otherwise not involved in the clinical trial (auditors). These individuals shall be permitted to inspect all trial-related documents (specifically: protocol, CRFs, patient files, IMP documentation, trial-related correspondence).

The sponsor and all participating study sites commit to cooperate during inspections by relevant authorities and in this respect to grant access to original documents to the authorised persons. All auditors commit to handle personal data and other data resulting from the study documents confidentially.

7.17 Documentation

Investigator Site File (ISF):

Each investigator receives an ISF that explains their obligations during the clinical trial. Among others, the folder contains the following documents:

- Protocol with annexes
- Investigator's brochure (IB)
- "Patient Information and Informed Consent Form for participation in the clinical trial" form in duplicate
- Other documents and forms for the investigator

In addition, the investigator receives:

- Access data to the eCRF, used to document all study-related data

All trial-related data is recorded in the electronic CRFs promptly by the responsible investigator. Where permitted by law, the documentation can be delegated to other members of the trial team. The CRFs are signed electronically by the investigator or deputy personally.

Electronic case report forms (eCRF):

The external service provider commissioned by the sponsor creates an electronic CRF, in which a study case report form can be created for each patient, into which all study-relevant information of the study participant is entered. All medical and non-medical members of the trial group involved in the study, with access authorisation, receive individual access data. The eCRF has an audit trail, meaning it is possible to trace at any time when the entries were made and by which user. With their electronic signature, the investigator or deputy confirms the correctness of all entries in the audit trail. The CRFs are checked by a monitor and the scope of the inspection is recorded in a monitoring report. The documentation procedure is also carried out for patients who leave the clinical study prematurely but for whom a CRF has been prepared. Forms completed by patients and the electronic patient diaries serve as source data and are also part of the CRF. The location of the corresponding source data is defined in the Source Data Location List (SDLL).

The investigator ensures that the information in the eCRF is correct and complete.

Deviations from the protocol:

All protocol deviations that the investigator, monitor, or data manager becomes aware of are reported to the sponsor, who evaluates them and, in the case of severe protocol deviations, will introduce corrective action. Severe protocol deviations are protocol deviations that could significantly impact the safety and integrity of a participating patient or the scientific power of the study.

7.17.1 Data management

All surveys conducted by the investigator during the visit must be documented primarily in the respective patient records, which are defined as source data. In addition, all questionnaires completed by the investigator and/or patient during the visit are classified as source data. Case report forms for documentation will be provided by the sponsor. In the event that the investigator uses an electronic data system without an audit trail for patient records, signed and dated printouts of the electronic patient records will be required on a regular basis. By signing and dating the printouts, the investigator confirms that the printouts and the originals (electronic patient records) match and that each printout covers the entire period since the last printout.

Furthermore, the following documents are considered source data: electronic patient diaries, ECG report printouts and laboratory reports.

The investigator and the study site allow study-related monitoring, sponsor-accompanied visits at monitoring, audits, institutional review board/independent ethics committee review, and regulatory inspections and grant direct access to source data/documents. By signing the written informed consent form, each study participant provides consent for source data verification (SDV).

The data is transmitted by the sites in an eCRF and checked for completeness and plausibility by the monitor in the study sites. The verification is carried out by means of cross-checking based on the available source data and the electronic case report forms. The monitor is required to inform the investigator about errors and missing data. The sponsor and persons and service providers authorised by the sponsor have access with read-only rights to the eCRF or access that allows queries to be made. During the monitoring visit or remotely (via access to the eCRF), the monitors check the plausibility of the entries and, after source data verification (SDV) and the signature of the investigator or deputy, release the data to data management for data correction and subsequent data evaluation.

Authorised data managers, the monitors, as well as persons authorised by the sponsor can submit data correction requests (queries) using their access to the eCRF.

In addition, the data in the eCRF is checked by programmed plausibility checks.

The patient diary is made available to patients in electronic form. Only the questionnaires performed during the visits (NPSI, SF-36, MOSS-SS, RMD, painDETECT, ABC, ICD-10 substance dependence questionnaire, assessment of treatment results and tolerability by the patient, global assessment of symptoms by the patient, assessment of treatment results by the investigator and MINI) are documented on paper and transferred from the sites to the eCRF, and the monitor performs the SDV. Further details are regulated in the data management plan.

In the event of technical problems with the electronic patient diary that make it impossible for the patient to make the entries in the electronic diary, a paper version of the patient diary can be used, and will be provided by the Sponsor as a backup.

7.17.2 Archiving

The protocol, the documentation, the arrangements made between investigators and the sponsor, and all other documents created in connection with the clinical trial must be stored for at least 15 years after the completion or early termination of the clinical trial, or for at least two years after the last marketing authorisation in the European Union (if no approval applications are being processed or inspected), or for at least two years after the formal completion of the clinical development of the investigational medicinal product. This applies regardless of the format in which the documents were created, e.g. electronic or paper-based (EU Directive 2001/83).

The sponsor, study site, and investigator will take all necessary measures to prevent accidental or premature destruction. Documents concerning pseudonymisation must be stored by the study site for at least fifteen years after the conclusion or termination of the clinical trial.

Should Regulation EU 536/2014 come into force during the term of the study and apply to this study, the above-mentioned retention periods will increase to 25 years.

The sponsor must always be informed before documents are destroyed after the storage periods expire.

8. Safety

8.1 Definitions of adverse events

The terms adverse event, serious adverse event, adverse drug reaction, serious adverse drug reaction, unexpected adverse drug reaction, and suspected unexpected serious adverse reaction are defined below.

8.1.1 Adverse event

An adverse event (AE) is any negative medical incident that affects a person who is participating in a clinical trial. The adverse event is not necessarily in a causal relationship with taking the investigational medicinal product or study-specific procedures. Adverse events include changes in condition, subjective and objective disease symptoms, concomitant illnesses, or accidents. Incidents that are not in a causal relationship with taking the IMP are evaluated as AEs if they occur during the clinical trial. In addition, the exacerbation of a disease or other changes are evaluated as AEs if these events occur due to stopping, abusing, or overdosing the IMP.

Diseases, clinical signs, and symptoms or pathological changes in blood parameters detected at Visit A1 (e.g., lab result deviations that are expected for the underlying illness) are not evaluated as AEs if they are detected again at later visits, unless they deteriorate or the frequency increases. An exception to the documentation requirement in the eCRF for these AEs does not release the investigator from their professional documentation duties.

The onset of a new pregnancy during this clinical trial is also regarded an adverse event. For reporting a new pregnancy, see Chapter 8.4.

8.1.2 Serious adverse event or serious adverse drug reaction

A serious adverse event (SAE) or serious adverse drug reaction (SADR) is any adverse event or adverse reaction that

- is fatal or life-threatening
- makes inpatient treatment or an extension to inpatient treatment necessary
- leads to lasting or serious disability
- results in a congenital abnormality or birth defect
- as well as any other event that meets a comparable criterion (at the discretion of the assessing investigator)

Events are considered life-threatening (in the above sense) if there was a risk of dying at the time of the event. A hospital admission is any inpatient admission of a participating patient that comprises at least one night (12am to 6am). Hospital stays planned before the first administration of the IMP do not count as serious adverse events, but must be documented in the patient file and eCRF.

If an adverse event is considered serious, the event will be documented on a separate SAE form in addition to the AE documentation. SAEs are subject to a legally prescribed reporting obligation (see Chapter 8.3).

8.1.3 Adverse drug reactions

An adverse drug reaction (ADR) is any disadvantageous and unforeseen reaction to an IMP, regardless of its dose. In this respect, 'reaction' refers to an 'event' that is at least possibly associated with the administration of the study product.

8.1.4 Unexpected adverse drug reaction

An unexpected adverse reaction is an adverse reaction, the type, frequency or severity of which does not correspond with the available information about the IMP. The expected adverse reactions are listed in the Reference Safety Information of the Investigator's Brochure.

8.1.5 Suspected unexpected serious adverse reaction

A suspected unexpected adverse reaction (SUSAR) is an adverse reaction, the type or severity of which does not correspond with the available information about the IMP, is assessed as serious, and for which a connection with the study product is estimated as at least possible.

8.2 Documentation and follow-up of adverse events

The sponsor shall adequately inform and brief all people involved in the treatment of participating patients about their responsibilities with respect to adverse events. All adverse events from the start of the study (visit A1) through to the end of the clinical trial are recorded in the eCRF. The occurrence of adverse events is asked about daily in the patient diary, checked by the investigator at each visit, and evaluated to see whether a causal relationship can be established with the IMP or the clinical trial procedures. In addition, the study site must also check the information in the patient diary between the visits, in particular regarding the documentation of SAEs in the diary.

If an adverse event occurs, the patient in question must be observed, regardless of the causal relationship between the AE and the IMP, until the symptoms have subsided, pathological laboratory parameters have returned to baseline, or there is a plausible explanation for the adverse event until the

death of the participating patient or until the clinical trial has finished for the patient in question. The follow-up period lasts 14 days after stopping treatment with the IMP.

8.2.1 Documentation of adverse events

All adverse events are recorded in the eCRF with the following information:

- Date and time of the start and end
- Intensity
- Expected or unexpected
- Outcome
- Connection with the IMP or the clinical trial
- Serious or non-serious
- Pausing or suspending administration of the IMP or other measures taken

8.2.2 Intensity of the adverse event

The investigator will classify the adverse events that occur based on their intensity as follows:

- Mild: Clinical symptom or sign that is well tolerated
- Moderate: Clinical symptom or sign that impairs normal activity
- Severe: Clinical symptom or sign that leads to severe disability, to incapacity for work or inability to perform everyday tasks

8.2.3 Connection between adverse events and the investigational medicinal product or the clinical trial

For each adverse event, the investigator will evaluate causality with the IMP or the clinical trial procedures. The type and pattern of the reaction, the temporal relationship with the administration of the IMP, the patient's clinical status, the concomitant medication, and other relevant clinical parameters are considered. If the event occurred as a result of lack of efficacy or based on the underlying illness, it is evaluated as not related.

In the double-blind Phases A and D of this study, the causality evaluation by the investigator acts as though the patient had received VER-01, provided that immediate emergency unblinding is not necessary for medical reasons.

The following classifications are defined for the causality of the adverse event with the IMP or the clinical trial:

- Definite: An event that follows a clear chronological process after use of the IMP or an event due to the clinical trial (e.g., blood draw) or in which the concentration of the product in body tissue or fluids is measured, follows a known or expected response pattern to the IMP, disappears after stopping the medication or reducing the dose, and returns if exposure resumes
- Likely: An event that follows a clear chronological process after use of the IMP or an event due to the clinical trial, follows a known or expected response pattern to the suspected IMP and disappears after stopping the medication or reducing the dose, and cannot be explained by the known characteristics of the patient's clinical condition
- Possibly related: An event that follows a clear chronological process after use of the IMP or an event due to the clinical trial, follows a known or expected response pattern to the suspected IMP, but which could also be easily triggered by a series of other factors
- Unlikely: An event for which sufficient information exists to conclude that there is no connection with the IMP or the clinical trial
- Not assessed: An event that was reported as an adverse event, but for which the connection with the study product has not yet been evaluated at the time of the notification because further data is required or is currently being collected
- Unassessable: It is not possible to estimate the connection

An adverse drug reaction is suspected if the causal relationship is estimated as at least "possible" or as "unassessable" or "not assessed". Events that are classified as "unlikely" do not count as suspected adverse reactions.

8.3 Notification of suspected unexpected serious adverse reactions and serious adverse events

Suspected unexpected serious adverse reactions are reported in accordance with the national law of the country in question. Regardless of the expected causal relationship, all serious adverse events over the course of the clinical trial must be documented in the relevant part of the eCRF by the investigator and reported on a separate SAE form to the CRO for Pharmacovigilance within 24 hours. The CRO for Pharmacovigilance will perform a second evaluation of the SAEs and, if they are classified as SUSARs, will inform the relevant authorities and institutional review boards/independent ethics committees. A graphical representation of the reporting is displayed in Figure 7.

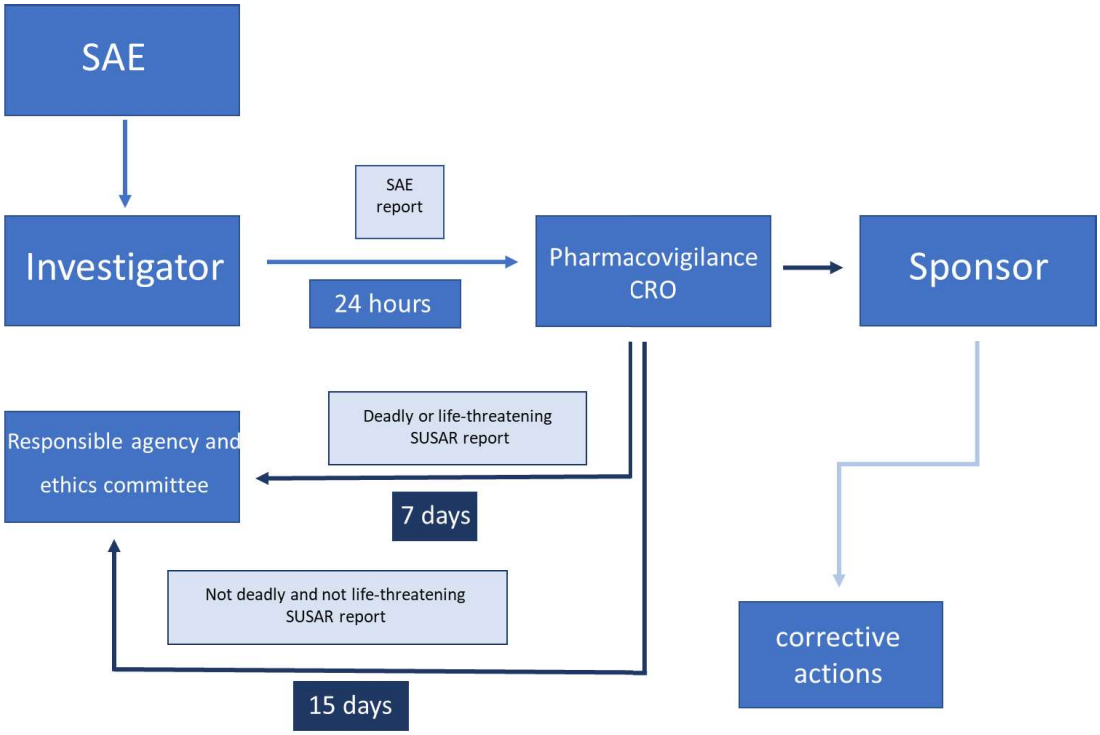

Figure 7: Graphic representation of the reporting of SAEs

8.4 Notification of a pregnancy

Any pregnancy must be documented on a separate pregnancy form and reported to the CRO for Pharmacovigilance within 72 hours of becoming aware of it. This applies for both male and female patients. Any pregnancy will be documented on a separate pregnancy form. A separate informed consent form for pregnant women is required for following up on the outcome of the pregnancy. Pregnancies are not regarded as SAEs, provided that the mother and fetus/child are not endangered.

8.5 Investigator's report to the CRO for Pharmacovigilance

All SAEs arising during or until 14 days after stopping taking/using the IMP must be reported by the investigator to the CRO for Pharmacovigilance immediately (within 24 hours) after becoming aware of it, via fax or email. The sponsor will provide the investigator with a corresponding SAE reporting form. The report is made to the CRO for Pharmacovigilance:

Winicker Norimed GmbH

Dr. Petra Hofmann

E-Mail: safety@winicker-norimed.com

Fax: 0049 911 92680 4444

Details about the investigator's reporting obligations can be found in a guideline in the ISF.

All SAEs of which the investigator becomes aware after the end of the study and which he suspects to be causally related to the investigational medicinal product must be reported by the investigator to the sponsor by e-mail or fax immediately (within 24 hours) after becoming aware of them:

Email: studienkoordination@vertanical.com

Fax: 0049 (0)89 62829142

8.6 Second review by the CRO for Pharmacovigilance

All serious adverse events (SAEs) are reviewed independently of the investigator's opinion in consultation with the CRO for Pharmacovigilance with regard to severity (see Chapter 8.2.2), causality (see Chapter 8.2.3) and expectability (see Chapter 8.1.4).

8.7 Unblinding in relation to a patient

Routine unblinding does not take place if SAEs arise, but only to help prevent risks for patients. In the case of suspected unexpected adverse reactions (SUSARs), unblinding of the patient takes place to verify causality before a report is made to the institutional review board/independent ethics committee and the responsible national competent authority. This means that unblinding and a re-evaluation of potential SUSARs is performed on this basis in order to decide whether the event actually has a causal relationship with the IMP and therefore can be classified as a SUSAR.

The processes for evaluating causality and for unblinding are described in the ISF. Unblinding takes place as described in Chapter 7.14.

8.8 Reporting to institutional review boards/independent ethics committees and responsible federal authorities

During the clinical trial, any suspected unexpected adverse reaction (SUSAR) that becomes known is reported to the responsible national competent authority and the institutional review board/independent ethics committee by the CRO for Pharmacovigilance.

Fatal or life-threatening SUSARs:

The responsible national competent authority and the central ethics committee will be informed by the CRO for Pharmacovigilance of all fatal or life threatening SUSARs. This takes place immediately, but by no later than seven calendar days after becoming aware of it. In any case, further relevant information is transmitted to the responsible national competent authority and the central ethics commit-

tee within a maximum of a further eight days. In the event of the death of an individual, the institutional review board/independent ethics committee in whose area of jurisdiction the death occurred will also be informed.

Non-fatal and non-life threatening SUSARs:

The responsible national competent authority and the central ethics committee will be informed by the CRO for Pharmacovigilance of all non-fatal and non-life threatening SUSARs by no later than fifteen calendar days after the sponsor becomes aware of them. Additional relevant information will be communicated as soon as possible. If information is incomplete at the time of notification, additional information for the purpose of adequate analysis is requested from the notifier or other available sources.

8.9 Corrective measures

The CRO for Pharmacovigilance shall report any matter that requires another review of the risk/benefit assessment of the IMP immediately, by no later than fifteen days after becoming aware of the matter, to the involved ethics committee and the responsible authorities of other member states in the European Union and other countries that are Contracting Parties of the Agreement on the European Economic Area, in whose territory the clinical trial is conducted. These include in particular:

- Isolated case reports of expected serious adverse reactions with an unexpected outcome
- An increase in the frequency of expected, serious, clinically relevant adverse reactions
- Suspected unexpected serious adverse reactions in patients after their participation in the clinical trial
- Events in connection with the conduct of the study or the development of the IMP that can possibly impact the safety of the person concerned

8.10 Investigator information about SUSARs

The CRO for Pharmacovigilance informs investigators of all SUSARs for the IMP, including further relevant information by the deadlines applicable for the responsible national competent authority.

If new information becomes known that differs from the scientific information provided, the CRO for Pharmacovigilance shall inform all investigators of this.

8.11 Annual report about the safety of trial subjects

Once per year, the sponsor's external pharmacovigilance service provider submits a trial subject safety report, which considers all relevant information available about the safety of patients during the reporting period, to the responsible federal authorities and the responsible authorities of other member states in the European Union and other countries that are Contracting Parties of the Agreement on the European Economic Area, in whose territory the clinical trial is conducted. The report is also submitted to the central ethics committee.

The annual safety report is drafted in accordance with the specifications of the ICH Guideline E2F "Development Safety Update Report – DSUR". The reporting period for the annual safety report begins upon the day of initial approval by the responsible national competent authority of a country in which the clinical trial is conducted. This date serves every year as the data lock point for the data to be included in the annual safety report. The sponsor shall submit these annual safety reports within 60 days after this lock point.

8.12 Risk management

8.12.1 Anticipated/foreseeable clinical benefits

The anticipated clinical benefit of VER-01 is a safe and tolerable pain reduction with low adverse reactions in patients with chronic non-specific low back pain in which drug treatment is indicated and previous optimised treatments with non-opioids have not led to sufficient pain relief or are unsuitable due to contraindications or intolerance.

Currently, the only available option for the long-term drug treatment of these patients are opioid analgesics. In consideration of the safety and side effects profile of opioids, the risks and benefits should be weighed up carefully before prescribing opioids. The high potential for abuse and the risk of physical and psychological addiction limit the long-term administration of opioids.

Due to expected lower adverse reactions and negligible potential for dependence and abuse, VER-01 is a more tolerable and safer drug treatment alternative for long-term treatment and provides a clear clinical benefit for patients.

8.12.2 Safety aspects when taking the investigational medicinal product

There is already comprehensive data about the oral administration of THC and high-THC extracts from clinical human and animal studies (performed with VER-01 and literature-based). Frequent side effects of THC and THC-rich extracts are:

- Changes to pulse rate and blood pressure and the resulting dizziness and fainting spells during the titration phase
- Potentially elevated risk of psychosis, depression, and other concomitant mental illnesses
- Short-term effects on the central nervous system, such as sleepiness, cognitive and motor restrictions, dizzy spells, balance and attention problems, dysarthria, dysgeusia, lethargy, memory problems, anxiety, illusions, mood swings and paranoia
- Potentially gastrointestinal adverse reactions, such as constipation, diarrhea, dry mouth, nausea, vomiting
- Metabolic disorders, such as increased appetite, anorexia
- Temporary sleep, emotional or appetite disturbances in the case of sudden withdrawal
- Potential impairment of spermatogenesis
-

The assessment of the likelihood of events occurring when taking VER-01 takes place according to the Reference Safety Information of the Investigator's Brochure. Further safety information regarding the use of VER-01 and any safety aspects to be considered can be found in Chapter 6.3.5 Safety and 7.3 Guidance for the Investigator of the currently valid version of the Investigator's Brochure.

8.12.3 Expected interactions with other pharmaceutical products

Orally administered THC undergoes hepatic metabolism by cytochrome P450 (CYP) oxidases, enzymes known to play an important role in the metabolism of many drugs. CYP3A4 and CYP2C9 catalyze the oxidation of THC to the psychoactive metabolite 11-hydroxy-THC (11-OH-THC), which is further oxidized to the inactive metabolite 11-carboxy-THC (THC-COOH).

In vitro studies with human hepatocytes show no evidence of activation or inhibition of CYP enzymes by THC at clinically relevant doses (up to 314 ng/ml THC) and in clinical studies, interactions between products containing THC (Sativex) and inhibitors and inducers of the major human cytochrome P-450 isoforms generally show a low risk of clinically significant drug interactions with oral administration.

The effect of rifampicin (CYP3A4 inducer), ketoconazole (CYP3A4 inhibitor) and omeprazole (CYP2C19 inhibitor) on the pharmacokinetic parameters of THC was in the range of high inter- and intra-individual variance normally observed.

Nevertheless, co-administration of VER-01 with CY2C19 inhibitors may result in a decrease in mean Cmax and mean AUC for THC and 11-OH-THC. Further, administration of a CYP3A4 inhibitor could increase mean Cmax and AUC for THC and 11-OH-THC. Accordingly, the daily dose of VER-01 may need

to be increased or decreased in order to balance the efficacy of the compound against potential adverse effects that may occur at higher doses. Therefore, a review of the dosing regimen is advised when starting or stopping the therapy with a CYP3A4 inhibitor or CYP3A4 inducer.

Furthermore, the investigator must inform patients that the use of CYP3A4 and CYP2C9 inhibitors, substrates, or inducers may lead to prolongation of the effect and possible side effects of the investigational medicinal product.

Furthermore the following pharmacodynamic interactions must be taken into account:

- Extended duration of action of pentobarbital and ether narcotics
- Antagonistic effect of amphetamines
- Increase in physostigmine toxicity
- Increase in the sedative effect of sedatives and sleeping pills
- Synergetic effect with gabapentin in terms of the reduction of allodynia in the case of neuropathic pain
- Potential interactions with tricyclic antidepressants with tachycardia, high blood pressure or mood swings
- Interactions with adrenalin, atropine, beta blockers and diuretics with tachycardia, orthostatic blood pressure, as well as an increase in blood pressure as a result

8.12.4 Risks in association with participation in the clinical trial

8.12.4.1 Risk due to blood draw

Measures: The investigator shall ensure that the blood draw ideally takes place while lying down and by trained staff.

Risk assessment: In rare cases, there is a residual risk of pain when inserting the needle, mild bleeding with subsequent bruising, which generally disappears within a few days, circulatory reactions, infection, thrombosis or injury to the surrounding tissue and nerves due to the needle.

Evaluation of residual risk: Reasonable, as the benefits of the blood draw to analyse blood values and safety monitoring of the patient outweigh the possible risks described.

8.12.4.2 Risk of accidents on the way to and from the study site

Measures: The investigator shall ensure that the patient’s condition is sufficiently stable to avoid accidents due to circulatory causes on the way home.

Risk assessment: There is a risk for the patient of accidents on the way to and from the study site. Traffic insurance is therefore taken out for all study subjects. Patients are informed in detail about the fact insurance has been taken out and the action to take in the event of an insurance claim in the patient information. In addition, patients are told that they should always assess their ability to drive themselves.

Evaluation of residual risk: Reasonable, as an accident on the way to or from the study site is unlikely.

8.12.4.3 Risk due to measurements with the ECG device

Measure: The investigator shall ensure that the ECG device is attached properly.

Risk assessment: The examination is practically risk-free. In rare cases, an itchy rash can develop in the area of the electrodes.

Evaluation of residual risk: Reasonable, as the benefits of measuring the QT interval and safety monitoring of the patient outweigh the possible risks described.

8.12.4.4 Risk associated with inclusion of elderly patients (without an upper age limit)

No specific studies on elderly patients are available, but other studies have included patients up to 90 years of age. In general, the pharmacokinetics and pharmacodynamics of medications may be altered in the elderly due to age-related physiological changes, multiple comorbidities, or use of other medications, leading to increased susceptibility to central nervous side effects. Side effects such as dizziness, feeling drunk, and impaired concentration may increase the risk of falls and injuries (e.g., when preparing food and hot drinks). Accordingly, special caution should be taken when including elderly patients, especially if they have problems performing daily activities such as preparing hot meals and drinks.

Measure: In elderly patients, special attention should be paid to personal safety, such as the preparation of hot meals and drinks. Whether the patient is suitable for participation in the study is up to the assessment of the investigator.

Risk assessment: There is a small residual risk of falls and injuries. Due to the slow titration at the beginning of the intake and the determination of the patient-specific optimal dose, the risk of excessive side effects is reasonable.

Evaluation of residual risk: Reasonable because the benefit of study participation by elderly patients outweighs the residual risk of falls and injuries.

8.12.5 Risk minimisation measures

The responsible investigator instructs the patients in the use of the IMP. In addition, information about using the IMP safely can be found in the patient information. To ensure that initial adverse reactions are minimised, the IMP is taken at a low dose of 2.5 mg per day when taken the first time at the beginning of the clinical trial and the dose is only increased gradually to the patient-specific effective dose.

8.12.6 Risk/benefit justification

VER-01 should only be used in this study for patients with chronic non-specific low back pain when drug treatment is indicated and a previous optimised treatment with non-opioids has not led to sufficient pain relief or was unsuitable due to contraindications or intolerance. For these patients, opioids are currently the only long-term treatment option available, although there are many limitations in the use of opioids and some guidelines even advise against their use in patients with non-specific chronic low back pain. There is therefore a major clinical need for a new treatment option. Products containing THC could be safer and more tolerable alternatives to opioids.

Overall, there are no major safety concerns, and in consideration of the possible risks (see Chapter 8.12.2, 8.12.3 and 8.12.4) as well as measures to minimise risks (see Chapter 8.12.5) and available treatment options, the potential advantages of VER-01 clearly outweigh these risks (see Chapter 8.12.1).

9. Statistical methods

9.1 General considerations

Details of the data evaluation are specified in a statistical analysis plan (SAP). This first version of the SAP focuses on the definition of statistical concepts and statistical analyses for Phase A of this study including all data collected for Phase A. The SAP will be updated and extended later to also include all details of the statistical analysis for Phase B, Phase C, and Phase D of this study. The SAP will be finalized prior to each database lock and prior to unblinding, if applicable. Changes to the planned statistical analysis must be specified in the final version of the SAP relevant for the respective study phase the latest.

After all patients randomised in Phase A and Phase D have completed the respective phase or have prematurely ended the study, a final review of the study data assessed in a blinded process (blind review data) is performed. This Blind Data Review also comprises all data from Phase B for patients who have completed or discontinued Phase B at this time. The review of the blinded data includes the discussion of all documented protocol deviations and the assessment and identification of intercurrent events. Further intercurrent events can be defined at the latest during the blind review, including the associated analysis strategy. This is the last point in time at which changes to the statistical analysis plan are possible. Finally, the associated blind review report is created. Unblinding will only be performed after the blind review report has been finalised and approved. The finalisation of the statistical analysis plan and the blind review report as well as the subsequent unblinding of the study database are formally documented.

A second review (Data Review) takes place after all patients have completed the study.

The analysis is carried out separately for each study phase, summarised in a report after each (Blind) Data Review. All data collected until the end of the respective phase are included in the analysis of each study phase.

Data are analysed using appropriate descriptive statistics. Depending on the scaling of the data, either sample statistics (e.g., number of observations (n), number of missing values, mean, standard deviation, minimum, median, maximum, lower and upper quartile) or frequency tables (with absolute and relative frequencies) are used. Analyses across study phases are defined separately for selected variables in the statistical analysis plan. Furthermore, whenever possible, the data analysis is additionally stratified by treatment arm and can be further differentiated (e.g., by visit, time point/frame of data collection, country or study site). All individual patient data will also be listed.

All p-values in the context of the exploratory analysis serve exclusively a descriptive and not a confirmatory interpretation. There is no adjustment for multiple testing. All p-values are calculated two-sided unless stated otherwise.

9.2 Determination of sample size

The necessary sample size was determined considering the following aspects:

- sufficient statistical power for the analysis of the primary efficacy endpoint in Phase A (change from baseline in pain intensity measured on an 11-point NRS)
- sufficient statistical power for the analysis of the key-secondary efficacy endpoint in Phase A (change from baseline in the NPSI total score for patients with a painDETECT score > 18)
- sufficient sample size to investigate long-term safety for at least six months in Phase B and for at least 12 months in Phase C
- sufficient statistical power to analyse the primary efficacy endpoint in Phase D (time to treatment failure).

The necessary sample size was determined separately for each aspect. The necessary sample size for the whole study corresponds to the maximum of the four individual sample sizes.

In consideration of these aspects and the multi-phase study design, 808 patients need to be randomised for this study. The patients are randomly assigned in a 1:1 ratio to one of the two treatment groups (VER-01 or placebo) at the beginning of Phase A (Section 7.13.1). The primary and the key-secondary efficacy endpoint are tested hierarchically. Therefore, the type I error does not need to be adjusted for multiple testing.

The sample size was not adjusted for expected drop-outs, since the data of patients who discontinued early from the study are considered in the statistical analysis according to the defined strategy via imputation procedures.

9.2.1 Determination of sample size for the primary efficacy endpoint in Phase A

The primary efficacy endpoint in Phase A is the change from baseline in mean pain intensity measured on an 11-point NRS (0-10 points).

To show superiority of treatment with VER-01 vs. placebo on a two-sided significance level of 5% and with 90% statistical power, 732 patients need to be randomly assigned in a 1:1 ratio to one of the two treatment groups in Phase A (VER-01 vs. placebo). With 366 evaluable patients in each treatment group, a mean change from baseline in pain intensity can be detected on the 11-point NRS that is 0.6

points lower for patients treated with VER-01 compared to patients treated with placebo. Additionally, a standard deviation of 2.5 points was assumed.

The sample size was calculated using SAS V9.4 *proc power*.

Similar effect sizes and standard deviations were identified based on a review of placebo-controlled clinical studies for patients suffering from chronic low back pain who were treated with cannabinoids [Whiting et al. (2015) [64], Nurmikko et al. (2007)[65]] or non-cannabinoids [Skljarevski et al. (2010)[66], Skljarevski et al. (2012)[67], Buynak et al. (2010)[68], Konno et al. (2016)[69]].

9.2.2 Determination of sample size for the key-secondary efficacy endpoint in Phase A

The key-secondary efficacy endpoint in Phase A is the change from baseline in the NPSI total score (0 - 1 points) for patients with a painDETECT score > 18.

To show superiority of treatment with VER-01 vs. placebo on a two-sided significance level of 5% and with 80% statistical power, 180 patients with a painDETECT score > 18 should be randomly assigned in a 1:1 ratio to one of the two treatment groups in Phase A (VER-01 vs. placebo). Due to the proportion of randomised patients with a painDETECT score of >18 (22.2%), the total necessary sample size is 808 patients.

With 90 evaluable patients per treatment group, a 0.105-point lower mean change from baseline in the NPSI total score of patients treated with VER-01 compared to patients treated with placebo can be detected. Additionally, a standard deviation of 0.25 points was assumed.

The sample size was calculated using SAS V9.4 *proc power*.

[64] Whiting, Penny F., et al. "Cannabinoids for medical use: a systematic review and meta-analysis." Jama 313.24 (2015): 2456-2473.

[65] Nurmikko, Turo J., et al. "Sativex successfully treats neuropathic pain characterised by allodynia: a randomised, double-blind, placebo-controlled clinical trial." Pain® 133.1-3 (2007): 210-220.

[66] Skljarevski, Vladimir, et al. "Efficacy and safety of duloxetine in patients with chronic low back pain." Spine 35.13 (2010): E578-E585.

[67] Skljarevski, Vladimir, et al. "Efficacy and safety of duloxetine in patients with chronic low back pain who used versus did not use concomitant nonsteroidal anti-inflammatory drugs or acetaminophen: A post hoc pooled analysis of 2 randomized, placebo-controlled trials." *Pain research and treatment* 2012 (2012).

[68] Buynak, Robert, et al. "Efficacy and safety of tapentadol extended release for the management of chronic low back pain: results of a prospective, randomized, double-blind, placebo-and active-controlled Phase III study." *Expert opinion on pharmacotherapy* 11.11 (2010): 1787-1804.

[69] Konno, Shinichi, et al. "Randomized, double-blind, placebo-controlled phase III trial of duloxetine monotherapy in Japanese patients with chronic low back pain." *Spine* 41.22 (2016): 1709.

Only a few clinical studies are published in the literature on the indication of chronic pain which analyse the NPSI total score [NPSI for patient with CLBP: Baron et al. (2015)[70], Baron et al. (2016)[71], Steigerwald et al. (2012)[72], Galvez et al. (2012)[73]; NPSI for patients with diabetic neuropathy: Soin et al. (2018)[74]]. Based on the available literature for opioids, the chosen effect size and standard deviation are plausible. A study of Überall et al. /2022)[75] however, indicates that the clearly greater therapy effect can be achieved with cannabis-based products. Overall, an estimation of the effect size is complicated by the fact that none of the referenced studies are placebo-controlled.

9.2.3 Determination of sample size for investigation of long-term safety of at least six months in Phase B and 12 months in Phase C

No formal sample size calculation was performed for study Phases B and C. However, the ICH E1 guideline requires the collection of safety data of at least 300 patients who were treated for six months and of 100 patients who were treated for 12 months. To fulfil this requirement, at least 300 patients need to complete Phase B and at least 100 patients need to complete Phase C. This requirement also has an impact on the total necessary sample size due to the multi-phase study design and due to expected drop-outs during Phases A and B.

On the assumption that 808 patients are randomised for Phase A and that approx. 25% of the patients discontinue early from study prior to Phase B (either early discontinuation from study in Phase A or no available informed consent for Phase B), approx. 606 patients complete Phase A. Assuming a drop-out rate of 40% during long-term phases B and C, 500 patients should be enrolled in Phase B so that 300 patients complete Phase B. This sample size is sufficient to meet the requirement of available long-

[70] Baron, Ralf, et al. "Effectiveness and safety of tapentadol prolonged release (PR) versus a combination of tapentadol PR and pregabalin for the management of severe, chronic low back pain with a neuropathic component: A randomized, double-blind, Phase 3b study." *Pain Practice* 15.5 (2015): 455-470.

[71] Baron, Ralf, et al. "Effectiveness of tapentadol prolonged release (PR) compared with oxycodone/naloxone PR for the management of severe chronic low back pain with a neuropathic component: a randomized, controlled, open-label, phase 3b/4 study." *Pain Practice* 16.5 (2016): 580-599.

[72] Steigerwald, Ilona, et al. "Effectiveness and safety of tapentadol prolonged release for severe, chronic low back pain with or without a neuropathic pain component: results of an open-label, phase 3b study." *Current medical research and opinion* 28.6 (2012): 911-936.

[73] Gálvez, Rafael, et al. "Tapentadol prolonged release versus strong opioids for severe, chronic low back pain: results of an open-label, phase 3b study." *Advances in therapy* 30.3 (2013): 229-259.

[74] Soin, Amol, et al. "A randomized, double-blind study of the effects of a sustained release formulation of sodium nitrite (SR-nitrite) on patients with diabetic neuropathy." *Pain physician* 21.2 (2018): 179-190.

[75] Ueberall Michael et al. “Effectiveness, safety, and tolerability of Nabiximols Oromusocal spray vs. typical oral long-acting opioid analgesics in patients with severe neuropathic back pain: Analysis of 6-month real-world data from Germany Pain e-Registry.” *Pain medicine* 23.4 (2022): 745-760.

term safety data for at least six months. Out of these 300 patients, a minimum of 150 patients are planned to be included in Phase C to collect sufficient long-term safety data over at least 12 months.

9.2.4 Determination of sample size for the efficacy endpoint in phase D

The primary endpoint to show maintenance of efficacy in Phase D is the time from randomisation to Phase D until treatment failure. Only patients who achieve clinical response at the end of Phase B are included in Phase D.

According to the assumptions in Section 9.2.3 it is expected that at least 300 patients will complete Phase B. Assuming that 45% of the patients achieve clinical response at the end of Phase B, 135 patients are available for inclusion in Phase D. Assuming identical drop-out rates for patients with a painDETECT score > 18, approx. 30 patients have a painDETECT Score > 18 (22.2% of patients randomised in Phase D).

No direct comparative data is available for Phase D. The clinical study by Langford et al. [76] comes closest, in which the effect of THC/CBD spray on central neuropathic pain was examined in patients with multiple sclerosis. In this study, a randomised placebo-controlled study phase is followed by a 14-week open label treatment period with subsequent randomised withdrawal for four weeks. Finally, treatment failure was documented for 24% of the patients treated with THC/CBD and for 57% of the patients treated with placebo.

Assuming that a similar result could be achieved, the results for sample size calculation were rounded conservatively to 25% treatment failures for VER-01 and 55% treatment failures after switching to placebo.

For the log-rank test comparing the two (exponential) survival curves with regards to time to treatment failure, a sample size of 78 (39 per treatment group) is required to achieve 80% statistical power under a two-sided significance level of 5%. Therefore, at least 80 patients need to be included in Phase D.

The sample size was calculated using the software PASS V16.

In case that more patients are available for inclusion, recruitment is stopped after 120 patients have been included .

[76] Langford RM, Marces J, Novotna A et al. A double-blind, randomized, placebo-controlled, parallel-group study of THC/CBD oromucosal spray in combination with the existing treatment regimen, in the relief of central neuropathic pain in patients with multiple sclerosis. J Neurol. 2013;260: 984-97.

9.3 Analysis of patient disposition

The number of screen failures (including the documented reasons, see Section 5.5, the number of randomised patients and the number of patients who were included in the study (who signed informed consent), discontinued the study (including documented reasons) or completed the study is summarised. The number of patients who prematurely discontinue the study medication are summarised and the details about intake of study medication are listed per patient. Intercurrent events are summarised and listed per patient. Study inclusion and protocol deviations are summarised and listed per patient.

The analysis of patient disposition is carried out separately for each study phase based on the defined study populations.

9.4 Analysis of demographics and baseline characteristics

All demographics and other baseline characteristics, including medical history and prior treatments and surgeries, are analysed descriptively. If several measurements of a variable were taken before the first intake of study medication or before randomisation (whichever is applicable), the last measured value is used as baseline value.

Demographics and baseline characteristics are analysed separately for each study phase based on the defined study populations.

9.5 Definition of intercurrent events

Intercurrent events were anticipated and defined considering the indication CLBP and the study design (Table 12). Intercurrent events occur by definition after randomisation or after the start of treatment. How these intercurrent events are handled for the statistical analysis is described in the framework of defining estimands.

Table 4: Definition of intercurrent events

Intercurrent Event	Phase	Definition
Early discontinuation from study due to intolerability	Phase A & D	Early discontinuation from study with documented reason "Adverse event". Relationship to study medication of the adverse event was judged to be at least "possibly related" or "unassessable"/"not assessed" by the investigator.
Early discontinuation from study due to lack of efficacy	Phase A & D	Early discontinuation from study with documented reason "*Lack of Efficacy"*.
Early discontinuation from study due to other reasons	Phase A & D	Early discontinuation from study due to all other reasons, i.e. all documented reasons other than *Intolerability* (i.e., documented reason is "Adverse event" related to study medication) or *Lack of Efficacy.*<br>In phase D, the reason for discontinuation *Non-compliance with investigational product* is handled separately and in accordance with the relevant

		intercurrent event “Non-ignorable non-compliance with dosing of study medication”.<br><br>Reasons for early discontinuation from study will be documented precisely in the eCRF. In case of multiple reasons for early discontinuation from study, adverse events related to intolerability and “*Lack of Efficacy”* need to be documented primarily.
Overdosage of allowed rescue medication (Section 7.7)	Phase A	A distinction is made between ignorable, non-ignorable and irrelevant overdosage. A non-ignorable overdosage is considered as an indication of ineffectiveness of study medication. An ignorable overdosage is not considered as an indication of ineffectiveness of study medication but the data collected after overdosage cannot be interpreted meaningfully. Irrelevant overdosage has no effect on the interpretation of collected data.<br><br>Definitions for diary-based endpoints collected on a daily basis (NRS):<br>Non-ignorable overdosage:<br>Exceeding the allowed maximum daily dose on at least four out of seven days (within one study week) in Weeks 8-15 or intake of rescue medication on at least five out of seven days (within one study week) independently of the daily dose in Weeks 8-15. This criterion is suspended in the titration phase and within the first four weeks of treatment (Weeks 1-7).<br>This exceedance has to be documented for at least three study weeks.<br>Ignorable overdosage:<br>Every exceedance of the allowed maximum daily dose within Week 15 or intake of rescue medication on at least five out of seven days in Week 15 which is not considered a non-ignorable overdosage.<br>Irrelevant overdosage:<br>Any other overdosage within Phase A is considered irrelevant.<br><br>Definitions for visit-based secondary endpoints:<br>Non-ignorable overdosage:<br>Exceeding the allowed maximum daily dose on at least four out of seven days (within one study week) in Weeks 8-15 or intake of rescue medication on at least five out of seven days (within one study week) independently of the daily dose in Weeks 8-15. This criterion is suspended in the titration phase and within the first four weeks of treatment (Weeks 1-7). This exceedance has to be documented for at least three study weeks.<br>Ignorable overdosage:<br>Every exceedance of the allowed maximum daily dose on the day when Visit A6 is performed or the day before which is not considered as non-ignorable overdosage.<br>Irrelevant overdosage:<br>Any other overdosage within Phase A is considered irrelevant.
Overdosage of allowed rescue medication (Section 7.7)	Phase D	Definitions for diary-based endpoints collected on a daily basis (NRS):<br><br>Non-ignorable overdosage:<br>Exceeding the allowed maximum daily dose within Phase D is considered as an indication of treatment failure and is considered a non-ignorable overdosage. An intake of rescue medication within the allowed maximum daily dose on at least five out of seven days within one study week is also

		considered as an indication for treatment failure. The treatment failure is documented for the fifth day of intake within the respective study week.
Intake of prohibited concomitant medication	Phase A	Patients taking prohibited concomitant medication should not be excluded from the study but must be continuously monitored. A non-ignorable intake of prohibited concomitant medication is an indication of ineffectiveness of the study medication. An ignorable intake of prohibited concomitant medication is no indication of ineffectiveness of study medication; however, data collected after intake cannot be interpreted as usual.<br>The documented indication for the intake of the prohibited concomitant medication is reviewed with respect to relationship to the indication CLBP within a manual medical review to classify ignorable or non-ignorable intake.<br><br>Non-ignorable intake:<br>Any intake of non-opioid or opioid analgesics or adjuvant analgesics with relationship to CLBP within or after Week 8 (Week 8 inclusive).<br><br>Ignorable intake:<br>Any intake of non-opioid or opioid analgesics or adjuvant analgesics without relationship to CLBP<br>- which occur within the relevant seven days including washout time for the assessments of the NRS in Week 15 for diary-based endpoints.<br>- which occur on the day of Visit A6 for the primary analysis or on the day before including washout time for visit-based endpoints.<br><br>Irrelevant intake without relationship to CLBP:<br>Any intake of non-opioid or opioid analgesics or adjuvant analgesics without relationship to CLBP which do not occur in the time frames defined above or any intake of non-opioid or opioid analgesics or adjuvant analgesics with relationship to CLBP in Week 1-7.
Intake of prohibited concomitant medication	Phase D	Non-ignorable intake:<br>Any intake of non-opioid or opioid analgesics or adjuvant analgesics with relationship to CLBP is an indication of treatment failure and is considered a non-ignorable intake.<br>Ignorable intake:<br>Any intake of non-opioid or opioid analgesics or adjuvant analgesics without relationship to CLBP is not an indication of treatment failure; however, data collected after intake cannot be interpreted as usual. The intake is considered an ignorable intake.
Start/adaptation of non-drug pain therapy	Phase A & D	Start/adaptation of a relevant non-drug pain therapy (see Table 6) was documented in the eCRF and the adaptation was judged as a relevant modification compared to baseline by the investigator.<br>Start or relevant adaptation of a non-drug pain therapy is no indication of non-effectiveness of study medication but data collected after the start/relevant adaptation cannot be interpreted meaningfully. Such

		starts/relevant adaptations of non-drug pain therapies are considered ignorable.<br>The adaptations of non-drug pain therapies which were not judged as relevant modifications compared to baseline by the investigator display no intercurrent events.
Non-compliance with dosage of study medication	Phase A	Ignorable non-compliance with dosage of verum:<br>Exceeding the maximum daily dose and/or the maximum individual dose on at least 4 out of 7 days within one study week starting from and including Week 8 is considered as an ignorable non-compliance.<br>Stopping the intake of verum within the last 14 days of Phase A (Week 14 and 15) is also considered as an ignorable non-compliance.<br><br>Irrelevant non-compliance with dosage of verum:<br>Every other exceedance of the maximum verum dose or any other interruption of the intake of verum is considered irrelevant.<br><br>Irrelevant non-compliance with dosage of placebo:<br>Exceeding the maximum placebo dose, or stopping or interrupting the intake of placebo is considered irrelevant.
Non-compliance with dosage of study medication	Phase D	Non-ignorable non-compliance with dosage of study medication:<br>Early discontinuation of study medication (either early discontinuation from study with documented reason “Non-compliance with study drug” or no intake of study medication is documented in the diary until the end of Phase D) is considered non-ignorable non-compliance.<br><br>Irrelevant non-compliance with dosage of study medication:<br>Exceeding the maximum dose of study medication is considered irrelevant non-compliance.

9.6 Phase A: Analysis of randomised parallel group comparison

The analysis of the randomised, placebo-controlled study phase to prove the efficacy and tolerability of VER-01 is carried out after completion of Phases A and D.

9.6.1 Study populations

The following study populations are used for the analysis.

Full Analysis Set Phase A (FAS_A):

The analysis data set used for efficacy is the Full Analysis Set (FAS_A). This data set contains all randomised patients who took at least one dose of the study medication in Phase A. The patients are assigned to the treatment group to which they were originally randomised.

Full Analysis Set Phase A for patients with neuropathic pain component (FAS_A_NE):

The assessment dataset is used for selected efficacy analyses and contains all patients of the FAS_A having a painDETECT score >18 at the screening visit.

Safety Analysis Set Phase A (SAF_A):

The Safety Analysis Set (SAF_A) used for the safety and tolerability analysis contains all randomised patients who took at least one dose of the study medication in Phase A. The patients are assigned to the respective treatment group according to the treatment actually taken.

9.6.2 Estimand of the primary endpoint in Phase A

The primary endpoint is the change from baseline in mean pain intensity measured in the morning on the 11-point NRS (defined in Section 7.15.1.1). The analysis of the primary endpoint is described by the estimand defined in Table 13, with the anticipated intercurrent events being taken from Table 12. According to the ICH E9 Addendum[77], an estimand consists of these five attributes: population, treatment, variable, population-level summary, and intercurrent event. A precise definition of the term *intercurrent event* is given in the ICH E9 addendum. The *de facto* estimand defined below corresponds to the behaviour of a patient that is usually expected in clinical practice.

Table 13: Estimand of the primary endpoint in Phase A

Attribute	Definition
**Population**	Patients with non-specific chronic low back pain (CLBP) of at least moderate intensity defined by the inclusion and exclusion criteria for Phase A, who were randomised to either treatment with VER-01 or placebo and who took at least one dose of study medication (FAS_A, as described in Section 9.6.1).
**Treatment**	VER-01 vs. placebo,<br>each including allowed rescue medication as well as irrelevant overdosage of allowed rescue medication, irrelevant overdosage of study medication and irrelevant intake of prohibited concomitant medication (see Table 12).
**Variable**	Change from baseline in mean pain intensity measured in the morning on the 11-point NRS. The difference in the patient-individual mean values of Week 15 (NRS_{W15}) and baseline (NRS_{base}; based on study week -1) is:<br>$CHG_{\square} = NRS_{W15} - NRS_{base}$
**Population-level summary**	Using $MCHG_{VER}$ = Mean change from baseline in the VER-01 arm,<br>and $MCHG_{Pl}$ = Mean change from baseline in the placebo arm, the two-sided null hypothesis H_0 is tested on the 5% level:<br>$H_0: MCHG_{VER} = MCHG_{Pl} \quad vs. \quad H_1: MCHG_{VER} \neq MCHG_{Pl}.$

[77] ICH Harmonised Guideline: Addendum on estimands and sensitivity analysis in clinical trials to the guideline on statistical principles for clinical trials. Nov. 2019.

	Superiority of VER-01 is shown if the null hypothesis can be rejected and the patients treated with VER-01 have on average a larger negative change (= improvement) compared to baseline, i.e. $MCHG_{VER} < MCHG_{Pl}$.<br>The confirmatory hypothesis is tested with an ANCOVA (analysis of covariance) model, which includes the variable defined above as response variable and the treatment group as the main effect as well as various baseline characteristics (neuropathic pain component, baseline NRS pain intensity, age, gender and country) as covariables.
**Intercurrent events**	The following strategies are used for the intercurrent events specified in Table 12. If imputations are used (details in Section 9.6.7), the strategy is the corresponding hypothetical scenario that results from the assumptions on which the imputation models are based. This means that under the Missing Not At Random (MNAR) assumption, the hypothetical scenario is considered that all patients behave like placebo patients. On the other hand, with the Missing at Random (MAR) assumption, the hypothetical scenario is considered that the patients behave like comparable patients from the same treatment group.<br><br><u>Early discontinuation from study due to intolerability</u><br>• Imputation under MNAR assumption: Jump to Reference (J2R).<br><u>Early discontinuation from study due to lack of efficacy</u><br>• Imputation under MNAR assumption: J2R.<br><u>Early discontinuation from study due to other reasons</u><br>• Imputation under MAR assumption: Multiple Imputation (MI).<br><u>Overdosage of allowed rescue medication</u><br>• Ignorable overdosage: Multiple Imputation under MAR assumption, MI.<br>• Non-ignorable overdosage: Imputation under MNAR assumption, J2R.<br>• Irrelevant overdosage: Using the measured values under the Treatment Policy Strategy.<br><u>Intake of prohibited concomitant medication</u><br>• Ignorable intake: Multiple Imputation under MAR assumption, MI.<br>• Non-ignorable intake: Imputation under MNAR assumption, J2R.<br>• Irrelevant intake: Using the measured values under the Treatment Policy Strategy.<br><u>Start/adaptation of non-drug pain therapy</u><br>• Ignorable start/adaptation: Multiple Imputation under MAR assumption, MI.<br><u>Non-compliance with dosage of study medication</u><br>• Ignorable non-compliance: Multiple Imputation under MAR assumption, MI.

	• Irrelevant non-compliance: Using the measured values under the Treatment Policy Strategy.

Sensitivity analyses

More conservative imputation methods will be specified in the SAP as sensitivity analyses or supportive estimands for the non-verifiable MAR / MNAR assumptions, e.g.

- Baseline observation carried forward (BOCF) instead of J2R
- Imputation of early discontinuation from study due to all reasons with J2R and last observation carried forward (LOCF)

or alternatively a gradual deterioration (in the sense of conservative adjustment) of the values using delta adjustment of the MI models (Tipping Point Analysis). The same sensitivity analyses are used for the secondary estimands.

Details are specified in the SAP.

9.6.3 Estimands of secondary endpoints Phase A

9.6.3.1 Key-secondary estimand: NPSI

As described in Section 7.15.1.2, the key-secondary endpoint of Phase A is the calculated change from baseline in Neuropathic Pain Symptom Inventory (NPSI) total score at Visit A6. This estimand is part of the confirmatory analysis and is therefore taken into account when determining the sample size (see Section 9.2.2). No adjustment of the significance level of 5% is necessary for this test, which is subordinate to the primary analysis, following the hierarchical testing principle. If the result of the statistical test of the primary analysis is not significant then the statistical test of the secondary analysis becomes immediately irrelevant for the confirmatory decision making.

The estimand for the NPSI total score can be defined as a de facto estimand in the same way as to the primary estimand, i.e. the estimated treatment effect should correspond to the treatment effect expected in clinical practice. The main differences are in the attributes population and variable; details are specified in Table 14.

Table 5: Key-secondary estimand of NPSI total score

Attribute	Definition
**Population**	Patients with non-specific chronic low back pain (CLBP) of at least moderate intensity defined by the inclusion and exclusion criteria for Phase A, who were randomised to either treatment with VER-01 or placebo, who took at least one dose of study medication, and who

	also stated a painDETECT score > 18 during screening (FAS_A_NE, as described in Section 9.6.1.).
**Treatment**	Identical to the primary estimand of Phase A.
**Variable**	Change from baseline in NPSI total score at Visit A6. The difference in the patient-individual NPSI total score at Visit A6 ($NPSI_{A6}$) and the NPSI total score at baseline ($NPSI_{base}$; collected at Visit A2) is: $$CHG = NPSI_{A6} - NPSI_{base}$$
**Population-level summary**	The confirmatory hypothesis, being defined in the same way as the primary estimand of Phase A (see Table 13), is tested with an ANCOVA (analysis of covariance) model, which includes the variable defined above as the response variable and the treatment group as the main effect as well as various baseline characteristics (baseline NPSI total score, age, gender and country) as covariables.
**Intercurrent event**	The strategies for dealing with intercurrent events are analogous to the primary estimand of Phase A. It should be noted, however, that the definitions of the intercurrent events "overdosage of allowed rescue medication" and "intake of prohibited concomitant medication" differ between the two endpoints, since the primary endpoint is diary-based and the key-secondary endpoint is visit-based (see Table 12).

9.6.3.2 Derived secondary estimands: Pain intensity (NRS)

An estimand is specified for the following secondary efficacy endpoints of Phase A:

Change from the baseline in pain intensity, measured in the morning on an 11-point scale for patients with a painDETECT score >18,

Change from baseline in pain intensity measured in the morning and evening on the 11-point NRS,

Pain responders (30%) in the morning as well as in the morning and in the evening on average,

Pain responders (50%) in the morning as well as in the morning and in the evening on average.

Both of the first mentioned can be defined and evaluated analogously to the primary estimand without further explanation. Only the attribute population needs to be adjusted and limited to patients with a painDETECT score >18 for the first secondary estimand. Only the attribute *variable* needs to be adjusted for the second secondary estimand, since the calculation of the patient-specific change from baseline is here based on observations from the morning and the evening. For this purpose, separate averages of all available morning and evening values are first calculated, and then the average value of the two calculated average values is determined.

Estimand of responder endpoints

The estimand of pain responders (30%/50%) in the morning as well as in the morning and in the evening is described in Table 15

Table 15: Secondary estimand of pain responders (30%/50%)

Attribute	Definition
**Population**	Analogous to primary estimand of phase A
**Treatment**	Analogous to primary estimand of phase A
**Variable**	Pain responder (30%/50%) in the morning (and in the morning and evening) is defined as described in Section 7.15.1.1.<br>The patient-individual mean values at baseline (NRS_{base}; based on study week -1) are subtracted from mean values of Week 15 (NRS_{W15}) and set in relation to baseline:<br>$$RCHG_{\square} = (NRS_{W15} - NRS_{base})/NRS_{base},$$<br>Patients are defined as 30% pain responders when $RCHG_{\square} \leq -0.3$.<br>Analogously, patients are defined as 50% pain responders when $RCHG_{\square} \leq -0.5$.
**Population-level summary**	The expected probability of a pain responder is analysed with a logistic regression model for the binary response variable pain responder (30%/50%) in the morning (and in the morning and evening). The treatment group is considered as the main effect and other baseline characteristics (neuropathic pain component, baseline NRS pain intensity, age, sex and country) as covariables.<br>The odds ratio of VER-01 vs. placebo including the 95% confidence interval based on the logistic regression model is regarded as the population-level summary. In order to support the proof of superiority of the investigational medicinal product, the lower limit of the odds ratio 95% confidence interval must not be less than 1.
**Intercurrent Event**	Analogous to the primary estimand of phase A. The imputations of the NRS from the primary estimand form the basis for the calculation of the responder variables, which are analysed with a logistic regression model.

For the pain responders in the morning and evening, the mean values for the run-in week (NRS_{base}) and for Week 15 (NRS_{W15}) are based on the values for the morning and evening. For the mean NRS pain intensity in the morning and evening, separate standard means for all relevant assessments in the morning and for all relevant assessments in the evening are calculated and the resulting means are averaged subsequently.

The number and percentage of pain responders (30%/50%) is summarised by treatment group and study week. Differences between the treatment groups are examined using an adequate statistical test.

9.6.3.3 Supporting estimands

Additional supporting estimands are defined for the following secondary estimands in this study:

Change from the baseline in pain intensity measured in the morning on an 11-point NRS scale for patients with a painDETECT score >18):

Two supporting estimands are defined by changing the population to patients with a PainDETECT score >15 and with a painDETECT score >12.

Change from the baseline in the NPSI total score for patients with a painDETECT score > 18 (key secondary estimand):

For this purpose, two supporting estimands are defined by changing the population to patients with a painDETECT score >15 and with a painDETECT score >12.

Pain responders in the morning and in the morning and evening:

For this, one supporting estimand each is defined, which uses the risk difference based on the calculation method of Ge et al.78 as the population-level summary instead of the odds ratio.

9.6.4 Analysis of efficacy

The endpoints described above by estimands as well as all other secondary endpoints for the efficacy analysis mentioned in Section 7.15.1.2 are analysed descriptively (see Section 9.1). For continuous endpoints, the absolute change from baseline by visit or time window is also calculated and summarised. Differences between the treatment groups are examined using appropriate statistical tests. The analysis of multidimensional scales is performed according to the specifications of the authors.

The following secondary endpoints are analysed in addition to the defined estimands in order to analyse the efficacy in Phase A.

Collected daily in the patient's diary:

- Mean NRS pain intensity in the morning, in the evening, and in the morning and evening, and change from baseline by study week

[78] Ge M, Durham LK, Meyer RD, Xie W, Thomas N. Covariate-adjusted difference in proportions from clinical trials using logistic regression and weighted risk differences. Drug Information Journal. 2011; 45(4): 481-493.

- Mean NRS pain intensity in the morning and change from baseline per study week for patients with a painDETECT score at screening >18, >15 and >12.
- Proportion of 30%/50% pain responders in the morning, in the evening, and in the morning and evening per study week
- Mean sleep quality (NRS) and change from baseline by study week
- Cumulative and relative dose of rescue medication taken in Phase A

Collected weekly in the patient's diary:

- Scores and changes from baseline of depression, anxiety, and stress: Depression-Anxiety-Stress Scales (DASS) by study week

Collected during the visits by the investigator:

- Percentage of patients by category based on the investigator's satisfaction with the treatment result per visit, 5-point Likert scale

Collected during the visits by the patient:

- Scores and changes from baseline of the individual NPSI sub scores per visit
- Percentage of patients by category based on the patient's satisfaction with the treatment result per visit, 5-point Likert scale
- Scores and changes from baseline in quality of life by visit: Short Form 36 (SF-36)
- Scores and changes from baseline of sleep quality by visit: Medical Outcomes Study Sleep Scale (MOS-SS)
- Scores and changes from baseline in bodily function, impairment due to back pain by visit, Roland Morris Disability (RMD) Questionnaire
- Percentage of patients by category of the overall assessment of symptoms by the patient by visit, patient global impression of change (PGIC), 7-point Likert scale

Further details of the analysis are defined in the statistical analysis plan.

9.6.5 Analysis of safety and tolerability

The following parameters are collected and analysed to assess the safety and tolerability in Phase A at the study visits mentioned in Table 11 (see Section 7.15.2):

- Occurrence of AEs/SAEs
- Complete blood count and clinical chemistry parameters using a whole blood sample
- 24-hour ECG (not for all study participants)

- Physical examination (lungs, heart, skin, lymph nodes, CNS)
- Vital signs (blood pressure, pulse rate, body temperature, body weight)
- Satisfaction of the patient with tolerability: 5-point Likert scale

Adverse events (AEs) are coded using the version of the MedDRA Thesaurus that was current at the start of the study; this version will be updated if necessary. The assessment of tolerability is primarily based on the frequency and severity of treatment-related adverse events (AEs). The number and percentage of patients with treatment emergent adverse events (TEAEs) is summarised together with the number of AEs by MedDRA System Organ Class and Preferred Term and by treatment group. This analysis is repeated separately for

- Serious AEs (SAEs),
- TEAEs/SAEs related to study medication,
- TEAEs leading to study discontinuation,
- TEAEs in causal connection with the study medication leading to discontinuation of the study,
- TEAEs leading to modification of the intake of the study medication (interruption, discontinuation, dose adjustment), and
- TEAEs in connection with the intake of the study medication and leading to modification of the intake of the study medication (interruption, discontinuation, dose adjustment).

To evaluate the severity and the causal relationship to the study medication of AEs, the maximum severity or the closest causal relationship per patient and class of AEs is taken into account. AEs occurring during the titration phase are presented separately. AEs that occur before the start of treatment are only listed.

Laboratory values and vital signs by time point as well as changes from baseline are analysed descriptively according to the standards for continuous parameters at the individual points in time. Furthermore, laboratory values are classified as below, within or above normal range and changes compared to baseline are presented in shift tables. The results of the ECG examination are classified by the investigator as clinically significant if applicable and changes from baseline are presented in shift tables.

The number and percentage of patients by response category of the patient's satisfaction with tolerability are summarised by visit.

9.6.6 Analysis of the potential for substance dependency and abuse

Analysis of the potential for substance dependency and abuse are carried out based on the study population SAF_A.

The Addiction Behaviors Checklist (ABC) according to Wu et al. (2006) contains 20 questions related to addiction-prone behaviour between and during visits. The absolute number of positive answers is summarised by treatment group and times of data collection.

AEs that could be related to abuse potential (e.g., euphoria-related events, attention disorders, mood swings, psychotic signs etc.) or withdrawal are identified using MedDRA Preferred Terms and are listed individually with Low Level Terms (LLT) and other information (time point of occurrence, duration, dosage of study medication, severity, outcome). All hospitalisations due to neurological or psychiatric events are examined on a case-by-case basis. Compliance data can also generate a signal for drug abuse.

9.6.7 Handling of missing data and strategies for imputation

The occurrence of intercurrent events can either prevent the collection of further data (e.g., in case of early discontinuation from study), or data collected after the occurrence of the intercurrent event cannot be considered in the statistical analysis because this would distort the interpretation of the results. If necessary, intercurrent events are handled adequately using imputation strategies so that all patients in the study population FAS_A are included in the analysis (missing data caused by the intercurrent event are imputed), or non-interpretable data are replaced (data collected after the occurrence of the intercurrent event are set to missing and imputed). An adequate imputation strategy is selected separately for every intercurrent event and is specified within the definition of estimands and related sensitivity analyses. If multiple intercurrent events occur per patient, a worst-case approach is applied, i.e. conservative imputation strategies are applied primarily.

The following imputation strategies are applied depending on the type of intercurrent event and the corresponding definitions for estimands and sensitivity analyses:

- Due to the potentially arbitrary (non-monotone) missing data pattern over time, all non-monotone missing data (e.g., due to non-compliance with eDiary completion or technical eDiary issues) is imputed using the MAR assumption first. Just enough missing data is imputed to transform the arbitrary missing data pattern into a monotone missing data pattern using the Markov Chain Monte Carlo (MCMC) method. All remaining missing data for the change from baseline in NRS pain intensity or the NPSI following a monotone missing data pattern is imputed subsequently using linear monotone regression models (either using the MAR- or the

MNAR assumption depending on which assumption is plausible). Multiple Imputation (MI): If the "Missing at random" (MAR) assumption is plausible, missing data is imputed using a multiple imputation model. At least the explanatory variables included in the regression models for the respective statistical analysis (i.e. treatment group, neuropathic pain component, baseline NRS for pain intensity or baseline NPSI total score, age, sex, and country) and the individual course of NRS pain intensity scores or NPSI total scores per patient at pre-defined time points are included in the imputation model. The pre-defined time points for the imputation of the change from baseline in NRS pain intensity at Week 15 are Weeks 3, 7 and 11. Respectively, the pre-defined time points for the imputation of the change from baseline in NPSI total score at Visit A6 are Visits A3, A4 and A5. Missing data in the individual course per patient are imputed chronologically in preliminary imputation steps depending on baseline characteristics and the preceding time points. Jump to Reference (J2R): If the "Missing not at random" (MNAR) assumption is plausible, missing data is imputed model-based using multiple imputation. Only patients from the reference group (placebo) are considered in the imputation model [Ayele et al. (2014)[78];Carpenter (2013)[79]]. Beyond that, the same explanatory variables as defined above for multiple imputation are considered.

- Baseline Observation Carried Forward (BOCF): All missing data is imputed using the baseline observation.
- Last Observation Carried Forward (LOCF): All missing data is imputed using the last collected observation.
    - Non-responder imputation: All missing data for endpoints analysing the presence of clinical response is imputed using "non-response".

Important baseline characteristics which are considered explanatory variables in the imputation models are needed for the evaluation of inclusion criteria and are available at baseline. Moreover, missing data or non-interpretable data caused by the occurrence of intercurrent events for the explanatory variables in the imputation models are imputed using multiple imputation whenever necessary.

The mean NRS pain intensity at Week 15 has to be available for the determination of the primary efficacy endpoint. The mean NRS pain intensity at baseline is needed to check the inclusion criteria and is available for all randomised patients. For the calculation of the mean pain intensity at Week 15,

78 Ayele BT, Lipkovich I, Molenberghs G, Mallinckrodt C (2014): A multiple-imputation-based approach to sensitivity analyses and effectiveness assessments in longitudinal clinical trials; Journal of biopharmaceutical statistics; 24: 211-228. doi: 10.1080/10543406.2013.859148.

79 James R. Carpenter , James H. Roger & Michael G. Kenward (2013) Analysis of Longitudinal Trials with Protocol Deviation: A Framework for Relevant, Accessible Assumptions, and Inference via Multiple Imputation, Journal of Biopharmaceutical Statistics, 23:6, 1352-1371, DOI: 10.1080/10543406.2013.834911

the NRS pain intensity has to be documented in the diary on at least four out of the seven days of study Week 15. The mean pain intensity in Week 15 is considered missing if fewer than four diary entries are available and, in this case, is imputed using the multiple imputation model specified above. The key-secondary endpoint is the change from baseline in the NPSI total score at Visit A6 and can only be determined if the NPSI at Visit A2 and A6 is fully completed. If the NPSI was not filled in completely at Visit A2, available answers for the NPSI filled in at Visit A1 are considered for the calculation of the total score at baseline. If the NPSI at Visit A1 was also not filled in (completely), the NPSI total score at baseline is imputed using multiple imputation depending on baseline characteristics as described above. Missing NPSI total scores at Visit A6 are imputed using the multiple imputation model specified above.

Another secondary endpoint is the change from baseline in pain intensity at Week 15 measured on the 11-point NRS in the morning and evening. The mean pain intensity in the morning and evening at Week 15 and at baseline needs to be available to determine this endpoint. For the calculation of the respective means, the NRS pain intensity has to be documented in the diary on at least four out of seven days in Week 15 in the morning and in the evening respectively and on at least five out of seven days in the morning and in the evening respectively for baseline (within the 7 days prior to Visit A2). If fewer diary entries are available in the relevant weeks, the values are considered as missing and are imputed using the multiple imputation models specified above.

Further details of the imputation strategies will be specified in the statistical analysis plan.

All other missing data will not be replaced.

9.7 Phase B: Analysis of open-label extension

This study phase primarily serves to demonstrate long-term safety and to examine the potential for substance dependency and abuse over 29 weeks.

9.7.1 Study populations

The following study population is used for the analysis.

Safety Analysis Set of Phase B (SAF_B):

The Safety Analysis Set (SAF_B) contains all patients who were included in Phase B and who took at least one dose of the study medication in Phase B.

Safety Analysis Set Phase B for first analysis (SAF_B1):

SAF_B1 is a subset of SAF_B and contains all patients included in Phase B, who took at least one dose of the study medication in Phase B and who had completed or prematurely discontinued Phase B at the time of the first analysis.

All data collected for patients who were included in Phase B but did not take any study medication are listed separately.

9.7.2 Analysis of safety / long-term safety

The analysis of the safety parameters for the study population SAF_B is carried out in the same way as described for Phase A in Section 9.6.5.

As the primary analysis in Phase B, the number and percentage of patients for whom an AE occurs in Phase B that is related to VER-01 from the investigator's point of view is summarised together with the number of AEs by MedDRA System Organ Class and Preferred Term. In a further analysis, the maximum severity of these AEs per patient and MedDRA Preferred Term is additionally taken into account.

9.7.3 Analysis of the potential for substance dependency and abuse

The analysis of the potential for substance dependency and abuse is carried out analogously to Phase A as described in Section 9.6.6.

9.7.4 Analysis of efficacy

The efficacy parameters are evaluated in the same way as for Phase A as described in Section 9.6.4 based on the study population SAF_B. In open label Phase B, a real comparison group is missing; therefore, the open label treatment is also depicted separately for the order of treatment based on the randomised treatment in Phase A (VER-01 to VER-01 or placebo to VER-01). The efficacy parameters are evaluated purely descriptively by time point/time frame/visit and, if necessary, further differentiations. Endpoints for which estimands were defined in Phase A are descriptively analysed as well in Phase B according to the standards for continuous or categorical endpoints. Changes from baseline relate to the baseline values of Phase A. A descriptive depiction of the results is provided per visit (for visit-based endpoints) and per study week (for diary-based endpoints).

9.7.5 Handling of missing data and strategies for imputation

All data will be evaluated as collected and missing data will not be replaced.

The individual responder status at the end of Phase B is determined based on the change from baseline in pain intensity (NRS) and decides whether the patient may be included in Phase D. Therefore, at least four out of seven diary entries must be observed within study Week 43. If fewer than four diary entries

are available, the patient is classified as non-responder and can only be assigned to Phase C. However, the missing values are not imputed for the statistical analysis.

9.8 Phase C: Analysis of open-label extension

This study phase is the continuation of the open label treatment of Phase B for an additional 26 weeks.

9.8.1 Study populations

The following study population is used for the analysis.

Safety Analysis Set of Phase C (SAF_C):

The Safety Analysis Set (SAF_C) contains all patients who were included in Phase C and who took at least one dose of the study medication in Phase C.

All data collected for patients who were included in Phase C but did not take any study medication are listed separately.

All descriptive analyses are presented for the entire SAF_C population.

9.8.2 Analysis of safety/long-term safety

The analysis of the safety parameters for the study population SAF_C is carried out in the same way as described for Phase A in Section 9.6.5.

As the primary analysis in Phase C, the number and proportion of patients in the study population SAF_C, for whom an AE occurs in Phases B and C that is related to VER-01 from the investigator's point of view is summarised together with the number of AEs by MedDRA System Organ Class and Preferred Term. In a further analysis, the maximum severity of these AEs per patient and MedDRA Preferred Term is additionally taken into account. In addition, study phases B and C are considered separately for all patients in the SAF_C study population.

9.8.3 Analysis of the potential for substance dependency and abuse

The analysis of the potential for substance dependency and abuse is carried out in the same way as for Phase A as described in Section 9.6.6.

In Phase C, the investigator also evaluates the substance dependency with a possible diagnosis (F12.2 according to ICD-10). The absolute number of positive answers according to the German Bundesärztekammer (2007) is summarised descriptively according to the standard for continuous parameters.

9.8.4 Analysis of effects of sudden drug withdrawal

During the wash-out phase of Phase C, the symptoms of drug withdrawal based on the Cannabis Withdrawal Scale (CWS) are documented additionally on a daily basis. Sum scores for intensity and functional impairment are descriptively analysed according to Allsop et al.

Moreover, AEs documented during the wash-out phase are summarised separately.

9.8.5 Analysis of efficacy

The efficacy parameters are analysed in the same way as for Phase A as described in Section 9.6.4 based on the study population SAF_C. For this purpose, all data collected between visit B7 and visit C13 are considered. In the open label Phases B and C, a real comparison group is missing, therefore the open label treatment is also depicted separately for the order of treatment based on the randomised treatment in Phase A (VER-01 to VER-01 or placebo to VER-01). The efficacy parameters are analysed purely descriptively according to time point/ time frame/ visit and, if necessary, further differentiations. Endpoints for which estimands were defined in Phase A are descriptively analysed as well in Phase C according to the standards for continuous or categorical endpoints. Changes from baseline refer to the baseline values of Phase A. A descriptive depiction of the results is provided per visit (for visit-based endpoints) and per study week (for diary-based endpoints).

9.8.6 Handling of missing data and strategies for imputation

All data will be evaluated as collected and missing data will not be imputed.

9.9 Phase D: Analysis of maintenance of efficacy

The placebo-controlled proof of a sustained therapeutic effect of VER-01 after treatment with VER-01 for six months is the primary goal of the placebo-controlled continuation of treatment with VER-01 in Phase D.

9.9.1 Study populations

The following study populations are used for the analysis.

Full Analysis Set Phase D (FAS_D):

The data set used for efficacy analysis is the Full Analysis Set (FAS_D). This data set contains all patients who were randomised to Phase D and who took at least one dose of the study medication in Phase D. The patients are assigned to the treatment group to which they were randomised.

Full Analysis Set Phase D for patients with a neuropathic pain component (FAS_D_NE):

The assessment dataset is used for the analysis of the NPSI and contains all patients of the FAS_D having a painDETECT score >18 at the screening visit.

Safety Analysis Set Phase D (SAF_D):

The analysis data set Safety Analysis Set (SAF_D), which is used for the safety and tolerability analysis, contains all randomised patients who took at least one dose of the study medication in Phase D. The patients are assigned to the respective treatment group according to the treatment actually taken.

9.9.2 Estimand of the primary endpoint in Phase D

As described in Section 7.15.1.1, the primary endpoint in Phase D is the time to treatment failure defined as the time in days from randomisation to Phase D (R2) until the first day of treatment failure. The estimand for the time to treatment failure can be defined as a de facto estimand in the same way as the estimands defined for Phase A. Hence, the estimated treatment effect should correspond to the treatment effect expected in clinical practice.

Table 16: Estimand of the primary endpoint in Phase D

Attribute	Definition
**Population**	Patients with non-specific chronic low back pain (CLBP) of at least moderate intensity defined by the inclusion and exclusion criteria for Phase D, who were randomised to either treatment with VER-01 or placebo and who took at least one dose of study medication (FAS_D, as described in Section 9.9.1) after long-term treatment with VER-01 for at least 29 weeks.
**Treatment**	VER-01 vs. placebo,<br>each including permitted rescue medication as well as any overdosage of study medication (see Table 12).
**Variable**	Time in days from randomisation to Phase D to the first day of treatment failure. Treatment failure is assessed by calculating the seven-day mean of the NRS pain intensity in the morning during the treatment phase on a daily basis which needs to increase by at least 20% and at least 1 point compared to baseline (mean of study Week 43). The first day within this seven-day window for which this criterion is fulfilled is subsequently defined as the first day of treatment failure. The mean can be calculated if not more than three days are missing within the seven-day time window; these missing days do not need to be imputed and can therefore not be selected as days of treatment failure.

**Population -level summary**	Cox model-based hazard ratio of VER-01 vs. placebo including 95% confidence interval. Superiority of VER-01 over placebo is shown if the upper bound of the confidence interval is <1.
**Intercurrent events**	The following strategies are applied for intercurrent events specified in Table 12.<br>1. Early discontinuation from study due to intolerability: Treated as event<br>2. Early discontinuation from study due to lack of efficacy: Treated as event<br>3. Early discontinuation from study due to other reasons: Treated as censoring in Cox model<br>4. Overdosage of allowed rescue medication: Treated as event<br>5. Non-ignorable intake of prohibited concomitant medication: Treated as event<br>6. Ignorable intake of prohibited concomitant medication: Multiple imputation under MAR assumption<br>7. Ignorable start/adaptation of non-drug pain therapy: Multiple imputation under MAR assumption<br>8. Non-compliance with dosage of study medication: Treated as event in case of non-ignorable non-compliance (permanent discontinuation of study medication); applying the treatment policy strategy and using the data as collected in case of irrelevant non-compliance (any overdosage of study medication).

The time to treatment failure is analysed in terms of a Cox model for the primary analysis. Events of treatment failure are defined according to the variables defined above. The occurrence of specific intercurrent events is treated as an event or censoring as described above. If multiple events or censoring occur for the same patient at different time points, the first event is analysed.

All patients who did not experience treatment failure until the end of Phase D are censored at this time point. In the Cox model, treatment group is considered as the main effect and the baseline characteristics of neuropathic pain component, baseline NRS pain intensity for Phase D, age and sex are considered covariables.

As part of sensitivity analyses, the two intercurrent events ignorable intake of non-permitted concomitant medication and ignorable start/adjustment of non-drug pain therapy are evaluated as non-ignorable and evaluated as treatment failure events.

9.9.3 Analysis of maintenance of efficacy

The number and percentage of patients with treatment failure is summarized by treatment group.

Moreover, the time to treatment failure in the treatment groups is presented using Kaplan-Meier plots. Supportive statistics such as the median time to treatment failure per treatment group are additionally displayed. Further details will be specified in the SAP.

All further efficacy endpoints are analysed as defined for Phase A in Section 9.6.4 based on the study population FAS_D or FAS_D_NE. All efficacy endpoints for which estimands were defined in Phase A are descriptively analysed as well in Phase D according to the standards for continuous or categorical endpoints. Due to the inclusion criteria in Phase D, the analysis of pain responders is only performed for the response definition of 30%. Baseline for Phase D refers to the time of randomisation to Phase D at Visit B10; baseline NRS for Phase D refers to study week 43.

9.9.4 Analysis of safety

All safety parameters are analysed based on the study population SAF_D as defined for Phase A in Section 9.6.5.

9.9.5 Analysis of the potential for substance dependency and abuse

The potential for substance dependency and abuse is analysed based on the study population SAF_D as defined for Phase A in Section 9.6.6.

In Phase D, the investigator also evaluates the substance dependency with a possible diagnosis (F12.2 according to ICD-10). The absolute number of positive answers according to the German Bundesärztekammer (2007) is summarised descriptively according to the standard for continuous parameters.

9.9.6 Analysis of effects of sudden drug withdrawal

During the treatment phase and the wash-out phase of Phase D, the symptoms of drug withdrawal based on the Cannabis Withdrawal Scale (CWS) are documented on a daily basis. Sum scores for intensity and functional impairment are descriptively analysed according to Allsop et al. based on the study population SAF_D.

Moreover, AEs documented during the wash-out phase are summarised separately.

9.9.7 Handling of missing data and strategies for imputation

Missing data of the primary endpoint of time to treatment failure is imputed in order to include all patients of the study population FAS_D in the analysis. Missing data due to the occurrence of specific intercurrent events or collected data that cannot be used for the statistical analysis due to the occurrence of specific intercurrent events does not have to be imputed, but they are considered events or

censoring in the framework of the time-to-event analysis (details are given in Section 9.9.2). An exception applies to the intercurrent event "ignorable start/adaptation of non-drug pain therapy" and the intercurrent event "ignorable intake of prohibited concomitant medication". All diary entries collected after the occurrence of these intercurrent events are considered missing. The imputation strategy for missing diary data is described in detail below.

For the calculation of the seven-day means, at least four out of seven diary entries must be available. If missing diary entries preclude the analysis of the primary endpoint, the seven-day means (also termed as *moving averages*) are imputed under the "missing at random" (MAR) assumption using multiple imputation. At least the covariables used in the corresponding analysis model (i.e. treatment group, neuropathic pain component, baseline NRS pain intensity for Phase D, age, sex) and the preceding seven-day mean are considered in the imputation model. This is the seven-day mean which starts one day earlier and can be calculated or was imputed previously (also depending on the baseline characteristics and the preceding seven-day mean). The seven-day mean based on the time window starting with Day 1 is imputed depending only on baseline characteristics if necessary. Moreover, seven-day means only need to be imputed if the patient in question did not discontinue early from study before. If the criterion of worsening from baseline is fulfilled for an imputed value, the first missing day within the time window is only considered as the first day of treatment failure if none of the observed values within the time window fulfils the criterion.

The imputation model has to be specified using fully conditional specification methods due to the non-monotone pattern of missing data over time; thus, the FCS statement will be used when specifying SAS code for the MI procedure. The change from baseline is imputed using regression models. Further details will be specified in the SAP. All other missing data will not be imputed.

9.10 Analysis of prior and concomitant medication

Prior and concomitant medications are coded according to the WHO Drug Global Dictionary and summarised with their absolute and relative frequencies at least by ATC level 2 and treatment group. For Phases A to D, therapies are defined as concomitant if at least one dose of the concomitant medication was taken while taking the study medication.

9.10.1 Analysis of rescue and concomitant pain medication

Concomitant pain medication is summarised separately for each study phase based on the respective study populations.

Concomitant rescue medication (see Section 7.7) is summarised separately for each study phase based on the respective study populations. The number of rescue medication taken is listed in a table by

treatment group for each study phase. As an additional endpoint for all Phases, the cumulative dose of rescue medication taken and the relative cumulative dose of rescue medication in relation to the individual study phase duration respectively, is summarised by treatment group.

9.10.2 Analysis of non-drug therapies

Non-drug therapies (e.g., physical or behavioural therapy, relaxation methods such as progressive muscle relaxation, rehabilitation exercises and functional training, acupuncture, occupational therapy, manual therapy such as manipulation/mobilisation, massage, back training, thermotherapy), which significantly modulate pain perception, are analysed separately for each study phase. Details are specified in the statistical analysis plan. These therapies are coded using the MedDRA Thesaurus.

9.11 Analysis of treatment exposure

Treatment exposure is analysed separately for each study phase based on the respective safety analysis set.

The amount of administered study medication is summarised in total by treatment group and includes the cumulative dose actually taken in mg. The titration phase is summarised separately in Phases A and B.

9.12 Interim analyses

No formal interim analysis is planned for this study. The database is locked for the analysis of the proof of efficacy and maintenance of efficacy when all randomised patients have completed Phases A and D or prematurely terminated the study. At the same time, a first analysis of long-term safety is carried out at this time based on all patients who have completed phase B at that time or who have terminated the study prematurely. Prior to database lock, all respective data was collected and cleaned and a final assessment of the blinded data (Blinded Data Review) has taken place. A second and final database lock is carried out after all patients have completed Phase C or have prematurely terminated the study and all collected data has been cleaned.

10. Changes to the protocol

To ensure largely comparable conditions at all study sites as well as in the interests of flawless data evaluation, there is no intention for any amendments to be made to the trial protocol. In exceptional cases however, changes to provisions in the trial protocol are possible. They only take place following mutual agreement between the sponsor, the NCI, and the statistician, as well as all signatories (authors) of this protocol. Any change to the study procedures set out in the protocol must be made in writing, specifying the reasons in question, and signed by all authors of this protocol (amendment).

In accordance with national regulations, substantial amendments that require authorisation are submitted by the sponsor to the institutional review board/independent ethics committee and the national authorities for approval and are only implemented once they have been approved. An exception is made for changes required to avert imminent danger.

10.1 Revisions

Revisions are listed in tabular form in Appendix 3.

11. Ethical and regulatory aspects

11.1 Legal regulations observed, guidelines

This clinical trial is conducted in compliance with the published principles of the Good Clinical Practice guideline (ICH-GCP) and the applicable legal provisions listed under Chapter 7.1. These principles include inter alia institutional review board/independent ethics committee processes, patient information and informed consent form, following the protocol, administrative documents, documentation of IMPs, data collection, patient fields (source documents), recording and reporting adverse events (AE), preparation for inspections and audits as well as storage of documents. All investigators and other staff directly involved in the trial will be informed that domestic and foreign monitoring authorities, the responsible federal authorities and personnel authorised by the sponsor of the clinical trial are entitled to view study documents and patient files at any time. The sponsor assures that the clinical trial is to be conducted in compliance with the approved protocol according to EMA/CHMP/ICH/135/1995 ICH E6[R2] 6.2.5 and Regulation (EU) No. 536/2014, respectively, and with the principles of GCP.

11.2 Reports to authorities, approvals, and registration

Before the start of the clinical trial, the relevant documents are submitted to the national authorities of the participating countries for approval. The sponsor has selected a central ethics committee from each participating country for this multicenter study in accordance with country-specific legal regulations. If required by country-specific legal regulations, the sponsor will provide the institutional review boards/independent ethics committees, which would be locally responsible for reviewing the proposed study sites in the case of a monocentric clinical trial, with all documents along with the application to the central ethics committee.

In addition, the import and export of the cannabis product are in accordance with the country-specific legal regulations. An import application/reporting decree is sent to the responsible authorities.

Before the start of the clinical trial, it is registered with the study register recognised by the WHO ClinicalTrials.gov (ClinicalTrials.gov Identifier: NCT04940741).

11.3 Subject insurance

Subject insurance is taken out for all enrolled patients in accordance with ICH-GCP Guidelines. Registered office, policy no., telephone and fax number of the insurance company are included in the patient information (see patient information).

Patients are informed in detail about the insurance in the patient information.

11.4 Institutional review boards/independent ethics committees

The sponsor is responsible for the application for a favourable opinion by an institutional review board/independent ethics committee formed in accordance with national law. The sponsor is authorised by the Principal Coordinating Investigator to submit an application for a favourable opinion to the central ethics committee. The clinical trial will only begin once a favourable opinion has been obtained from the central ethics committee. In every additional study site, the clinical trial is only conducted once the central ethics committee - and, if required by country-specific legal regulations, in consultation with the locally involved ethics committees - has determined the suitability of the study site and the qualifications of the investigators.

11.5 Ethical conduct of the clinical trial

This protocol and any subsequent amendments to the protocol were or are applied in accordance with the Declaration of Helsinki, in the most recently amended version of October 2013 (Fortaleza), in accordance with Regulation (EU) 536/2014.

11.6 Data protection

The provisions of the relevant national and European data protection laws are observed. It is ensured that all examination materials and data are suitably anonymised before scientific evaluation in accordance with the relevant data protection provisions.

Organizational and technical measures are taken to prevent unauthorized access, disclosure, dissemination, alteration or loss of information and processed personal data. An explicit description of the organizational and technical measures to comply with the applicable regulations on the protection of personal data are listed in Appendix 4 I. A description of the rules for the timely and efficient handling of a data breach in order to limit or prevent damage is provided in Appendix 4 II.

12. Use of data and publication

12.1 Final report

The relevant authorities and the institutional review board/independent ethics committee are informed of the end of the clinical trial within 90 days after the regular end of the study and within 15 days in the case of premature termination.

Within a year after the end of the clinical trial, the responsible national competent authority and the institutional review board/independent ethics committee will be sent the summary of the final report for the clinical trial, containing all significant events during the clinical trial.

12.2 Publication

It is intended for the results of the clinical trial to be published in a scientific journal in due course and/or to be presented at international conferences. In principle, the publication of the entire clinical trial is to be prioritised. The “Uniform requirements for manuscripts submitted to biomedical journals of the International Committee of Medical Journal Editors (ICMJE) (JAMA 1997;277:927-34) are observed.

The sponsor has the right of first publication of the study results, regardless of the method of publication. Publications are oral or written publications relating to the clinical trial, its results, or the investigational medicinal products.

The study site/principal investigator has the right to publish the data and results that arise during the conduct of the trial at the study site after the initial publication has been made by the sponsor, or if such initial publication is not made at least 12 months after the completion of the data analysis by the sponsor. The same applies to the right of the study site to use the results for internal, non-commercial research and teaching purposes.

The study site/principal investigator will send the planned publication to the sponsor for review at least 30 days prior to submission to the publisher. Within this period, the sponsor may request the removal of confidential information (except for data and results obtained from the conduct of the study). The sponsor may also request that publication be delayed for an additional 60 days if this is necessary to protect its rights to the results.

For all publications by the sponsor, data protection must be ensured for all personal data of the patients concerned as well as the personal data of the participating investigators.

It is also intended for the clinical trial to be registered in a public register in line with ICMJE recommendations (see also Chapter 11.2).

By signing the statement of participation, the investigator declares their agreement that the results of this clinical trial can be submitted to national and international competent authorities, the German Medical Association, the National Association of Statutory Health Insurance Physicians and the medical insurance companies. At the same time, the investigator declares their consent to their name, address, qualifications, and the extent of their involvement in the clinical trial being disclosed in this respect.

13. Bibliography

13.1 Laws and guidelines

- The European Agency for the Evaluation of Medicinal Product. Note for Guidance on Good Clinical Practice (CPMP/ICH/135/95).
- Clinical trial registration: a statement from the International Committee of Medical Journal Editors. Accessed at http://www.icmje.org/clin_trial.pdf on 22 May 2007.
- The European Agency for the Evaluation of Medicinal Product. Note for Guidance Structure and Content of Clinical Study Reports (CPMP/ICH/137/95).
- WHO. Causality Assessment of Suspected Adverse Reactions. http://www.who-umc.org/DynPage.aspx?id=22682
- the ICH-GCP Guideline on Good Clinical Practice CPMP/ICH/135/95 (GCP Guideline)
- Declaration of Helsinki in the 1996 version (ICH Topic E6, Guideline for GCP) with the revisions of Edinburgh (2000), the clarifications of Washington (2002) and Tokyo (2004), as well as the revisions of Seoul (2008) and Fortaleza (2013),
- Regulation on the application of Good Clinical Practice in the conduct of clinical trials with medicinal products for human use (GCP Regulation - GCP-V),
- Regulation (EU) No. 536/2014 of the European Parliament and of the Council of 16 April 2014 on clinical trials on medicinal products for human use and repealing Directive 2001/20/EC
- Directive 2001/83/EC of the European Parliament and of the Council of 6 November 2001 on the Community code relating to medicinal products for human use
- Directive 2003/94/EC laying down the principles and guidelines of good manufacturing practice in respect of medicinal products for human use and investigational medicinal products for human use,
- Directive 2005/28/EC laying down principles and detailed guidelines for good clinical practice as regards investigational medicinal products for human use, as well as the requirements for authorisation of the manufacturing or importation of such products
- the detailed guidance of the European Commission on the handling of adverse drug reactions reports from clinical trials of April 2004 with the revision of 2006 (ENTR/CT3)
- Regulation (EU) 2016/679 of the European Parliament and of the Council of 27 April 2016 on the protection of individuals with regard to the processing of personal data (EU-GDPR)
- country-specific legal regulations (in particular the Medicines Act of the countries involved in the study),

- as well as other applicable legal regulations in the currently valid version.

14. Measures in the event of the regular conduct of the study being disrupted due to COVID-19

14.1 Carrying out patient visits

Should it not be possible to hold a visit on site as a result of COVID-19 (e.g. as a result of a patient being in quarantine), the visit must take place virtually (as a telephone or video visit), with the exception of the following visits:

- **Visit A1:** it is not possible for the screening visit to be a virtual visit. The visit must be postponed.
- **Visit A2:** if the visit cannot take place on site within the intended time frame (8+3 days after Visit A1), the visit can be postponed by a maximum of one week (+ max. 7 days). If the patient cannot attend Visit A2 on site within the prolonged period, this must be documented as a screen failure and if applicable the run-in phase can be repeated as part of re-screening.
- **Visit A6, B10, D12, C14, follow-up:** it is not possible to hold these visits virtually and the visit must be postponed to the earliest possible time. In the meantime, an unscheduled virtual visit should be performed, however, (e.g. to ask about AEs/SAEs).

As part of the virtual visit, all activities planned for the visit that do not require the patient's physical presence will be performed. Visit content that requires a patient to be present in person includes study-specific tests (physical examinations, measurement of vital signs, drug tests, pregnancy tests, blood draws, ECG) and return and dispensing of the investigational medicinal product and rescue medication.

Should it be necessary to take back/dispense investigational medicinal product and/or rescue medication before the next on-site visit takes place in order to guarantee the patient's treatment, the patient can authorise a trusted person to be their representative. This authorisation must authorise the representative to receive the investigational medicinal product and/or the rescue medication from the study site and then hand it over to the patient. The study site should check the identity of the representative against the authorisation before taking back/dispensing investigational medicinal product and/or rescue medication and should issue a receipt of dispensing of investigational medicinal product and/or rescue medication to the representative. In addition, receipt of the investigational medicinal product/rescue medication by the patients should be confirmed via telephone. The process must be clearly documented in the patient file.

14.2 Carrying out monitoring visits

If an on-site monitoring visit is not possible as a result of COVID-19, remote monitoring visits should take place until the next on-site monitoring visit. Further details are described in the monitoring plan.

Remote source data verification cannot take place.
