# Supplementary material for: Full-spectrum extract from Cannabis sativa DKJ127 for chronic low back pain: a phase 3 randomized placebo-controlled trial
Source: Nat Med. 2025 Sep 29;31(12):4189–96. doi: 10.1038/s41591-025-03977-0 (PMC12705446; doi:10.1038/s41591-025-03977-0)
Supplement: Supplementary file 2 — Reporting Summary [file 41591_2025_3977_MOESM2_ESM.pdf]

nature portfolio

Corresponding author(s): Prof. Matthias Karst, MD

Last updated by author(s): Aug 7, 2025

Reporting Summary

Nature Portfolio wishes to improve the reproducibility of the work that we publish. This form provides structure for consistency and transparency in reporting. For further information on Nature Portfolio policies, see our Editorial Policies and the Editorial Policy Checklist.

Statistics

For all statistical analyses, confirm that the following items are present in the figure legend, table legend, main text, or Methods section.

n/a	Confirmed	
☐	☒	The exact sample size (*n*) for each experimental group/condition, given as a discrete number and unit of measurement
☒	☐	A statement on whether measurements were taken from distinct samples or whether the same sample was measured repeatedly
☐	☒	The statistical test(s) used AND whether they are one- or two-sided<br>*Only common tests should be described solely by name; describe more complex techniques in the Methods section.*
☐	☒	A description of all covariates tested
☐	☒	A description of any assumptions or corrections, such as tests of normality and adjustment for multiple comparisons
☐	☒	A full description of the statistical parameters including central tendency (e.g. means) or other basic estimates (e.g. regression coefficient) AND variation (e.g. standard deviation) or associated estimates of uncertainty (e.g. confidence intervals)
☐	☒	For null hypothesis testing, the test statistic (e.g. *F*, *t*, *r*) with confidence intervals, effect sizes, degrees of freedom and *P* value noted<br>*Give P values as exact values whenever suitable.*
☒	☐	For Bayesian analysis, information on the choice of priors and Markov chain Monte Carlo settings
☐	☒	For hierarchical and complex designs, identification of the appropriate level for tests and full reporting of outcomes
☒	☐	Estimates of effect sizes (e.g. Cohen's *d*, Pearson's *r*), indicating how they were calculated

Our web collection on statistics for biologists contains articles on many of the points above.

Software and code

Policy information about availability of computer code

Data collection	Clincase by Quadratek Data Solutions, version 2.7, was used to capture and store subject clinical data.
Data analysis	Analyses were performed with SAS software, version 9.4 (SAS Institute).

For manuscripts utilizing custom algorithms or software that are central to the research but not yet described in published literature, software must be made available to editors and reviewers. We strongly encourage code deposition in a community repository (e.g. GitHub). See the Nature Portfolio guidelines for submitting code & software for further information.

Data

Policy information about availability of data

All manuscripts must include a data availability statement. This statement should provide the following information, where applicable:

- Accession codes, unique identifiers, or web links for publicly available datasets
- A description of any restrictions on data availability
- For clinical datasets or third party data, please ensure that the statement adheres to our policy

The study protocol is provided in the Supplementary Information. Access to anonymized individual data and blank case report forms that underlie the results reported in this article can be requested by qualified researchers for academic purposes. Vertanical provides access within 3 months following review and approval of a research proposal, statistical analysis plan, and execution of a data access agreement. Data are available to request after the indication studied has been

approved in the USA and the European Union for a period of 5 years. Data will be shared through a secure online platform. Submit requests via https://vertanical.com/.

Research involving human participants, their data, or biological material

Policy information about studies with human participants or human data. See also policy information about sex, gender (identity/presentation), and sexual orientation and race, ethnicity and racism.

Reporting on sex and gender	Self-reported sex of the study participants is presented in the demographics table. Results of sex-treatment interaction analysis has been included in the Appendix and the Results section. Gender was not recorded in the study and no adjacent analyses performed.
Reporting on race, ethnicity, or other socially relevant groupings	The race of study participants is reported as part of the demographic and baseline characteristics, which are presented for each study phase in the main manuscript or the extended data.
Population characteristics	Mean age was 52 years. 57% of participants were of female sex. Mean BMI was 29. Included were participants with non-specific chronic low back pain, with or without a neuropathic pain component and indicated drug treatment. Details on patient demographics and baseline characteristics across the different study phases are provided in the manuscript and extended data.
Recruitment	Patients were primarily recruited via notices in doctors' offices, adverts in magazines, and online advertising. In addition, investigators participating in the study recruited patients from their databases. The sponsor was not involved in subject selection.
Ethics oversight	The trial was approved by Ethics Committees in each country and written informed consent was obtained from all participants. The trial was authorised by all relevant national competent authorities (Austria, Germany) and registered with EudraCT (2020-000107-36). A full list of involved ethics committees is provided in the appendix.

Note that full information on the approval of the study protocol must also be provided in the manuscript.

Field-specific reporting

Please select the one below that is the best fit for your research. If you are not sure, read the appropriate sections before making your selection.

☒ Life sciences ☐ Behavioural & social sciences ☐ Ecological, evolutionary & environmental sciences

For a reference copy of the document with all sections, see nature.com/documents/nr-reporting-summary-flat.pdf

Life sciences study design

All studies must disclose on these points even when the disclosure is negative.

Sample size	To demonstrate superiority of VER-01 over placebo for the primary endpoint in phase A, a total of 732 participants had to be randomized in a 1:1 ratio. The sample size calculation was based on an assumed treatment difference of 0.6 NRS points, a standard deviation (SD) of 2.5 points, a 2-sided significance level of 5% and 90% statistical power. For the key secondary endpoint of phase A, 180 participants with a neuropathic pain component had to be randomized in a 1:1 ratio to show superiority of VER-01 vs. placebo. This calculation was based on an assumed treatment difference of 10.5 NPSI points, a SD of 25.0, a 2-sided significance level of 5% and 80% statistical power. Based on a proportion of 22% of participants with a neuropathic pain component, these assumptions resulted in a total sample size of 808 participants for phase A.
Data exclusions	No data were excluded.
Replication	Predefined study plans including protocol and statistical analysis plan, together with rigorous GCP oversight both from authorities and the sponsor ensure replicability of the results. A follow-up trial comparing VER-01 with opioids confirms the significant efficacy and favourable safety results reported for VER-01 in this trial.
Randomization	The study included two randomizations, conducted at the beginning of phases A and D. Eligible subjects were randomly assigned in a 1:1 ratio to VER-01 or placebo. Randomization was stratified according to presence of a neuropathic pain component. Randomization took place in blocks of four, using a computer-generated randomization list. The size of the randomization blocks was not disclosed to the investigators.
Blinding	Participants, investigators, and study site personnel were masked to treatment assignment. VER-01 and the matching placebo were dispensed in identical amber glass bottles.

Reporting for specific materials, systems and methods

We require information from authors about some types of materials, experimental systems and methods used in many studies. Here, indicate whether each material, system or method listed is relevant to your study. If you are not sure if a list item applies to your research, read the appropriate section before selecting a response.

Materials & experimental systems

n/a	Involved in the study
☒	☐ Antibodies
☒	☐ Eukaryotic cell lines
☒	☐ Palaeontology and archaeology
☒	☐ Animals and other organisms
☐	☒ Clinical data
☒	☐ Dual use research of concern
☒	☐ Plants

Methods

n/a	Involved in the study
☒	☐ ChIP-seq
☒	☐ Flow cytometry
☒	☐ MRI-based neuroimaging

Clinical data

Policy information about clinical studies
All manuscripts should comply with the ICMJE guidelines for publication of clinical research and a completed CONSORT checklist must be included with all submissions.

Clinical trial registration: ClinicalTrials.gov identifier: NCT04940741

Study protocol: Attached as supplementary information.

Data collection: 66 outpatient sites and university-based hospitals in Germany and Austria. Enrollement took place between July 7, 2021, and June 30, 2023. The last visit of the study was on March 26, 2024.

Outcomes:

The primary endpoint in phase A was the change from baseline (Week -1) to Week 15 in the mean weekly pain intensity in the morning, measured on an 11-point NRS (0 = "no pain" and 10 = "worst pain imaginable"). The key-secondary endpoint in phase A was the change from baseline (CFB) in the Neuropathic Pain System Inventory (NPSI) total score at the end of phase A (visit A6) for participants with a neuropathic pain component. The NPSI includes 10 items measuring the severity of spontaneous pain (4 items), painful attacks (3 items), provoked pain (2 items), and abnormal sensations (1 item). Each item was scored on an 11-point NRS. The total score ranges from 0 (no symptoms) to 100 (worst imaginable symptoms).
No primary endpoint was defined for the open-label study phases B and C. The primary endpoint of phase D was the time to treatment failure, defined as an increase in the 7-day mean of NRS morning pain intensity by ≥20% and ≥1 point compared to phase D baseline (week 27).
Across all study phases, the proportion of participants achieving ≥30% and ≥50% pain reduction was evaluated, reflecting outcomes widely considered clinically meaningful.
Additional diary-based secondary efficacy endpoints assessed in all study phases included: change in mean pain intensity (11-point NRS), intake of rescue medication, sleep quality on a 11-point NRS (0 = “not impacted”; 10 = “completely impacted“), as well as the proportion of participants with ≥30% and ≥50% sleep quality improvement.
Visit-based secondary efficacy endpoints assessed in all study phases included CFB in NPSI total scores, Patient Global Impression of Change (PGIC) on a 7-point Likert scale using the question “How is your low back pain in comparison to before participation in the study?” (0 = “very much better” to 6 = “very much worse”), and quality of life evaluated with the Short Form Health Survey 36v2 (SF-36). The SF-36 contains 36 questions about physical and mental wellbeing and was evaluated based on 8 domain scores (physical functioning, role-physical, bodily pain, general health, vitality, social functioning, role-emotional, and mental health) which were summarized to form 2 higher-ordered component summary measures (Physical Health Component Summary [PCS] and the Mental Health Component Summary [MCS]). The scores ranged from 0 (maximum disability) to 100 (no disability).
The following visit-based secondary efficacy endpoints were additionally assessed in study phase A: the degree of disability was evaluated with the Roland Morris Disability Questionnaire (RMDQ) at baseline and end of treatment. The RMDQ is a 24-item questionnaire which evaluates how low back pain affects functional activities, with scores ranging from 0 to 24, where higher scores indicate greater disability. In addition, sleep quality was measured using the Medical Outcomes Study Sleep Scale (MOS-SS), which consists of 12 items that assess perceived initiation and maintenance of sleep, respiratory problems during sleep, sleep duration, perceived adequacy of sleep and daytime somnolence. The evaluation of the MOS-SS was based on 2 sleep problems indices. Higher sleep scores indicate a clinically favorable outcome.
Safety was assessed based on the incidence of adverse events, including their seriousness, severity and relationship to study drug. In addition, patients’ satisfaction with tolerability was evaluated visit-based using a 5-point Likert scale. Substance dependence and abuse potential were measured visit-based with the Addiction Behavior Checklist (ABC), a 20-item checklist used to monitor sings of addiction. An ABC sum score of 3 or more indicates inappropriate drug use. Withdrawal symptoms were recorded daily in the eDiary with the CWS during treatment in phase D and during wash-out in phase C and D. The CWS consists of 19 items representing potential withdrawal symptoms associated with cannabis withdrawal. Each item is rated on a 0 to 10 scale, where 0 indicates "not at all" and 10 indicates "extremely".

Plants

Seed stocks	N/A
Novel plant genotypes	N/A
Authentication	N/A
